# *Porphyromonas gingivalis* Provokes Exosome Secretion and Paracrine Immune Senescence in Bystander Dendritic Cells

**DOI:** 10.3389/fcimb.2021.669989

**Published:** 2021-06-01

**Authors:** Ranya Elsayed, Mahmoud Elashiry, Yutao Liu, Ahmed El-Awady, Mark Hamrick, Christopher W. Cutler

**Affiliations:** ^1^ Department of Periodontics, Dental College of Georgia, Augusta University, Augusta, GA, United States; ^2^ Department of Cellular Biology and Anatomy, Medical College of Georgia, Augusta, GA, United States

**Keywords:** (MeSH): dendritic cells, exosomes, immune senescence, periodontitis, *Porphyromonas gingivalis*

## Abstract

Periodontitis is a disease of ageing or inflammaging, and is comorbid with other more severe age-related chronic diseases. With advanced age comes an increase in accumulation of senescent cells that release soluble and insoluble pro-inflammatory factors collectively termed the senescence associated secretory phenotype (SASP). In the present report, we examined whether immune cells typical of those at the oral mucosa-microbe interface, are vulnerable to cellular senescence (CS) and the role of dysbiotic oral pathogen *Porphyromonas gingivalis*. Bone marrow-derived dendritic cells (DCs) from young (yDCs) and old (oDCs) mice were co-cultured *in vitro* with CS inducer doxorubicin or *P.gingivalis (Pg)*, plus or minus senolytic agent rapamycin. CS profiling revealed elevated CS mediators SA-β-Gal, p16 ^INK4A^, p53, and p21^Waf1/Clip1^ in oDCs, or yDCs in response to doxorubicin or *P. gingivalis*, reversible with rapamycin. Functional studies indicate impaired maturation function of oDCs, and yDC exposed to *P. gingivalis*; moreover, OVA-driven proliferation of CD4+ T cells from young OTII transgenic mice was impaired by oDCs or yDCs+Pg. The SASP of DCs, consisting of secreted exosomes and inflammasome-related cytokines was further analyzed. Exosomes of DCs cocultured with *P. gingivalis* (PgDCexo) were purified, quantitated and characterized. Though typical in terms of size, shape and phenotype, PgDCexo were 2-fold greater in number than control DCs, with several important distinctions. Namely, PgDCexo were enriched in age-related miRNAs, and miRNAs reported to disrupt immune homeostasis through negative regulation of apoptosis and autophagy functions. We further show that PgDCexo were enriched in *P. gingivalis* fimbrial adhesin protein mfa1 and in inflammasome related cytokines IL-1β, TNFα and IL-6. Functionally PgDCexo were readily endocytosed by recipient yDCs, amplifying functional impairment in maturation and ability to promote Ova-driven proliferation of OTII CD4+ T cells from young mice. In conclusion *P. gingivalis* induces premature (autocrine) senescence in DCs by direct cellular invasion and greatly amplifies senescence, in paracrine, of bystander DCs by secretion of inflammatory exosomes. The implications of this pathological pathway for periodontal disease *in vivo* is under investigation in mouse models.

## Introduction

Periodontitis (PD) in adults is a chronic inflammatory disease of ageing or inflammaging (Ebersole et al., 2018; Qin et al., 2020). This is particularly striking when the National Health and Nutrition Examination Survey (NHANES) data from 2009-2010 are stratified by patient age, with PD prevalence increasing from 24.4% in adults aged 30-34 yrs., to 70.1% in those aged 65 yrs. and over (Eke et al., 2012). Periodontitis has been linked to other age-related diseases with more serious mortality and morbidity profiles such as cancer (Michaud et al., 2017), Alzheimer’s disease (Teixeira et al., 2017), and atherosclerosis (Carrion et al., 2012). *Porphyromonas gingivalis (P.gingivalis)* is considered a “keystone” pathogen in PD due to its outsized influence on the local microflora. *P. gingivalis* induces a dysbiosis by disruption of innate immunity, wherein symbiotic commensals become accessory pathogens (Hajishengallis et al., 2011; Hajishengallis et al., 2012; Dutzan et al., 2018) through unclear mechanisms. The antigen presenting cells dendritic cells (DCs), responsible for bridging innate and adaptive immunity, infiltrate the gingiva in PD (Jotwani et al., 2001; Jotwani and Cutler, 2003; Jotwani et al., 2003; Jotwani et al., 2004), where *P. gingivalis* and other microbes are taken up *in vivo* (Carrion et al., 2012; El-Awady et al., 2019; Rajendran et al., 2019). *P.gingivalis* also invades epithelial, endothelial and smooth muscle cells (Li et al., 2008). Invasion of DCs by *P.gingivalis* occurs through coordinate regulation of its minor (mfa-1) and major (fimA) fimbriae (Zeituni et al., 2009; Zeituni et al., 2010; El-Awady et al., 2015; El-Awady et al., 2015; El-Awady et al., 2019). *P.gingivalis’* viability in DCs depends on an mTORC1-dependent anti-autophagosomal/lysosomal pathway (Arjunan et al., 2016; Arjunan et al., 2018; Meghil et al., 2019), while viability of the host DCs is preserved by an anti-apoptotic signaling pathway involving hyperactivation of Akt1 (Arjunan et al., 2016; Arjunan et al., 2018; Meghil et al., 2019). Hyperactivation of Akt1 and antiapoptotic pathways (Meghil et al., 2019) have recently been linked to premature cellular senescence (CS) (Nogueira et al., 2008).

CS is typified by irreversible cell cycle arrest (in replicative cells), increased apoptosis resistance and activation of the senescence-associated secretory phenotype or SASP (Acosta et al., 2013). CS, first reported by Hayflick and colleagues (Hayflick and Moorhead, 1961) as cell cycle arrest after a finite number of cell divisions, (also known as Hayflick limit), is now recognized as a more complex phenomenon. CS can be caused by a variety of stressors which can lead to different types of senescence. In addition to replicative senescence, oncogene-induced senescence, genotoxic-induced senescence, developmental senescence and tissue repair senescence have been described (Gorgoulis et al., 2019). Fundamentally, CS is a defense mechanism to prevent dissemination of tissue damage and protect against tumorigenesis; however, accumulation of senescent cells can be harmful to the host environment. Although senescent cells are quiescent, they are metabolically active and have the capacity to release a plethora of pro-inflammatory mediators collectively known as SASP. The SASP can promote paracrine senescence in neighboring cells and elicit a chronic inflammatory status known as inflammaging. The SASP consist of a distinct profile of inflammatory cytokines and extracellular vesicles (EV), e.g. exosomes, collectively called the secretome. Exosomes are nano-sized EV of endosomal origin secreted by all cells (Jakhar and Crasta, 2019), and contain proteins, RNAs and other moieties reflective of the homeostatic or pathologic state of the donor cells. Exosomal microribonucleic acids (miRNA) have emerged as novel biological biomarkers of aging (Machida et al., 2015). Saliva exosomes (Murillo et al., 2019) for example, are being studied for molecular clues about cancer initiation/progression (Nonaka and Wong, 2017). From a functional standpoint exosomes can amplify CS signaling in an autocrine fashion, or transmit CS to non-aged cells in paracrine (Acosta et al., 2013). Advanced age-associated alveolar bone loss, reported in mice, is attenuated by senolytic agent rapamycin (An et al., 2017).

Keratinocytes (Moffatt-Jauregui et al., 2013) and DCs (Jotwani et al., 2001; Jotwani and Cutler, 2003; Jotwani et al., 2004), both resident cells at the front line of the oral mucosal-microbe interface, may be particularly vulnerable to CS induction. Indeed, we show here by CS profiling, DCs derived from bone marrow of young mice (yDCs) are readily provoked to CS by exposure to canonical CS stressor doxorubicin and *P. gingivalis* 381. The response of yDCs to these stressors included elevated IL-1β and TNFa mRNA levels, but impaired maturation response, and impaired antigen presentation to OTII CD4 T cells, consistent with immune senescence. Moreover, DCs from old mice (oDCs) are similarly impaired at baseline. *P. gingivalis* has been identified inside DCs *in vitro* and *in vivo* in PD patients (Carrion et al., 2012; El-Awady et al., 2019; Rajendran et al., 2019). Here we show that *P. gingivalis* invasion of yDCs is a predicate of robust senescence; moreover, resultant yDCs had a 2-fold greater secretion of exosomes. These *P. gingivalis-*induced DC exosomes (PgDCexo) while morphologically typical exosomes, are enriched in anti-apoptosis and anti-autophagy miRNAs, inflammasome proteins IL-6 and mature IL-1b, and unexpectedly, the minor mfa1 fimbrial protein of *P. gingivalis;* moreover, PgDCexo *are* taken up by recipient yDCs, promoting immune senescence in recipient young DCs. Overall the results support *P. gingivalis* as a potent cell stressor of CS, both directly through infection of cells and indirectly through induction of the exosomal SASP; thereby promoting immune senescence in paracrine to bystander cells.

## Materials and Methods

### Ethics Statement

The Institutional Animal Care and Use Committee (IACUC) of Augusta University (protocol # 2013-0586) approved all experimental procedures on C57BL/6 mice.

### Generation and Culture of Bone Marrow Derived Dendritic Cells From Young (yDCs) and Old (oDCs) Mice

Generation of BMDCs was performed as previously described (Elashiry et al., 2020; Elsayed et al., 2020). Briefly, bone marrow was isolated from femurs and tibiae of young (2 months) and old (22-24 months) C57B6 mice. Red blood cells were lysed using ACK cell lysis buffer (Invitrogen, Thermofisher scientific, and Columbia, SC, USA). Cells were cultured in complete media (RPMI 1640 containing 10% FBS and 100 IU/mL penicillin/streptomycin) in the presence of murine GM-CSF (20 ng/ml) and IL-4(20 ng/ml) (Peprotech, Rocky Hill, NJ, USA). Culture media was changed every 2 days with the addition of fresh growth factors. Cells were re-incubated on day 6 in EXO depleted complete media to generate iDCs or in the presence of 1µg/ml LPS (Sigma, St. Louis, M.O., USA) to generate mature stimDCs for 48 hours. On day 8, cells were washed and re-incubated in EXO free media for 24 h and on day 9, cells were isolated and, culture supernatants were collected for EXO purification. In some of the experiments, bone marrow derived dendritic cells (DCs) were treated at day 6 of culture with Doxorubicin (Sigma Aldrich, St Louise, MO, USA) at a dose of 50nM, Ecoli LPS (Sigma Aldrich, St Louise, MO, USA) at 100ng/ml. *P.gingivalis* LPS (Invivogen Inc., San Diego, CA, USA) at 100ng/ml, Rapamycin 1µM(R8781)(Sigma Aldrich, St Louise, MO, USA) or PgDCexo, StimDCexo, iDCexo 108 particles/ml (Elashiry et al., 2020).

### *P.gingivalis* Bacterial Culture and DCs Infection

*P.gingivalis* 381 (Pg) was maintained anaerobically in (10%, H2, 10% CO2, and 80% N2) in a Coy lab vinyl anaerobic chamber (Coy Laboratory Products, Inc., Grass Lake, MI) at 37◦C in Wilkins-Chalgren anaerobe broth. Bacterial cells were maintained until mid-log phase. Bacterial CFU were calculated based on a spectrophotometer OD 660 reading of 0.11, previously determined to equal 5x10^7 CFU (El-Awady et al., 2015; Meghil et al., 2019). At day 6 of DC culture, cells were pulsed with *P.gingivalis* at a multiplicity of infection (MOI) of 10 for 48 hours. On day 8, cells were washed and incubated for 24 hrs and on day 9, cells were isolated and, culture supernatants were collected for EXO purification.

### Exosome Isolation and Purification

Exosome isolation was performed as previously described (Kim et al., 2005; Elashiry et al., 2020). Briefly, supernatants from DC cultures were subjected to differential centrifugation (successive centrifugations at 500 g for (5 min), 2000g for (20 min), and 10,000 g for (30 min) to eliminate cells and debris. This was followed by ultrafiltration 2x with 0.2 µm and 2x with 100 kDA filters (to remove microvesicles and free proteins) and ultracentrifugation for 90 min at 120,000 g. Then EXO pellets were washed with PBS and ultra-centrifuged 2x at 120,000 g for 90 min, and finally re-suspended in 100 µl of PBS, stored in -80 for further analysis and experiments.

### Nanotracking Analysis of DC-Derived Exosomes

For quantification of size and count of nanoparticles in suspension, Nano tracking analysis (NTA) was performed using Zeta view PMX 110 (Particle Metrix, Meerbusch, Germany) as previously described (Helwa et al., 2017; Elashiry et al., 2020). Briefly, 10 µl of the sample was diluted in 1xPBS and loaded into the sample chamber, then size and concentration of the sample were automatically calculated by the software (ZetaView 8.02.28).

### Electron Microscopy

As described previously (Helwa et al., 2017; Elashiry et al., 2020) exosome sample was fixed in 4% paraformaldehyde in 0.1M cacodylate buffer ph 7.4 overnight. Twenty microliter (20ul) of suspended exosome preparation was applied to a carbon-Formvar coated 200 mesh nickel grid and allowed to stand 30 minutes. The excess sample was wicked off onto Whatman filter paper. Grids were floated exosome side down onto a 20 µl drop of 1M Ammonium Chloride for 30 minutes to quench aldehyde groups from the fixation step. Grids were floated on drops of blocking buffer (0.4% BSA in PBS) for 2 hours. Then rinsed 3 X 5 minutes each with PBS. Grids were set up as follows and allowed to incubate in blocking buffer or the primary antibody (anti CD63, anti CD81, anti Mfa1) for 1 hour. Grids were floated on drops of 1.4 nm secondary antibody nanogold (Nanoprobes, Inc.) diluted 1:1000 in blocking buffer for 1 hour. Grids were rinsed 3 X 5 minutes each with PBS. Grids were rinsed 3 X 5 minutes each with DI H₂O. For double labelling, grids were enhanced 12 minutes (for Mfa1 antibody) and 3 minutes (for CD81 antibody) in Gold Enhance EM (gold enhancement reagent, Nanoprobes, Inc.) and rinsed in ice cold DI H₂O to stop enhancement. The different times for enhancement allows for the gold particles labeling the first primary antibody to be enhanced twice producing larger size gold particles between 20 and 30 nm in size. The gold particles labeling the second primary antibody is enhanced only once and for a much shorter time to produce gold particles which are smaller in size, between 10 and 12 nm in size. Then grids were negatively stained in 2% aqueous Uranyl Acetate and wicked dry. Grids were allowed to air dry before being examined in a JEM 1230 transmission electron microscope (JEOL USA inc., Peabody MA) at 110 kV and imaged with an UltraScan 4000 CCD camera & First Light Digital Camera Controller (Gatan Inc., Pleasanton, CA.)

### miRNA Microarray of DC-Derived Exosomes

Total EXO RNA Kit (4478545), (Thermofisher Scientific, Waltham MA, USA) was used to isolate miRNAs from exosomes according to manufacturer’s instructions. The concentration of miRNA was measured using a NanoDrop spectrophotometer (Thermo Scientific) and analysis of the quality of miRNA was performed using an Agilent 2100 Bioanalyzer. Analysis of miRNAs was performed using an Affymetrix GeneChip^®^ miRNA 4.0 Array at the Integrated Genomics Core, Augusta University, GA. A P-value cut-off of 0.05 and the miRNAs with a fold change above 1.5 were considered differentially expressed. Bioinformatic analysis was performed using ClastVist software and TargetScan software was used to detect computationally predicted miRNA-mRNA target relationships.

### Senescence Associated β Galactosidase (SA-β-Gal) Histochemical Staining Kit

DCs suspension were subjected to cytospins on a microscopic slide before fixation and staining. SA-β-gal histochemical staining kit (CS0030) **(**Sigma Aldrich, St Louise, MO, USA) was used according to the manufacturer’s instructions. Briefly, cells were fixed with fixation buffer supplied with the kit (20% formaldehyde, 2% glutaraldehyde, 70.4 mM Na2HPO4, 14.7 mM KH2PO4, 1.37 M NaCl, and 26.8 mM KCl), for 6-8 minutes then washed 3x with PBS. This was followed by adding staining solution containing X-Gal and incubated for 12 hours at 37°C with no CO2. Cells were then washed with PBS and visualized under EVOS cell imaging system (Evos FL Auto Imaging System, Thermofisher) and at least 5 random pictures were taken per sample with 60x objective lens.

### SA-β-Gal Flow Cytometry Staining Kit

Cell Event Senescence Green flow cytometry assay kit (Thermofisher Scientific, Waltham MA, USA) was used according to the manufacturer’s instruction. Briefly DCs were first stained for cell surface marker CD11c using regular staining protocol discussed in detail in the following sections. After the last wash, cells were fixed with 4% paraformaldehyde (PFA) for 10 minutes at room temperature followed by washing to remove the fixative solution. Cells were then incubated with Cell Event Senescence Green probe (Thermofisher Scientific, Waltham MA, USA) at 1:500 dilution for 2 hours at 37°C with no CO2. Cells were then washed with PBS, 2% FBS and data acquired by MACSQuant analyzer machine and MACSQuantify software (Miltenyi Biotech Auburn, CA, USA).

### Exosome Uptake *In Vitro*

Analysis of exosome uptake consisted of labelling exosomes with Dil (D282, Thermofisher Scientific, Waltham MA, USA), then coculturing them with recipient DCs for 24h. Cells were subjected to cytospins, fixed and stained on glass slides with Alex flour 647 phalloidin (A22287) and DAPI (D1306) (Invitrogen, Thermofisher scientific West Columbia, SC, USA). Slides were then visualized by Zeiss upright confocal microscope (Carl Zeiss AG, Oberkochen, Germany) and images were taken with 40x objective lens.

### *P*.*gingivalis* Uptake by DCs In Vitro

*P.gingivalis* was labeled with 10 μM carboxyfluoresceine succinimidyl ester (CFSE ebioscience, Invitrogen, Thermofisher Scientific West Columbia, SC, USA) for 1 hour at 37 degree with shaking, then residual stain removed by successive washings and bacterium suspended at 10 MOI for uptake experiments, with/without Cytochalasin D 1uM (Sigma Aldrich, St Louise, MO, USA) for 48 hours, after which cells were harvested and analyzed by flow cytometry and SA-B-gal staining. Cells were harvested, thoroughly washed and then fixed with 4% paraformaldehyde, permeabilized with 0.1% Triton X-100 in PBS and stained with actin ActinRed 555 ReadyProbes Reagent and nuclear counterstaining using DAPI mounting medium; ProLong Gold Antifade Mountant (Invitrogen, Thermofisher scientific, Waltham MA, USA), then images were taken using Zeiss 780 upright confocal microscope (Carl Zeiss AG, Oberkochen, Germany).

### Flow Cytometry and Antibodies

FACS Staining Buffer (Thermofisher Scientific, Waltham MA, USA) was used to stain cells on ice. FC receptors (FcR) were blocked using mouse FcR blocking reagent (Miltenyi Biotec, Auburn, CA, USA) for 15 minutes protected from light followed by incubation with conjugated antibodies on ice for 30 minutes. Cells were then washed and re-suspended in FACS buffer and data was acquired using MACSQuant analyzer machine and MACSQuantify software (Miltenyi Biotech Auburn, CA, USA). Antibodies used: CD11c APC; clone N418 (Affymetrix, eBioscience, Thermofisher Scientific, Waltham MA, USA)., CD86 (B7-2) PE; clone GL1(Affymetrix, eBioscience, Thermofisher Scientific, Waltham MA, USA)., CD80 FITC; clone 16-10A1(Invitrogen, Thermofisher Scientific, Waltham MA, USA), CD4 Vioblue; clone GK1.5 (Invitrogen, Thermofisher Scientific, Waltham MA, USA), MHCII Viogreen; clone M5/114.15.2, (Miltenyi Biotech Auburn, CA, USA).

### Real Time PCR

Total RNA was isolated using QIAGEN RNeasy mini kit (Qiagen, Inc., Valencia, CA, and USA). RNA purity and concentration were measured using Nanodrop (NanoDrop 1000 UV-VIS Spectrophotometer Software Ver.3.8.1, Thermofisher Scientific). Ratio of 260/280 of 2.0 was considered adequate for analysis. Reverse transcription to cDNA was performed using the High-Capacity cDNA Reverse Transcription Kit (Applied Biosystem, Thermofisher Scientific, Waltham MA, USA) in total reaction of 20 μL. Quantitative real-time PCR was performed using TaqMan fast advanced master mix (Applied Biosystem, Thermofisher Scientific, Waltham MA, USA) and Taq-Man Gene Expression assay (Applied Biosystem, Foster City, CA, USA) specific for: IL6 (Mm00446190_m1), TNF (Mm00443258_m1) and IL1B (Mm00434228_m1), internal control Glyceraldehyde-3-phosphate dehydrogenase (GAPDH Mm99999915_m1). RT-PCR was run in StepOnePlus Real-Time PCR System. Calculation of relative mRNA expression was performed using delta-delta CT and presented as relative fold-change.

### Western Blotting Analysis, Antibodies

Cells or exosomes were lysed using RIPA buffer with the addition of protease/phosphatase inhibitor cocktail and incubated for 20 minutes on ice, then protein lysates stored at -80° till further use. After denaturation of proteins lysates, equal protein concentrations and volumes were loaded and separated by 10-15% Mini-PROTEAN TGX Precast Protein Gel (Bio-Rad Laboratories, Hercules, CA), and transferred onto PVDF membranes (Sigma-Aldrich). Membranes were blocked with 5% nonfat dry milk in TBST, then incubated with primary antibodies at 4° overnight. After washing with TBST, membranes were incubated with HRP-conjugated secondary antibodies for 1 h at room temperature. Membranes were then washed and developed by ECL kit and imaged with ChemiDoc MP Imaging Gel (Bio-Rad Laboratories, Hercules, CA). Antibodies used: anti-Beta-actin (8H10D10) as loading control (Cell Signaling Technology, Danvers, MA, USA). Anti-mouse anti p21^Waf1/Clip1^ (#64016) (Cell Signaling Technology, Danvers, MA, USA). Anti-mouse anti p16 ^INK4A^ PA1030670 (Thermofisher Scientific, Waltham MA, USA) and anti p53(#2524) (Cell Signaling Technology, Danvers, MA, USA), anti-TSG101 (MA1-23296) (Thermofisher Scientific, Waltham MA, USA), anti-Alix (MA1-83977) (Thermofisher Scientific, Waltham MA, USA), anti CD81(#10037) (Cell Signaling Technology, Danvers, MA, USA) Anti IL6(12912) (Cell Signaling Technology, Danvers, MA, USA), anti cleaved-IL-1ββ (#52718) (Cell Signaling Technology, Danvers, MA, USA), anti TNFa (#11948) (Cell Signaling Technology, Danvers, MA, USA), anti-Mfa1 (mAb 89.15 against the native minor fimbriae (Zeituni et al., 2010), generated at the Cell Culture/Hybridoma Facility at Stony Brook University, as reported (Carrion et al., 2012)), secondary antibodies: anti-mouse IgG HRP-linked (#7076) (Cell Signaling Technology, Danvers, MA, USA), anti-rabbit IgG HRP-linked (# 7074) (Cell Signaling Technology, Danvers, MA, USA).

### T Cell Isolation and Antigen Presentation

OVA antigen-specific T cells were isolated from OT-II transgenic mice (B6.Cg-Tg(TcraTcrb)425Cbn/J; Jackson Laboratory) using negative selection with Mouse T-cell Enrichment Kit (MagniSort; Thermofisher Scientific, West Columbia, SC, USA). T cell purity was assessed by flow cytometry analysis, using CD4 antibody. OT-II T cells were stained with 0.5 μM CFSE (ebioscience, Invitrogen, Thermofisher Scientific West Columbia, SC, USA) for 15 minutes at 37°C, after which cells were washed 2 times. DCs pretreated with PgDCexo, iDCexo, or StimDCexo, or infected with *P.gingivalis* (10 MOI) ± Rapamycin were harvested, washed thoroughly and pulsed with 3 μg/mL OVA323-339 peptide (Sigma Aldrich, St Louise, MO, USA) for 24 hours. Cells were then harvested, thoroughly washed and co-cultured with T cells at 1:10 DC: T cell ratio in 96-well round- bottom plates with complete RPMI 1640 (10% fetal bovine serum FBS,1% Pen Strep, 1x non-essential amino acids, 0.1% b-mercaptoetanol). After a 72-hours incubation at 37°C with 5% CO2, the proliferation of CFSE-labeled CD4+ T cells was then assessed by flow cytometry.

### Magnetic Separation of CD81, CD9, CD63 Positive EXO

PgDCEXO were incubated with microbeads that recognize CD81, CD9 and CD63 positive exosomes (pan EXO) (Miltenyi Biotech Auburn, CA, USA). Magnetic separation of labeled EXO was performed by loading it onto a µ column placed in a magnetic field of µMACS separator (Miltenyi Biotech Auburn, CA, USA). The magnetically labeled EXO are retained within the column and the unlabeled vesicles run through the column. The positively selected retained EXO are then eluted from the column. The number of particles in the run through (passage1) of unlabeled extracellular vesicles (EV) fraction was then counted using NTA. Magnetic separation of the run through was repeated (passage 2) to completely deplete pan EXO from the run through and then particles counted in the final run through of unlabeled EV and a percentage to the total number of EV was calculated. The eluate (CD81, CD9 and CD63 positive exosomes) and the run through were then analyzed using WB.

### Statistical Analysis

Data was analyzed using GraphPad Prism 6 (GraphPad Software, La Jolla, CA). Data analysis was performed by two-way or one ANOVA with significance defined as P < 0.05, and confidence level of 95% confidence interval followed by Tukey’s multiple-comparisons test. Values are expressed as mean ± standard deviation (SD) and experiments were repeated 3 times.

## Results

### Murine DCs From Old Mice, or Young Mice Exposed to *P. gingivalis*, Are Senescent

**Figure 1:**
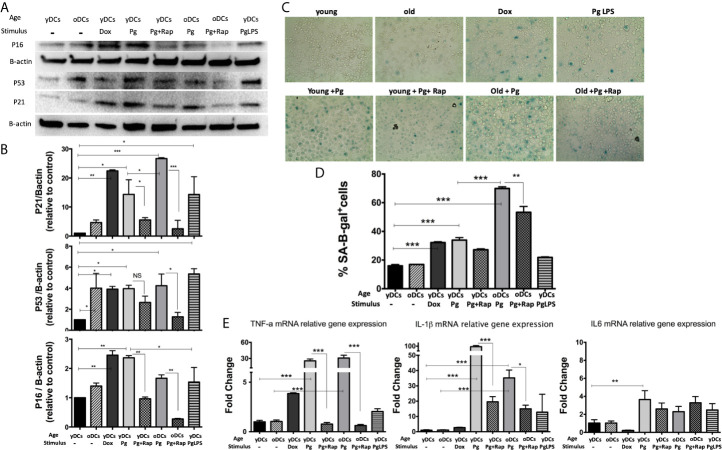
*P.gingivalis* induces immune senescence in murine bone marrow derived DCs (DCs): At day 6, DCs from young (yDCs) and old (oDCs) mice were co-cultured with *P.gingivalis* (10 MOI) ± Rapamycin (1µM), yDCs were treated with Dox (50nM) or *P.gingivalis* LPS (100ng/ml) and CS profiling was performed at day 8. **(A)** Representative western blot images for p16 ^INK4A^, p53, and p21^Waf1/Clip1^ protein expression in BMDCS treated with *P.gingivalis* ± Rapamycin, Dox or LPS. **(B)** Densitometric analysis of p16 ^INK4A^, p53, and p21^Waf1/Clip1^ protein expression in BMDCs. β-actin was used as a loading control. Band densities were normalized to β-actin, and data presented as fold change relative to the control (yDCs with no stimulus). **(C)** Representative images of SA-β-gal staining of BMDCs at PH 6, blue stain indicates senescence **(D)** Quantification of SA-β-gal positive cells using FACS analysis. **(E)** qPCR analysis showing relative mRNA expression of TNFa, IL-1β- and IL6 in BMDCs treated with *P.gingivalis* ± Rapamycin, Dox or LPS. Relative gene expression was calculated using delta-delta CT method and presented as fold change relative to the control (yDCs.). Analysis was done using one-way ANOVA and Tukey multiple comparison *post hoc* test (Data are expressed as means ± SD, *p<0.05, **p<0.01, ***p<0.001). NS, not significant.

DCs are resident sentinel immune cells in oral mucosa and the bloodstream, previously shown to contain *P. gingivalis* microbial signatures in periodontitis (PD) patients (Carrion et al., 2012; El-Awady et al., 2019; Rajendran et al., 2019). Here, bone marrow derived DCs (DCs) from young (yDCs) or old (oDCs) mice were cocultured with *P. gingivalis* or doxorubicin and subjected to CS-profiling. Both *P. gingivalis* and doxorubicin stimulated an increase in p16 ^INK4A^, p53 and p21^Waf1/Clip1^ in yDCs (**Figures 1A, B**). oDCs had a trend towards constitutively higher levels of senescence markers than yDCs shown by immunoblot analysis of induced p16 ^INK4A^, p53 and p21^Waf1/Clip1,^however, it only reached significance level in P53 upregulation. Chromogenic staining (**Figure 1C**) and FACS analysis (**Figure 1D**) confirmed SA-β-Gal induction in yDCs and oDCs in response to *P. gingivalis* or doxorubicin. Senolytic agent rapamycin (Sargiacomo et al., 2020) reversed *P. gingivalis*-induced p16 ^INK4A^, p53 and p21^Waf1/Clip1^ and *P.gingivalis*-induced SA-β-Gal in in yDCs and oDCs. *P.gingivalis* LPS induced increased expression of p53 and p21^Waf1/Clip1^, but not p16 ^INK4A^ and SA-β-Gal in yDCs compared to control yDCs with no infection. IL-1β, TNFa, and IL6 are known to be major constituents of the SASP. Here we show that *P. gingivalis* was the most potent inducer of IL-1b, and TNFa transcripts in yDCs and oDCs while increasing IL-6 relative mRNA expression only in yDCs. Rapamycin ablated TNFa, and reduced IL-1b but did not influence IL-6 induction by *P. gingivalis* in yDCs (**Figure 1E**).

### Impairment of Maturation Function in oDCs or yDCs Exposed to *P. gingivalis*

**Figure 2:**
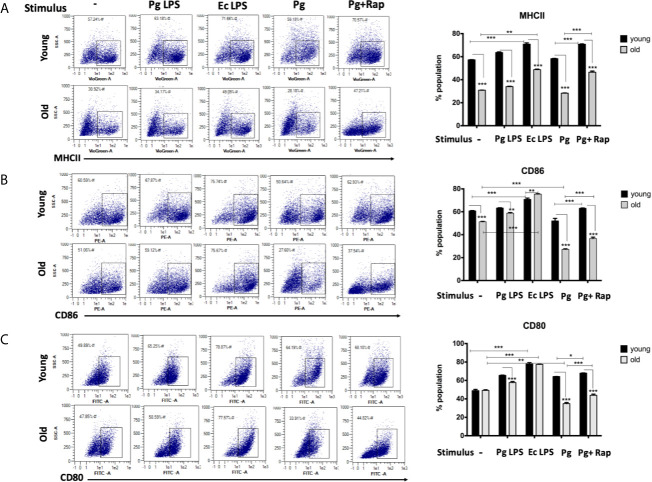
*P.gingivalis* impairs maturation of bone marrow derived dendritic cells(DCs) from young and old mice. At day 6, DCs from young (yDCs) and old (oDCs) mice were co-cultured with *P.gingivalis* (10 MOI) ± Rapamycin (1µM), Pg LPS (100ng/ml) or Ecoli LPS (100ng/ml). Cells were harvested at day 8 and analyzed by flow cytometry for the expression of maturation markers, MHCII and co stimulatory molecules CD80 and CD86. **(A)** Flow cytometry scatter gram and its corresponding bar graph showing % of MHCII positive cells in young (upper panel) and old (lower panel), DCs gated on CD11c^+^ cells (gate not shown). **(B)** Flow cytometry scatter gram and its corresponding bar graph showing % of CD86 positive cells in young (upper panel) and old (lower panel), DCs gated on CD11c^+^ cells (gate not shown).**(C)** Flow cytometry scatter gram and its corresponding bar graph showing % of CD80 positive cells in young (upper panel) and old (lower panel), DCs gated on CD11c^+^ cells (gate not shown). Analysis was done using Two-way ANOVA and Dunn’s correction test. (Data are expressed as means ± SD, *p<0.05, **p<0.01, ***p<0.001). NS, not significant.

In view of CS profiling results, we examined the phenotype and maturation functions of oDCs and yDCs before and after co-culture with *P. gingivalis* or LPS by FACS analysis (**Figure 2A**). Constitutive expression of CD86 and MHCII was lower in oDCs than yDCs. E. coli LPS elicited a strong DC maturation response, relative to PgLPS in yDCs, but oDCs were less responsive to either stimulant*. P.gingivalis* did not induce maturation of yDCs or oDCs, relative to control and levels were significantly lower than that induced by E.coli LPS, and CD86 and MHCII significantly lower than that induced by Pg LPS. Interestingly, *P.gingivalis* significantly reduced constitutive CD86, and CD80 levels in oDCs (51.06%, 47.8%) compared to control oDCs with no infection (27.6% and 33.9%) respectively (**Figures 2B, C**). Rapamycin reversed *P.gingivalis*-induced deficit in maturation as shown by a significant increase in expression of MHCII, CD86 and CD80 in yDCs and oDCs.

### *P. gingivalis* Invasion for Robust Immune Senescence in yDCs

**Figure 3:**
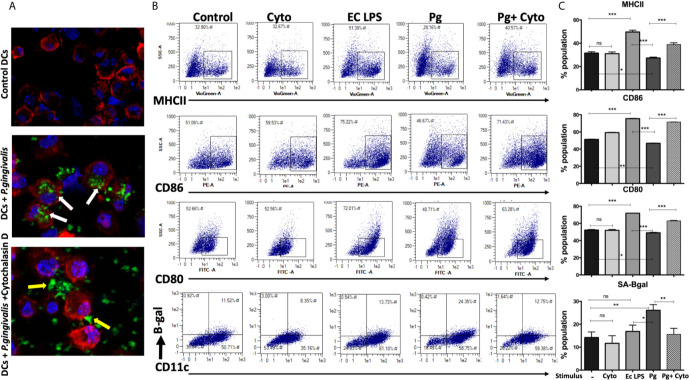
*P.gingivalis*-induced senescence and impaired maturation in yDCs is abrogated by inhibition of its uptake by DCs. At day 6, yDCs were co-cultured with *P.gingivalis* (10MOI) ± Cytochalasin D (1ug/ml), or cytochalasin D alone, or E-coli LPS. Cells were harvested at day 8 and analyzed by flow cytometry for the expression of maturation markers, MHCII and co stimulatory molecules CD80 and CD86 and SA-β-gal. **(A)** Representative confocal microscopy images showing uptake (white arrows) of CFSE labeled *P.gingivalis* (green) into DCs with labeled actin (red) and counterstained with DAPI for nuclear staining (blue). Yellow arrows indicating the inhibition of uptake and accumulation of CFSE labeled *P.gingivalis* on the outer surface of the cell. **(B)** Flow cytometry scattergrams showing effect of inhibition of *P.gingivalis* uptake using cytochalasin D on expression of maturation markers, measured by % population of MHCII(first panel), CD86 (second panel) and CD80 (third panel) and activation of senescence marker SA-β-gal (fourth panel). All gated on CD11c^+^ cells (gate not shown). **(C)** Summary bar graphs of the flow cytometry data). Analysis was done using one-way ANOVA and Tukey multiple comparison *post hoc* test (Data are expressed as means ± SD, *p<0.05, **p<0.01, ***p<0.001). NS, not significant.

We hypothesized that active invasion of DCs by *P. gingivalis* (Zeituni et al., 2009; Zeituni et al., 2010; Zeituni et al., 2010; Acosta et al., 2013) was a critical factor in robust immune senescence. Accordingly, yDCs were cocultured with CFSE-labeled *P. gingivalis* in the presence or absence of cytochalasin D. Invasion was imaged by confocal microscopy, with SA-β-Gal, MHCII, CD80 and CD86 expression assessed by flow cytometry. Images show internalized *P. gingivalis* (**Figure 3A**), associated with increased SA-β-Gal expression on yDCs, and unresponsiveness in MHCII, CD80 and CD86 expression (**Figures 3B, C**). Pre-treatment of yDCs with cytochalasin D reduced *P. gingivalis* invasion, reversed SA-β-Gal response and restored the MHCII, CD86 and CD80 responsiveness (**Figures 3B, C**). E coli LPS control established that yDCs were fully capable of maturation.

### PgDCexo Are Typical Exosomes With Distinctive Characteristics

**Figure 4:**
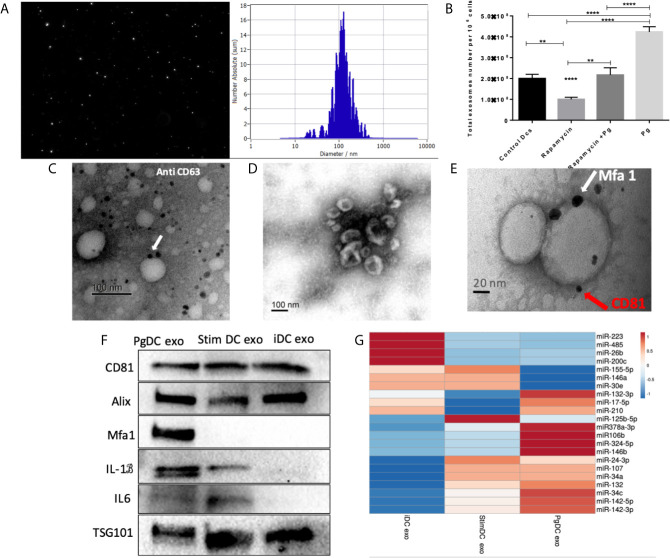
Characterization of exosomes released from DCs infected with *P.gingivalis* (PgDCexo): **(A)** Nano tracking analysis (NTA) to determine Exo number and size distribution in nanometer (nm). **(B)** Total number of Exo released per 10^6^ cells as determined by NTA from DCs treated or non-treated with Pg and/or Rapamycin **(C)** Immuno-gold TEM showing EXO marker, tetraspanin CD63. **(D)** SEM showing characteristic Exo morphology. **(E)** Co-localization of Mfa1 bacterial protein (large- sized gold Nano particle indicated with white arrow) with the eukaryotic cellular exosomal marker, CD81 (small-sized gold Nano particle indicated with red arrow) using TEM immune-gold double labeling **(F)** Western blot showing Exo-related markers TSG101, Alix, and tetraspanin CD81 and proinflammatory cytokines IL6, TNFa and IL-1b and Mfa1 in exosomes released from control immature DCs (iDCs), Ecoli LPS-stimulated mature DCs (StimDCexo) and Pg infected DCs (PgDCexo). **(G)** miRNA analysis of Pg or Ecoli LPS-induced DCs Exo (PgDC exo and StimDCexo respectively) compared to immature control DCs exo; (iDCexo). (iDCs), control immature DCs exo, (StimDCexo), Ecoli LPS-stimulated mature DCs exo; (PgDCexo), Pg-infected DCs. **p<0.01, ****p<0.001, 1 Way ANOVA.

CS is a complex cellular inflammatory process in non-immune (Naylor et al., 2013), and immune cells (Mondal et al., 2013; Salminen, 2020); most notable feature being induction of the SASP. Exosomes have emerged as an important characteristic of the SASP (Acosta et al., 2013). Here, exosomes released by *P.gingivalis* infected yDCs (PgDCexo), relative to uninfected control DCs were isolated, characterized and quantitated. Correct size distribution (30-150 nm) was confirmed by nanotracking analysis (**Figure 4A**). Enumeration of secreted exosomes indicate 2-fold greater number of exosomes from *P. gingivalis*-infected yDCs (=4.2 x 10^8^ per 10^6^ DCs) than control DCs (ctl DC) (=2 x 10^8^). This increase was ablated by senolytic agent rapamycin (=2.2 x 10^8^) (**Figure 4B**). Correct phenotype of typical DC exosomes was confirmed by immunogold TEM for CD63 (**Figure 4C**) and SEM showing characteristic cup-shaped morphology (**Figure 4D**). Western blotting analysis (**Figure 4F**) establishes typical exosomal markers CD81, Alix and TSG101 on PgDCexo and control iDC exo and stim DCexo (Elashiry et al., 2020). PgDCexo also express the minor Mfa1 fimbrial protein, an adhesin used by *P.gingivalis* to invade DCs *in vitro* (Zeituni et al., 2010) and *in vivo* (Carrion et al., 2012; El-Awady et al., 2015; El-Awady et al., 2019). Co-localization of Mfa1 fimbrial protein with the tetraspanin CD81, a eukaryotic exosomal marker was confirmed using TEM immuno-gold double labeling (**Figure 4E**). Passage of PgDC exo over a Pan exo magnetic bead column 1x and 2x removed 99.9% and 99.99% of particles, respectively. Column eluate was enriched in Mfa1, CD81, Alix, TSG101 and CD9, consistent with bona fide exosomes, while run-through contained trace levels of Mfa1, Alix, TSG101 and CD9 (**Supplementary Figure 1**). PgDCexo also contain IL-6 and mature IL-1β, consistent with inflammasome activation and the SASP (Acosta et al., 2013). StimDCexo, from donor yDCs treated with LPS, also expressed IL-6 and IL-1β, as previously reported (Elashiry et al., 2020), albeit at lower levels than PgDCexo (**Figure 4F**). Bioinformatics analysis of miRNA content of exosomes (**Figure 4G**) show miRNAs upregulated (red) and down regulated (blue) in PgDCexo, relative to control exosomes from imDCs or LPS-matured DCs (stimDC). Distinct pattern of blue/red miRNAs in PgDCexo versus controls is particularly striking. Of particular note are miRNAs involved in disruption of immune homeostasis, through anti-apoptosis and anti-autophagy functions, including miR106b, miR-17-5p, miR378a-3p, miR-324-5p, miR-132-3p. MiR17-5p was 1.71-fold upregulated in PgDCexo vs. imDCexo, and 1.2-fold downregulated in mDCexo vs imDCexo (**Figure 4G**). MiRNA 132-3p was 4-fold upregulated (p<0.05) in Pg-induced exo, consistent with that previously observed in aged BMDCs (Park et al., 2013).

### DC Exosomal SASP Induced by *P. gingivalis* Amplifies Paracrine Immune Senescence in Recipient yDC

**Figure 5:**
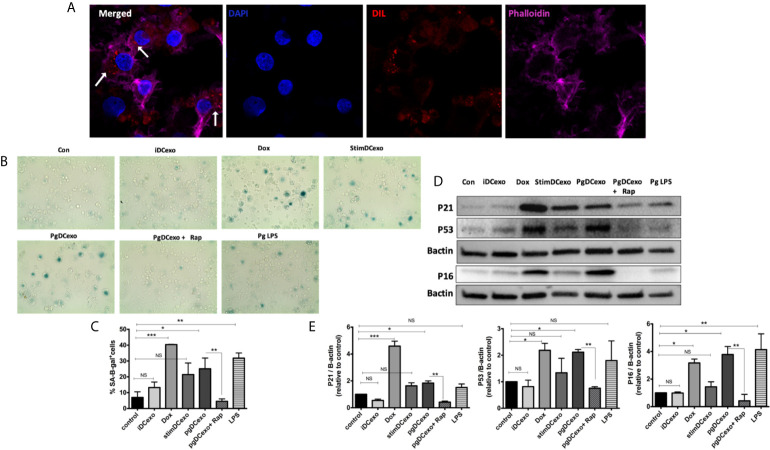
PgDCexo induces paracrine immune senescence in yDCs: **(A)** Confocal microscopy images showing uptake of Dil-labelled EXO (red) by yDCs with phalloidin actin staining (magenta) and counter stained with blue nuclear stain DAPI (white arrows indicating EXO internalization by yDCs). **(B)** Representative images of SA-β-gal staining of yDCs treated with PgDCexo ± Rapamycin, Stim DCexo, iDCexo, Dox, Pg LPS or received no treatment (Con) at PH 6, blue stain indicates senescence. **(C)** Quantification of % population of SA-β-gal^+^ DCs using FACS analysis. **(D)** Representative immunoblot image of p16 ^INK4A^, p53, and p21^Waf1/Clip1^ expression in yDCs with different treatments. **(E)** Densitometric analysis of p16 ^INK4A^, p53, and p21^Waf1/Clip1^ protein expression in yDCs with different treatments. β-actin was used as a loading control. Band densities were normalized to β-actin, and data presented as fold change relative to the control. Analysis was done using one-way ANOVA and Tukey multiple comparison *post hoc* test (Data are expressed as means ± SD, *p<0.05, **p<0.001, ***p<0.0001). (iDCs), control immature DCs exo; (StimDCexo), Ecoli LPS-stimulated mature DCs exo; (PgDCexo), Pg-infected DCs. NS, not significant.

We hypothesized PgDCexo would be potent inducers of immune senescence in non-senescent recipient yDCs. Co-culture of Dil-labeled PgDCexo with recipient yDCs confirmed their uptake as shown by confocal microscopy (**Figure 5A**). CS profiling of recipient yDCs indicate activation of SA-β-Gal, by chromogenic staining (**Figure 5B**) and confirmed by flow cytometry using fluorescent SA-β-Gal staining (**Figure 5C**). Moreover, this increase was significant compared to control yDCs and yDCs treated with exosomes from immature DCs (iDCexo). A significant increase in SA-β-Gal^+^ DCs was also induced directly by doxorubicin and Pg LPS, while stimDCexo induced a non-significant trend for increased SA-β-Gal^+^ DCs (**Figure 5C**). Western blot analysis of PgDCexo recipient DCs (**Figures 5D, E**) showed a significant upregulated expression of p16 ^INK4A^, p53 and p21^Waf1/Clip1^ relative to non-treated DCs and similar to Dox-treated yDCs. No effect on p16 ^INK4A^, p53 and p21^Waf1/Clip1^ expression was observed with iDCexo treatment, while stimDCexo induced upregulation which was not statistically significant. Pg LPS showed an upregulation in p16 ^INK4A^ with no significant effect on p53 and p21^Waf1/Clip1^ expression. Rapamycin reversed PgDCexo-induced senescence (**Figures 5C, E**).

### Maturation and Antigen Presenting Functions of Recipient DCs Shut Down by PgDCexo

**Figure 6:**
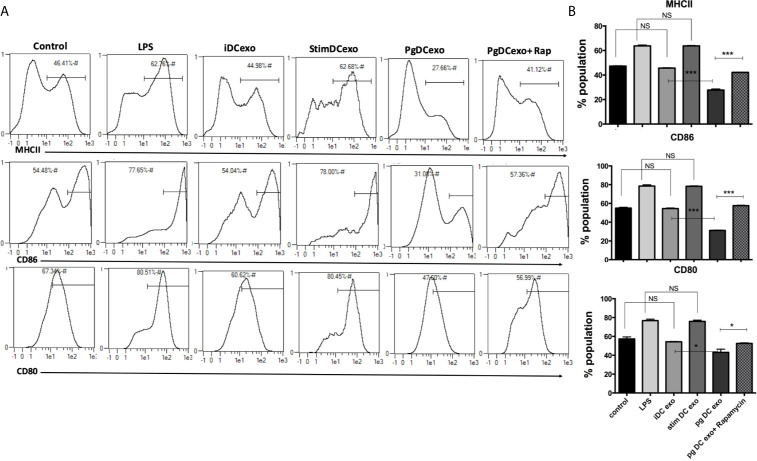
PgDCexo impairs maturation of recipient yDCs: At day 6, yDCs were treated with PgDCexo ± Rapamycin, StimDCexo, iDCexo, Dox, E-coli LPS or received no treatment (Con) and incubated for 48 hrs. Cells were harvested at day 8 and analyzed by flow cytometry for the expression of maturation markers MHCII and co stimulatory molecules CD80 and CD86. **(A)** Representative flow cytometry histograms showing % of MHCII^+^ population in yDCs (upper panel) %CD86^+^ (middle panel) and %CD80+ (lower panel) gated on CD11c^+^ cells (gate not shown). **(B)** Summary bar graphs for flow cytometry data shown in **(A)**. Analysis was done using one-way ANOVA and Tukey multiple comparison *post hoc* test (Data are expressed as means ± SD, *p<0.05, ***p<0.001). (iDCs), control immature DCs exo; (StimDCexo), Ecoli LPS-stimulated mature DCs exo; (PgDCexo) Pg-infected DCs. NS, not significant.

**Figure 7:**
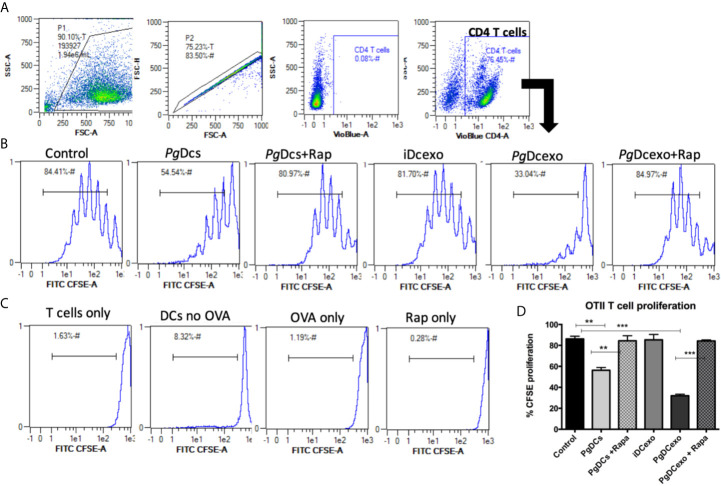
PgDCexo impairs antigen presentation of recipient yDCs: **(A)** Flow cytometry scatter grams showing Gating strategy. **(B)** Representative flow cytometry histograms showing % proliferation of CFSE labeled splenic ovalbumin specific CD4 T cells from OTII mice co-cultured with pre-treated yDCs. **(C)** Flow cytometry histograms showing % of CFSE proliferation in negative controls for proliferation which include T cells only, yDC/T cells only, Ova/T cells, and Rap/T cells. **(D)** Summary bar graphs for flow cytometry data shown in **(B)**. Analysis was done using one-way ANOVA and Tukey multiple comparison *post hoc* test (Data are expressed as means ± SD, **p<0.01, ***p<0.001). (iDCs), control immature DCs exo; (StimDCexo), Ecoli LPS-stimulated mature DCs exo; (PgDCexo), Pg-infected DCs.

The maturation and antigen presentation functions of yDCs cocultured with PgDCexo were examined. FACS analysis revealed ablation of maturation function of PgDCexo recipient yDCs as shown by a significant decrease in maturation markers MHCII, CD86 and CD80 (**Figures 6A, B**), which was restored by rapamycin treatment. Positive control LPS and stimDCexo, but not iDCexo, promoted yDC maturation,. Ability of yDCs treated with PgDCexo to engage in antigen presentation was assessed by coculture of DCs with Ova peptide and CFSE labeled splenic CD4+ T cells from OTII mice. T cell proliferation was assessed by percentage loss of CFSE with each T cell generation, with gating shown in **Figure 7A**). Negative controls for proliferation include T cells only, yDC/T cells only, Ova/T cells, and Rap/T cells, none of which supported proliferation (**Figure 7C**). Robust proliferation of T cells (=84.4%) was induced by yDCs pulsed with Ova. Coculture of yDCs with PgDCexo ablated T cell proliferation by 51%. This was reversed with rapamycin. Direct infection of yDCs with *P. gingivalis* inhibited T cell proliferation to Ova by 30%, which was restored with rapamycin. Control iDC exo from immature yDCs did not alter T cell proliferation (**Figure 7B**). Summary graphs are shown in **Figure 7D**.

## Discussion

Periodontitis **(**PD), a disease of inflammaging, is comorbid with other age-related diseases (Eke et al., [[NoYear]]; Carrion et al., 2012; Michaud et al., 2017; Teixeira et al., 2017; Zhang et al., 2019) but underlying mechanisms remain to be elucidated. The goals of the present report were to examine whether immune cells, representative of those at the oral mucosal-microbe interface in PD (Cutler and Teng, 2007; Hovav, 2014; Dutzan et al., 2016), are vulnerable to CS *in vitro*, as induced by the dysbiotic pathogen *P. gingivalis* (Hajishengallis et al., 2011; Hajishengallis et al., 2012; Dutzan et al., 2018), and whether this occurs by both direct means (i.e. through infection of target cells) and indirect means (i.e. through induction of the exosomal SASP.

Inflammatory DCs convey *P.gingivalis* and other microbes to distant sites (Carrion et al., 2012; Rajendran et al., 2019), which may have profound implications for age-related comorbid diseases associated with PD. *P. gingivalis* directly invades DCs, hyperactivating the Akt-1/Foxo1 pathway (Arjunan et al., 2016; Arjunan et al., 2018; Meghil et al., 2019). This pathway: Akt1, pFOXO1, FOXP3, IDO1, BIM and Bcl-2, is hyperactivated in PD in humans (Arjunan et al., 2018) and in mice (Song et al., 2017), and has been recognized for its anti-apoptotic, immunosuppressive functions in DCs, leading to disruption of immune homeostasis in PD (Arjunan et al., 2018). However, the link to premature senescence (Nogueira et al., 2008) vis á vis *P.gingivalis* infection was not appreciated at the time. Accordingly, the focus here was on analysis of bone marrow derived DCs from young (yDCs) and old (oDCs) mice, by subjecting them to coculture with prototypic CS inducer doxorubicin or *P.gingivalis* 381, followed by CS profiling (Gorgoulis et al., 2019). Interestingly, yDCs were provoked to premature CS, evidenced by significant activation of SA-B-Gal, p16 ^INK4A^, p53, and p21^Waf1/Clip1^. Further, our data suggest that direct ‘premature aging’ of yDCs is mediated by invasion of *P.gingivalis*. This was confirmed by pre-treatment of yDCs with cytochalasin D, which reduced invasion, reversed SA-B-Gal response and restored the MHCII, CD86 and CD80 response.

Age-related impairment of the immune response (Hajishengallis, 2010; Boots et al., 2013; Ebersole et al., 2016; Preshaw et al., 2017) is known as immune senescence, and has been reported in monocytes, macrophages, DCs and T cells [reviewed in (Linton and Thoman, 2014)]. This is coupled with the constitutive release of inflammatory mediators, cytokines, and chemokines, leading to elevated basal inflammation or “inflammaging” (Ebersole et al., 2016). DCs from aged mice have impaired antigen-presenting function (Moretto et al., 2008; Pereira et al., 2011; Li et al., 2012), decreased migratory capacity (Wu et al., 2016), and increased production of inflammatory cytokines (Agrawal et al., 2012; Ebersole et al., 2016). However, the role of aged DCs in immune senescence in response to etiological agents of PD are presently unclear. Results revealed elevated basal levels of senescence markers in oDCs relative to yDCs. However, it only reached significance level in P53 marker. Nonetheless, these elevations in the basal levels were sufficient to induce a more exaggerated response of oDCs to *P.gingivalis* induction of P21 and SA-B-gal. Moreover, significant lower constitutive expression of basal levels of MHCII and co-stimulatory molecule CD86 in oDCs relative to yDCs shown here are consistent with DC immune senescence accompanied with aging (Shurin et al., 2007). Interestingly, *P.gingivalis*-induced SA-B-gal and p21^Waf1/Clip1^ and impaired maturation were more aggravated in oDCs relative to yDCs, abrogated with a senotherapeutic agent, Rapamycin. This suggests that a bidirectional relationship of PD and CS might exist, wherein inducers of PD can promote CS and CS accompanied with aging can promote PD.

CS is traditionally ascribed to cell cycle arrest in proliferating cells, but can also occur in terminally differentiated cells (Herranz and Gil, 2018; Sapieha and Mallette, 2018; Wengerodt et al., 2019) such as neurons (Jurk et al., 2012), hepatocytes (Kang et al., 2011), osteocytes (Aquino-Martinez et al., 2020), and macrophages (Hall et al., 2016). Macrophages from aged mice upregulate expression of p16 ^INK4A^ and SA-B-gal (Hall et al., 2016), as do macrophages isolated from peritoneal inflammation (Liu et al., 2019). However, macrophages can express elevated p16 ^INK4A^ and SA-B-gal due to physiological stimuli which do not necessarily indicate senescence (Hall et al., 2017), thus necessitating that a more comprehensive analysis of senescence be conducted. Thus, a functional analysis of yDCs ‘prematurely aged’ by *P. gingivalis* was performed, revealing a profound impairment of maturation functions, and inability to stimulate antigen specific T cell proliferation, similarly to oDCs.

Microbial-induced senescence has been variously attributed to DNA damage by bacterial genotoxins (Aquino-Martinez et al., 2020), oxidative stress induction and ROS production (Aquino-Martinez et al., 2020; Humphreys et al., 2020) or persistent activation of TLR4-NF-KB-P21-P53 pathway (Feng et al., 2014). In the present study, the impairment of maturation of yDCs and oDCs, was accompanied by a robust IL-1b, TNFa and IL6 response, consistent with an inflammasome mediated response to *P.gingivalis* (Arjunan et al., 2016; Tyagi et al., 2017). NLR pyrin domain-containing 3 (NLRP3) inflammasome activation is involved in senescence induction (Luo et al., 2019; Shi et al., 2019) and its inhibition suppresses CS in mesenchymal stromal cells (Shi et al., 2019). NLRP3 inflammasome could play a potential role in *P.gingivalis*-induced immune senescence in DCs, however, further analysis of caspase-1 and/or caspase 11 (Paroli et al., 2018) activation are required to examine such a role.

Induction of the exosomal SASP is an indirect, yet more powerful mechanism by which *P. gingivalis* amplifies senescence in bystander cells. The SASP consists of secreted inflammatory cytokines, chemokines, growth factors and extracellular vesicles (EV**)** (e.g. exosomes), collectively called the secretome. The secretome can amplify CS signaling in an autocrine fashion or can transmit CS to bystander cells in a paracrine fashion (Acosta et al., 2013). Senescent fibroblasts *via* the SASP transmit an immunosuppressive tumorigenic response in mice (Ruhland et al., 2016). Exosomes are a particularly active component of SASP (Jakhar and Crasta, 2019). Infection alters the exosome biogenesis and number from the host cell (Zhang et al., 2018). Indeed, an increase in the number of generated exosomes was observed from DCs infected with *P.gingivalis*, through as yet unclear mechanisms. Presumably, this involves deployment of endocytic pathways of exosome biogenesis by pathogenic bacteria. Autophagy inhibition promotes an increase in exosome secretion to compensate for the accumulation of damaged proteins or organelles, whilst activation of autophagy reduces exosome release due to the fusion of MVB and autophagosomes (Tian et al., 2019). Consistent with this notion is our observation that *P.gingivalis*-induced exosome release was ablated by senolytic (and pro-autophagy agent) rapamycin.

Exosomes derived from infected cells carry bacterial RNA and protein (Zhang et al., 2018), and can contribute to spread of infection and disease pathologies (Zhang et al., 2018). The DC invasin Mfa1 fimbrial protein (Zeituni et al., 2009; Zeituni et al., 2010) was detected on PgDCexo by immunoblot and confirmed by immuno-gold transmission electron microscopy. Colocalization of Mfa1 with the tetraspanin CD81, a well-defined eukaryotic exosomal marker, suggests a common pathway of biogenesis. Studies have shown that outer membrane vesicles (OMV) secreted by bacteria range from 50-250 nm (Xie, 2015) and contribute to the virulence of the bacteria by carrying a number of antigenic proteins from the parent cell (Schwechheimer and Kuehn, 2015). Thus, a minor contribution of outer membrane vesicles (OMV) from Pg cannot be ruled out. However, based on results of anti- CD81, CD9, and CD63- linked magnetic bead separation of PgDC exo (**Figure S1**) only 0.01% of the total particles could potentially be Pg OMVs. Moreover, immunoblot analysis indicates that the Mfa-1 protein incorporated in the eluate while barely detectable in the run through (**Figure S1C**). These results indicate that the majority of PgDC exo are DC-derived exosomes that contain Pg Mfa-1 fimbrial adhesin, through an as yet undefined biogenesis pathway. Whether the Mfa-1 is present on the surface membrane of the exosome and/or present inside the exosomal lumen remains to be determined. Previously, our group has shown that exosomes protect their protein cargo against proteolytic degradation (Elashiry et al., 2020). *P**.gingivalis*, through its glycosylated mfa1 fimbriae, binds to C-type lectin receptor CD209 to invade DCs (Zeituni et al., 2009; Zeituni et al., 2010; El-Awady et al., 2015; El-Awady et al., 2015; El-Awady et al., 2019). This activates mTORC1 dependent anti-autophagy signaling pathway (El-Awady et al., 2015; Arjunan et al., 2016; Arjunan et al., 2018; Meghil et al., 2019). Thus, the presence of Mfa-1 on/in DC exosomes should confer distinct functional advantage for penetration into tissues and blood for enhanced and sustainable bacterial virulence.

Perhaps most significantly, PgDCexo were taken up by recipient (bystander) yDCs, promoting their senescence induction, by shutting down maturation and antigen presenting functions. Further studies are required to dissect the mechanisms involved, presumably by a combination of direct surface receptor binding, endocytosis, and intracellular signaling (Jakhar and Crasta, 2019; Elashiry et al., 2020). In this context, the microRNA (miRNA) content of PgDCexo, is worth mentioning, as it results from a complex sorting process during cellular biogenesis and export (Villarroya-Beltri et al., 2013). MiRNAs are especially active in post transcriptional regulation of genes (Stark et al., 2008) of CS and aging (reviewed in (Smith-Vikos and Slack, 2012)). Reported functions of miRNAs induced in PgDCexo include inhibition of beclin-mediated autophagy (Chatterjee et al., 2014), repression of ULK1 and LC3I/II (Duan et al., 2015) (miR17-5p), prevention of autophagy-dependent eradication of intracellular bacteria (Lu et al., 2014) (miR106B). Four-fold upregulation of miRNA 132-3p in PgDCexo (p<0.05), is consistent with aged bone-marrow derived DCs (BMDC). Other miRNAs in aged BMDC under normal and activated conditions include miR-155, miR-223, miR-146a, miR-146b, miR-132, miR-142-5p, and miR-142-3p. A frequently identified miRNA in PgDCexo was miR-24-3p, also identified in saliva exosomes of those with advanced age (p = 0.042), and with periodontal disease progression (Machida et al., 2015). Ten miRNAs recently identified to be deregulated early, in another disease of aging Alzheimer’s disease (Swarbrick et al., 2019), recently linked to *P.gingivalis* infections and periodontitis (Ryder, 2020). These include hsa-mir-107, hsa-mir-26b, hsa-mir-30e, hsa-mir-34a, hsa-mir-485, hsa-mir200c, hsa-mir-210, hsa-mir-146a, hsa-mir-34c and hsa-mir-125b.

In conclusion, *P. gingivalis* induces premature senescence through direct cellular invasion(autocrine) and by stimulating secretion of inflammatory exosomes that amplify immune senescence in paracrine to bystander cells. Our study signifies a potential role of exosome-mediated signaling in the pathogenesis of periodontal disease. In vivo studies are underway to evaluate such role and further dissect the mechanism of PgDCexo-induced paracrine senescence in PD.

## Data Availability Statement

The raw data supporting the conclusions of this article will be made available by the authors, in response to a detailed written request.

## Ethics Statement

The Institutional Animal Care and Use Committee (IACUC) of Augusta University (protocol # 2013-0586) approved all experimental procedures on C57BL/6 mice.

## Author Contributions

Study design (CC, RE, and ME). Data generation, analysis, and interpretation (RE, ME, CC, and YL). Intellectual input (AE, YL, and MH). Manuscript preparation (CC and RE). All authors contributed to the article and approved the submitted version.

## Funding

This project was supported by a grant from the Carlos and Marguerite Mason Trust and by an Intramural grant from the Office of the Vice President for Research, Augusta University.

## Conflict of Interest

The authors declare that the research was conducted in the absence of any commercial or financial relationships that could be construed as a potential conflict of interest.

## Supplementary Material

Supplementary Figure 1
**(A)** Schematic diagram showing the work flow of magnetic separation of EXO labeled with CD81, CD9 and CD63. **(B)** Nano tracking analysis (NTA) to determine Exo number and size distribution in nanometer(nm) within the run through. Y-axis is linear in all three histograms, with the range being, from left to right: 10^11^, 10^8^, 10^7.^ **(C)** WB analysis of proteins in the eluate and the run through after magnetic separation.
