# Supplementary figures and images for: Porphyromonas gingivalis Provokes Exosome Secretion and Paracrine Immune Senescence in Bystander Dendritic Cells
Source: Front Cell Infect Microbiol. 2021 Jun 1;11:669989. doi: 10.3389/fcimb.2021.669989 (PMC8204290; doi:10.3389/fcimb.2021.669989)

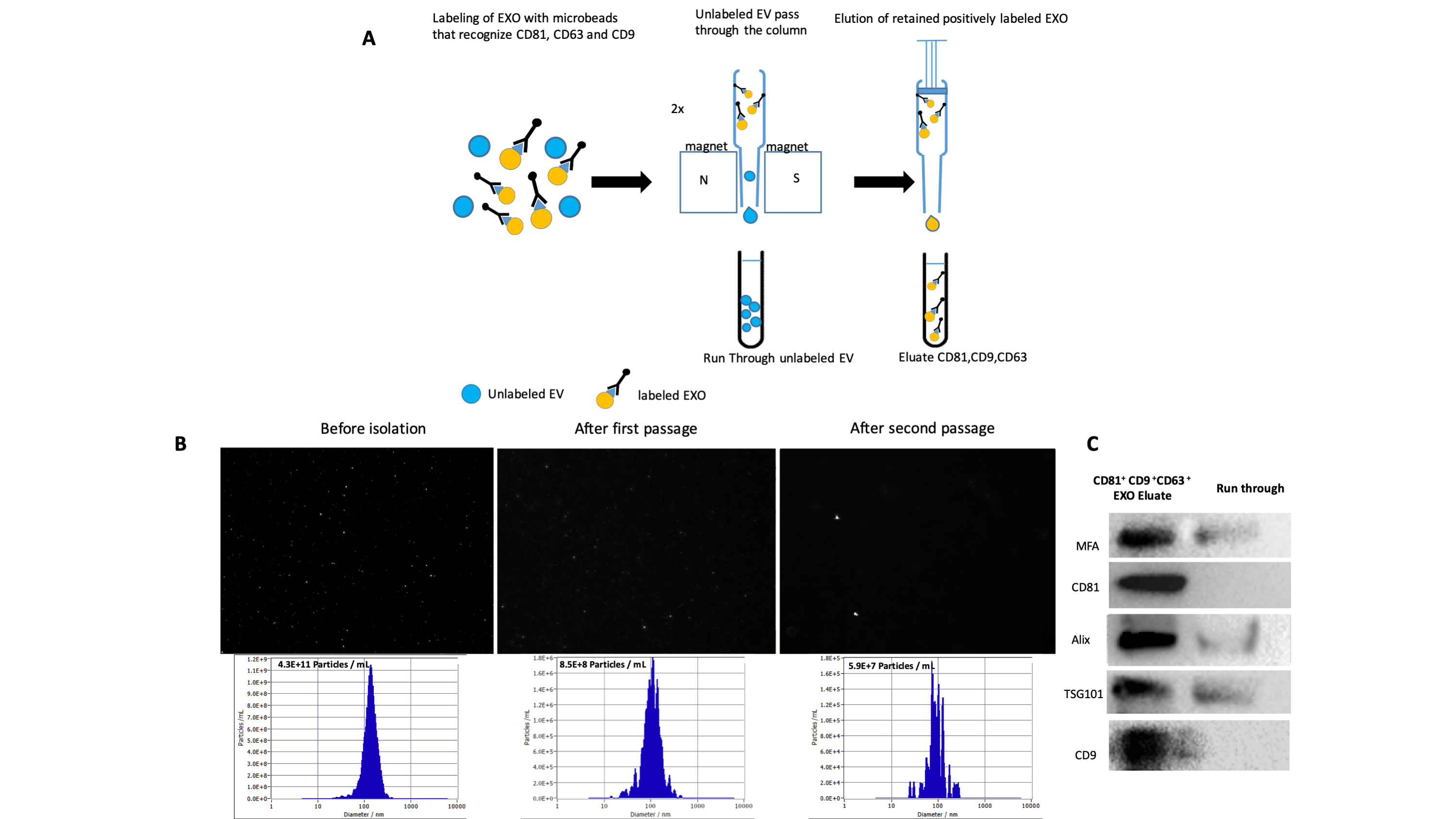

Supplement: Supplementary Figure 1 — (A) Schematic diagram showing the work flow of magnetic separation of EXO labeled with CD81, CD9 and CD63. (B) Nano tracking analysis (NTA) to determine Exo number and size distribution in nanometer(nm) within the run through. Y-axis is linear in all three histograms, with the range being, from left to right: 1011, 108, 107. (C) WB analysis of proteins in the eluate and the run through after magnetic separation. [file Image_1.tiff]
