# Supplementary material for: Porphyromonas gingivalis Provokes Exosome Secretion and Paracrine Immune Senescence in Bystander Dendritic Cells
Source: Front Cell Infect Microbiol. 2021 Jun 1;11:669989. doi: 10.3389/fcimb.2021.669989 (PMC8204290; doi:10.3389/fcimb.2021.669989)
Supplement: Supplementary file 2 [file DataSheet_1.pdf]

Transcript ID(Array Design)	p-value(Pg DCs EXO vs. IDCs EXO)	Fold-Change(Pg DCs EXO vs. IDCs EXO)	Fold-Change(PgDCs EXO vs. IDCs EXO) (Description)
mmu-miR-7686-5p	0.221662	-8.89226	IM+PG down vs IM
mmu-miR-6937-5p	0.00364356	-5.97725	IM+PG down vs IM
mmu-miR-7047-5p	0.0603667	-5.60967	IM+PG down vs IM
mmu-miR-1934-3p	0.0532507	-5.45053	IM+PG down vs IM
mmu-miR-7648-3p	0.0381767	-5.43617	IM+PG down vs IM
mmu-miR-6970-5p	0.0726036	-5.38736	IM+PG down vs IM
mmu-miR-7658-5p	0.0531123	-5.2432	IM+PG down vs IM
mmu-miR-3620-5p	0.229898	-4.86296	IM+PG down vs IM
mmu-miR-7221-3p	0.23828	-4.82106	IM+PG down vs IM
mmu-miR-346-3p	0.114584	-4.38773	IM+PG down vs IM
mmu-miR-3102-5p	0.0555091	-4.35322	IM+PG down vs IM
mmu-miR-7235-5p	0.0163528	-4.0757	IM+PG down vs IM
mmu-miR-762	0.135797	-3.81281	IM+PG down vs IM
mmu-miR-690	0.202291	-3.2503	IM+PG down vs IM
mmu-miR-155-5p	0.0449271	-2.86175	IM+PG down vs IM
mmu-miR-7238-5p	0.027082	-2.66956	IM+PG down vs IM
mmu-miR-6909-5p	0.0634542	-2.48152	IM+PG down vs IM
mmu-miR-6944-5p	0.275264	-2.34358	IM+PG down vs IM
mmu-miR-328-5p	0.0236299	-2.3104	IM+PG down vs IM
mmu-miR-705	0.213561	-2.26764	IM+PG down vs IM
mmu-miR-501-3p	0.327052	-2.25156	IM+PG down vs IM
mmu-miR-7044-5p	0.12651	-2.12162	IM+PG down vs IM
mmu-miR-6931-5p	0.233608	-2.10149	IM+PG down vs IM
mmu-miR-5126	0.0259735	-2.07952	IM+PG down vs IM
mmu-miR-3077-5p	0.00913228	-2.06893	IM+PG down vs IM
mmu-miR-7023-5p	0.113949	-2.05197	IM+PG down vs IM
mmu-miR-3547-5p	0.0175812	-2.03986	IM+PG down vs IM
mmu-miR-6991-5p	0	-2.00707	IM+PG down vs IM
mmu-miR-7687-5p	0.262462	-1.9675	IM+PG down vs IM
mmu-miR-6970-3p	0.267102	-1.8886	IM+PG down vs IM
mmu-miR-6987-3p	0.0206953	-1.83748	IM+PG down vs IM
mmu-miR-8109	0.135159	-1.78686	IM+PG down vs IM
mmu-miR-6240	0.158675	-1.75289	IM+PG down vs IM
mmu-miR-675-5p	0.15835	-1.75022	IM+PG down vs IM
mmu-miR-6959-5p	0.11819	-1.73577	IM+PG down vs IM
mmu-miR-7085-5p	0.353361	-1.73547	IM+PG down vs IM
mmu-miR-6349	0	-1.72898	IM+PG down vs IM
mmu-miR-147-3p	0	-1.70869	IM+PG down vs IM

mmu-miR-7118-5p	0.287403	-1.70346	IM+PG down vs IM
mmu-miR-1968-3p	0.214591	-1.69036	IM+PG down vs IM
mmu-mir-6946	0	-1.68263	IM+PG down vs IM
mmu-miR-6911-5p	0.147226	-1.6816	IM+PG down vs IM
mmu-miR-667-5p	0.0562083	-1.67889	IM+PG down vs IM
mmu-miR-7003-3p	0.332797	-1.67136	IM+PG down vs IM
mmu-miR-7115-3p	0.0764824	-1.66344	IM+PG down vs IM
mmu-miR-3547-3p	0.312207	-1.64132	IM+PG down vs IM
mmu-miR-3968	0	-1.63502	IM+PG down vs IM
mmu-miR-7654-3p	0	-1.63502	IM+PG down vs IM
mmu-miR-291b-3p	0.21217	-1.61956	IM+PG down vs IM
mmu-miR-6906-5p	0	-1.61033	IM+PG down vs IM
mmu-miR-504-3p	0.31582	-1.61016	IM+PG down vs IM
mmu-miR-1943-3p	0.0449297	-1.6038	IM+PG down vs IM
mmu-miR-6910-5p	0.022971	-1.59665	IM+PG down vs IM
mmu-mir-3070b	0.0567623	-1.59604	IM+PG down vs IM
mmu-miR-5100	0	-1.58551	IM+PG down vs IM
mmu-miR-344e-5p	0	-1.56688	IM+PG down vs IM
mmu-miR-344h-5p	0	-1.56688	IM+PG down vs IM
mmu-miR-7030-5p	0.180906	-1.56091	IM+PG down vs IM
mmu-miR-3544-5p	0.180954	-1.56072	IM+PG down vs IM
mmu-mir-219a-1	0.0398973	-1.55228	IM+PG down vs IM
mmu-miR-802-5p	0.0456346	-1.54885	IM+PG down vs IM
mmu-miR-1188-3p	0.046483	-1.54835	IM+PG down vs IM
mmu-miR-16-1-3p	0.0403608	-1.54444	IM+PG down vs IM
mmu-miR-714	0.349836	-1.54029	IM+PG down vs IM
mmu-miR-8119	0.235073	-1.52457	IM+PG down vs IM
mmu-mir-5099	0	-1.52003	IM+PG down vs IM
mmu-miR-1982-5p	0.156164	-1.51242	IM+PG down vs IM
mmu-miR-544-3p	0.267527	-1.50938	IM+PG down vs IM
mmu-let-7e-5p	0.247139	-1.50855	IM+PG down vs IM
mmu-mir-6978	0.212088	-1.50809	IM+PG down vs IM
mmu-miR-6914-3p	0.345445	-1.4968	IM+PG down vs IM
mmu-miR-770-5p	0.198921	-1.49567	IM+PG down vs IM
mmu-miR-7229-3p	0.0436605	-1.49439	IM+PG down vs IM
mmu-mir-485	0.153295	-1.4909	IM+PG down vs IM
mmu-miR-7668-5p	0.153424	-1.49038	IM+PG down vs IM
mmu-miR-1931	0.263575	-1.48489	IM+PG down vs IM
mmu-mir-26a-1	0.178556	-1.48115	IM+PG down vs IM
mmu-miR-7045-5p	0.410378	-1.47561	IM+PG down vs IM
mmu-miR-195a-3p	0.166356	-1.47298	IM+PG down vs IM
mmu-miR-3971	0	-1.47084	IM+PG down vs IM
mmu-miR-5624-3p	0.0554564	-1.47046	IM+PG down vs IM

mmu-miR-3963	0.170987	-1.46608	IM+PG down vs IM
mmu-mir-8101	0.111251	-1.46472	IM+PG down vs IM
mmu-miR-6990-3p	0.209629	-1.46312	IM+PG down vs IM
mmu-mir-7066	0	-1.46182	IM+PG down vs IM
mmu-miR-210-3p	0.666667	-1.45613	IM+PG down vs IM
mmu-miR-7020-5p	0.232467	-1.45046	IM+PG down vs IM
mmu-miR-3110-5p	0.232467	-1.45046	IM+PG down vs IM
mmu-mir-8101	0.115788	-1.44252	IM+PG down vs IM
mmu-mir-6374	0.175888	-1.44092	IM+PG down vs IM
mmu-miR-6917-5p	0.207215	-1.4371	IM+PG down vs IM
mmu-miR-345-3p	0.266816	-1.43682	IM+PG down vs IM
mmu-miR-6239	0.0735849	-1.43331	IM+PG down vs IM
mmu-miR-101c	0.276876	-1.4323	IM+PG down vs IM
mmu-miR-2861	0.123791	-1.43184	IM+PG down vs IM
mmu-miR-341-5p	0.203119	-1.43143	IM+PG down vs IM
mmu-mir-6926	0.203366	-1.43076	IM+PG down vs IM
mmu-miR-211-3p	0	-1.43064	IM+PG down vs IM
mmu-mir-6975	0.223952	-1.4252	IM+PG down vs IM
mmu-miR-7081-5p	0.481752	-1.42456	IM+PG down vs IM
mmu-mir-6935	0.279473	-1.42158	IM+PG down vs IM
mmu-miR-6922-5p	0.182289	-1.42158	IM+PG down vs IM
mmu-mir-484	0.0755763	-1.42149	IM+PG down vs IM
mmu-mir-195a	0.0753199	-1.42137	IM+PG down vs IM
mmu-miR-7229-5p	0.214839	-1.4193	IM+PG down vs IM
mmu-mir-7654	0	-1.40883	IM+PG down vs IM
mmu-miR-34a-5p	0	-1.40883	IM+PG down vs IM
mmu-mir-669n	0.165242	-1.40815	IM+PG down vs IM
mmu-miR-7092-5p	0.187913	-1.40583	IM+PG down vs IM
mmu-miR-379-3p	0.238831	-1.40534	IM+PG down vs IM
mmu-mir-6418	0.233528	-1.40194	IM+PG down vs IM
mmu-let-7i-5p	0.442661	-1.40139	IM+PG down vs IM
mmu-mir-301b	0.102566	-1.4009	IM+PG down vs IM
mmu-miR-339-5p	0.169481	-1.39558	IM+PG down vs IM
mmu-miR-182-5p	0.169481	-1.39558	IM+PG down vs IM
mmu-mir-8114	0.276872	-1.39558	IM+PG down vs IM
mmu-miR-338-5p	0.218642	-1.39281	IM+PG down vs IM
mmu-mir-501	0.118568	-1.38796	IM+PG down vs IM
mmu-mir-21a	0	-1.38748	IM+PG down vs IM
mmu-miR-351-5p	0	-1.38748	IM+PG down vs IM
mmu-miR-429-3p	0	-1.38748	IM+PG down vs IM
mmu-miR-6935-5p	0	-1.38748	IM+PG down vs IM
mmu-miR-216b-3p	0.0715234	-1.38648	IM+PG down vs IM
mmu-miR-760-3p	0.053679	-1.38608	IM+PG down vs IM

mmu-miR-7220-3p	0.261811	-1.3854	IM+PG down vs IM
mmu-miR-3472	0.261811	-1.3854	IM+PG down vs IM
mmu-miR-665-3p	0.199483	-1.38402	IM+PG down vs IM
mmu-miR-880-3p	0.062641	-1.38258	IM+PG down vs IM
mmu-mir-3074-2	0.062641	-1.38258	IM+PG down vs IM
mmu-miR-8095	0.219367	-1.38185	IM+PG down vs IM
mmu-miR-223-5p	0.139207	-1.38012	IM+PG down vs IM
mmu-miR-466m-5p	0.0545205	-1.37908	IM+PG down vs IM
mmu-miR-669m-5p	0.0545205	-1.37908	IM+PG down vs IM
mmu-miR-8110	0.289678	-1.37858	IM+PG down vs IM
mmu-miR-3065-3p	0.0382854	-1.3723	IM+PG down vs IM
mmu-miR-7689-3p	0.483672	-1.36749	IM+PG down vs IM
mmu-miR-7088-3p	0.457253	-1.36544	IM+PG down vs IM
mmu-miR-196a-1-3p	0.252422	-1.36427	IM+PG down vs IM
mmu-mir-8120	0.223321	-1.36239	IM+PG down vs IM
mmu-miR-6950-3p	0.0658067	-1.36106	IM+PG down vs IM
mmu-miR-7017-5p	0.509716	-1.36097	IM+PG down vs IM
mmu-mir-99b	0.452763	-1.35941	IM+PG down vs IM
mmu-mir-3081	0	-1.35728	IM+PG down vs IM
mmu-mir-7210	0	-1.35728	IM+PG down vs IM
mmu-mir-23b	0	-1.35728	IM+PG down vs IM
mmu-mir-199a-1	0	-1.35728	IM+PG down vs IM
mmu-mir-221	0	-1.35728	IM+PG down vs IM
mmu-mir-7069	0	-1.35728	IM+PG down vs IM
mmu-mir-7077	0	-1.35728	IM+PG down vs IM
mmu-mir-7087	0	-1.35728	IM+PG down vs IM
mmu-miR-330-5p	0	-1.35728	IM+PG down vs IM
mmu-miR-381-5p	0	-1.35728	IM+PG down vs IM
mmu-miR-6516-3p	0	-1.35728	IM+PG down vs IM
mmu-miR-6908-3p	0	-1.35728	IM+PG down vs IM
mmu-miR-344g-5p	0	-1.35728	IM+PG down vs IM
mmu-miR-6931-3p	0.450801	-1.35686	IM+PG down vs IM
mmu-miR-8100	0.0871636	-1.3544	IM+PG down vs IM
mmu-miR-1843a-3p	0.0777135	-1.35055	IM+PG down vs IM
mmu-miR-881-3p	0	-1.34964	IM+PG down vs IM
mmu-miR-669l-3p	0.470722	-1.34782	IM+PG down vs IM
mmu-miR-7072-3p	0.470722	-1.34782	IM+PG down vs IM
mmu-miR-290a-3p	0.470722	-1.34782	IM+PG down vs IM
mmu-miR-21b	0.470722	-1.34782	IM+PG down vs IM
mmu-mir-3074-2	0.0681259	-1.34674	IM+PG down vs IM
mmu-miR-3473f	0.0681259	-1.34674	IM+PG down vs IM
mmu-mir-6401	0.250876	-1.34378	IM+PG down vs IM
mmu-miR-302b-5p	0.465687	-1.3409	IM+PG down vs IM

mmu-miR-27b-3p	0	-1.33906	IM+PG down vs IM
mmu-mir-130c	0.0579298	-1.33669	IM+PG down vs IM
mmu-mir-7007	0	-1.33616	IM+PG down vs IM
mmu-miR-7046-3p	0	-1.33616	IM+PG down vs IM
mmu-mir-692-1	0.331285	-1.33406	IM+PG down vs IM
mmu-mir-692-2	0.331285	-1.33406	IM+PG down vs IM
mmu-mir-692-3	0.331285	-1.33406	IM+PG down vs IM
mmu-miR-669k-5p	0.27043	-1.33311	IM+PG down vs IM
mmu-miR-325-5p	0.0529377	-1.33147	IM+PG down vs IM
mmu-let-7a-1	0.625425	-1.32876	IM+PG down vs IM
mmu-mir-1839	0.0111226	-1.32587	IM+PG down vs IM
mmu-miR-1194	0.227545	-1.32573	IM+PG down vs IM
mmu-miR-466d-3p	0.423235	-1.3256	IM+PG down vs IM
mmu-miR-669d-3p	0.453361	-1.32543	IM+PG down vs IM
mmu-miR-6991-3p	0	-1.32451	IM+PG down vs IM
mmu-mir-3072	0	-1.32423	IM+PG down vs IM
mmu-miR-590-3p	0	-1.32423	IM+PG down vs IM
mmu-miR-6975-5p	0	-1.32423	IM+PG down vs IM
mmu-miR-7664-5p	0	-1.32423	IM+PG down vs IM
mmu-mir-200a	0	-1.32423	IM+PG down vs IM
mmu-miR-6414	0.432228	-1.32397	IM+PG down vs IM
mmu-miR-7017-3p	0	-1.32211	IM+PG down vs IM
mmu-miR-7080-5p	0.27798	-1.32136	IM+PG down vs IM
mmu-miR-20a-3p	0.438878	-1.31849	IM+PG down vs IM
mmu-mir-1a-1	0.203208	-1.31655	IM+PG down vs IM
mmu-miR-31-3p	0	-1.31257	IM+PG down vs IM
mmu-mir-450a-1	0.0578639	-1.31194	IM+PG down vs IM
mmu-miR-6968-5p	0.17671	-1.31184	IM+PG down vs IM
mmu-miR-7049-3p	0.343191	-1.3111	IM+PG down vs IM
mmu-miR-18b-5p	0.438717	-1.30934	IM+PG down vs IM
mmu-miR-7679-3p	0.231405	-1.30932	IM+PG down vs IM
mmu-mir-32	0.437892	-1.3085	IM+PG down vs IM
mmu-miR-6337	0.239408	-1.30564	IM+PG down vs IM
mmu-miR-7001-5p	0.666667	-1.30357	IM+PG down vs IM
mmu-mir-705	0	-1.30071	IM+PG down vs IM
mmu-mir-7688	0	-1.30071	IM+PG down vs IM
mmu-miR-187-5p	0	-1.30071	IM+PG down vs IM
mmu-miR-7119-3p	0	-1.30071	IM+PG down vs IM
mmu-miR-219c-5p	0	-1.30071	IM+PG down vs IM
mmu-mir-218-2	0.077212	-1.30001	IM+PG down vs IM
mmu-mir-6372	0.077212	-1.30001	IM+PG down vs IM
mmu-miR-1966-3p	0.077212	-1.30001	IM+PG down vs IM
mmu-mir-671	0.077212	-1.30001	IM+PG down vs IM

mmu-miR-350-3p	0.077212	-1.30001	IM+PG down vs IM
mmu-mir-3065	0.270952	-1.29871	IM+PG down vs IM
mmu-miR-149-3p	0.114372	-1.29601	IM+PG down vs IM
mmu-miR-6967-3p	0.0107791	-1.29556	IM+PG down vs IM
mmu-mir-719	0.199158	-1.29358	IM+PG down vs IM
mmu-miR-3109-3p	0.414716	-1.29196	IM+PG down vs IM
mmu-mir-3473a	0.396562	-1.29189	IM+PG down vs IM
mmu-mir-669f	0.252052	-1.2887	IM+PG down vs IM
mmu-miR-7064-5p	0.303246	-1.28666	IM+PG down vs IM
mmu-miR-23b-3p	0.150124	-1.28191	IM+PG down vs IM
mmu-mir-7650	0.406887	-1.28086	IM+PG down vs IM
mmu-miR-509-5p	0.197273	-1.27891	IM+PG down vs IM
mmu-miR-3072-3p	0	-1.27623	IM+PG down vs IM
mmu-miR-3092-3p	0	-1.27623	IM+PG down vs IM
mmu-miR-3473a	0	-1.27623	IM+PG down vs IM
mmu-miR-7008-5p	0	-1.27623	IM+PG down vs IM
mmu-miR-200c-5p	0	-1.27623	IM+PG down vs IM
mmu-mir-466i	0	-1.27623	IM+PG down vs IM
mmu-miR-7085-3p	0.786923	-1.27613	IM+PG down vs IM
mmu-miR-6974-5p	0.265466	-1.27541	IM+PG down vs IM
mmu-miR-10a-5p	0.35911	-1.2752	IM+PG down vs IM
mmu-mir-539	0.355023	-1.2727	IM+PG down vs IM
mmu-miR-7025-5p	0.666667	-1.27265	IM+PG down vs IM
mmu-miR-3074-2-3p	0.0727316	-1.27185	IM+PG down vs IM
mmu-miR-671-3p	0.0727316	-1.27185	IM+PG down vs IM
mmu-miR-6962-5p	0.364401	-1.26959	IM+PG down vs IM
mmu-mir-207	0.302866	-1.26933	IM+PG down vs IM
mmu-mir-6965	0.258014	-1.26736	IM+PG down vs IM
mmu-miR-3475-3p	0.360401	-1.26714	IM+PG down vs IM
mmu-mir-381	0.360401	-1.26714	IM+PG down vs IM
mmu-mir-16-1	0.34471	-1.26667	IM+PG down vs IM
mmu-miR-6943-3p	0.388655	-1.26271	IM+PG down vs IM
mmu-miR-5119	0.388655	-1.26271	IM+PG down vs IM
mmu-mir-299a	0.350033	-1.26109	IM+PG down vs IM
mmu-miR-7663-5p	0.327141	-1.2591	IM+PG down vs IM
mmu-mir-6392	0.328339	-1.25783	IM+PG down vs IM
mmu-miR-758-3p	0.328339	-1.25783	IM+PG down vs IM
mmu-miR-7659-3p	0.343871	-1.25767	IM+PG down vs IM
mmu-mir-381	0.371111	-1.25632	IM+PG down vs IM
mmu-miR-3073b-3p	0.341286	-1.25628	IM+PG down vs IM
mmu-mir-378d	0.19357	-1.25628	IM+PG down vs IM
mmu-miR-6971-5p	0.354827	-1.25621	IM+PG down vs IM
mmu-miR-1930-3p	0.666667	-1.25587	IM+PG down vs IM

mmu-miR-7047-3p	0.338666	-1.25489	IM+PG down vs IM
mmu-mir-6987	0.260056	-1.2543	IM+PG down vs IM
mmu-mir-3473c	0.333333	-1.25212	IM+PG down vs IM
mmu-mir-6380	0.333333	-1.25212	IM+PG down vs IM
mmu-miR-466h-3p	0.703596	-1.2519	IM+PG down vs IM
mmu-mir-7033	0	-1.25175	IM+PG down vs IM
mmu-miR-1942	0	-1.25175	IM+PG down vs IM
mmu-miR-6990-5p	0	-1.25175	IM+PG down vs IM
mmu-mir-8112	0.262498	-1.25134	IM+PG down vs IM
mmu-mir-3086	0.360803	-1.25032	IM+PG down vs IM
mmu-miR-101b-3p	0.360803	-1.25032	IM+PG down vs IM
mmu-let-7k	0.360804	-1.25032	IM+PG down vs IM
mmu-mir-218-1	0.284607	-1.24887	IM+PG down vs IM
mmu-miR-329-5p	0.338483	-1.24745	IM+PG down vs IM
mmu-mir-3971	0.323237	-1.24711	IM+PG down vs IM
mmu-miR-6913-5p	0.323237	-1.24711	IM+PG down vs IM
mmu-miR-539-3p	0.322514	-1.24677	IM+PG down vs IM
mmu-miR-1a-1-5p	0.365708	-1.24564	IM+PG down vs IM
mmu-miR-7082-5p	0.36	-1.24563	IM+PG down vs IM
mmu-miR-6976-3p	0.385229	-1.24301	IM+PG down vs IM
mmu-miR-7650-3p	0.392965	-1.24196	IM+PG down vs IM
mmu-mir-676	0.344491	-1.2416	IM+PG down vs IM
mmu-miR-7236-5p	0.344491	-1.2416	IM+PG down vs IM
mmu-mir-3073a	0.344153	-1.24143	IM+PG down vs IM
mmu-miR-1955-5p	0.507708	-1.24067	IM+PG down vs IM
mmu-miR-302c-3p	0.308157	-1.24014	IM+PG down vs IM
mmu-miR-7082-3p	0.312101	-1.23947	IM+PG down vs IM
mmu-mir-3544	0.346866	-1.23935	IM+PG down vs IM
mmu-mir-7038	0.306287	-1.23932	IM+PG down vs IM
mmu-miR-1199-5p	0.286833	-1.23875	IM+PG down vs IM
mmu-miR-7118-3p	0.375036	-1.23707	IM+PG down vs IM
mmu-miR-3962	0.375036	-1.23707	IM+PG down vs IM
mmu-mir-5135	0.375036	-1.23707	IM+PG down vs IM
mmu-mir-7018	0.375036	-1.23707	IM+PG down vs IM
mmu-mir-499	0.322705	-1.23696	IM+PG down vs IM
mmu-miR-7094-3p	0.333333	-1.23612	IM+PG down vs IM
mmu-miR-7234-5p	0.337157	-1.23355	IM+PG down vs IM
mmu-miR-7213-3p	0.327381	-1.23334	IM+PG down vs IM
mmu-miR-411-3p	0.292065	-1.23329	IM+PG down vs IM
mmu-miR-1839-3p	0.708818	-1.23329	IM+PG down vs IM
mmu-miR-7668-3p	0.269659	-1.23196	IM+PG down vs IM
mmu-miR-465d-5p	0.318938	-1.22956	IM+PG down vs IM
mmu-mir-147	0.358846	-1.22845	IM+PG down vs IM

mmu-miR-6905-5p	0.358846	-1.22845	IM+PG down vs IM
mmu-mir-470	0.358846	-1.22845	IM+PG down vs IM
mmu-miR-7682-5p	0.358846	-1.22845	IM+PG down vs IM
mmu-mir-6898	0.358846	-1.22845	IM+PG down vs IM
mmu-mir-6998	0.358846	-1.22845	IM+PG down vs IM
mmu-miR-193a-5p	0.358846	-1.22845	IM+PG down vs IM
mmu-mir-148b	0.357133	-1.22759	IM+PG down vs IM
mmu-mir-5103	0.33395	-1.22724	IM+PG down vs IM
mmu-mir-450a-1	0.468488	-1.22525	IM+PG down vs IM
mmu-miR-10b-5p	0.268416	-1.22464	IM+PG down vs IM
mmu-miR-7062-3p	0.348452	-1.22336	IM+PG down vs IM
mmu-mir-1900	0.348452	-1.22336	IM+PG down vs IM
mmu-mir-190a	0.30275	-1.22277	IM+PG down vs IM
mmu-mir-465a	0.151275	-1.22038	IM+PG down vs IM
mmu-mir-465b-1	0.151275	-1.22038	IM+PG down vs IM
mmu-mir-465b-2	0.151275	-1.22038	IM+PG down vs IM
mmu-mir-223	0.341783	-1.22027	IM+PG down vs IM
mmu-mir-7039	0.341783	-1.22027	IM+PG down vs IM
mmu-mir-6935	0.295775	-1.22002	IM+PG down vs IM
mmu-miR-466q	0.377586	-1.21627	IM+PG down vs IM
mmu-miR-7086-3p	0.399973	-1.21616	IM+PG down vs IM
mmu-miR-6982-5p	0.450098	-1.21577	IM+PG down vs IM
mmu-mir-3473g	0.330935	-1.21549	IM+PG down vs IM
mmu-miR-7059-3p	0.374934	-1.21492	IM+PG down vs IM
mmu-miR-7038-5p	0.374934	-1.21492	IM+PG down vs IM
mmu-let-7b-5p	0.204353	-1.21409	IM+PG down vs IM
mmu-miR-7089-5p	0.469139	-1.21345	IM+PG down vs IM
mmu-mir-804	0	-1.21284	IM+PG down vs IM
mmu-miR-877-5p	0	-1.21284	IM+PG down vs IM
mmu-miR-1224-3p	0	-1.21284	IM+PG down vs IM
mmu-miR-741-5p	0	-1.21284	IM+PG down vs IM
mmu-mir-29b-1	0	-1.21272	IM+PG down vs IM
mmu-mir-181a-1	0	-1.21272	IM+PG down vs IM
mmu-mir-181a-1	0	-1.21272	IM+PG down vs IM
mmu-mir-8109	0	-1.21272	IM+PG down vs IM
mmu-miR-6417	0	-1.21272	IM+PG down vs IM
mmu-miR-7687-3p	0	-1.21272	IM+PG down vs IM
mmu-miR-876-5p	0.317075	-1.20981	IM+PG down vs IM
mmu-mir-551b	0.317075	-1.20981	IM+PG down vs IM
mmu-mir-7653	0.317075	-1.20981	IM+PG down vs IM
mmu-mir-7036b	0.265438	-1.2091	IM+PG down vs IM
mmu-miR-224-3p	0.445971	-1.2079	IM+PG down vs IM
mmu-miR-5112	0	-1.2076	IM+PG down vs IM

mmu-miR-496a-3p	0.385535	-1.20663	IM+PG down vs IM
mmu-mir-6389	0.0539244	-1.20615	IM+PG down vs IM
mmu-miR-1231-3p	0.307211	-1.20602	IM+PG down vs IM
mmu-miR-181a-2-3p	0.307095	-1.20597	IM+PG down vs IM
mmu-miR-107-3p	0	-1.20574	IM+PG down vs IM
mmu-mir-350	0.184738	-1.20497	IM+PG down vs IM
mmu-miR-151-5p	0.618083	-1.20419	IM+PG down vs IM
mmu-mir-758	0.349592	-1.20313	IM+PG down vs IM
mmu-miR-3089-3p	0.297786	-1.20257	IM+PG down vs IM
mmu-miR-1970	0.296854	-1.20224	IM+PG down vs IM
mmu-miR-103-2-5p	0.436716	-1.20178	IM+PG down vs IM
mmu-mir-7037	0	-1.20154	IM+PG down vs IM
mmu-miR-7676-3p	0	-1.20154	IM+PG down vs IM
mmu-mir-762	0.466645	-1.20137	IM+PG down vs IM
mmu-miR-5101	0.666667	-1.1999	IM+PG down vs IM
mmu-miR-1936	0.446917	-1.19944	IM+PG down vs IM
mmu-mir-7231	0.415395	-1.19923	IM+PG down vs IM
mmu-mir-6896	0.530162	-1.19912	IM+PG down vs IM
mmu-miR-423-3p	0.423727	-1.19852	IM+PG down vs IM
mmu-miR-466g	0.568395	-1.19473	IM+PG down vs IM
mmu-miR-3091-5p	0.273951	-1.19456	IM+PG down vs IM
mmu-mir-7012	0.301287	-1.19422	IM+PG down vs IM
mmu-miR-6927-3p	0.434091	-1.19141	IM+PG down vs IM
mmu-miR-6945-3p	0.434091	-1.19141	IM+PG down vs IM
mmu-miR-7073-3p	0.434091	-1.19141	IM+PG down vs IM
mmu-miR-7665-3p	0.434091	-1.19141	IM+PG down vs IM
mmu-miR-181d-3p	0	-1.19113	IM+PG down vs IM
mmu-mir-202	0.150058	-1.19008	IM+PG down vs IM
mmu-mir-29b-1	0.120991	-1.18956	IM+PG down vs IM
mmu-miR-7069-5p	0.116515	-1.18859	IM+PG down vs IM
mmu-miR-214-5p	0.30938	-1.18776	IM+PG down vs IM
mmu-miR-136-5p	0.466497	-1.18776	IM+PG down vs IM
mmu-miR-6967-5p	0.469764	-1.18704	IM+PG down vs IM
mmu-miR-7651-5p	0.135049	-1.1867	IM+PG down vs IM
mmu-miR-1934-5p	0.135049	-1.1867	IM+PG down vs IM
mmu-miR-742-3p	0.666667	-1.18635	IM+PG down vs IM
mmu-mir-300	0.443072	-1.18551	IM+PG down vs IM
mmu-mir-7094-1	0.462099	-1.18476	IM+PG down vs IM
mmu-mir-7094-2	0.462099	-1.18476	IM+PG down vs IM
mmu-miR-878-3p	0.462099	-1.18476	IM+PG down vs IM
mmu-miR-6539	0.731103	-1.18396	IM+PG down vs IM
mmu-mir-2861	0.169119	-1.18362	IM+PG down vs IM
mmu-miR-7070-5p	0.1195	-1.18336	IM+PG down vs IM

mmu-miR-6361	0.1195	-1.18336	IM+PG down vs IM
mmu-miR-1928	0.464372	-1.18332	IM+PG down vs IM
mmu-mir-7064	0.238589	-1.18315	IM+PG down vs IM
mmu-let-7f-1	0.419114	-1.1831	IM+PG down vs IM
mmu-mir-434	0.419114	-1.1831	IM+PG down vs IM
mmu-miR-345-5p	0.666667	-1.18285	IM+PG down vs IM
mmu-mir-7065	0.402241	-1.18278	IM+PG down vs IM
mmu-mir-6940	0.233228	-1.18275	IM+PG down vs IM
mmu-mir-682	0	-1.18055	IM+PG down vs IM
mmu-miR-7093-3p	0.157527	-1.17992	IM+PG down vs IM
mmu-miR-7007-5p	0.409349	-1.17821	IM+PG down vs IM
mmu-mir-467f	0.666667	-1.17791	IM+PG down vs IM
mmu-miR-874-5p	0.666667	-1.17791	IM+PG down vs IM
mmu-miR-29b-1-5p	0.428379	-1.17725	IM+PG down vs IM
mmu-miR-433-3p	0.141941	-1.17657	IM+PG down vs IM
mmu-mir-331	0.141941	-1.17657	IM+PG down vs IM
mmu-mir-8091	0.141941	-1.17657	IM+PG down vs IM
mmu-miR-8111	0.458897	-1.17566	IM+PG down vs IM
mmu-miR-335-5p	0.204327	-1.17551	IM+PG down vs IM
mmu-mir-802	0.204327	-1.17551	IM+PG down vs IM
mmu-miR-449a-3p	0.266478	-1.1746	IM+PG down vs IM
mmu-mir-5615-2	0.12575	-1.17326	IM+PG down vs IM
mmu-miR-3070a-5p	0.12575	-1.17326	IM+PG down vs IM
mmu-miR-3070b-5p	0.12575	-1.17326	IM+PG down vs IM
mmu-miR-181b-2-3p	0.12575	-1.17326	IM+PG down vs IM
mmu-miR-6336	0.12575	-1.17326	IM+PG down vs IM
mmu-mir-6347	0.12575	-1.17326	IM+PG down vs IM
mmu-miR-6920-3p	0.12575	-1.17326	IM+PG down vs IM
mmu-miR-5621-5p	0.702858	-1.1732	IM+PG down vs IM
mmu-miR-7036-3p	0.367916	-1.1721	IM+PG down vs IM
mmu-miR-1247-3p	0.187015	-1.17154	IM+PG down vs IM
mmu-miR-7005-5p	0.110202	-1.17094	IM+PG down vs IM
mmu-miR-763	0.252742	-1.17092	IM+PG down vs IM
mmu-miR-7061-5p	0.438912	-1.17088	IM+PG down vs IM
mmu-miR-677-5p	0.438912	-1.17088	IM+PG down vs IM
mmu-miR-7042-3p	0.438912	-1.17088	IM+PG down vs IM
mmu-miR-124-3p	0.438912	-1.17088	IM+PG down vs IM
mmu-miR-1251-3p	0.110586	-1.17029	IM+PG down vs IM
mmu-miR-410-5p	0.110586	-1.17029	IM+PG down vs IM
mmu-miR-3075-3p	0.110586	-1.17029	IM+PG down vs IM
mmu-miR-106a-3p	0.666667	-1.17019	IM+PG down vs IM
mmu-miR-5627-5p	0.371755	-1.16968	IM+PG down vs IM
mmu-mir-653	0.412542	-1.16941	IM+PG down vs IM

mmu-miR-32-3p	0.245327	-1.16902	IM+PG down vs IM
mmu-miR-6997-3p	0.167889	-1.16742	IM+PG down vs IM
mmu-miR-7089-3p	0.0948465	-1.16734	IM+PG down vs IM
mmu-miR-802-3p	0.167195	-1.16728	IM+PG down vs IM
mmu-miR-295-5p	0.724426	-1.1672	IM+PG down vs IM
mmu-mir-5626	0	-1.16685	IM+PG down vs IM
mmu-miR-31-5p	0	-1.16685	IM+PG down vs IM
mmu-miR-297c-5p	0	-1.16685	IM+PG down vs IM
mmu-miR-1192	0	-1.16685	IM+PG down vs IM
mmu-miR-181c-3p	0	-1.16685	IM+PG down vs IM
mmu-miR-5107-5p	0	-1.16685	IM+PG down vs IM
mmu-miR-6909-3p	0	-1.16685	IM+PG down vs IM
mmu-miR-6981-5p	0	-1.16685	IM+PG down vs IM
mmu-miR-341-3p	0.586432	-1.16636	IM+PG down vs IM
mmu-miR-7230-5p	0.429516	-1.16603	IM+PG down vs IM
mmu-mir-7660	0.373722	-1.16562	IM+PG down vs IM
mmu-mir-30f	0.666667	-1.16502	IM+PG down vs IM
mmu-mir-874	0.666667	-1.16502	IM+PG down vs IM
mmu-miR-470-5p	0.0159952	-1.16428	IM+PG down vs IM
mmu-miR-873a-5p	0.666667	-1.16419	IM+PG down vs IM
mmu-miR-5129-5p	0.151533	-1.1641	IM+PG down vs IM
mmu-mir-5112	0	-1.16171	IM+PG down vs IM
mmu-mir-5616	0	-1.16171	IM+PG down vs IM
mmu-mir-6982	0	-1.16171	IM+PG down vs IM
mmu-mir-7212	0	-1.16171	IM+PG down vs IM
mmu-mir-7679	0	-1.16171	IM+PG down vs IM
mmu-miR-331-3p	0	-1.16171	IM+PG down vs IM
mmu-miR-200b-5p	0	-1.16171	IM+PG down vs IM
mmu-miR-1947-5p	0	-1.16171	IM+PG down vs IM
mmu-miR-1249-5p	0	-1.16171	IM+PG down vs IM
mmu-miR-3063-5p	0	-1.16171	IM+PG down vs IM
mmu-miR-3085-3p	0	-1.16171	IM+PG down vs IM
mmu-miR-6929-5p	0	-1.16171	IM+PG down vs IM
mmu-miR-6965-3p	0	-1.16171	IM+PG down vs IM
mmu-miR-7009-5p	0	-1.16171	IM+PG down vs IM
mmu-miR-7035-5p	0	-1.16171	IM+PG down vs IM
mmu-miR-7647-5p	0	-1.16171	IM+PG down vs IM
mmu-mir-133b	0.666667	-1.16091	IM+PG down vs IM
mmu-miR-7091-5p	0.134478	-1.16082	IM+PG down vs IM
mmu-mir-741	0.134478	-1.16082	IM+PG down vs IM
mmu-mir-5104	0.134478	-1.16082	IM+PG down vs IM
mmu-miR-3097-3p	0.134478	-1.16082	IM+PG down vs IM
mmu-miR-190b-5p	0.134478	-1.16082	IM+PG down vs IM

mmu-miR-6933-3p	0.134478	-1.16082	IM+PG down vs IM
mmu-mir-196b	0.207531	-1.1602	IM+PG down vs IM
mmu-miR-6393	0.193689	-1.15728	IM+PG down vs IM
mmu-mir-6929	0.415869	-1.15713	IM+PG down vs IM
mmu-mir-467a-3	0.415869	-1.15713	IM+PG down vs IM
mmu-mir-467a-6	0.415869	-1.15713	IM+PG down vs IM
mmu-mir-467a-10	0.415869	-1.15713	IM+PG down vs IM
mmu-mir-344g	0.0313984	-1.15658	IM+PG down vs IM
mmu-miR-449c-3p	0.727784	-1.15542	IM+PG down vs IM
mmu-miR-672-5p	0.180166	-1.15457	IM+PG down vs IM
mmu-miR-19a-5p	0.666667	-1.1543	IM+PG down vs IM
mmu-miR-107-5p	0.666667	-1.1543	IM+PG down vs IM
mmu-mir-7214	0.721597	-1.15324	IM+PG down vs IM
mmu-miR-7646-5p	0.400189	-1.15311	IM+PG down vs IM
mmu-mir-6338	0.400189	-1.15311	IM+PG down vs IM
mmu-miR-1963	0.400189	-1.15311	IM+PG down vs IM
mmu-mir-541	0.0021739	-1.15211	IM+PG down vs IM
mmu-mir-375	0.211109	-1.15192	IM+PG down vs IM
mmu-miR-741-3p	0	-1.15179	IM+PG down vs IM
mmu-miR-7039-3p	0	-1.15179	IM+PG down vs IM
mmu-miR-7058-5p	0	-1.15179	IM+PG down vs IM
mmu-mir-431	0	-1.15179	IM+PG down vs IM
mmu-mir-494	0	-1.15179	IM+PG down vs IM
mmu-mir-369	0	-1.15179	IM+PG down vs IM
mmu-mir-374b	0	-1.15179	IM+PG down vs IM
mmu-mir-667	0	-1.15179	IM+PG down vs IM
mmu-mir-670	0	-1.15179	IM+PG down vs IM
mmu-mir-466c-1	0	-1.15179	IM+PG down vs IM
mmu-mir-1901	0	-1.15179	IM+PG down vs IM
mmu-mir-669m-2	0	-1.15179	IM+PG down vs IM
mmu-mir-1951	0	-1.15179	IM+PG down vs IM
mmu-mir-3064	0	-1.15179	IM+PG down vs IM
mmu-mir-5621	0	-1.15179	IM+PG down vs IM
mmu-mir-5627	0	-1.15179	IM+PG down vs IM
mmu-mir-6386	0	-1.15179	IM+PG down vs IM
mmu-mir-6910	0	-1.15179	IM+PG down vs IM
mmu-mir-7649	0	-1.15179	IM+PG down vs IM
mmu-mir-126b	0	-1.15179	IM+PG down vs IM
mmu-mir-8108	0	-1.15179	IM+PG down vs IM
mmu-mir-8111	0	-1.15179	IM+PG down vs IM
mmu-mir-8111	0	-1.15179	IM+PG down vs IM
mmu-miR-129-5p	0	-1.15179	IM+PG down vs IM
mmu-miR-383-5p	0	-1.15179	IM+PG down vs IM

mmu-miR-410-3p	0	-1.15179	IM+PG down vs IM
mmu-miR-489-3p	0	-1.15179	IM+PG down vs IM
mmu-miR-547-3p	0	-1.15179	IM+PG down vs IM
mmu-miR-488-5p	0	-1.15179	IM+PG down vs IM
mmu-miR-186-3p	0	-1.15179	IM+PG down vs IM
mmu-let-7b-3p	0	-1.15179	IM+PG down vs IM
mmu-miR-871-5p	0	-1.15179	IM+PG down vs IM
mmu-miR-466f	0	-1.15179	IM+PG down vs IM
mmu-miR-1251-5p	0	-1.15179	IM+PG down vs IM
mmu-miR-3063-3p	0	-1.15179	IM+PG down vs IM
mmu-let-7e-3p	0	-1.15179	IM+PG down vs IM
mmu-miR-487b-5p	0	-1.15179	IM+PG down vs IM
mmu-miR-5136	0	-1.15179	IM+PG down vs IM
mmu-miR-6918-3p	0	-1.15179	IM+PG down vs IM
mmu-miR-6929-3p	0	-1.15179	IM+PG down vs IM
mmu-miR-6942-5p	0	-1.15179	IM+PG down vs IM
mmu-miR-7070-3p	0	-1.15179	IM+PG down vs IM
mmu-miR-7072-5p	0	-1.15179	IM+PG down vs IM
mmu-miR-6546-5p	0	-1.15179	IM+PG down vs IM
mmu-miR-7662-3p	0	-1.15179	IM+PG down vs IM
mmu-miR-7675-5p	0	-1.15179	IM+PG down vs IM
mmu-miR-8105	0	-1.15179	IM+PG down vs IM
mmu-mir-297a-1	0	-1.15179	IM+PG down vs IM
mmu-miR-3078-5p	0	-1.15149	IM+PG down vs IM
mmu-miR-7024-3p	0.162948	-1.15128	IM+PG down vs IM
mmu-mir-3087	0.162948	-1.15128	IM+PG down vs IM
mmu-miR-150-3p	0.666667	-1.15075	IM+PG down vs IM
mmu-miR-7222-3p	0	-1.14931	IM+PG down vs IM
mmu-miR-7669-5p	0	-1.14931	IM+PG down vs IM
mmu-mir-219b	0.144908	-1.14804	IM+PG down vs IM
mmu-let-7d-5p	0.115339	-1.14538	IM+PG down vs IM
mmu-miR-6402	0.666667	-1.14502	IM+PG down vs IM
mmu-miR-6389	0.666667	-1.14502	IM+PG down vs IM
mmu-miR-7006-5p	0.345693	-1.14477	IM+PG down vs IM
mmu-miR-7688-3p	0.416381	-1.14465	IM+PG down vs IM
mmu-miR-98-5p	0.123412	-1.1444	IM+PG down vs IM
mmu-mir-429	0.123412	-1.1444	IM+PG down vs IM
mmu-miR-134-3p	0.129065	-1.14366	IM+PG down vs IM
mmu-miR-6369	0.703125	-1.13974	IM+PG down vs IM
mmu-miR-770-3p	0.666667	-1.13967	IM+PG down vs IM
mmu-mir-542	0.568662	-1.13967	IM+PG down vs IM
mmu-mir-7648	0	-1.13959	IM+PG down vs IM
mmu-miR-6948-5p	0	-1.13959	IM+PG down vs IM

mmu-mir-194-1	0	-1.13959	IM+PG down vs IM
mmu-mir-194-1	0	-1.13959	IM+PG down vs IM
mmu-let-7b	0	-1.13959	IM+PG down vs IM
mmu-mir-7a-2	0	-1.13959	IM+PG down vs IM
mmu-mir-574	0	-1.13959	IM+PG down vs IM
mmu-mir-467e	0	-1.13959	IM+PG down vs IM
mmu-mir-1930	0	-1.13959	IM+PG down vs IM
mmu-mir-1957a	0	-1.13959	IM+PG down vs IM
mmu-mir-1961	0	-1.13959	IM+PG down vs IM
mmu-mir-2136	0	-1.13959	IM+PG down vs IM
mmu-mir-599	0	-1.13959	IM+PG down vs IM
mmu-mir-3062	0	-1.13959	IM+PG down vs IM
mmu-mir-466o	0	-1.13959	IM+PG down vs IM
mmu-mir-1231	0	-1.13959	IM+PG down vs IM
mmu-mir-3473e	0	-1.13959	IM+PG down vs IM
mmu-mir-7003	0	-1.13959	IM+PG down vs IM
mmu-mir-7005	0	-1.13959	IM+PG down vs IM
mmu-miR-295-3p	0	-1.13959	IM+PG down vs IM
mmu-miR-376a-3p	0	-1.13959	IM+PG down vs IM
mmu-miR-499-5p	0	-1.13959	IM+PG down vs IM
mmu-miR-30c-1-3p	0	-1.13959	IM+PG down vs IM
mmu-miR-29a-5p	0	-1.13959	IM+PG down vs IM
mmu-miR-879-5p	0	-1.13959	IM+PG down vs IM
mmu-miR-872-3p	0	-1.13959	IM+PG down vs IM
mmu-miR-1190	0	-1.13959	IM+PG down vs IM
mmu-miR-3060-3p	0	-1.13959	IM+PG down vs IM
mmu-miR-3064-5p	0	-1.13959	IM+PG down vs IM
mmu-miR-3065-5p	0	-1.13959	IM+PG down vs IM
mmu-miR-3087-3p	0	-1.13959	IM+PG down vs IM
mmu-miR-190a-3p	0	-1.13959	IM+PG down vs IM
mmu-miR-496a-5p	0	-1.13959	IM+PG down vs IM
mmu-miR-5114	0	-1.13959	IM+PG down vs IM
mmu-miR-6374	0	-1.13959	IM+PG down vs IM
mmu-miR-6916-5p	0	-1.13959	IM+PG down vs IM
mmu-miR-6930-5p	0	-1.13959	IM+PG down vs IM
mmu-miR-7007-3p	0	-1.13959	IM+PG down vs IM
mmu-miR-7068-5p	0	-1.13959	IM+PG down vs IM
mmu-miR-7091-3p	0	-1.13959	IM+PG down vs IM
mmu-miR-7651-3p	0	-1.13959	IM+PG down vs IM
mmu-miR-7036b-3p	0	-1.13959	IM+PG down vs IM
mmu-miR-126b-3p	0	-1.13959	IM+PG down vs IM
mmu-mir-467b	0.517809	-1.13957	IM+PG down vs IM
mmu-mir-382	0.721637	-1.13885	IM+PG down vs IM

mmu-mir-6948	0.0108232	-1.13814	IM+PG down vs IM
mmu-miR-6951-3p	0.666667	-1.13678	IM+PG down vs IM
mmu-miR-467e-3p	0.666667	-1.13678	IM+PG down vs IM
mmu-miR-7652-3p	0.666667	-1.13678	IM+PG down vs IM
mmu-let-7c-1	0.0690113	-1.13611	IM+PG down vs IM
mmu-miR-19a-3p	0.666667	-1.13605	IM+PG down vs IM
mmu-mir-7046	0.666667	-1.13605	IM+PG down vs IM
mmu-miR-7074-5p	0.666667	-1.13605	IM+PG down vs IM
mmu-miR-329-3p	0.666667	-1.13605	IM+PG down vs IM
mmu-miR-302a-3p	0.666667	-1.13605	IM+PG down vs IM
mmu-miR-7225-3p	0.666667	-1.13605	IM+PG down vs IM
mmu-mir-677	0.775898	-1.13529	IM+PG down vs IM
mmu-miR-497-3p	0.0470275	-1.1335	IM+PG down vs IM
mmu-mir-29b-2	0.0486917	-1.13329	IM+PG down vs IM
mmu-miR-764-3p	0.723443	-1.13329	IM+PG down vs IM
mmu-miR-5617-5p	0	-1.13317	IM+PG down vs IM
mmu-miR-7117-3p	0.728536	-1.13164	IM+PG down vs IM
mmu-mir-3098	0.728536	-1.13164	IM+PG down vs IM
mmu-miR-3098-3p	0.22653	-1.13135	IM+PG down vs IM
mmu-miR-3070b-3p	0.22653	-1.13135	IM+PG down vs IM
mmu-mir-582	0.628815	-1.13035	IM+PG down vs IM
mmu-miR-29a-3p	0.765315	-1.13027	IM+PG down vs IM
mmu-mir-30c-2	0.733318	-1.13007	IM+PG down vs IM
mmu-miR-8117	0.617055	-1.12971	IM+PG down vs IM
mmu-miR-3098-5p	0.0256658	-1.12885	IM+PG down vs IM
mmu-miR-5620-5p	0.0709558	-1.12879	IM+PG down vs IM
mmu-miR-7212-3p	0.756646	-1.12878	IM+PG down vs IM
mmu-miR-1897-5p	0.211633	-1.1287	IM+PG down vs IM
mmu-mir-5622	0	-1.12853	IM+PG down vs IM
mmu-miR-449b	0.725414	-1.12765	IM+PG down vs IM
mmu-miR-879-3p	0	-1.12704	IM+PG down vs IM
mmu-mir-185	0	-1.12704	IM+PG down vs IM
mmu-mir-370	0	-1.12704	IM+PG down vs IM
mmu-mir-7028	0	-1.12704	IM+PG down vs IM
mmu-miR-485-3p	0	-1.12704	IM+PG down vs IM
mmu-miR-682	0	-1.12704	IM+PG down vs IM
mmu-miR-1187	0	-1.12704	IM+PG down vs IM
mmu-miR-3073a-5p	0	-1.12704	IM+PG down vs IM
mmu-miR-3075-5p	0	-1.12704	IM+PG down vs IM
mmu-miR-7240-3p	0	-1.12704	IM+PG down vs IM
mmu-miR-7675-3p	0	-1.12704	IM+PG down vs IM
mmu-mir-7031	0.218101	-1.12433	IM+PG down vs IM
mmu-miR-5130	0	-1.12245	IM+PG down vs IM

mmu-miR-1941-3p	0.650857	-1.12238	IM+PG down vs IM
mmu-miR-687	0.172104	-1.12232	IM+PG down vs IM
mmu-mir-6391	0.551137	-1.12169	IM+PG down vs IM
mmu-miR-26a-1-3p	0	-1.12135	IM+PG down vs IM
mmu-mir-6943	0.740468	-1.12127	IM+PG down vs IM
mmu-mir-6979	0.818004	-1.12119	IM+PG down vs IM
mmu-mir-1247	0.173323	-1.1191	IM+PG down vs IM
mmu-miR-467f	0.568107	-1.11885	IM+PG down vs IM
mmu-miR-509-3p	0.666667	-1.11882	IM+PG down vs IM
mmu-miR-6977-3p	0.666667	-1.11882	IM+PG down vs IM
mmu-mir-675	0.733646	-1.11878	IM+PG down vs IM
mmu-mir-466b-8	0.226965	-1.11877	IM+PG down vs IM
mmu-miR-6955-5p	0.226965	-1.11877	IM+PG down vs IM
mmu-mir-6384	0.747357	-1.11838	IM+PG down vs IM
mmu-miR-7677-5p	0.177781	-1.11798	IM+PG down vs IM
mmu-miR-6954-5p	0.177781	-1.11798	IM+PG down vs IM
mmu-mir-6236	0.177781	-1.11798	IM+PG down vs IM
mmu-mir-6903	0.195152	-1.11681	IM+PG down vs IM
mmu-mir-294	0.195943	-1.11673	IM+PG down vs IM
mmu-miR-3069-5p	0.519208	-1.11637	IM+PG down vs IM
mmu-mir-7240	0.206965	-1.11559	IM+PG down vs IM
mmu-miR-6993-3p	0.206965	-1.11559	IM+PG down vs IM
mmu-mir-450b	0.206965	-1.11559	IM+PG down vs IM
mmu-mir-467a-1	0.206965	-1.11559	IM+PG down vs IM
mmu-miR-182-3p	0.206965	-1.11559	IM+PG down vs IM
mmu-mir-3069	0.583452	-1.11522	IM+PG down vs IM
mmu-miR-1957b	0.717906	-1.11464	IM+PG down vs IM
mmu-mir-3964	0.743762	-1.11436	IM+PG down vs IM
mmu-miR-216a-5p	0.740346	-1.11395	IM+PG down vs IM
mmu-miR-292b-5p	0	-1.11385	IM+PG down vs IM
mmu-miR-7223-5p	0.718433	-1.1132	IM+PG down vs IM
mmu-miR-669a-5p	0.23748	-1.11271	IM+PG down vs IM
mmu-miR-669p-5p	0.23748	-1.11271	IM+PG down vs IM
mmu-mir-217	0.185609	-1.11244	IM+PG down vs IM
mmu-mir-5108	0.185609	-1.11244	IM+PG down vs IM
mmu-mir-16-2	0.666667	-1.11206	IM+PG down vs IM
mmu-mir-7043	0.666667	-1.11206	IM+PG down vs IM
mmu-mir-1931	0.666667	-1.11206	IM+PG down vs IM
mmu-miR-6940-3p	0.821299	-1.11037	IM+PG down vs IM
mmu-mir-3108	0.216971	-1.10955	IM+PG down vs IM
mmu-miR-20b-5p	0.216971	-1.10955	IM+PG down vs IM
mmu-miR-7115-5p	0.175041	-1.10832	IM+PG down vs IM
mmu-miR-92b-5p	0.575036	-1.10795	IM+PG down vs IM

mmu-miR-7239-3p	0.194973	-1.10642	IM+PG down vs IM
mmu-mir-681	0.194973	-1.10642	IM+PG down vs IM
mmu-mir-7052	0.194973	-1.10642	IM+PG down vs IM
mmu-mir-669a-1	0.194973	-1.10642	IM+PG down vs IM
mmu-mir-669a-2	0.194973	-1.10642	IM+PG down vs IM
mmu-mir-466b-4	0.194973	-1.10642	IM+PG down vs IM
mmu-mir-466b-6	0.194973	-1.10642	IM+PG down vs IM
mmu-mir-411	0.194973	-1.10642	IM+PG down vs IM
mmu-mir-669a-4	0.194973	-1.10642	IM+PG down vs IM
mmu-mir-669a-5	0.194973	-1.10642	IM+PG down vs IM
mmu-mir-669a-6	0.194973	-1.10642	IM+PG down vs IM
mmu-mir-669a-7	0.194973	-1.10642	IM+PG down vs IM
mmu-mir-669a-8	0.194973	-1.10642	IM+PG down vs IM
mmu-mir-669a-9	0.194973	-1.10642	IM+PG down vs IM
mmu-mir-669a-10	0.194973	-1.10642	IM+PG down vs IM
mmu-mir-669a-11	0.194973	-1.10642	IM+PG down vs IM
mmu-mir-669a-12	0.194973	-1.10642	IM+PG down vs IM
mmu-mir-5615-2	0.194973	-1.10642	IM+PG down vs IM
mmu-mir-8105	0.194973	-1.10642	IM+PG down vs IM
mmu-miR-9-5p	0.194973	-1.10642	IM+PG down vs IM
mmu-miR-1961	0.194973	-1.10642	IM+PG down vs IM
mmu-miR-6966-3p	0.194973	-1.10642	IM+PG down vs IM
mmu-miR-6715-5p	0.194973	-1.10642	IM+PG down vs IM
mmu-mir-6911	0.529123	-1.10577	IM+PG down vs IM
mmu-miR-877-3p	0.204581	-1.10552	IM+PG down vs IM
mmu-miR-701-5p	0.490828	-1.10547	IM+PG down vs IM
mmu-mir-128-2	0.462819	-1.1043	IM+PG down vs IM
mmu-mir-3078	0.658941	-1.10429	IM+PG down vs IM
mmu-mir-7662	0	-1.10407	IM+PG down vs IM
mmu-miR-193b-5p	0	-1.10407	IM+PG down vs IM
mmu-miR-7056-3p	0	-1.10407	IM+PG down vs IM
mmu-mir-6355	0.117078	-1.10373	IM+PG down vs IM
mmu-miR-6397	0.17372	-1.10362	IM+PG down vs IM
mmu-miR-6976-5p	0.720711	-1.10297	IM+PG down vs IM
mmu-miR-103-3p	0.615755	-1.10239	IM+PG down vs IM
mmu-miR-7219-5p	0.175779	-1.10228	IM+PG down vs IM
mmu-mir-3102	0.746663	-1.10202	IM+PG down vs IM
mmu-miR-153-3p	0.736672	-1.10184	IM+PG down vs IM
mmu-mir-3093	0	-1.10178	IM+PG down vs IM
mmu-mir-5133	0	-1.10178	IM+PG down vs IM
mmu-miR-409-3p	0	-1.10178	IM+PG down vs IM
mmu-miR-448-5p	0.521764	-1.10156	IM+PG down vs IM
mmu-miR-3535	0.666667	-1.10129	IM+PG down vs IM

mmu-miR-6964-5p	0.666667	-1.10117	IM+PG down vs IM
mmu-miR-7674-3p	0.666667	-1.10117	IM+PG down vs IM
mmu-miR-669a-3-3p	0.666667	-1.10117	IM+PG down vs IM
mmu-miR-7670-3p	0.666667	-1.10117	IM+PG down vs IM
mmu-mir-188	0.666667	-1.10117	IM+PG down vs IM
mmu-miR-5113	0.840889	-1.10117	IM+PG down vs IM
mmu-miR-98-3p	0.449521	-1.09974	IM+PG down vs IM
mmu-miR-337-3p	0.867508	-1.0993	IM+PG down vs IM
mmu-miR-6538	0.00275828	-1.09872	IM+PG down vs IM
mmu-miR-367-3p	0.743769	-1.09863	IM+PG down vs IM
mmu-mir-295	0.743949	-1.09832	IM+PG down vs IM
mmu-miR-675-3p	0.481548	-1.09804	IM+PG down vs IM
mmu-mir-497	0	-1.09752	IM+PG down vs IM
mmu-mir-3063	0	-1.09752	IM+PG down vs IM
mmu-mir-466p	0	-1.09752	IM+PG down vs IM
mmu-mir-3969	0	-1.09752	IM+PG down vs IM
mmu-mir-7651	0	-1.09752	IM+PG down vs IM
mmu-miR-1a-3p	0	-1.09752	IM+PG down vs IM
mmu-miR-1193-5p	0	-1.09752	IM+PG down vs IM
mmu-miR-669e-3p	0	-1.09752	IM+PG down vs IM
mmu-miR-6984-3p	0	-1.09752	IM+PG down vs IM
mmu-miR-7216-5p	0	-1.09752	IM+PG down vs IM
mmu-miR-7094b-2-5p	0.894765	-1.09556	IM+PG down vs IM
mmu-miR-3067-5p	0.828337	-1.09518	IM+PG down vs IM
mmu-miR-467d-3p	0.813339	-1.09376	IM+PG down vs IM
mmu-miR-463-5p	0.856355	-1.09354	IM+PG down vs IM
mmu-mir-6382	0.50526	-1.09352	IM+PG down vs IM
mmu-mir-465a	0.50526	-1.09352	IM+PG down vs IM
mmu-miR-6396	0.50526	-1.09352	IM+PG down vs IM
mmu-mir-6342	0.617666	-1.09317	IM+PG down vs IM
mmu-mir-19b-1	0.756227	-1.09306	IM+PG down vs IM
mmu-miR-3475-5p	0.666667	-1.09286	IM+PG down vs IM
mmu-miR-292-3p	0.666667	-1.09286	IM+PG down vs IM
mmu-mir-328	0.666667	-1.09286	IM+PG down vs IM
mmu-miR-7025-3p	0.666667	-1.09286	IM+PG down vs IM
mmu-miR-3068-3p	0.666667	-1.09286	IM+PG down vs IM
mmu-mir-3474	0.458321	-1.09274	IM+PG down vs IM
mmu-mir-10a	0.769785	-1.09271	IM+PG down vs IM
mmu-mir-19a	0	-1.09209	IM+PG down vs IM
mmu-mir-695	0	-1.09209	IM+PG down vs IM
mmu-mir-208b	0	-1.09209	IM+PG down vs IM
mmu-mir-1971	0	-1.09209	IM+PG down vs IM
mmu-mir-6416	0	-1.09209	IM+PG down vs IM

mmu-mir-7228	0	-1.09209	IM+PG down vs IM
mmu-mir-7667	0	-1.09209	IM+PG down vs IM
mmu-miR-493-5p	0	-1.09209	IM+PG down vs IM
mmu-miR-6537-5p	0	-1.09209	IM+PG down vs IM
mmu-miR-6901-3p	0	-1.09209	IM+PG down vs IM
mmu-miR-7071-3p	0	-1.09209	IM+PG down vs IM
mmu-mir-7089	0.622799	-1.0915	IM+PG down vs IM
mmu-mir-322	0.666667	-1.09076	IM+PG down vs IM
mmu-miR-136-3p	0.666667	-1.09076	IM+PG down vs IM
mmu-miR-7000-5p	0.666667	-1.09076	IM+PG down vs IM
mmu-mir-130a	0.666667	-1.09076	IM+PG down vs IM
mmu-miR-7664-3p	0.666667	-1.09076	IM+PG down vs IM
mmu-mir-29b-2	0.459702	-1.09048	IM+PG down vs IM
mmu-mir-344d-1	0	-1.09014	IM+PG down vs IM
mmu-miR-199b-5p	0.707182	-1.0901	IM+PG down vs IM
mmu-mir-297a-3	0.585915	-1.08896	IM+PG down vs IM
mmu-mir-1894	0.744585	-1.08839	IM+PG down vs IM
mmu-mir-6516	0.5465	-1.08839	IM+PG down vs IM
mmu-miR-7671-3p	0.66886	-1.0879	IM+PG down vs IM
mmu-miR-1957a	0.489912	-1.08741	IM+PG down vs IM
mmu-miR-7658-3p	0.645107	-1.08688	IM+PG down vs IM
mmu-mir-669a-3	0	-1.08618	IM+PG down vs IM
mmu-mir-6406	0	-1.08618	IM+PG down vs IM
mmu-mir-7226	0	-1.08618	IM+PG down vs IM
mmu-miR-101a-3p	0	-1.08618	IM+PG down vs IM
mmu-miR-291a-3p	0	-1.08618	IM+PG down vs IM
mmu-miR-381-3p	0	-1.08618	IM+PG down vs IM
mmu-miR-1897-3p	0	-1.08618	IM+PG down vs IM
mmu-miR-3058-5p	0	-1.08618	IM+PG down vs IM
mmu-miR-3106-5p	0	-1.08618	IM+PG down vs IM
mmu-miR-3090-3p	0	-1.08618	IM+PG down vs IM
mmu-miR-194-1-3p	0	-1.08618	IM+PG down vs IM
mmu-miR-101b-5p	0	-1.08618	IM+PG down vs IM
mmu-miR-486-3p	0	-1.08618	IM+PG down vs IM
mmu-miR-208b-5p	0	-1.08618	IM+PG down vs IM
mmu-miR-6905-3p	0	-1.08618	IM+PG down vs IM
mmu-miR-6946-3p	0	-1.08618	IM+PG down vs IM
mmu-miR-7087-5p	0	-1.08618	IM+PG down vs IM
mmu-miR-7681-3p	0	-1.08618	IM+PG down vs IM
mmu-miR-7685-5p	0	-1.08618	IM+PG down vs IM
mmu-miR-8096	0	-1.08618	IM+PG down vs IM
mmu-miR-8097	0	-1.08618	IM+PG down vs IM
mmu-mir-532	0.649133	-1.08561	IM+PG down vs IM

mmu-miR-99b-5p	0.666667	-1.08555	IM+PG down vs IM
mmu-miR-7646-3p	0.666667	-1.08555	IM+PG down vs IM
mmu-miR-487b-3p	0.666667	-1.08555	IM+PG down vs IM
mmu-mir-6385	0.666667	-1.08555	IM+PG down vs IM
mmu-miR-542-5p	0.666667	-1.08555	IM+PG down vs IM
mmu-miR-10b-3p	0.666667	-1.08555	IM+PG down vs IM
mmu-miR-1258-5p	0.666667	-1.08555	IM+PG down vs IM
mmu-miR-299b-5p	0.666667	-1.08555	IM+PG down vs IM
mmu-miR-8113	0.666667	-1.08555	IM+PG down vs IM
mmu-mir-146a	0.666667	-1.08555	IM+PG down vs IM
mmu-mir-124-2	0.666667	-1.08555	IM+PG down vs IM
mmu-mir-7115	0.666667	-1.08555	IM+PG down vs IM
mmu-mir-1264	0.666667	-1.08555	IM+PG down vs IM
mmu-miR-467a-5p	0.666667	-1.08555	IM+PG down vs IM
mmu-miR-2183	0.666667	-1.08555	IM+PG down vs IM
mmu-miR-23a-5p	0.666667	-1.08555	IM+PG down vs IM
mmu-mir-5124b	0.666667	-1.08555	IM+PG down vs IM
mmu-miR-7650-5p	0.666667	-1.08555	IM+PG down vs IM
mmu-mir-292	0.666667	-1.08555	IM+PG down vs IM
mmu-miR-139-5p	0.666667	-1.08555	IM+PG down vs IM
mmu-miR-139-3p	0.666667	-1.08555	IM+PG down vs IM
mmu-miR-367-5p	0.666667	-1.08555	IM+PG down vs IM
mmu-mir-193b	0.666667	-1.08555	IM+PG down vs IM
mmu-mir-7684	0.42904	-1.08529	IM+PG down vs IM
mmu-mir-30a	0	-1.08486	IM+PG down vs IM
mmu-mir-698	0	-1.08486	IM+PG down vs IM
mmu-mir-92b	0	-1.08486	IM+PG down vs IM
mmu-mir-1954	0	-1.08486	IM+PG down vs IM
mmu-mir-1954	0	-1.08486	IM+PG down vs IM
mmu-miR-466a-5p	0	-1.08486	IM+PG down vs IM
mmu-miR-574-5p	0	-1.08486	IM+PG down vs IM
mmu-miR-6406	0	-1.08486	IM+PG down vs IM
mmu-miR-7672-5p	0	-1.08486	IM+PG down vs IM
mmu-mir-135b	0.514245	-1.08374	IM+PG down vs IM
mmu-miR-219a-1-3p	0.754273	-1.08288	IM+PG down vs IM
mmu-miR-1a-2-5p	0.857674	-1.08258	IM+PG down vs IM
mmu-mir-5119	0.840635	-1.08188	IM+PG down vs IM
mmu-miR-134-5p	0.471694	-1.0814	IM+PG down vs IM
mmu-mir-3107	0.760816	-1.08137	IM+PG down vs IM
mmu-miR-196b-5p	0.760816	-1.08137	IM+PG down vs IM
mmu-mir-668	0.547737	-1.08133	IM+PG down vs IM
mmu-miR-26a-5p	0.490268	-1.08087	IM+PG down vs IM
mmu-mir-1932	0.726773	-1.08022	IM+PG down vs IM

mmu-let-7a-1	0.666667	-1.08021	IM+PG down vs IM
mmu-miR-667-3p	0.666667	-1.08021	IM+PG down vs IM
mmu-mir-5106	0.666667	-1.08021	IM+PG down vs IM
mmu-miR-5135	0.666667	-1.08021	IM+PG down vs IM
mmu-miR-6953-5p	0.617931	-1.07967	IM+PG down vs IM
mmu-mir-142	0.751669	-1.07857	IM+PG down vs IM
mmu-miR-6769b-5p	0	-1.07821	IM+PG down vs IM
mmu-mir-6897	0.459656	-1.07801	IM+PG down vs IM
mmu-mir-467d	0.666667	-1.07798	IM+PG down vs IM
mmu-miR-7688-5p	0.666667	-1.07798	IM+PG down vs IM
mmu-miR-743a-5p	0.666667	-1.07798	IM+PG down vs IM
mmu-miR-6999-3p	0.666667	-1.07798	IM+PG down vs IM
mmu-miR-29b-2-5p	0.666667	-1.07798	IM+PG down vs IM
mmu-mir-122	0.666667	-1.07798	IM+PG down vs IM
mmu-miR-6938-5p	0.666667	-1.07798	IM+PG down vs IM
mmu-miR-1912-3p	0.666667	-1.07798	IM+PG down vs IM
mmu-mir-103-1	0.666667	-1.07798	IM+PG down vs IM
mmu-mir-7118	0.666667	-1.07798	IM+PG down vs IM
mmu-miR-6962-3p	0.666667	-1.07798	IM+PG down vs IM
mmu-miR-7079-5p	0.666667	-1.07798	IM+PG down vs IM
mmu-mir-467a-2	0.666667	-1.07798	IM+PG down vs IM
mmu-mir-467a-4	0.666667	-1.07798	IM+PG down vs IM
mmu-mir-467a-5	0.666667	-1.07798	IM+PG down vs IM
mmu-mir-467a-7	0.666667	-1.07798	IM+PG down vs IM
mmu-mir-467a-8	0.666667	-1.07798	IM+PG down vs IM
mmu-mir-467a-9	0.666667	-1.07798	IM+PG down vs IM
mmu-miR-881-5p	0.666667	-1.07798	IM+PG down vs IM
mmu-miR-143-5p	0.666667	-1.07798	IM+PG down vs IM
mmu-miR-6925-3p	0.666667	-1.07798	IM+PG down vs IM
mmu-mir-201	0.666667	-1.07798	IM+PG down vs IM
mmu-mir-3099	0.666667	-1.07798	IM+PG down vs IM
mmu-miR-3057-3p	0.666667	-1.07798	IM+PG down vs IM
mmu-miR-365-1-5p	0.666667	-1.07798	IM+PG down vs IM
mmu-mir-466f-3	0.666667	-1.07798	IM+PG down vs IM
mmu-miR-6373	0.666667	-1.07798	IM+PG down vs IM
mmu-mir-376c	0.666667	-1.07798	IM+PG down vs IM
mmu-mir-1948	0.666667	-1.07798	IM+PG down vs IM
mmu-miR-92b-3p	0.666667	-1.07783	IM+PG down vs IM
mmu-mir-7032	0.666667	-1.07783	IM+PG down vs IM
mmu-mir-7055	0.666667	-1.07783	IM+PG down vs IM
mmu-mir-7078	0.666667	-1.07783	IM+PG down vs IM
mmu-mir-3960	0.666667	-1.07783	IM+PG down vs IM
mmu-mir-466f-4	0	-1.0772	IM+PG down vs IM

mmu-miR-7002-3p	0.588304	-1.07685	IM+PG down vs IM
mmu-miR-6987-5p	0.765003	-1.07646	IM+PG down vs IM
mmu-let-7f-5p	0.800133	-1.07629	IM+PG down vs IM
mmu-miR-6358	0.647029	-1.07613	IM+PG down vs IM
mmu-mir-1942	0.737951	-1.07559	IM+PG down vs IM
mmu-mir-216a	0.737951	-1.07559	IM+PG down vs IM
mmu-miR-669f-5p	0.760371	-1.07542	IM+PG down vs IM
mmu-miR-743b-3p	0.661117	-1.07398	IM+PG down vs IM
mmu-miR-7031-3p	0.245875	-1.07395	IM+PG down vs IM
mmu-miR-290b-3p	0.666667	-1.07352	IM+PG down vs IM
mmu-miR-468-5p	0.666667	-1.07352	IM+PG down vs IM
mmu-miR-6339	0.666667	-1.07352	IM+PG down vs IM
mmu-mir-6414	0.666667	-1.07352	IM+PG down vs IM
mmu-miR-3100-5p	0.666667	-1.07352	IM+PG down vs IM
mmu-mir-1251	0.666667	-1.07352	IM+PG down vs IM
mmu-mir-3070a	0.666667	-1.07352	IM+PG down vs IM
mmu-mir-3070b	0.666667	-1.07352	IM+PG down vs IM
mmu-miR-6401	0.666667	-1.07352	IM+PG down vs IM
mmu-mir-7067	0.666667	-1.07352	IM+PG down vs IM
mmu-mir-6398	0.666667	-1.07352	IM+PG down vs IM
mmu-mir-497b	0.666667	-1.07352	IM+PG down vs IM
mmu-miR-7653-3p	0.666667	-1.07322	IM+PG down vs IM
mmu-miR-126b-5p	0.666667	-1.07322	IM+PG down vs IM
mmu-mir-3473f	0.666667	-1.07322	IM+PG down vs IM
mmu-miR-130b-5p	0.666667	-1.07322	IM+PG down vs IM
mmu-mir-1906-1	0.767983	-1.07322	IM+PG down vs IM
mmu-mir-1906-2	0.767983	-1.07322	IM+PG down vs IM
mmu-miR-3076-5p	0.666667	-1.07322	IM+PG down vs IM
mmu-miR-18b-3p	0.666667	-1.07322	IM+PG down vs IM
mmu-mir-742	0.666667	-1.07322	IM+PG down vs IM
mmu-miR-669d-2-3p	0.666667	-1.07322	IM+PG down vs IM
mmu-miR-101a-5p	0.666667	-1.07322	IM+PG down vs IM
mmu-mir-338	0.666667	-1.07322	IM+PG down vs IM
mmu-miR-466c-5p	0.666667	-1.07322	IM+PG down vs IM
mmu-miR-1195	0.666667	-1.07322	IM+PG down vs IM
mmu-mir-5126	0.835261	-1.07254	IM+PG down vs IM
mmu-mir-592	0.627067	-1.07237	IM+PG down vs IM
mmu-miR-350-5p	0.627067	-1.07237	IM+PG down vs IM
mmu-miR-3960	0.158107	-1.07122	IM+PG down vs IM
mmu-miR-7055-5p	0.666667	-1.0708	IM+PG down vs IM
mmu-miR-3095-5p	0.666667	-1.0708	IM+PG down vs IM
mmu-miR-146b-3p	0.886071	-1.06929	IM+PG down vs IM
mmu-miR-6907-5p	0.759932	-1.06908	IM+PG down vs IM

mmu-mir-300	0.759932	-1.06908	IM+PG down vs IM
mmu-miR-7656-3p	0.700112	-1.06848	IM+PG down vs IM
mmu-miR-380-3p	0.777323	-1.06841	IM+PG down vs IM
mmu-miR-615-3p	0.760969	-1.06801	IM+PG down vs IM
mmu-mir-7666	0.666667	-1.06765	IM+PG down vs IM
mmu-miR-7653-5p	0.666667	-1.06751	IM+PG down vs IM
mmu-miR-141-3p	0.666667	-1.06751	IM+PG down vs IM
mmu-miR-377-5p	0.666667	-1.06751	IM+PG down vs IM
mmu-miR-92a-2-5p	0.666667	-1.06751	IM+PG down vs IM
mmu-miR-6986-3p	0.666667	-1.06751	IM+PG down vs IM
mmu-miR-7680-3p	0.666667	-1.06751	IM+PG down vs IM
mmu-mir-7011	0.666667	-1.06751	IM+PG down vs IM
mmu-miR-300-3p	0.666667	-1.06751	IM+PG down vs IM
mmu-miR-669h-3p	0.666667	-1.06751	IM+PG down vs IM
mmu-miR-3112-5p	0.666667	-1.06751	IM+PG down vs IM
mmu-mir-5113	0.666667	-1.06751	IM+PG down vs IM
mmu-miR-145a-3p	0.666667	-1.06751	IM+PG down vs IM
mmu-miR-485-5p	0.666667	-1.06751	IM+PG down vs IM
mmu-miR-92a-1-5p	0.666667	-1.06751	IM+PG down vs IM
mmu-miR-7063-5p	0.666667	-1.06751	IM+PG down vs IM
mmu-mir-376c	0.666667	-1.06751	IM+PG down vs IM
mmu-mir-1952	0.666667	-1.06751	IM+PG down vs IM
mmu-miR-6949-5p	0.666667	-1.06751	IM+PG down vs IM
mmu-miR-6340	0.666667	-1.06751	IM+PG down vs IM
mmu-mir-1956	0.666667	-1.06751	IM+PG down vs IM
mmu-miR-465a-3p	0.666667	-1.06751	IM+PG down vs IM
mmu-miR-465b-3p	0.666667	-1.06751	IM+PG down vs IM
mmu-miR-465c-3p	0.666667	-1.06751	IM+PG down vs IM
mmu-miR-205-5p	0.666667	-1.06751	IM+PG down vs IM
mmu-miR-6942-3p	0.666667	-1.06751	IM+PG down vs IM
mmu-mir-6928	0.666667	-1.06751	IM+PG down vs IM
mmu-mir-1946b	0.666667	-1.06751	IM+PG down vs IM
mmu-miR-7661-5p	0.666667	-1.06751	IM+PG down vs IM
mmu-miR-1258-3p	0.666667	-1.06751	IM+PG down vs IM
mmu-mir-7035	0.666667	-1.06751	IM+PG down vs IM
mmu-miR-7672-3p	0.666667	-1.06751	IM+PG down vs IM
mmu-mir-30e	0.666667	-1.06751	IM+PG down vs IM
mmu-mir-30e	0.666667	-1.06751	IM+PG down vs IM
mmu-mir-291b	0.666667	-1.06751	IM+PG down vs IM
mmu-mir-7678	0.666667	-1.06751	IM+PG down vs IM
mmu-miR-29c-3p	0.666667	-1.06751	IM+PG down vs IM
mmu-miR-7011-3p	0.666667	-1.06751	IM+PG down vs IM
mmu-miR-7067-5p	0.666667	-1.06751	IM+PG down vs IM

mmu-mir-491	0.711125	-1.06685	IM+PG down vs IM
mmu-miR-28a-5p	0.86308	-1.06679	IM+PG down vs IM
mmu-miR-3112-3p	0.666667	-1.06661	IM+PG down vs IM
mmu-miR-6899-3p	0.666667	-1.06661	IM+PG down vs IM
mmu-miR-873b	0.666667	-1.06661	IM+PG down vs IM
mmu-mir-7016	0.775308	-1.06603	IM+PG down vs IM
mmu-miR-6989-5p	0.63662	-1.065	IM+PG down vs IM
mmu-miR-3620-3p	0.301008	-1.06451	IM+PG down vs IM
mmu-mir-24-2	0.690241	-1.06412	IM+PG down vs IM
mmu-mir-873b	0.316691	-1.06397	IM+PG down vs IM
mmu-miR-3070a-3p	0.817106	-1.06244	IM+PG down vs IM
mmu-mir-6541	0.773622	-1.06226	IM+PG down vs IM
mmu-mir-760	0.708772	-1.06198	IM+PG down vs IM
mmu-miR-6900-3p	0.710785	-1.06171	IM+PG down vs IM
mmu-miR-491-5p	0.666667	-1.06162	IM+PG down vs IM
mmu-miR-488-3p	0.666667	-1.06162	IM+PG down vs IM
mmu-miR-704	0.666667	-1.06162	IM+PG down vs IM
mmu-mir-22	0.666667	-1.06162	IM+PG down vs IM
mmu-mir-134	0.666667	-1.06162	IM+PG down vs IM
mmu-mir-7a-1	0.666667	-1.06162	IM+PG down vs IM
mmu-mir-7646	0.666667	-1.06162	IM+PG down vs IM
mmu-mir-6537	0.666667	-1.06162	IM+PG down vs IM
mmu-miR-7035-3p	0.666667	-1.06162	IM+PG down vs IM
mmu-miR-489-5p	0.666667	-1.06162	IM+PG down vs IM
mmu-miR-6936-5p	0.666667	-1.06162	IM+PG down vs IM
mmu-miR-219b-3p	0.666667	-1.06162	IM+PG down vs IM
mmu-miR-3066-3p	0.666667	-1.06162	IM+PG down vs IM
mmu-miR-7039-5p	0.666667	-1.06162	IM+PG down vs IM
mmu-mir-1843b	0.666667	-1.06162	IM+PG down vs IM
mmu-mir-1958	0.666667	-1.06162	IM+PG down vs IM
mmu-mir-196a-2	0.666667	-1.06162	IM+PG down vs IM
mmu-mir-6909	0.666667	-1.06162	IM+PG down vs IM
mmu-miR-7053-3p	0.666667	-1.06162	IM+PG down vs IM
mmu-miR-679-3p	0.666667	-1.06162	IM+PG down vs IM
mmu-mir-7056	0.666667	-1.06162	IM+PG down vs IM
mmu-miR-669i	0.666667	-1.06162	IM+PG down vs IM
mmu-let-7a-2-3p	0.666667	-1.06162	IM+PG down vs IM
mmu-miR-5099	0.666667	-1.06162	IM+PG down vs IM
mmu-mir-6359	0.666667	-1.06162	IM+PG down vs IM
mmu-miR-6902-5p	0.666667	-1.06162	IM+PG down vs IM
mmu-mir-7117	0.666667	-1.06162	IM+PG down vs IM
mmu-miR-6898-3p	0.666667	-1.06162	IM+PG down vs IM
mmu-mir-30a	0.666667	-1.06162	IM+PG down vs IM

mmu-miR-6910-3p	0.666667	-1.06162	IM+PG down vs IM
mmu-mir-128-2	0.666667	-1.06162	IM+PG down vs IM
mmu-mir-6346	0.666667	-1.06162	IM+PG down vs IM
mmu-miR-3108-5p	0.666667	-1.06162	IM+PG down vs IM
mmu-miR-7086-5p	0.666667	-1.06162	IM+PG down vs IM
mmu-mir-7017	0.666667	-1.06162	IM+PG down vs IM
mmu-miR-7042-5p	0.666667	-1.06162	IM+PG down vs IM
mmu-miR-6933-5p	0.767695	-1.06162	IM+PG down vs IM
mmu-miR-676-3p	0.767695	-1.06162	IM+PG down vs IM
mmu-miR-199a-5p	0.748672	-1.06103	IM+PG down vs IM
mmu-mir-374c	0.748672	-1.06103	IM+PG down vs IM
mmu-miR-453	0.367602	-1.06098	IM+PG down vs IM
mmu-miR-187-3p	0.729787	-1.06053	IM+PG down vs IM
mmu-miR-8106	0.776221	-1.06007	IM+PG down vs IM
mmu-miR-294-5p	0.784146	-1.0586	IM+PG down vs IM
mmu-miR-3079-3p	0.784146	-1.0586	IM+PG down vs IM
mmu-miR-1954	0.666667	-1.05829	IM+PG down vs IM
mmu-mir-3961	0.666667	-1.05829	IM+PG down vs IM
mmu-mir-6966	0.666667	-1.05829	IM+PG down vs IM
mmu-miR-455-5p	0.666667	-1.05829	IM+PG down vs IM
mmu-miR-6367	0.666667	-1.05829	IM+PG down vs IM
mmu-mir-139	0.666667	-1.05829	IM+PG down vs IM
mmu-miR-7649-5p	0.766949	-1.05801	IM+PG down vs IM
mmu-mir-703	0.790736	-1.05725	IM+PG down vs IM
mmu-mir-344d-3	0.790736	-1.05725	IM+PG down vs IM
mmu-mir-200c	0.742879	-1.05715	IM+PG down vs IM
mmu-miR-467c-5p	0.780272	-1.05686	IM+PG down vs IM
mmu-mir-7237	0.828266	-1.05654	IM+PG down vs IM
mmu-mir-449a	0.745708	-1.05643	IM+PG down vs IM
mmu-mir-24-2	0.745708	-1.05643	IM+PG down vs IM
mmu-miR-302d-3p	0.745708	-1.05643	IM+PG down vs IM
mmu-miR-7670-5p	0.782224	-1.05539	IM+PG down vs IM
mmu-miR-211-5p	0.782224	-1.05539	IM+PG down vs IM
mmu-miR-6347	0.924226	-1.05537	IM+PG down vs IM
mmu-miR-1291	0.757028	-1.05504	IM+PG down vs IM
mmu-miR-532-3p	0.803065	-1.05486	IM+PG down vs IM
mmu-mir-155	0.805473	-1.05449	IM+PG down vs IM
mmu-mir-6970	0.790507	-1.05393	IM+PG down vs IM
mmu-mir-1190	0.790507	-1.05393	IM+PG down vs IM
mmu-mir-6925	0.790507	-1.05393	IM+PG down vs IM
mmu-miR-29c-5p	0.790507	-1.05393	IM+PG down vs IM
mmu-miR-7018-3p	0.790507	-1.05393	IM+PG down vs IM
mmu-miR-804	0.790507	-1.05393	IM+PG down vs IM

mmu-miR-346-5p	0.666667	-1.05388	IM+PG down vs IM
mmu-miR-696	0.666667	-1.05388	IM+PG down vs IM
mmu-miR-7064-3p	0.666667	-1.05388	IM+PG down vs IM
mmu-miR-3474	0.666667	-1.05388	IM+PG down vs IM
mmu-mir-145a	0.666667	-1.05388	IM+PG down vs IM
mmu-miR-3107-3p	0.666667	-1.05388	IM+PG down vs IM
mmu-miR-429-5p	0.666667	-1.05075	IM+PG down vs IM
mmu-miR-6537-3p	0.666667	-1.05061	IM+PG down vs IM
mmu-miR-1198-5p	0.892047	-1.05044	IM+PG down vs IM
mmu-miR-653-3p	0.782236	-1.05043	IM+PG down vs IM
mmu-miR-6983-3p	0.783047	-1.04989	IM+PG down vs IM
mmu-miR-6968-3p	0.790374	-1.04978	IM+PG down vs IM
mmu-miR-452-3p	0.790374	-1.04978	IM+PG down vs IM
mmu-miR-6993-5p	0.790374	-1.04978	IM+PG down vs IM
mmu-miR-6934-5p	0.790374	-1.04978	IM+PG down vs IM
mmu-mir-374c	0.790374	-1.04978	IM+PG down vs IM
mmu-mir-297a-4	0.790374	-1.04978	IM+PG down vs IM
mmu-mir-181c	0.790374	-1.04978	IM+PG down vs IM
mmu-miR-541-3p	0.666667	-1.04975	IM+PG down vs IM
mmu-mir-3076	0.666667	-1.04975	IM+PG down vs IM
mmu-miR-7013-5p	0	-1.04945	IM+PG down vs IM
mmu-miR-7b-5p	0.901894	-1.04876	IM+PG down vs IM
mmu-miR-6992-3p	0	-1.04869	IM+PG down vs IM
mmu-miR-7076-5p	0.905248	-1.04868	IM+PG down vs IM
mmu-mir-496a	0.799128	-1.04833	IM+PG down vs IM
mmu-mir-488	0.799128	-1.04833	IM+PG down vs IM
mmu-mir-489	0.799128	-1.04833	IM+PG down vs IM
mmu-mir-205	0.799128	-1.04833	IM+PG down vs IM
mmu-miR-669j	0.666667	-1.0483	IM+PG down vs IM
mmu-miR-8091	0.821159	-1.04822	IM+PG down vs IM
mmu-mir-6368	0.883235	-1.04798	IM+PG down vs IM
mmu-mir-493	0.786493	-1.04768	IM+PG down vs IM
mmu-mir-1907	0.801572	-1.04687	IM+PG down vs IM
mmu-miR-7660-3p	0.844704	-1.04685	IM+PG down vs IM
mmu-miR-463-3p	0.0688179	-1.04646	IM+PG down vs IM
mmu-mir-467h	0.901476	-1.04605	IM+PG down vs IM
mmu-miR-7021-5p	0.76936	-1.04593	IM+PG down vs IM
mmu-mir-299b	0.76936	-1.04593	IM+PG down vs IM
mmu-miR-7026-5p	0.769361	-1.04593	IM+PG down vs IM
mmu-miR-6418-3p	0.769361	-1.04593	IM+PG down vs IM
mmu-mir-365-2	0.769361	-1.04593	IM+PG down vs IM
mmu-mir-490	0.790078	-1.04551	IM+PG down vs IM
mmu-miR-128-1-5p	0.790078	-1.04551	IM+PG down vs IM

mmu-mir-302c	0.790079	-1.04551	IM+PG down vs IM
mmu-miR-688	0.790079	-1.04551	IM+PG down vs IM
mmu-miR-7061-3p	0.790079	-1.04551	IM+PG down vs IM
mmu-miR-412-3p	0.790079	-1.04551	IM+PG down vs IM
mmu-mir-1843b	0.790079	-1.04551	IM+PG down vs IM
mmu-miR-3082-3p	0.790079	-1.04551	IM+PG down vs IM
mmu-miR-218-5p	0.790325	-1.04537	IM+PG down vs IM
mmu-miR-3102-3p.2-3p	0.932094	-1.04491	IM+PG down vs IM
mmu-miR-3071-3p	0.700776	-1.04452	IM+PG down vs IM
mmu-mir-669o	0.800139	-1.04399	IM+PG down vs IM
mmu-mir-19b-1	0.800139	-1.04399	IM+PG down vs IM
mmu-mir-669p-1	0.800139	-1.04399	IM+PG down vs IM
mmu-mir-669p-2	0.800139	-1.04399	IM+PG down vs IM
mmu-mir-6420	0.144219	-1.04351	IM+PG down vs IM
mmu-miR-7667-3p	0.829786	-1.04303	IM+PG down vs IM
mmu-miR-1264-3p	0.758589	-1.04286	IM+PG down vs IM
mmu-miR-494-5p	0.830802	-1.04276	IM+PG down vs IM
mmu-miR-7674-5p	0.802977	-1.04246	IM+PG down vs IM
mmu-mir-873a	0.802977	-1.04246	IM+PG down vs IM
mmu-mir-6983	0.802977	-1.04246	IM+PG down vs IM
mmu-miR-6954-3p	0.911216	-1.04242	IM+PG down vs IM
mmu-miR-30d-3p	0.795942	-1.04224	IM+PG down vs IM
mmu-miR-505-5p	0.666667	-1.04157	IM+PG down vs IM
mmu-mir-717	0.776579	-1.04157	IM+PG down vs IM
mmu-miR-7054-3p	0.666667	-1.04157	IM+PG down vs IM
mmu-miR-466d-5p	0.666667	-1.04157	IM+PG down vs IM
mmu-miR-7080-3p	0.666667	-1.04157	IM+PG down vs IM
mmu-miR-2136	0.666667	-1.04157	IM+PG down vs IM
mmu-mir-207	0.666667	-1.04157	IM+PG down vs IM
mmu-miR-7043-3p	0.666667	-1.04157	IM+PG down vs IM
mmu-mir-7685	0.816897	-1.04135	IM+PG down vs IM
mmu-miR-7051-5p	0.852287	-1.04128	IM+PG down vs IM
mmu-miR-7066-3p	0.944162	-1.0412	IM+PG down vs IM
mmu-mir-7243	0.806381	-1.04071	IM+PG down vs IM
mmu-miR-496b	0.0779119	-1.04055	IM+PG down vs IM
mmu-miR-344c-3p	0.794624	-1.0404	IM+PG down vs IM
mmu-miR-7004-3p	0.821012	-1.03935	IM+PG down vs IM
mmu-miR-5134-3p	0.835778	-1.03822	IM+PG down vs IM
mmu-miR-7240-5p	0.863282	-1.03789	IM+PG down vs IM
mmu-mir-183	0	-1.03762	IM+PG down vs IM
mmu-mir-7689	0	-1.03762	IM+PG down vs IM
mmu-miR-370-5p	0	-1.03762	IM+PG down vs IM
mmu-miR-6900-5p	0	-1.03762	IM+PG down vs IM

mmu-miR-6958-3p	0	-1.03762	IM+PG down vs IM
mmu-mir-1981	0.826053	-1.03704	IM+PG down vs IM
mmu-mir-1905	0.785408	-1.03696	IM+PG down vs IM
mmu-mir-6938	0.785408	-1.03696	IM+PG down vs IM
mmu-miR-3104-3p	0.815645	-1.03637	IM+PG down vs IM
mmu-miR-210-5p	0.825641	-1.03498	IM+PG down vs IM
mmu-mir-7001	0.859966	-1.03395	IM+PG down vs IM
mmu-mir-6997	0.825425	-1.03234	IM+PG down vs IM
mmu-miR-709	0.920714	-1.0315	IM+PG down vs IM
mmu-miR-3091-3p	0.874198	-1.03127	IM+PG down vs IM
mmu-mir-7242	0.831108	-1.03023	IM+PG down vs IM
mmu-mir-344d-2	0.871794	-1.03002	IM+PG down vs IM
mmu-mir-344d-2	0.871794	-1.03002	IM+PG down vs IM
mmu-miR-3569-3p	0.803527	-1.0294	IM+PG down vs IM
mmu-miR-7094-1-5p	0.841962	-1.0288	IM+PG down vs IM
mmu-miR-7119-5p	0.841962	-1.0288	IM+PG down vs IM
mmu-miR-466k	0.949795	-1.02856	IM+PG down vs IM
mmu-miR-7236-3p	0.838527	-1.02767	IM+PG down vs IM
mmu-mir-6994	0.838527	-1.02767	IM+PG down vs IM
mmu-miR-3062-3p	0.838527	-1.02767	IM+PG down vs IM
mmu-miR-698-3p	0.899337	-1.02698	IM+PG down vs IM
mmu-mir-547	0.666667	-1.02683	IM+PG down vs IM
mmu-mir-106b	0.666667	-1.02683	IM+PG down vs IM
mmu-mir-505	0.666667	-1.02683	IM+PG down vs IM
mmu-let-7f-2	0.666667	-1.02683	IM+PG down vs IM
mmu-miR-6403	0.841707	-1.02665	IM+PG down vs IM
mmu-miR-450b-5p	0.834744	-1.02639	IM+PG down vs IM
mmu-miR-26b-3p	0.834744	-1.02639	IM+PG down vs IM
mmu-mir-181b-2	0.864741	-1.02564	IM+PG down vs IM
mmu-miR-1b-5p	0.960865	-1.02551	IM+PG down vs IM
mmu-miR-1892	0.593657	-1.02508	IM+PG down vs IM
mmu-miR-449a-5p	0.847425	-1.02489	IM+PG down vs IM
mmu-mir-297a-2	0.666667	-1.02442	IM+PG down vs IM
mmu-mir-344b	0.666667	-1.02442	IM+PG down vs IM
mmu-mir-6397	0.666667	-1.02442	IM+PG down vs IM
mmu-miR-6411	0.666667	-1.02442	IM+PG down vs IM
mmu-mir-219c	0.666667	-1.02442	IM+PG down vs IM
mmu-mir-6986	0.666667	-1.02442	IM+PG down vs IM
mmu-mir-455	0.666667	-1.02442	IM+PG down vs IM
mmu-miR-7022-3p	0.666667	-1.02442	IM+PG down vs IM
mmu-miR-206-3p	0.666667	-1.02442	IM+PG down vs IM
mmu-miR-294-3p	0.666667	-1.02442	IM+PG down vs IM
mmu-mir-410	0.666667	-1.02442	IM+PG down vs IM

mmu-mir-3547	0.666667	-1.02442	IM+PG down vs IM
mmu-mir-6980	0.666667	-1.02442	IM+PG down vs IM
mmu-miR-125a-3p	0.666667	-1.02442	IM+PG down vs IM
mmu-let-7j	0.666667	-1.02442	IM+PG down vs IM
mmu-miR-7050-3p	0.84954	-1.02427	IM+PG down vs IM
mmu-mir-3080	0.868736	-1.0237	IM+PG down vs IM
mmu-miR-6955-3p	0.858997	-1.02347	IM+PG down vs IM
mmu-mir-144	0.8612	-1.02285	IM+PG down vs IM
mmu-miR-6343	0.913655	-1.02256	IM+PG down vs IM
mmu-miR-6998-3p	0.849478	-1.02214	IM+PG down vs IM
mmu-miR-199a-3p	0.869637	-1.02211	IM+PG down vs IM
mmu-miR-199b-3p	0.869637	-1.02211	IM+PG down vs IM
mmu-miR-466p-5p	0.913006	-1.02204	IM+PG down vs IM
mmu-mir-710	0.903761	-1.02189	IM+PG down vs IM
mmu-miR-3102-3p	0.866057	-1.02152	IM+PG down vs IM
mmu-mir-1a-2	0.666667	-1.02151	IM+PG down vs IM
mmu-miR-6353	0.666667	-1.02151	IM+PG down vs IM
mmu-mir-3473d	0.666667	-1.02151	IM+PG down vs IM
mmu-mir-3104	0.666667	-1.02151	IM+PG down vs IM
mmu-miR-3085-5p	0.666667	-1.02151	IM+PG down vs IM
mmu-let-7f-1	0.666667	-1.02151	IM+PG down vs IM
mmu-miR-6973a-3p	0.666667	-1.02151	IM+PG down vs IM
mmu-mir-6546	0.666667	-1.02151	IM+PG down vs IM
mmu-mir-7092	0.666667	-1.02151	IM+PG down vs IM
mmu-mir-6417	0.666667	-1.02151	IM+PG down vs IM
mmu-miR-5619-3p	0.666667	-1.02151	IM+PG down vs IM
mmu-miR-322-3p	0.666667	-1.02151	IM+PG down vs IM
mmu-miR-499-3p	0.945378	-1.02151	IM+PG down vs IM
mmu-miR-320-5p	0.666667	-1.02151	IM+PG down vs IM
mmu-mir-100	0.666667	-1.02151	IM+PG down vs IM
mmu-mir-1941	0.666667	-1.02151	IM+PG down vs IM
mmu-mir-28c	0.666667	-1.02151	IM+PG down vs IM
mmu-mir-6914	0.666667	-1.02151	IM+PG down vs IM
mmu-mir-6960	0.666667	-1.02151	IM+PG down vs IM
mmu-miR-26b-5p	0.666667	-1.02151	IM+PG down vs IM
mmu-miR-30b-3p	0.666667	-1.02151	IM+PG down vs IM
mmu-miR-1843b-5p	0.666667	-1.02151	IM+PG down vs IM
mmu-miR-5125	0.666667	-1.02151	IM+PG down vs IM
mmu-miR-6924-5p	0.666667	-1.02151	IM+PG down vs IM
mmu-miR-7211-3p	0.666667	-1.02151	IM+PG down vs IM
mmu-miR-216c-3p	0.666667	-1.02151	IM+PG down vs IM
mmu-mir-3071	0.666667	-1.02151	IM+PG down vs IM
mmu-miR-421-3p	0.9756	-1.02151	IM+PG down vs IM

mmu-miR-7066-5p	0.876691	-1.02087	IM+PG down vs IM
mmu-miR-3071-5p	0.781348	-1.02046	IM+PG down vs IM
mmu-miR-7213-5p	0.936204	-1.02032	IM+PG down vs IM
mmu-miR-344g-3p	0.868969	-1.01916	IM+PG down vs IM
mmu-mir-1936	0.618391	-1.01903	IM+PG down vs IM
mmu-miR-7051-3p	0.666667	-1.01863	IM+PG down vs IM
mmu-miR-1906	0.666667	-1.01863	IM+PG down vs IM
mmu-miR-7228-3p	0.666667	-1.01863	IM+PG down vs IM
mmu-miR-7084-3p	0.666667	-1.01863	IM+PG down vs IM
mmu-mir-337	0.666667	-1.01863	IM+PG down vs IM
mmu-mir-6919	0.666667	-1.01863	IM+PG down vs IM
mmu-miR-6897-3p	0.666667	-1.01863	IM+PG down vs IM
mmu-mir-598	0.666667	-1.01863	IM+PG down vs IM
mmu-miR-543-3p	0.666667	-1.01863	IM+PG down vs IM
mmu-miR-7077-5p	0.666667	-1.01863	IM+PG down vs IM
mmu-miR-706	0.666667	-1.01863	IM+PG down vs IM
mmu-miR-504-5p	0.666667	-1.01863	IM+PG down vs IM
mmu-let-7e	0.666667	-1.01863	IM+PG down vs IM
mmu-miR-7215-3p	0.666667	-1.01863	IM+PG down vs IM
mmu-miR-7657-5p	0.666667	-1.01863	IM+PG down vs IM
mmu-miR-1966-5p	0.666667	-1.01863	IM+PG down vs IM
mmu-mir-767	0.666667	-1.01863	IM+PG down vs IM
mmu-miR-7578	0.666667	-1.01863	IM+PG down vs IM
mmu-miR-6481	0.666667	-1.01863	IM+PG down vs IM
mmu-mir-669e	0.666667	-1.01863	IM+PG down vs IM
mmu-mir-5136	0.666667	-1.01863	IM+PG down vs IM
mmu-mir-7063	0.666667	-1.01863	IM+PG down vs IM
mmu-miR-7059-5p	0.666667	-1.01863	IM+PG down vs IM
mmu-let-7i-3p	0.666667	-1.01863	IM+PG down vs IM
mmu-mir-125b-1	0.666667	-1.01863	IM+PG down vs IM
mmu-mir-540	0.666667	-1.01863	IM+PG down vs IM
mmu-miR-137-5p	0.666667	-1.01863	IM+PG down vs IM
mmu-mir-3067	0.666667	-1.01863	IM+PG down vs IM
mmu-miR-3970	0.666667	-1.01863	IM+PG down vs IM
mmu-mir-683-1	0.666667	-1.01863	IM+PG down vs IM
mmu-mir-683-2	0.666667	-1.01863	IM+PG down vs IM
mmu-miR-669b-5p	0.666667	-1.01863	IM+PG down vs IM
mmu-mir-130a	0.666667	-1.01863	IM+PG down vs IM
mmu-mir-19b-2	0.666667	-1.01863	IM+PG down vs IM
mmu-mir-19b-2	0.666667	-1.01863	IM+PG down vs IM
mmu-mir-148a	0.666667	-1.01863	IM+PG down vs IM
mmu-mir-3475	0.666667	-1.01863	IM+PG down vs IM
mmu-mir-1191	0.666667	-1.01863	IM+PG down vs IM

mmu-mir-1892	0.666667	-1.01863	IM+PG down vs IM
mmu-mir-5619	0.666667	-1.01863	IM+PG down vs IM
mmu-mir-6362	0.666667	-1.01863	IM+PG down vs IM
mmu-mir-6957	0.666667	-1.01863	IM+PG down vs IM
mmu-mir-7211	0.666667	-1.01863	IM+PG down vs IM
mmu-miR-183-5p	0.666667	-1.01863	IM+PG down vs IM
mmu-miR-219a-5p	0.666667	-1.01863	IM+PG down vs IM
mmu-miR-365-3p	0.666667	-1.01863	IM+PG down vs IM
mmu-miR-375-3p	0.666667	-1.01863	IM+PG down vs IM
mmu-miR-7a-1-3p	0.666667	-1.01863	IM+PG down vs IM
mmu-miR-344d-2-5p	0.666667	-1.01863	IM+PG down vs IM
mmu-miR-16-2-3p	0.666667	-1.01863	IM+PG down vs IM
mmu-miR-184-5p	0.666667	-1.01863	IM+PG down vs IM
mmu-miR-6381	0.666667	-1.01863	IM+PG down vs IM
mmu-miR-7015-3p	0.666667	-1.01863	IM+PG down vs IM
mmu-miR-142b	0.666667	-1.01863	IM+PG down vs IM
mmu-mir-343	0.948917	-1.01849	IM+PG down vs IM
mmu-mir-302a	0.91671	-1.018	IM+PG down vs IM
mmu-mir-92a-2	0.939328	-1.01792	IM+PG down vs IM
mmu-miR-467d-5p	0.915892	-1.01646	IM+PG down vs IM
mmu-miR-883b-5p	0.887712	-1.01633	IM+PG down vs IM
mmu-mir-378c	0.666667	-1.01606	IM+PG down vs IM
mmu-mir-7022	0.666667	-1.01606	IM+PG down vs IM
mmu-miR-6975-3p	0.666667	-1.01606	IM+PG down vs IM
mmu-mir-302d	0.666667	-1.01606	IM+PG down vs IM
mmu-let-7f-1-3p	0.666667	-1.01606	IM+PG down vs IM
mmu-miR-5618-3p	0.666667	-1.01606	IM+PG down vs IM
mmu-mir-219c	0.666667	-1.01606	IM+PG down vs IM
mmu-miR-6949-3p	0.666667	-1.01606	IM+PG down vs IM
mmu-mir-219b	0.666667	-1.01606	IM+PG down vs IM
mmu-miR-467c-3p	0.666667	-1.01606	IM+PG down vs IM
mmu-miR-5134-5p	0.666667	-1.01606	IM+PG down vs IM
mmu-mir-3094	0.666667	-1.01606	IM+PG down vs IM
mmu-miR-7008-3p	0.666667	-1.01606	IM+PG down vs IM
mmu-mir-1197	0.666667	-1.01606	IM+PG down vs IM
mmu-mir-5121	0.666667	-1.01606	IM+PG down vs IM
mmu-mir-129b	0.666667	-1.01606	IM+PG down vs IM
mmu-miR-3113-3p	0.666667	-1.01606	IM+PG down vs IM
mmu-miR-6386	0.666667	-1.01606	IM+PG down vs IM
mmu-miR-7040-3p	0.666667	-1.01606	IM+PG down vs IM
mmu-miR-7235-3p	0.666667	-1.01606	IM+PG down vs IM
mmu-miR-7084-5p	0.928541	-1.01477	IM+PG down vs IM
mmu-miR-467g	0.666667	-1.01473	IM+PG down vs IM

mmu-miR-3105-3p	0.958804	-1.01442	IM+PG down vs IM
mmu-let-7c-5p	0.910462	-1.01399	IM+PG down vs IM
mmu-miR-7027-3p	0.666667	-1.01387	IM+PG down vs IM
mmu-mir-7241	0.666667	-1.01387	IM+PG down vs IM
mmu-miR-8098	0.666667	-1.01387	IM+PG down vs IM
mmu-miR-7055-3p	0.979799	-1.01248	IM+PG down vs IM
mmu-miR-6923-5p	0.87481	-1.01246	IM+PG down vs IM
mmu-miR-216b-5p	0.978027	-1.01206	IM+PG down vs IM
mmu-mir-8106	0.967547	-1.01154	IM+PG down vs IM
mmu-miR-203-5p	0.975669	-1.01138	IM+PG down vs IM
mmu-miR-128-3p	0.950953	-1.01104	IM+PG down vs IM
mmu-mir-1897	0.913246	-1.01049	IM+PG down vs IM
mmu-miR-7678-3p	0.975635	-1.01048	IM+PG down vs IM
mmu-miR-6927-5p	0.908871	-1.01022	IM+PG down vs IM
mmu-miR-3078-3p	0.909021	-1.0102	IM+PG down vs IM
mmu-mir-5125	0.918004	-1.00888	IM+PG down vs IM
mmu-miR-466a-3p	0.918004	-1.00888	IM+PG down vs IM
mmu-miR-466e-3p	0.918004	-1.00888	IM+PG down vs IM
mmu-mir-7013	0.918004	-1.00888	IM+PG down vs IM
mmu-mir-7021	0.973761	-1.00875	IM+PG down vs IM
mmu-mir-26a-2	0	-1.00865	IM+PG down vs IM
mmu-miR-215-5p	0.931541	-1.00838	IM+PG down vs IM
mmu-miR-7069-3p	0.981933	-1.00836	IM+PG down vs IM
mmu-mir-6967	0.933877	-1.00741	IM+PG down vs IM
mmu-mir-219a-2	0.976047	-1.00735	IM+PG down vs IM
mmu-miR-6944-3p	0.935008	-1.00725	IM+PG down vs IM
mmu-miR-7663-3p	0.918776	-1.00674	IM+PG down vs IM
mmu-miR-224-5p	0.968851	-1.00639	IM+PG down vs IM
mmu-mir-153	0.973096	-1.00603	IM+PG down vs IM
mmu-miR-344d-3-5p	0.939908	-1.00602	IM+PG down vs IM
mmu-mir-6539	0.99106	-1.00528	IM+PG down vs IM
mmu-miR-301a-5p	0.974418	-1.00524	IM+PG down vs IM
mmu-miR-718	0.958982	-1.00486	IM+PG down vs IM
mmu-miR-6932-3p	0.956241	-1.00455	IM+PG down vs IM
mmu-miR-6416-3p	0.991711	-1.0045	IM+PG down vs IM
mmu-mir-1943	0.980112	-1.00445	IM+PG down vs IM
mmu-miR-299b-3p	0.981235	-1.00389	IM+PG down vs IM
mmu-miR-129b-3p	0.988348	-1.00344	IM+PG down vs IM
mmu-mir-5131	0.97193	-1.0032	IM+PG down vs IM
mmu-miR-215-3p	0.965462	-1.00318	IM+PG down vs IM
mmu-miR-6360	0.965462	-1.00318	IM+PG down vs IM
mmu-miR-3966	0.965462	-1.00318	IM+PG down vs IM
mmu-miR-202-3p	0.970678	-1.00315	IM+PG down vs IM

mmu-miR-471-3p	0.970678	-1.00315	IM+PG down vs IM
mmu-miR-6924-3p	0.968884	-1.00311	IM+PG down vs IM
mmu-mir-127	0.99307	-1.00262	IM+PG down vs IM
mmu-mir-7655	0.99307	-1.00262	IM+PG down vs IM
mmu-mir-1194	0.982126	-1.00171	IM+PG down vs IM
mmu-mir-6390	0.998118	-1.00067	IM+PG down vs IM
mmu-miR-744-3p	0.99674	-1.00032	IM+PG down vs IM
mmu-mir-9-2	?	1	ange between IM+PG and IM
mmu-mir-133a-1	?	1	ange between IM+PG and IM
mmu-mir-140	?	1	ange between IM+PG and IM
mmu-mir-187	?	1	ange between IM+PG and IM
mmu-mir-203	?	1	ange between IM+PG and IM
mmu-mir-298	?	1	ange between IM+PG and IM
mmu-mir-34c	?	1	ange between IM+PG and IM
mmu-mir-130b	?	1	ange between IM+PG and IM
mmu-mir-196a-1	?	1	ange between IM+PG and IM
mmu-let-7b	?	1	ange between IM+PG and IM
mmu-mir-96	?	1	ange between IM+PG and IM
mmu-mir-339	?	1	ange between IM+PG and IM
mmu-mir-345	?	1	ange between IM+PG and IM
mmu-mir-138-1	?	1	ange between IM+PG and IM
mmu-mir-362	?	1	ange between IM+PG and IM
mmu-mir-377	?	1	ange between IM+PG and IM
mmu-mir-378a	?	1	ange between IM+PG and IM
mmu-mir-335	?	1	ange between IM+PG and IM
mmu-mir-133a-2	?	1	ange between IM+PG and IM
mmu-mir-215	?	1	ange between IM+PG and IM
mmu-mir-466a	?	1	ange between IM+PG and IM
mmu-mir-468	?	1	ange between IM+PG and IM
mmu-mir-483	?	1	ange between IM+PG and IM
mmu-mir-367	?	1	ange between IM+PG and IM
mmu-mir-503	?	1	ange between IM+PG and IM
mmu-mir-1249	?	1	ange between IM+PG and IM
mmu-mir-770	?	1	ange between IM+PG and IM
mmu-mir-672	?	1	ange between IM+PG and IM
mmu-mir-3059	?	1	ange between IM+PG and IM
mmu-mir-674	?	1	ange between IM+PG and IM
mmu-mir-680-1	?	1	ange between IM+PG and IM
mmu-mir-688	?	1	ange between IM+PG and IM
mmu-mir-697	?	1	ange between IM+PG and IM
mmu-mir-704	?	1	ange between IM+PG and IM
mmu-mir-707	?	1	ange between IM+PG and IM
mmu-mir-713	?	1	ange between IM+PG and IM

mmu-mir-500	?	1	ange between IM+PG and IM
mmu-mir-615	?	1	ange between IM+PG and IM
mmu-mir-181d	?	1	ange between IM+PG and IM
mmu-mir-181d	?	1	ange between IM+PG and IM
mmu-mir-871	?	1	ange between IM+PG and IM
mmu-mir-190b	?	1	ange between IM+PG and IM
mmu-mir-297c	?	1	ange between IM+PG and IM
mmu-mir-466e	?	1	ange between IM+PG and IM
mmu-mir-466f-2	?	1	ange between IM+PG and IM
mmu-mir-466g	?	1	ange between IM+PG and IM
mmu-mir-875	?	1	ange between IM+PG and IM
mmu-mir-669d	?	1	ange between IM+PG and IM
mmu-mir-669d	?	1	ange between IM+PG and IM
mmu-mir-669i	?	1	ange between IM+PG and IM
mmu-mir-1893	?	1	ange between IM+PG and IM
mmu-mir-1306	?	1	ange between IM+PG and IM
mmu-mir-1955	?	1	ange between IM+PG and IM
mmu-mir-1962	?	1	ange between IM+PG and IM
mmu-mir-1964	?	1	ange between IM+PG and IM
mmu-mir-1982	?	1	ange between IM+PG and IM
mmu-mir-3074-1	?	1	ange between IM+PG and IM
mmu-mir-3074-1	?	1	ange between IM+PG and IM
mmu-mir-3085	?	1	ange between IM+PG and IM
mmu-mir-669d-2	?	1	ange between IM+PG and IM
mmu-mir-466c-2	?	1	ange between IM+PG and IM
mmu-mir-3092	?	1	ange between IM+PG and IM
mmu-mir-3095	?	1	ange between IM+PG and IM
mmu-mir-3100	?	1	ange between IM+PG and IM
mmu-mir-344f	?	1	ange between IM+PG and IM
mmu-mir-3967	?	1	ange between IM+PG and IM
mmu-mir-5046	?	1	ange between IM+PG and IM
mmu-mir-5120	?	1	ange between IM+PG and IM
mmu-mir-5122	?	1	ange between IM+PG and IM
mmu-mir-5123	?	1	ange between IM+PG and IM
mmu-mir-5617	?	1	ange between IM+PG and IM
mmu-mir-6237	?	1	ange between IM+PG and IM
mmu-mir-6348	?	1	ange between IM+PG and IM
mmu-mir-6349	?	1	ange between IM+PG and IM
mmu-mir-6350	?	1	ange between IM+PG and IM
mmu-mir-21b	?	1	ange between IM+PG and IM
mmu-mir-6381	?	1	ange between IM+PG and IM
mmu-mir-6395	?	1	ange between IM+PG and IM
mmu-mir-6396	?	1	ange between IM+PG and IM

mmu-mir-6403	?	1 ange between IM+PG and IM
mmu-mir-6411	?	1 ange between IM+PG and IM
mmu-mir-6412	?	1 ange between IM+PG and IM
mmu-mir-6415	?	1 ange between IM+PG and IM
mmu-mir-6419	?	1 ange between IM+PG and IM
mmu-mir-6540	?	1 ange between IM+PG and IM
mmu-mir-6901	?	1 ange between IM+PG and IM
mmu-mir-6902	?	1 ange between IM+PG and IM
mmu-mir-6904	?	1 ange between IM+PG and IM
mmu-mir-6905	?	1 ange between IM+PG and IM
mmu-mir-6922	?	1 ange between IM+PG and IM
mmu-mir-6927	?	1 ange between IM+PG and IM
mmu-mir-6934	?	1 ange between IM+PG and IM
mmu-mir-6936	?	1 ange between IM+PG and IM
mmu-mir-6944	?	1 ange between IM+PG and IM
mmu-mir-6947	?	1 ange between IM+PG and IM
mmu-mir-6949	?	1 ange between IM+PG and IM
mmu-mir-6959	?	1 ange between IM+PG and IM
mmu-mir-6969	?	1 ange between IM+PG and IM
mmu-mir-6976	?	1 ange between IM+PG and IM
mmu-mir-6989	?	1 ange between IM+PG and IM
mmu-mir-6990	?	1 ange between IM+PG and IM
mmu-mir-7008	?	1 ange between IM+PG and IM
mmu-mir-7019	?	1 ange between IM+PG and IM
mmu-mir-7040	?	1 ange between IM+PG and IM
mmu-mir-7041	?	1 ange between IM+PG and IM
mmu-mir-7042	?	1 ange between IM+PG and IM
mmu-mir-7047	?	1 ange between IM+PG and IM
mmu-mir-7059	?	1 ange between IM+PG and IM
mmu-mir-7061	?	1 ange between IM+PG and IM
mmu-mir-7080	?	1 ange between IM+PG and IM
mmu-mir-7081	?	1 ange between IM+PG and IM
mmu-mir-7091	?	1 ange between IM+PG and IM
mmu-mir-7223	?	1 ange between IM+PG and IM
mmu-mir-7229	?	1 ange between IM+PG and IM
mmu-mir-7232	?	1 ange between IM+PG and IM
mmu-mir-7235	?	1 ange between IM+PG and IM
mmu-mir-7238	?	1 ange between IM+PG and IM
mmu-mir-7578	?	1 ange between IM+PG and IM
mmu-mir-7658	?	1 ange between IM+PG and IM
mmu-mir-7675	?	1 ange between IM+PG and IM
mmu-mir-7676-1	?	1 ange between IM+PG and IM
mmu-mir-7676-2	?	1 ange between IM+PG and IM

mmu-mir-7680	?	1	ange between IM+PG and IM
mmu-mir-7687	?	1	ange between IM+PG and IM
mmu-mir-1258	?	1	ange between IM+PG and IM
mmu-mir-8092	?	1	ange between IM+PG and IM
mmu-mir-8104	?	1	ange between IM+PG and IM
mmu-mir-8116	?	1	ange between IM+PG and IM
mmu-miR-29b-3p	?	1	ange between IM+PG and IM
mmu-miR-127-3p	?	1	ange between IM+PG and IM
mmu-miR-9-3p	?	1	ange between IM+PG and IM
mmu-miR-186-5p	?	1	ange between IM+PG and IM
mmu-miR-195a-5p	?	1	ange between IM+PG and IM
mmu-miR-30e-5p	?	1	ange between IM+PG and IM
mmu-miR-297a-5p	?	1	ange between IM+PG and IM
mmu-miR-19b-3p	?	1	ange between IM+PG and IM
mmu-miR-326-3p	?	1	ange between IM+PG and IM
mmu-miR-344-3p	?	1	ange between IM+PG and IM
mmu-miR-223-3p	?	1	ange between IM+PG and IM
mmu-miR-33-5p	?	1	ange between IM+PG and IM
mmu-miR-133b-3p	?	1	ange between IM+PG and IM
mmu-miR-369-3p	?	1	ange between IM+PG and IM
mmu-miR-302c-5p	?	1	ange between IM+PG and IM
mmu-miR-497-5p	?	1	ange between IM+PG and IM
mmu-miR-133a-5p	?	1	ange between IM+PG and IM
mmu-miR-669c-5p	?	1	ange between IM+PG and IM
mmu-miR-297b-5p	?	1	ange between IM+PG and IM
mmu-miR-700-3p	?	1	ange between IM+PG and IM
mmu-miR-707	?	1	ange between IM+PG and IM
mmu-miR-501-5p	?	1	ange between IM+PG and IM
mmu-miR-676-5p	?	1	ange between IM+PG and IM
mmu-miR-761	?	1	ange between IM+PG and IM
mmu-miR-744-5p	?	1	ange between IM+PG and IM
mmu-miR-125b-2-3p	?	1	ange between IM+PG and IM
mmu-miR-127-5p	?	1	ange between IM+PG and IM
mmu-miR-188-3p	?	1	ange between IM+PG and IM
mmu-miR-302a-5p	?	1	ange between IM+PG and IM
mmu-let-7a-1-3p	?	1	ange between IM+PG and IM
mmu-miR-93-3p	?	1	ange between IM+PG and IM
mmu-miR-323-5p	?	1	ange between IM+PG and IM
mmu-miR-325-3p	?	1	ange between IM+PG and IM
mmu-miR-10a-3p	?	1	ange between IM+PG and IM
mmu-miR-138-1-3p	?	1	ange between IM+PG and IM
mmu-miR-20b-3p	?	1	ange between IM+PG and IM
mmu-miR-297b-3p	?	1	ange between IM+PG and IM

mmu-miR-742-5p	?	1	ange between IM+PG and IM
mmu-miR-297a-3p	?	1	ange between IM+PG and IM
mmu-miR-297c-3p	?	1	ange between IM+PG and IM
mmu-miR-466b-3p	?	1	ange between IM+PG and IM
mmu-miR-466c-3p	?	1	ange between IM+PG and IM
mmu-miR-466e-5p	?	1	ange between IM+PG and IM
mmu-miR-582-3p	?	1	ange between IM+PG and IM
mmu-miR-376c-5p	?	1	ange between IM+PG and IM
mmu-let-7c-2-3p	?	1	ange between IM+PG and IM
mmu-miR-669d-5p	?	1	ange between IM+PG and IM
mmu-miR-466j	?	1	ange between IM+PG and IM
mmu-miR-1197-3p	?	1	ange between IM+PG and IM
mmu-miR-1900	?	1	ange between IM+PG and IM
mmu-miR-1907	?	1	ange between IM+PG and IM
mmu-miR-1894-5p	?	1	ange between IM+PG and IM
mmu-miR-1927	?	1	ange between IM+PG and IM
mmu-miR-1933-5p	?	1	ange between IM+PG and IM
mmu-miR-1933-3p	?	1	ange between IM+PG and IM
mmu-miR-1945	?	1	ange between IM+PG and IM
mmu-miR-1306-3p	?	1	ange between IM+PG and IM
mmu-miR-669m-3p	?	1	ange between IM+PG and IM
mmu-miR-669o-5p	?	1	ange between IM+PG and IM
mmu-miR-1951	?	1	ange between IM+PG and IM
mmu-miR-669n	?	1	ange between IM+PG and IM
mmu-miR-1969	?	1	ange between IM+PG and IM
mmu-miR-1247-5p	?	1	ange between IM+PG and IM
mmu-miR-1298-3p	?	1	ange between IM+PG and IM
mmu-miR-3061-3p	?	1	ange between IM+PG and IM
mmu-miR-3067-3p	?	1	ange between IM+PG and IM
mmu-miR-3069-3p	?	1	ange between IM+PG and IM
mmu-miR-3072-5p	?	1	ange between IM+PG and IM
mmu-miR-466o-3p	?	1	ange between IM+PG and IM
mmu-miR-466p-3p	?	1	ange between IM+PG and IM
mmu-miR-3093-3p	?	1	ange between IM+PG and IM
mmu-miR-3094-5p	?	1	ange between IM+PG and IM
mmu-miR-3101-5p	?	1	ange between IM+PG and IM
mmu-miR-344b-5p	?	1	ange between IM+PG and IM
mmu-miR-3104-5p	?	1	ange between IM+PG and IM
mmu-miR-3470b	?	1	ange between IM+PG and IM
mmu-miR-132-5p	?	1	ange between IM+PG and IM
mmu-miR-153-5p	?	1	ange between IM+PG and IM
mmu-miR-135b-3p	?	1	ange between IM+PG and IM
mmu-miR-135a-2-3p	?	1	ange between IM+PG and IM

mmu-miR-128-2-5p	?	1	ange between IM+PG and IM
mmu-miR-361-3p	?	1	ange between IM+PG and IM
mmu-miR-363-5p	?	1	ange between IM+PG and IM
mmu-miR-547-5p	?	1	ange between IM+PG and IM
mmu-miR-668-5p	?	1	ange between IM+PG and IM
mmu-miR-665-5p	?	1	ange between IM+PG and IM
mmu-miR-672-3p	?	1	ange between IM+PG and IM
mmu-miR-670-3p	?	1	ange between IM+PG and IM
mmu-miR-700-5p	?	1	ange between IM+PG and IM
mmu-miR-421-5p	?	1	ange between IM+PG and IM
mmu-miR-1199-3p	?	1	ange between IM+PG and IM
mmu-miR-664-5p	?	1	ange between IM+PG and IM
mmu-miR-3964	?	1	ange between IM+PG and IM
mmu-miR-3969	?	1	ange between IM+PG and IM
mmu-miR-5106	?	1	ange between IM+PG and IM
mmu-miR-5122	?	1	ange between IM+PG and IM
mmu-miR-5124a	?	1	ange between IM+PG and IM
mmu-miR-5615-3p	?	1	ange between IM+PG and IM
mmu-miR-1231-5p	?	1	ange between IM+PG and IM
mmu-miR-5622-5p	?	1	ange between IM+PG and IM
mmu-miR-5622-3p	?	1	ange between IM+PG and IM
mmu-miR-5626-3p	?	1	ange between IM+PG and IM
mmu-miR-5710	?	1	ange between IM+PG and IM
mmu-miR-1929-3p	?	1	ange between IM+PG and IM
mmu-miR-219a-2-3p	?	1	ange between IM+PG and IM
mmu-miR-5132-3p	?	1	ange between IM+PG and IM
mmu-miR-6238	?	1	ange between IM+PG and IM
mmu-miR-6244	?	1	ange between IM+PG and IM
mmu-miR-6351	?	1	ange between IM+PG and IM
mmu-miR-6354	?	1	ange between IM+PG and IM
mmu-miR-6356	?	1	ange between IM+PG and IM
mmu-miR-6370	?	1	ange between IM+PG and IM
mmu-miR-6380	?	1	ange between IM+PG and IM
mmu-miR-6392-5p	?	1	ange between IM+PG and IM
mmu-miR-6407	?	1	ange between IM+PG and IM
mmu-miR-6409	?	1	ange between IM+PG and IM
mmu-miR-6410	?	1	ange between IM+PG and IM
mmu-miR-6419	?	1	ange between IM+PG and IM
mmu-miR-6540-5p	?	1	ange between IM+PG and IM
mmu-miR-6896-5p	?	1	ange between IM+PG and IM
mmu-miR-6901-5p	?	1	ange between IM+PG and IM
mmu-miR-6904-3p	?	1	ange between IM+PG and IM
mmu-miR-6914-5p	?	1	ange between IM+PG and IM

mmu-miR-6918-5p	?	1	ange between IM+PG and IM
mmu-miR-6919-5p	?	1	ange between IM+PG and IM
mmu-miR-6921-5p	?	1	ange between IM+PG and IM
mmu-miR-6923-3p	?	1	ange between IM+PG and IM
mmu-miR-6926-5p	?	1	ange between IM+PG and IM
mmu-miR-6945-5p	?	1	ange between IM+PG and IM
mmu-miR-6953-3p	?	1	ange between IM+PG and IM
mmu-miR-6956-5p	?	1	ange between IM+PG and IM
mmu-miR-6958-5p	?	1	ange between IM+PG and IM
mmu-miR-6961-3p	?	1	ange between IM+PG and IM
mmu-miR-6969-5p	?	1	ange between IM+PG and IM
mmu-miR-6978-3p	?	1	ange between IM+PG and IM
mmu-miR-6988-3p	?	1	ange between IM+PG and IM
mmu-miR-6995-5p	?	1	ange between IM+PG and IM
mmu-miR-7004-5p	?	1	ange between IM+PG and IM
mmu-miR-7009-3p	?	1	ange between IM+PG and IM
mmu-miR-7011-5p	?	1	ange between IM+PG and IM
mmu-miR-7014-3p	?	1	ange between IM+PG and IM
mmu-miR-7015-5p	?	1	ange between IM+PG and IM
mmu-miR-7018-5p	?	1	ange between IM+PG and IM
mmu-miR-7031-5p	?	1	ange between IM+PG and IM
mmu-miR-7032-3p	?	1	ange between IM+PG and IM
mmu-miR-7033-5p	?	1	ange between IM+PG and IM
mmu-miR-7052-3p	?	1	ange between IM+PG and IM
mmu-miR-7062-5p	?	1	ange between IM+PG and IM
mmu-miR-7065-5p	?	1	ange between IM+PG and IM
mmu-miR-7067-3p	?	1	ange between IM+PG and IM
mmu-miR-7068-3p	?	1	ange between IM+PG and IM
mmu-miR-7079-3p	?	1	ange between IM+PG and IM
mmu-miR-7083-5p	?	1	ange between IM+PG and IM
mmu-miR-7093-5p	?	1	ange between IM+PG and IM
mmu-miR-7214-5p	?	1	ange between IM+PG and IM
mmu-miR-7217-3p	?	1	ange between IM+PG and IM
mmu-miR-7224-3p	?	1	ange between IM+PG and IM
mmu-miR-7226-5p	?	1	ange between IM+PG and IM
mmu-miR-7228-5p	?	1	ange between IM+PG and IM
mmu-miR-7234-3p	?	1	ange between IM+PG and IM
mmu-miR-7239-5p	?	1	ange between IM+PG and IM
mmu-miR-7647-3p	?	1	ange between IM+PG and IM
mmu-miR-7660-5p	?	1	ange between IM+PG and IM
mmu-miR-7673-3p	?	1	ange between IM+PG and IM
mmu-miR-7680-5p	?	1	ange between IM+PG and IM
mmu-miR-7681-5p	?	1	ange between IM+PG and IM

mmu-miR-7682-3p	?	1	ange between IM+PG and IM
mmu-miR-216c-5p	?	1	ange between IM+PG and IM
mmu-miR-290b-5p	?	1	ange between IM+PG and IM
mmu-miR-8102	?	1	ange between IM+PG and IM
mmu-miR-8107	?	1	ange between IM+PG and IM
mmu-miR-8116	?	1	ange between IM+PG and IM
mmu-miR-200c-3p	1	1	ange between IM+PG and IM
mmu-let-7i	?	1	ange between IM+PG and IM
mmu-mir-99a	?	1	ange between IM+PG and IM
mmu-mir-126a	?	1	ange between IM+PG and IM
mmu-mir-128-1	?	1	ange between IM+PG and IM
mmu-mir-133a-1	?	1	ange between IM+PG and IM
mmu-mir-152	?	1	ange between IM+PG and IM
mmu-mir-191	?	1	ange between IM+PG and IM
mmu-mir-206	?	1	ange between IM+PG and IM
mmu-mir-290a	?	1	ange between IM+PG and IM
mmu-let-7d	?	1	ange between IM+PG and IM
mmu-let-7f-2	?	1	ange between IM+PG and IM
mmu-mir-26b	?	1	ange between IM+PG and IM
mmu-mir-103-2	?	1	ange between IM+PG and IM
mmu-mir-341	?	1	ange between IM+PG and IM
mmu-mir-346	?	1	ange between IM+PG and IM
mmu-mir-199b	?	1	ange between IM+PG and IM
mmu-mir-9-1	?	1	ange between IM+PG and IM
mmu-mir-9-1	?	1	ange between IM+PG and IM
mmu-mir-363	?	1	ange between IM+PG and IM
mmu-mir-376a	?	1	ange between IM+PG and IM
mmu-mir-379	?	1	ange between IM+PG and IM
mmu-mir-665	?	1	ange between IM+PG and IM
mmu-mir-1298	?	1	ange between IM+PG and IM
mmu-mir-686	?	1	ange between IM+PG and IM
mmu-mir-504	?	1	ange between IM+PG and IM
mmu-mir-669g	?	1	ange between IM+PG and IM
mmu-mir-669j	?	1	ange between IM+PG and IM
mmu-mir-1195	?	1	ange between IM+PG and IM
mmu-mir-1904	?	1	ange between IM+PG and IM
mmu-mir-1934	?	1	ange between IM+PG and IM
mmu-mir-466m	?	1	ange between IM+PG and IM
mmu-mir-3110	?	1	ange between IM+PG and IM
mmu-mir-3472	?	1	ange between IM+PG and IM
mmu-mir-5114	?	1	ange between IM+PG and IM
mmu-mir-5116	?	1	ange between IM+PG and IM
mmu-mir-5127	?	1	ange between IM+PG and IM

mmu-mir-5134	?	1	ange between IM+PG and IM
mmu-mir-5618	?	1	ange between IM+PG and IM
mmu-mir-5710	?	1	ange between IM+PG and IM
mmu-mir-6241	?	1	ange between IM+PG and IM
mmu-mir-6353	?	1	ange between IM+PG and IM
mmu-mir-6360	?	1	ange between IM+PG and IM
mmu-mir-145b	?	1	ange between IM+PG and IM
mmu-mir-6366	?	1	ange between IM+PG and IM
mmu-mir-6376	?	1	ange between IM+PG and IM
mmu-mir-6377	?	1	ange between IM+PG and IM
mmu-mir-6394	?	1	ange between IM+PG and IM
mmu-mir-6395	?	1	ange between IM+PG and IM
mmu-mir-451b	?	1	ange between IM+PG and IM
mmu-mir-451b	?	1	ange between IM+PG and IM
mmu-mir-6923	?	1	ange between IM+PG and IM
mmu-mir-6924	?	1	ange between IM+PG and IM
mmu-mir-6950	?	1	ange between IM+PG and IM
mmu-mir-6951	?	1	ange between IM+PG and IM
mmu-mir-6974	?	1	ange between IM+PG and IM
mmu-mir-6995	?	1	ange between IM+PG and IM
mmu-mir-6999	?	1	ange between IM+PG and IM
mmu-mir-7020	?	1	ange between IM+PG and IM
mmu-mir-7024	?	1	ange between IM+PG and IM
mmu-mir-7036	?	1	ange between IM+PG and IM
mmu-mir-7058	?	1	ange between IM+PG and IM
mmu-mir-7070	?	1	ange between IM+PG and IM
mmu-mir-7073	?	1	ange between IM+PG and IM
mmu-mir-7221	?	1	ange between IM+PG and IM
mmu-mir-7222	?	1	ange between IM+PG and IM
mmu-mir-7227	?	1	ange between IM+PG and IM
mmu-mir-7674	?	1	ange between IM+PG and IM
mmu-mir-216c	?	1	ange between IM+PG and IM
mmu-mir-8094	?	1	ange between IM+PG and IM
mmu-mir-8110	?	1	ange between IM+PG and IM
mmu-mir-8115	?	1	ange between IM+PG and IM
mmu-miR-99a-5p	?	1	ange between IM+PG and IM
mmu-miR-133a-3p	?	1	ange between IM+PG and IM
mmu-miR-196a-5p	?	1	ange between IM+PG and IM
mmu-miR-200a-3p	?	1	ange between IM+PG and IM
mmu-miR-340-3p	?	1	ange between IM+PG and IM
mmu-miR-363-3p	?	1	ange between IM+PG and IM
mmu-miR-433-5p	?	1	ange between IM+PG and IM
mmu-miR-291b-5p	?	1	ange between IM+PG and IM

mmu-miR-684	?	1	ange between IM+PG and IM
mmu-miR-713	?	1	ange between IM+PG and IM
mmu-miR-505-3p	?	1	ange between IM+PG and IM
mmu-miR-592-5p	?	1	ange between IM+PG and IM
mmu-miR-671-5p	?	1	ange between IM+PG and IM
mmu-miR-551b-3p	?	1	ange between IM+PG and IM
mmu-miR-22-5p	?	1	ange between IM+PG and IM
mmu-miR-339-3p	?	1	ange between IM+PG and IM
mmu-miR-743b-5p	?	1	ange between IM+PG and IM
mmu-miR-105	?	1	ange between IM+PG and IM
mmu-miR-343	?	1	ange between IM+PG and IM
mmu-miR-875-5p	?	1	ange between IM+PG and IM
mmu-miR-669k-3p	?	1	ange between IM+PG and IM
mmu-miR-1898	?	1	ange between IM+PG and IM
mmu-miR-1938	?	1	ange between IM+PG and IM
mmu-miR-344d-1-5p	?	1	ange between IM+PG and IM
mmu-miR-3066-5p	?	1	ange between IM+PG and IM
mmu-miR-3080-3p	?	1	ange between IM+PG and IM
mmu-miR-3101-3p	?	1	ange between IM+PG and IM
mmu-miR-206-5p	?	1	ange between IM+PG and IM
mmu-miR-96-3p	?	1	ange between IM+PG and IM
mmu-miR-34a-3p	?	1	ange between IM+PG and IM
mmu-miR-301b-5p	?	1	ange between IM+PG and IM
mmu-miR-544-5p	?	1	ange between IM+PG and IM
mmu-miR-598-5p	?	1	ange between IM+PG and IM
mmu-miR-466i-5p	?	1	ange between IM+PG and IM
mmu-miR-1948-5p	?	1	ange between IM+PG and IM
mmu-miR-5104	?	1	ange between IM+PG and IM
mmu-miR-5118	?	1	ange between IM+PG and IM
mmu-miR-5625-5p	?	1	ange between IM+PG and IM
mmu-miR-6237	?	1	ange between IM+PG and IM
mmu-miR-6387	?	1	ange between IM+PG and IM
mmu-miR-5709-3p	?	1	ange between IM+PG and IM
mmu-miR-6912-3p	?	1	ange between IM+PG and IM
mmu-miR-6915-3p	?	1	ange between IM+PG and IM
mmu-miR-6994-3p	?	1	ange between IM+PG and IM
mmu-miR-7001-3p	?	1	ange between IM+PG and IM
mmu-miR-7026-3p	?	1	ange between IM+PG and IM
mmu-miR-7074-3p	?	1	ange between IM+PG and IM
mmu-miR-7078-3p	?	1	ange between IM+PG and IM
mmu-miR-7081-3p	?	1	ange between IM+PG and IM
mmu-miR-7211-5p	?	1	ange between IM+PG and IM
mmu-miR-7231-5p	?	1	ange between IM+PG and IM

mmu-miR-7665-5p	?	1	ange between IM+PG and IM
mmu-miR-3569-5p	?	1	ange between IM+PG and IM
mmu-miR-7676-5p	?	1	ange between IM+PG and IM
mmu-miR-1191b-5p	?	1	ange between IM+PG and IM
mmu-miR-465d-3p	?	1	ange between IM+PG and IM
mmu-miR-6980-5p	0.998942	1.00046	IM+PG up vs IM
mmu-miR-3097-5p	0.99718	1.00063	IM+PG up vs IM
mmu-mir-1929	0.997088	1.0007	IM+PG up vs IM
mmu-miR-411-5p	0.997088	1.0007	IM+PG up vs IM
mmu-mir-449c	0.997088	1.0007	IM+PG up vs IM
mmu-mir-1187	0.997088	1.0007	IM+PG up vs IM
mmu-miR-5120	0.997088	1.0007	IM+PG up vs IM
mmu-mir-6961	0.995442	1.00117	IM+PG up vs IM
mmu-mir-3966	0.995921	1.00191	IM+PG up vs IM
mmu-miR-351-3p	0.993337	1.00242	IM+PG up vs IM
mmu-mir-466h	0.983276	1.00352	IM+PG up vs IM
mmu-mir-7673	0	1.00388	IM+PG up vs IM
mmu-miR-450b-3p	0.991773	1.00411	IM+PG up vs IM
mmu-mir-3084-1	0.985619	1.00488	IM+PG up vs IM
mmu-mir-3084-2	0.985619	1.00488	IM+PG up vs IM
mmu-mir-743a	0.900546	1.00505	IM+PG up vs IM
mmu-miR-7655-5p	0.900546	1.00505	IM+PG up vs IM
mmu-mir-881	0.900546	1.00505	IM+PG up vs IM
mmu-mir-34b	0.9891	1.00544	IM+PG up vs IM
mmu-miR-6960-3p	0.9891	1.00544	IM+PG up vs IM
mmu-mir-546	0.976093	1.00615	IM+PG up vs IM
mmu-miR-374b-3p	0.902596	1.00647	IM+PG up vs IM
mmu-miR-344h-3p	0.902595	1.00647	IM+PG up vs IM
mmu-miR-3473e	0.666667	1.00672	IM+PG up vs IM
mmu-mir-5623	0.985558	1.00674	IM+PG up vs IM
mmu-miR-137-3p	0.983301	1.00716	IM+PG up vs IM
mmu-mir-2139	0.983301	1.00716	IM+PG up vs IM
mmu-mir-409	0.96962	1.00737	IM+PG up vs IM
mmu-miR-3572-3p	0.944465	1.00798	IM+PG up vs IM
mmu-miR-1949	0.84284	1.00933	IM+PG up vs IM
mmu-miR-5616-5p	0.84284	1.00933	IM+PG up vs IM
mmu-miR-880-5p	0.859618	1.00941	IM+PG up vs IM
mmu-mir-7025	0.976861	1.00958	IM+PG up vs IM
mmu-mir-135a-2	0.866054	1.00984	IM+PG up vs IM
mmu-miR-7662-5p	0.666667	1.00989	IM+PG up vs IM
mmu-miR-6385	0	1.01044	IM+PG up vs IM
mmu-mir-8100	0.97906	1.01056	IM+PG up vs IM
mmu-mir-1933	0	1.01128	IM+PG up vs IM

mmu-mir-6930	0	1.01128	IM+PG up vs IM
mmu-miR-5624-5p	0.666667	1.01144	IM+PG up vs IM
mmu-miR-6941-5p	0.97253	1.01149	IM+PG up vs IM
mmu-mir-666	0.974223	1.01171	IM+PG up vs IM
mmu-mir-101b	0.666667	1.01182	IM+PG up vs IM
mmu-mir-448	0.666667	1.01182	IM+PG up vs IM
mmu-miR-204-3p	0.666667	1.01182	IM+PG up vs IM
mmu-miR-28b	0.666667	1.01182	IM+PG up vs IM
mmu-mir-3470a	0.666667	1.01182	IM+PG up vs IM
mmu-miR-540-5p	0.666667	1.01182	IM+PG up vs IM
mmu-mir-199a-1	0.666667	1.01182	IM+PG up vs IM
mmu-miR-34c-3p	0.666667	1.01182	IM+PG up vs IM
mmu-mir-486	0.969323	1.01198	IM+PG up vs IM
mmu-mir-466b-1	0.769481	1.01219	IM+PG up vs IM
mmu-miR-299a-5p	0.769481	1.01219	IM+PG up vs IM
mmu-mir-8117	0.769481	1.01219	IM+PG up vs IM
mmu-mir-466b-5	0.76948	1.01219	IM+PG up vs IM
mmu-mir-466b-7	0.76948	1.01219	IM+PG up vs IM
mmu-miR-3095-3p	0.76948	1.01219	IM+PG up vs IM
mmu-miR-6359	0.76948	1.01219	IM+PG up vs IM
mmu-mir-693	0.76948	1.01219	IM+PG up vs IM
mmu-miR-6404	0.76948	1.01219	IM+PG up vs IM
mmu-mir-7677	0.796329	1.01229	IM+PG up vs IM
mmu-miR-374c-5p	0.968604	1.01229	IM+PG up vs IM
mmu-miR-2139	0.967848	1.01264	IM+PG up vs IM
mmu-miR-5123	0.918386	1.01344	IM+PG up vs IM
mmu-miR-8090	0.876277	1.01398	IM+PG up vs IM
mmu-miR-6412	0.772623	1.01434	IM+PG up vs IM
mmu-miR-3967	0.689372	1.01475	IM+PG up vs IM
mmu-miR-6972-3p	0.689371	1.01475	IM+PG up vs IM
mmu-mir-6955	0.754141	1.0151	IM+PG up vs IM
mmu-mir-125b-1	0.201032	1.01514	IM+PG up vs IM
mmu-miR-190b-3p	0.720177	1.01515	IM+PG up vs IM
mmu-miR-6939-3p	0.720177	1.01515	IM+PG up vs IM
mmu-mir-721	0.666667	1.01541	IM+PG up vs IM
mmu-mir-495	0.666667	1.01541	IM+PG up vs IM
mmu-miR-30a-5p	0.666667	1.01541	IM+PG up vs IM
mmu-mir-3965	0.666667	1.01541	IM+PG up vs IM
mmu-miR-7013-3p	0.666667	1.01541	IM+PG up vs IM
mmu-miR-6950-5p	0.666667	1.01541	IM+PG up vs IM
mmu-miR-1981-3p	0.666667	1.01541	IM+PG up vs IM
mmu-mir-5124a	0.666667	1.01541	IM+PG up vs IM
mmu-mir-7219	0.666667	1.01541	IM+PG up vs IM

mmu-miR-291a-5p	0.958399	1.01541	IM+PG up vs IM
mmu-miR-1895	0.666667	1.01541	IM+PG up vs IM
mmu-mir-495	0.666667	1.01541	IM+PG up vs IM
mmu-mir-1192	0.666667	1.01541	IM+PG up vs IM
mmu-miR-7073-5p	0.666667	1.01541	IM+PG up vs IM
mmu-miR-568	0.666667	1.01541	IM+PG up vs IM
mmu-mir-105	0.666667	1.01541	IM+PG up vs IM
mmu-mir-6973a	0.666667	1.01541	IM+PG up vs IM
mmu-mir-5625	0.710472	1.01548	IM+PG up vs IM
mmu-mir-7659	0.666667	1.01606	IM+PG up vs IM
mmu-miR-6986-5p	0.969833	1.01615	IM+PG up vs IM
mmu-mir-7230	0.947811	1.01624	IM+PG up vs IM
mmu-mir-1b	0.956009	1.01642	IM+PG up vs IM
mmu-mir-690	0.666667	1.01689	IM+PG up vs IM
mmu-miR-6932-5p	0.666667	1.01689	IM+PG up vs IM
mmu-mir-6993	0.666667	1.01689	IM+PG up vs IM
mmu-miR-450a-2-3p	0.666667	1.01689	IM+PG up vs IM
mmu-miR-200b-3p	0.666667	1.01689	IM+PG up vs IM
mmu-mir-27a	0.666667	1.01689	IM+PG up vs IM
mmu-mir-452	0.666667	1.01689	IM+PG up vs IM
mmu-miR-7034-5p	0.666667	1.01689	IM+PG up vs IM
mmu-mir-1927	0.666667	1.01689	IM+PG up vs IM
mmu-miR-6384	0.666667	1.01689	IM+PG up vs IM
mmu-miR-202-5p	0.666667	1.01689	IM+PG up vs IM
mmu-mir-3061	0.666667	1.01689	IM+PG up vs IM
mmu-miR-6364	0.666667	1.01689	IM+PG up vs IM
mmu-mir-154	0.666667	1.01689	IM+PG up vs IM
mmu-mir-302b	0.666667	1.01689	IM+PG up vs IM
mmu-mir-743b	0.666667	1.01689	IM+PG up vs IM
mmu-mir-876	0.666667	1.01689	IM+PG up vs IM
mmu-mir-6365	0.666667	1.01689	IM+PG up vs IM
mmu-mir-6393	0.666667	1.01689	IM+PG up vs IM
mmu-mir-6921	0.666667	1.01689	IM+PG up vs IM
mmu-mir-6962	0.666667	1.01689	IM+PG up vs IM
mmu-mir-6973b	0.666667	1.01689	IM+PG up vs IM
mmu-mir-7026	0.666667	1.01689	IM+PG up vs IM
mmu-miR-540-3p	0.666667	1.01689	IM+PG up vs IM
mmu-miR-692	0.666667	1.01689	IM+PG up vs IM
mmu-miR-301b-3p	0.666667	1.01689	IM+PG up vs IM
mmu-miR-1893	0.666667	1.01689	IM+PG up vs IM
mmu-miR-6899-5p	0.666667	1.01689	IM+PG up vs IM
mmu-miR-6911-3p	0.666667	1.01689	IM+PG up vs IM
mmu-miR-654-3p	0.958297	1.01689	IM+PG up vs IM

mmu-mir-214	0.666667	1.01689	IM+PG up vs IM
mmu-miR-6376	0.639062	1.01772	IM+PG up vs IM
mmu-miR-344-5p	0.676447	1.01797	IM+PG up vs IM
mmu-miR-3094-3p	0.666667	1.0183	IM+PG up vs IM
mmu-miR-6241	0.666667	1.0183	IM+PG up vs IM
mmu-miR-466l-3p	0.666667	1.0183	IM+PG up vs IM
mmu-mir-3109	0.666667	1.0183	IM+PG up vs IM
mmu-mir-5132	0.666667	1.0183	IM+PG up vs IM
mmu-miR-6952-3p	0.666667	1.0183	IM+PG up vs IM
mmu-miR-6989-3p	0.666667	1.0183	IM+PG up vs IM
mmu-miR-195b	0.666667	1.0183	IM+PG up vs IM
mmu-miR-5121	0.666667	1.0183	IM+PG up vs IM
mmu-miR-7669-3p	0.666667	1.0183	IM+PG up vs IM
mmu-mir-5107	0.666667	1.0183	IM+PG up vs IM
mmu-miR-686	0.666667	1.0183	IM+PG up vs IM
mmu-mir-7051	0.666667	1.0183	IM+PG up vs IM
mmu-mir-291a	0.666667	1.0183	IM+PG up vs IM
mmu-mir-291a	0.666667	1.0183	IM+PG up vs IM
mmu-let-7c-2	0.666667	1.0183	IM+PG up vs IM
mmu-let-7c-2	0.666667	1.0183	IM+PG up vs IM
mmu-mir-877	0.666667	1.0183	IM+PG up vs IM
mmu-mir-6912	0.666667	1.0183	IM+PG up vs IM
mmu-mir-7093	0.666667	1.0183	IM+PG up vs IM
mmu-mir-692-1	0.958251	1.0183	IM+PG up vs IM
mmu-miR-683	0.666667	1.0183	IM+PG up vs IM
mmu-miR-7232-3p	0.666667	1.0183	IM+PG up vs IM
mmu-miR-7000-3p	0.666667	1.0183	IM+PG up vs IM
mmu-miR-7220-5p	0.666667	1.01863	IM+PG up vs IM
mmu-miR-7218-3p	0.977822	1.01875	IM+PG up vs IM
mmu-miR-3074-5p	0.96436	1.01885	IM+PG up vs IM
mmu-mir-125a	0.95555	1.01922	IM+PG up vs IM
mmu-miR-6394	0.666667	1.01947	IM+PG up vs IM
mmu-miR-1982-3p	0.666667	1.01947	IM+PG up vs IM
mmu-mir-6352	0.666667	1.01947	IM+PG up vs IM
mmu-miR-702-3p	0.666667	1.01947	IM+PG up vs IM
mmu-mir-6956	0.666667	1.01947	IM+PG up vs IM
mmu-miR-7041-5p	0.666667	1.01947	IM+PG up vs IM
mmu-mir-3620	0.666667	1.01947	IM+PG up vs IM
mmu-miR-1956	0.666667	1.01947	IM+PG up vs IM
mmu-mir-25	0.666667	1.01947	IM+PG up vs IM
mmu-mir-496b	0.666667	1.01947	IM+PG up vs IM
mmu-miR-6415	0.666667	1.01947	IM+PG up vs IM
mmu-mir-7216	0.666667	1.01947	IM+PG up vs IM

mmu-miR-3073b-5p	0.666667	1.01947	IM+PG up vs IM
mmu-mir-6920	0.666667	1.01947	IM+PG up vs IM
mmu-miR-5116	0.666667	1.01947	IM+PG up vs IM
mmu-mir-6958	0.666667	1.01947	IM+PG up vs IM
mmu-mir-471	0.666667	1.01947	IM+PG up vs IM
mmu-mir-6378	0.666667	1.01947	IM+PG up vs IM
mmu-mir-7009	0.666667	1.01947	IM+PG up vs IM
mmu-miR-7214-3p	0.666667	1.01947	IM+PG up vs IM
mmu-miR-21a-5p	0.95914	1.01952	IM+PG up vs IM
mmu-mir-700	0	1.01955	IM+PG up vs IM
mmu-miR-6902-3p	0.924182	1.01963	IM+PG up vs IM
mmu-miR-19b-2-5p	0.92765	1.02027	IM+PG up vs IM
mmu-miR-7044-3p	0	1.02039	IM+PG up vs IM
mmu-mir-6942	0.942423	1.02254	IM+PG up vs IM
mmu-miR-6974-3p	0.942423	1.02254	IM+PG up vs IM
mmu-miR-495-5p	0.952589	1.02254	IM+PG up vs IM
mmu-mir-669h	0	1.02301	IM+PG up vs IM
mmu-mir-1946b	0	1.02301	IM+PG up vs IM
mmu-mir-3112	0	1.02301	IM+PG up vs IM
mmu-mir-5118	0	1.02301	IM+PG up vs IM
mmu-miR-99a-3p	0	1.02301	IM+PG up vs IM
mmu-miR-466l-5p	0	1.02301	IM+PG up vs IM
mmu-mir-376b	0	1.02378	IM+PG up vs IM
mmu-mir-759	0	1.02378	IM+PG up vs IM
mmu-miR-298-3p	0	1.02378	IM+PG up vs IM
mmu-miR-6992-5p	0	1.02378	IM+PG up vs IM
mmu-mir-1224	0	1.02378	IM+PG up vs IM
mmu-mir-466d	0	1.02378	IM+PG up vs IM
mmu-mir-3470b	0	1.02378	IM+PG up vs IM
mmu-mir-21c	0	1.02378	IM+PG up vs IM
mmu-mir-6907	0	1.02378	IM+PG up vs IM
mmu-mir-7006	0	1.02378	IM+PG up vs IM
mmu-mir-8097	0	1.02378	IM+PG up vs IM
mmu-miR-152-3p	0	1.02378	IM+PG up vs IM
mmu-miR-148b-3p	0	1.02378	IM+PG up vs IM
mmu-miR-196a-2-3p	0	1.02378	IM+PG up vs IM
mmu-miR-337-5p	0	1.02378	IM+PG up vs IM
mmu-miR-1899	0	1.02378	IM+PG up vs IM
mmu-miR-1929-5p	0	1.02378	IM+PG up vs IM
mmu-miR-1958	0	1.02378	IM+PG up vs IM
mmu-miR-3099-5p	0	1.02378	IM+PG up vs IM
mmu-miR-146a-3p	0	1.02378	IM+PG up vs IM
mmu-miR-185-3p	0	1.02378	IM+PG up vs IM

mmu-miR-103-1-5p	0	1.02378	IM+PG up vs IM
mmu-miR-302d-5p	0	1.02378	IM+PG up vs IM
mmu-miR-6540-3p	0	1.02378	IM+PG up vs IM
mmu-miR-6907-3p	0	1.02378	IM+PG up vs IM
mmu-miR-7037-3p	0	1.02378	IM+PG up vs IM
mmu-miR-7216-3p	0	1.02378	IM+PG up vs IM
mmu-mir-24-1	0	1.02378	IM+PG up vs IM
mmu-mir-30d	0	1.02378	IM+PG up vs IM
mmu-mir-1983	0	1.02378	IM+PG up vs IM
mmu-mir-133c	0	1.02378	IM+PG up vs IM
mmu-mir-7681	0	1.02378	IM+PG up vs IM
mmu-miR-451a	0	1.02378	IM+PG up vs IM
mmu-miR-708-3p	0	1.02378	IM+PG up vs IM
mmu-miR-455-3p	0	1.02378	IM+PG up vs IM
mmu-miR-3103-3p	0	1.02378	IM+PG up vs IM
mmu-miR-122-3p	0	1.02378	IM+PG up vs IM
mmu-miR-6957-5p	0	1.02378	IM+PG up vs IM
mmu-miR-6965-5p	0	1.02378	IM+PG up vs IM
mmu-miR-8094	0	1.02378	IM+PG up vs IM
mmu-miR-382-5p	0.957006	1.02385	IM+PG up vs IM
mmu-mir-3107	0.93897	1.02419	IM+PG up vs IM
mmu-miR-7041-3p	0.916983	1.02423	IM+PG up vs IM
mmu-miR-7661-3p	0.779372	1.02496	IM+PG up vs IM
mmu-miR-7049-5p	0.779372	1.02496	IM+PG up vs IM
mmu-mir-7088	0	1.02547	IM+PG up vs IM
mmu-miR-30b-5p	0	1.02547	IM+PG up vs IM
mmu-mir-327	0.943364	1.02616	IM+PG up vs IM
mmu-miR-7654-5p	0.942436	1.02678	IM+PG up vs IM
mmu-miR-6516-5p	0.942436	1.02678	IM+PG up vs IM
mmu-miR-382-3p	0.942436	1.02678	IM+PG up vs IM
mmu-mir-378b	0.666667	1.02715	IM+PG up vs IM
mmu-miR-22-3p	0.944047	1.02737	IM+PG up vs IM
mmu-miR-467b-3p	0.932479	1.02741	IM+PG up vs IM
mmu-miR-1960	0.855996	1.02862	IM+PG up vs IM
mmu-miR-491-3p	0.966493	1.02865	IM+PG up vs IM
mmu-mir-465d	0.928565	1.02943	IM+PG up vs IM
mmu-mir-141	0	1.03105	IM+PG up vs IM
mmu-mir-15a	0	1.03105	IM+PG up vs IM
mmu-mir-29c	0	1.03105	IM+PG up vs IM
mmu-mir-107	0	1.03105	IM+PG up vs IM
mmu-mir-6238	0	1.03105	IM+PG up vs IM
mmu-mir-7224	0	1.03105	IM+PG up vs IM
mmu-miR-154-5p	0	1.03105	IM+PG up vs IM

mmu-miR-449c-5p	0	1.03105	IM+PG up vs IM
mmu-miR-743a-3p	0	1.03105	IM+PG up vs IM
mmu-miR-6541	0	1.03105	IM+PG up vs IM
mmu-mir-129-1	0	1.03105	IM+PG up vs IM
mmu-mir-129-1	0	1.03105	IM+PG up vs IM
mmu-mir-30c-1	0	1.03105	IM+PG up vs IM
mmu-mir-323	0	1.03105	IM+PG up vs IM
mmu-mir-673	0	1.03105	IM+PG up vs IM
mmu-mir-711	0	1.03105	IM+PG up vs IM
mmu-mir-3070a	0	1.03105	IM+PG up vs IM
mmu-mir-5101	0	1.03105	IM+PG up vs IM
mmu-mir-6351	0	1.03105	IM+PG up vs IM
mmu-mir-6364	0	1.03105	IM+PG up vs IM
mmu-mir-6916	0	1.03105	IM+PG up vs IM
mmu-miR-30e-3p	0	1.03105	IM+PG up vs IM
mmu-miR-192-5p	0	1.03105	IM+PG up vs IM
mmu-miR-328-3p	0	1.03105	IM+PG up vs IM
mmu-miR-99b-3p	0	1.03105	IM+PG up vs IM
mmu-miR-124-5p	0	1.03105	IM+PG up vs IM
mmu-miR-183-3p	0	1.03105	IM+PG up vs IM
mmu-miR-331-5p	0	1.03105	IM+PG up vs IM
mmu-miR-125b-1-3p	0	1.03105	IM+PG up vs IM
mmu-miR-876-3p	0	1.03105	IM+PG up vs IM
mmu-miR-1903	0	1.03105	IM+PG up vs IM
mmu-miR-3088-3p	0	1.03105	IM+PG up vs IM
mmu-miR-201-3p	0	1.03105	IM+PG up vs IM
mmu-miR-208a-5p	0	1.03105	IM+PG up vs IM
mmu-miR-25-5p	0	1.03105	IM+PG up vs IM
mmu-miR-19b-1-5p	0	1.03105	IM+PG up vs IM
mmu-miR-551b-5p	0	1.03105	IM+PG up vs IM
mmu-miR-5619-5p	0	1.03105	IM+PG up vs IM
mmu-miR-6338	0	1.03105	IM+PG up vs IM
mmu-miR-6344	0	1.03105	IM+PG up vs IM
mmu-miR-6355	0	1.03105	IM+PG up vs IM
mmu-miR-130c	0	1.03105	IM+PG up vs IM
mmu-miR-6392-3p	0	1.03105	IM+PG up vs IM
mmu-miR-7006-3p	0	1.03105	IM+PG up vs IM
mmu-miR-7014-5p	0	1.03105	IM+PG up vs IM
mmu-miR-7063-3p	0	1.03105	IM+PG up vs IM
mmu-miR-7075-3p	0	1.03105	IM+PG up vs IM
mmu-miR-7223-3p	0	1.03105	IM+PG up vs IM
mmu-miR-7243-5p	0	1.03105	IM+PG up vs IM
mmu-miR-497b	0	1.03105	IM+PG up vs IM

mmu-mir-193a	0	1.03105	IM+PG up vs IM
mmu-mir-129-2	0	1.03105	IM+PG up vs IM
mmu-mir-687	0	1.03105	IM+PG up vs IM
mmu-mir-702	0	1.03105	IM+PG up vs IM
mmu-mir-421	0	1.03105	IM+PG up vs IM
mmu-mir-1902	0	1.03105	IM+PG up vs IM
mmu-mir-669l	0	1.03105	IM+PG up vs IM
mmu-mir-3060	0	1.03105	IM+PG up vs IM
mmu-mir-3066	0	1.03105	IM+PG up vs IM
mmu-mir-3471-1	0	1.03105	IM+PG up vs IM
mmu-mir-5128	0	1.03105	IM+PG up vs IM
mmu-mir-6354	0	1.03105	IM+PG up vs IM
mmu-mir-6370	0	1.03105	IM+PG up vs IM
mmu-mir-6407	0	1.03105	IM+PG up vs IM
mmu-mir-6410	0	1.03105	IM+PG up vs IM
mmu-mir-6937	0	1.03105	IM+PG up vs IM
mmu-mir-7054	0	1.03105	IM+PG up vs IM
mmu-mir-7215	0	1.03105	IM+PG up vs IM
mmu-mir-7669	0	1.03105	IM+PG up vs IM
mmu-mir-8098	0	1.03105	IM+PG up vs IM
mmu-miR-138-5p	0	1.03105	IM+PG up vs IM
mmu-miR-377-3p	0	1.03105	IM+PG up vs IM
mmu-miR-302b-3p	0	1.03105	IM+PG up vs IM
mmu-miR-712-5p	0	1.03105	IM+PG up vs IM
mmu-miR-27b-5p	0	1.03105	IM+PG up vs IM
mmu-miR-340-5p	0	1.03105	IM+PG up vs IM
mmu-miR-335-3p	0	1.03105	IM+PG up vs IM
mmu-miR-466h-5p	0	1.03105	IM+PG up vs IM
mmu-miR-467b-5p	0	1.03105	IM+PG up vs IM
mmu-miR-3068-5p	0	1.03105	IM+PG up vs IM
mmu-miR-3081-3p	0	1.03105	IM+PG up vs IM
mmu-miR-3093-5p	0	1.03105	IM+PG up vs IM
mmu-miR-152-5p	0	1.03105	IM+PG up vs IM
mmu-miR-192-3p	0	1.03105	IM+PG up vs IM
mmu-miR-6346	0	1.03105	IM+PG up vs IM
mmu-miR-6391	0	1.03105	IM+PG up vs IM
mmu-miR-6898-5p	0	1.03105	IM+PG up vs IM
mmu-miR-6921-3p	0	1.03105	IM+PG up vs IM
mmu-miR-6973a-5p	0	1.03105	IM+PG up vs IM
mmu-miR-6998-5p	0	1.03105	IM+PG up vs IM
mmu-miR-6769b-3p	0	1.03105	IM+PG up vs IM
mmu-miR-7090-5p	0	1.03105	IM+PG up vs IM
mmu-miR-7219-3p	0	1.03105	IM+PG up vs IM

mmu-mir-29a	0.00495386	1.03119	IM+PG up vs IM
mmu-miR-7078-5p	0.929624	1.03159	IM+PG up vs IM
mmu-miR-7233-5p	0.747976	1.03192	IM+PG up vs IM
mmu-mir-92a-1	0.929074	1.03231	IM+PG up vs IM
mmu-mir-466j	0.0502059	1.03256	IM+PG up vs IM
mmu-miR-6922-3p	0.0502065	1.03256	IM+PG up vs IM
mmu-mir-3106	0.0502065	1.03256	IM+PG up vs IM
mmu-mir-3083	0.0502065	1.03256	IM+PG up vs IM
mmu-mir-878	0.0502065	1.03256	IM+PG up vs IM
mmu-mir-7000	0.0502065	1.03256	IM+PG up vs IM
mmu-miR-292b-3p	0.854693	1.03302	IM+PG up vs IM
mmu-miR-3059-5p	0.854693	1.03302	IM+PG up vs IM
mmu-mir-467h	0.866252	1.03334	IM+PG up vs IM
mmu-mir-9-2	0	1.03407	IM+PG up vs IM
mmu-mir-135a-1	0	1.03407	IM+PG up vs IM
mmu-mir-325	0	1.03407	IM+PG up vs IM
mmu-mir-329	0	1.03407	IM+PG up vs IM
mmu-mir-199a-2	0	1.03407	IM+PG up vs IM
mmu-mir-384	0	1.03407	IM+PG up vs IM
mmu-mir-694	0	1.03407	IM+PG up vs IM
mmu-mir-879	0	1.03407	IM+PG up vs IM
mmu-mir-18b	0	1.03407	IM+PG up vs IM
mmu-mir-568	0	1.03407	IM+PG up vs IM
mmu-mir-872	0	1.03407	IM+PG up vs IM
mmu-mir-1899	0	1.03407	IM+PG up vs IM
mmu-mir-1967	0	1.03407	IM+PG up vs IM
mmu-mir-432	0	1.03407	IM+PG up vs IM
mmu-mir-3105	0	1.03407	IM+PG up vs IM
mmu-mir-3962	0	1.03407	IM+PG up vs IM
mmu-mir-344h-1	0	1.03407	IM+PG up vs IM
mmu-mir-344h-2	0	1.03407	IM+PG up vs IM
mmu-mir-7085	0	1.03407	IM+PG up vs IM
mmu-mir-6715	0	1.03407	IM+PG up vs IM
mmu-mir-8096	0	1.03407	IM+PG up vs IM
mmu-mir-3535	0	1.03407	IM+PG up vs IM
mmu-miR-323-3p	0	1.03407	IM+PG up vs IM
mmu-miR-324-3p	0	1.03407	IM+PG up vs IM
mmu-miR-330-3p	0	1.03407	IM+PG up vs IM
mmu-miR-653-5p	0	1.03407	IM+PG up vs IM
mmu-miR-664-3p	0	1.03407	IM+PG up vs IM
mmu-miR-669p-3p	0	1.03407	IM+PG up vs IM
mmu-miR-205-3p	0	1.03407	IM+PG up vs IM
mmu-miR-212-5p	0	1.03407	IM+PG up vs IM

mmu-miR-3961	0	1.03407	IM+PG up vs IM
mmu-miR-6948-3p	0	1.03407	IM+PG up vs IM
mmu-miR-6963-5p	0	1.03407	IM+PG up vs IM
mmu-miR-6981-3p	0	1.03407	IM+PG up vs IM
mmu-miR-6995-3p	0	1.03407	IM+PG up vs IM
mmu-miR-7005-3p	0	1.03407	IM+PG up vs IM
mmu-miR-7210-5p	0	1.03407	IM+PG up vs IM
mmu-miR-8092	0	1.03407	IM+PG up vs IM
mmu-mir-7657	0.919032	1.03447	IM+PG up vs IM
mmu-mir-669m-1	0.5944	1.03453	IM+PG up vs IM
mmu-miR-674-3p	0.932649	1.03491	IM+PG up vs IM
mmu-miR-292-5p	0.857893	1.03491	IM+PG up vs IM
mmu-miR-6996-3p	0.91829	1.03503	IM+PG up vs IM
mmu-miR-666-3p	0.0438049	1.0355	IM+PG up vs IM
mmu-miR-3058-3p	0.0438049	1.0355	IM+PG up vs IM
mmu-miR-6906-3p	0.927541	1.0355	IM+PG up vs IM
mmu-mir-7082	0.927541	1.0355	IM+PG up vs IM
mmu-mir-299b	0.0460235	1.03558	IM+PG up vs IM
mmu-miR-1b-3p	0.461086	1.03681	IM+PG up vs IM
mmu-mir-136	0	1.03694	IM+PG up vs IM
mmu-mir-151	0	1.03694	IM+PG up vs IM
mmu-mir-208a	0	1.03694	IM+PG up vs IM
mmu-mir-5130	0	1.03694	IM+PG up vs IM
mmu-mir-6361	0	1.03694	IM+PG up vs IM
mmu-mir-6388	0	1.03694	IM+PG up vs IM
mmu-miR-141-5p	0	1.03694	IM+PG up vs IM
mmu-miR-5618-5p	0	1.03694	IM+PG up vs IM
mmu-miR-6916-3p	0	1.03694	IM+PG up vs IM
mmu-miR-6937-3p	0	1.03694	IM+PG up vs IM
mmu-miR-6972-5p	0.666667	1.03699	IM+PG up vs IM
mmu-mir-344c	0.914328	1.03732	IM+PG up vs IM
mmu-mir-7050	0.914328	1.03732	IM+PG up vs IM
mmu-miR-450a-1-3p	0.423871	1.03763	IM+PG up vs IM
mmu-miR-3106-3p	0.924042	1.03788	IM+PG up vs IM
mmu-mir-124-3	0.315676	1.03813	IM+PG up vs IM
mmu-mir-124-1	0.315676	1.03813	IM+PG up vs IM
mmu-mir-124-2	0.315676	1.03813	IM+PG up vs IM
mmu-miR-6928-3p	0.91268	1.03829	IM+PG up vs IM
mmu-miR-490-3p	0.91268	1.03829	IM+PG up vs IM
mmu-miR-144-3p	0.91268	1.03829	IM+PG up vs IM
mmu-miR-374c-3p	0.818305	1.0389	IM+PG up vs IM
mmu-mir-143	0.818305	1.0389	IM+PG up vs IM
mmu-mir-98	0.818305	1.0389	IM+PG up vs IM

mmu-mir-1928	0.818305	1.0389	IM+PG up vs IM
mmu-miR-6903-5p	0.818305	1.0389	IM+PG up vs IM
mmu-mir-6481	0.818305	1.0389	IM+PG up vs IM
mmu-miR-7224-5p	0.818305	1.0389	IM+PG up vs IM
mmu-mir-299a	0	1.03931	IM+PG up vs IM
mmu-mir-8099-1	0	1.03931	IM+PG up vs IM
mmu-mir-8099-2	0	1.03931	IM+PG up vs IM
mmu-miR-695	0	1.03931	IM+PG up vs IM
mmu-miR-872-5p	0	1.03931	IM+PG up vs IM
mmu-miR-5110	0	1.03931	IM+PG up vs IM
mmu-miR-6418-5p	0	1.03931	IM+PG up vs IM
mmu-miR-6930-3p	0	1.03931	IM+PG up vs IM
mmu-miR-467h	0.219897	1.03935	IM+PG up vs IM
mmu-miR-1947-3p	0.220444	1.03938	IM+PG up vs IM
mmu-miR-6956-3p	0.381343	1.03955	IM+PG up vs IM
mmu-miR-6379	0.706232	1.04021	IM+PG up vs IM
mmu-miR-425-3p	0.388715	1.04022	IM+PG up vs IM
mmu-miR-710	0.827428	1.0403	IM+PG up vs IM
mmu-miR-344b-3p	0.827428	1.0403	IM+PG up vs IM
mmu-miR-871-3p	0.827428	1.0403	IM+PG up vs IM
mmu-miR-654-5p	0.955572	1.04032	IM+PG up vs IM
mmu-miR-6715-3p	0.914026	1.04103	IM+PG up vs IM
mmu-miR-30a-3p	0.914026	1.04103	IM+PG up vs IM
mmu-miR-6915-5p	0.914026	1.04103	IM+PG up vs IM
mmu-miR-1904	0.3976	1.04107	IM+PG up vs IM
mmu-miR-6400	0.3976	1.04107	IM+PG up vs IM
mmu-miR-6362	0.3976	1.04107	IM+PG up vs IM
mmu-let-7g	0.835944	1.04137	IM+PG up vs IM
mmu-mir-423	0.913223	1.04155	IM+PG up vs IM
mmu-mir-3058	0.420115	1.04207	IM+PG up vs IM
mmu-miR-7058-3p	0.4115	1.04252	IM+PG up vs IM
mmu-miR-691	0.4115	1.04252	IM+PG up vs IM
mmu-miR-708-5p	0.916726	1.04259	IM+PG up vs IM
mmu-miR-669l-5p	0.414557	1.04286	IM+PG up vs IM
mmu-miR-7065-3p	0.414557	1.04286	IM+PG up vs IM
mmu-mir-6900	0.414557	1.04286	IM+PG up vs IM
mmu-mir-7084	0.906489	1.044	IM+PG up vs IM
mmu-miR-3086-5p	0.666667	1.04424	IM+PG up vs IM
mmu-miR-6348	0.901239	1.0443	IM+PG up vs IM
mmu-miR-370-3p	0.819489	1.04434	IM+PG up vs IM
mmu-miR-546	0.903853	1.04502	IM+PG up vs IM
mmu-mir-6408	0.901462	1.04529	IM+PG up vs IM
mmu-miR-673-3p	0.353399	1.0455	IM+PG up vs IM

mmu-miR-344d-3p	0.899149	1.04681	IM+PG up vs IM
mmu-mir-1957b	0.869025	1.04719	IM+PG up vs IM
mmu-miR-181b-1-3p	0.869025	1.04719	IM+PG up vs IM
mmu-miR-7057-5p	0.80232	1.04729	IM+PG up vs IM
mmu-mir-184	0.813703	1.04756	IM+PG up vs IM
mmu-miR-1943-5p	0.903465	1.04816	IM+PG up vs IM
mmu-miR-760-5p	0.903465	1.04816	IM+PG up vs IM
mmu-miR-693-5p	0.3501	1.04846	IM+PG up vs IM
mmu-miR-7117-5p	0.3501	1.04846	IM+PG up vs IM
mmu-mir-6367	0.3501	1.04846	IM+PG up vs IM
mmu-mir-466j	0.3501	1.04846	IM+PG up vs IM
mmu-mir-6387	0.413339	1.04977	IM+PG up vs IM
mmu-miR-701-3p	0.364646	1.04992	IM+PG up vs IM
mmu-mir-7664	0.364646	1.04992	IM+PG up vs IM
mmu-mir-3968	0.364646	1.04992	IM+PG up vs IM
mmu-miR-3473g	0.364646	1.04992	IM+PG up vs IM
mmu-miR-155-3p	0.316567	1.05	IM+PG up vs IM
mmu-miR-764-5p	0.316567	1.05	IM+PG up vs IM
mmu-miR-3079-5p	0.449492	1.05024	IM+PG up vs IM
mmu-miR-148a-5p	0.666667	1.05075	IM+PG up vs IM
mmu-miR-493-3p	0.666667	1.05075	IM+PG up vs IM
mmu-miR-7045-3p	0.666667	1.05075	IM+PG up vs IM
mmu-miR-7657-3p	0.666667	1.05075	IM+PG up vs IM
mmu-mir-425	0.893085	1.05097	IM+PG up vs IM
mmu-miR-1839-5p	0.893085	1.05097	IM+PG up vs IM
mmu-miR-142-3p	0.375712	1.05112	IM+PG up vs IM
mmu-mir-1903	0.375712	1.05112	IM+PG up vs IM
mmu-mir-292b	0.375712	1.05112	IM+PG up vs IM
mmu-miR-1188-5p	0.88863	1.05141	IM+PG up vs IM
mmu-mir-763	0.861913	1.05157	IM+PG up vs IM
mmu-miR-7242-5p	0.388825	1.05266	IM+PG up vs IM
mmu-mir-3088	0.347955	1.05299	IM+PG up vs IM
mmu-mir-744	0.870323	1.05321	IM+PG up vs IM
mmu-miR-129-1-3p	0.393425	1.05323	IM+PG up vs IM
mmu-mir-7213	0.393425	1.05323	IM+PG up vs IM
mmu-mir-6336	0.393652	1.05326	IM+PG up vs IM
mmu-mir-3101	0.879158	1.05348	IM+PG up vs IM
mmu-miR-8093	0.666667	1.05388	IM+PG up vs IM
mmu-mir-1a-1	0.666667	1.05388	IM+PG up vs IM
mmu-mir-466l	0.666667	1.05388	IM+PG up vs IM
mmu-miR-500-3p	0.886926	1.05405	IM+PG up vs IM
mmu-mir-6971	0.886883	1.05419	IM+PG up vs IM
mmu-miR-6946-5p	0.359127	1.0542	IM+PG up vs IM

mmu-mir-7683	0.318711	1.05446	IM+PG up vs IM
mmu-miR-18a-3p	0.318711	1.05446	IM+PG up vs IM
mmu-mir-7656	0.849573	1.05464	IM+PG up vs IM
mmu-miR-7677-3p	0.364199	1.05477	IM+PG up vs IM
mmu-miR-7666-3p	0.886792	1.0548	IM+PG up vs IM
mmu-mir-141	0	1.05613	IM+PG up vs IM
mmu-mir-192	0	1.05613	IM+PG up vs IM
mmu-mir-1946a	0	1.05613	IM+PG up vs IM
mmu-mir-3097	0	1.05613	IM+PG up vs IM
mmu-mir-8102	0	1.05613	IM+PG up vs IM
mmu-miR-1968-5p	0	1.05613	IM+PG up vs IM
mmu-miR-3057-5p	0	1.05613	IM+PG up vs IM
mmu-miR-3074-1-3p	0	1.05613	IM+PG up vs IM
mmu-miR-130a-5p	0	1.05613	IM+PG up vs IM
mmu-miR-365-2-5p	0	1.05613	IM+PG up vs IM
mmu-miR-6913-3p	0	1.05613	IM+PG up vs IM
mmu-miR-7022-5p	0	1.05613	IM+PG up vs IM
mmu-miR-219b-5p	0	1.05613	IM+PG up vs IM
mmu-mir-6913	0.30754	1.05687	IM+PG up vs IM
mmu-miR-6335	0.770246	1.05698	IM+PG up vs IM
mmu-miR-7233-3p	0.880292	1.05729	IM+PG up vs IM
mmu-miR-1894-3p	0.939508	1.05747	IM+PG up vs IM
mmu-miR-8114	0.736822	1.05754	IM+PG up vs IM
mmu-mir-708	0.425335	1.05778	IM+PG up vs IM
mmu-miR-1941-5p	0.666667	1.05826	IM+PG up vs IM
mmu-miR-669e-5p	0.666667	1.05826	IM+PG up vs IM
mmu-mir-344i	0.666667	1.05826	IM+PG up vs IM
mmu-mir-344i	0.666667	1.05826	IM+PG up vs IM
mmu-miR-384-3p	0.666667	1.05826	IM+PG up vs IM
mmu-mir-7661	0.666667	1.05826	IM+PG up vs IM
mmu-miR-6341	0.666667	1.05826	IM+PG up vs IM
mmu-mir-7034	0.666667	1.05826	IM+PG up vs IM
mmu-miR-679-5p	0.666667	1.05826	IM+PG up vs IM
mmu-mir-882	0.666667	1.05826	IM+PG up vs IM
mmu-mir-7060	0.666667	1.05826	IM+PG up vs IM
mmu-miR-6997-5p	0.666667	1.05826	IM+PG up vs IM
mmu-mir-701	0.785975	1.05826	IM+PG up vs IM
mmu-miR-434-3p	0.882907	1.0585	IM+PG up vs IM
mmu-mir-5129	0.805723	1.05966	IM+PG up vs IM
mmu-miR-712-3p	0.876096	1.06026	IM+PG up vs IM
mmu-miR-6964-3p	0.512113	1.06047	IM+PG up vs IM
mmu-miR-452-5p	0.468108	1.06161	IM+PG up vs IM
mmu-mir-182	0	1.06229	IM+PG up vs IM

mmu-mir-101a	0	1.06229	IM+PG up vs IM
mmu-mir-3070a	0	1.06229	IM+PG up vs IM
mmu-mir-6373	0	1.06229	IM+PG up vs IM
mmu-mir-7057	0	1.06229	IM+PG up vs IM
mmu-mir-7062	0	1.06229	IM+PG up vs IM
mmu-mir-7652	0	1.06229	IM+PG up vs IM
mmu-miR-298-5p	0	1.06229	IM+PG up vs IM
mmu-miR-100-5p	0	1.06229	IM+PG up vs IM
mmu-miR-673-5p	0	1.06229	IM+PG up vs IM
mmu-miR-154-3p	0	1.06229	IM+PG up vs IM
mmu-miR-7b-3p	0	1.06229	IM+PG up vs IM
mmu-miR-3544-3p	0	1.06229	IM+PG up vs IM
mmu-miR-5617-3p	0	1.06229	IM+PG up vs IM
mmu-miR-6395	0	1.06229	IM+PG up vs IM
mmu-miR-6959-3p	0	1.06229	IM+PG up vs IM
mmu-mir-654	0.518072	1.06229	IM+PG up vs IM
mmu-mir-3073b	0	1.06229	IM+PG up vs IM
mmu-mir-6933	0	1.06229	IM+PG up vs IM
mmu-miR-379-5p	0	1.06229	IM+PG up vs IM
mmu-miR-431-3p	0	1.06229	IM+PG up vs IM
mmu-miR-6363	0	1.06229	IM+PG up vs IM
mmu-miR-384-5p	0.666667	1.06268	IM+PG up vs IM
mmu-miR-466m-3p	0.666667	1.06268	IM+PG up vs IM
mmu-miR-3083-3p	0.666667	1.06268	IM+PG up vs IM
mmu-miR-3077-3p	0.666667	1.06268	IM+PG up vs IM
mmu-mir-33	0.666667	1.06268	IM+PG up vs IM
mmu-miR-6988-5p	0.666667	1.06268	IM+PG up vs IM
mmu-mir-6239	0.666667	1.06268	IM+PG up vs IM
mmu-miR-6903-3p	0.666667	1.06268	IM+PG up vs IM
mmu-mir-27b	0.666667	1.06268	IM+PG up vs IM
mmu-mir-27b	0.666667	1.06268	IM+PG up vs IM
mmu-mir-380	0.666667	1.06268	IM+PG up vs IM
mmu-mir-344-1	0.791597	1.06268	IM+PG up vs IM
mmu-mir-1291	0.713365	1.06358	IM+PG up vs IM
mmu-miR-144-5p	0.0257283	1.06462	IM+PG up vs IM
mmu-mir-511	0.771551	1.06469	IM+PG up vs IM
mmu-miR-5621-3p	0.799238	1.06471	IM+PG up vs IM
mmu-miR-5107-3p	0.297808	1.06535	IM+PG up vs IM
mmu-mir-3089	0.777021	1.06569	IM+PG up vs IM
mmu-miR-25-3p	0.666667	1.0668	IM+PG up vs IM
mmu-miR-882	0.666667	1.0668	IM+PG up vs IM
mmu-miR-6357	0.666667	1.0668	IM+PG up vs IM
mmu-mir-691	0.666667	1.0668	IM+PG up vs IM

mmu-mir-6945	0.666667	1.0668	IM+PG up vs IM
mmu-miR-383-3p	0.666667	1.0668	IM+PG up vs IM
mmu-mir-6399	0.666667	1.0668	IM+PG up vs IM
mmu-miR-5615-5p	0.666667	1.0668	IM+PG up vs IM
mmu-miR-6920-5p	0.666667	1.0668	IM+PG up vs IM
mmu-mir-6404	0.666667	1.0668	IM+PG up vs IM
mmu-miR-3100-3p	0.666667	1.0668	IM+PG up vs IM
mmu-mir-6409	0.666667	1.0668	IM+PG up vs IM
mmu-miR-6897-5p	0.666667	1.0668	IM+PG up vs IM
mmu-miR-7652-5p	0.666667	1.0668	IM+PG up vs IM
mmu-mir-296	0.531513	1.06698	IM+PG up vs IM
mmu-mir-24-1	0.715332	1.06805	IM+PG up vs IM
mmu-miR-465a-5p	0.817394	1.06807	IM+PG up vs IM
mmu-miR-129b-5p	0.763455	1.06839	IM+PG up vs IM
mmu-let-7f-2-3p	0.763455	1.06839	IM+PG up vs IM
mmu-miR-21c	0.424493	1.06875	IM+PG up vs IM
mmu-miR-1967	0.805511	1.06884	IM+PG up vs IM
mmu-miR-6416-5p	0	1.0693	IM+PG up vs IM
mmu-miR-3084-3p	0.481455	1.06946	IM+PG up vs IM
mmu-mir-211	0.873076	1.06975	IM+PG up vs IM
mmu-mir-150	0.430768	1.06998	IM+PG up vs IM
mmu-miR-7671-5p	0.666667	1.07009	IM+PG up vs IM
mmu-mir-3113	0.666667	1.07009	IM+PG up vs IM
mmu-miR-28c	0.666667	1.07009	IM+PG up vs IM
mmu-mir-1198	0.666667	1.07009	IM+PG up vs IM
mmu-mir-194-2	0.666667	1.07009	IM+PG up vs IM
mmu-miR-374b-5p	0.666667	1.07009	IM+PG up vs IM
mmu-miR-541-5p	0.800512	1.07009	IM+PG up vs IM
mmu-miR-1912-5p	0.808911	1.07077	IM+PG up vs IM
mmu-miR-5623-5p	0.666667	1.0708	IM+PG up vs IM
mmu-mir-7075	0.69047	1.07115	IM+PG up vs IM
mmu-miR-7237-5p	0.488702	1.07149	IM+PG up vs IM
mmu-mir-5709	0	1.07158	IM+PG up vs IM
mmu-miR-7673-5p	0	1.07158	IM+PG up vs IM
mmu-miR-6352	0.14212	1.07201	IM+PG up vs IM
mmu-mir-6992	0.728804	1.07229	IM+PG up vs IM
mmu-miR-466f-5p	0.230536	1.0724	IM+PG up vs IM
mmu-mir-204	0.716656	1.07269	IM+PG up vs IM
mmu-miR-5627-3p	0.769597	1.07287	IM+PG up vs IM
mmu-miR-7090-3p	0.769597	1.07287	IM+PG up vs IM
mmu-mir-6985	0.787803	1.07498	IM+PG up vs IM
mmu-miR-7649-3p	0.75683	1.07559	IM+PG up vs IM
mmu-miR-142-5p	0.666667	1.07567	IM+PG up vs IM

mmu-miR-327	0.262613	1.07581	IM+PG up vs IM
mmu-mir-15b	0	1.07589	IM+PG up vs IM
mmu-mir-23a	0	1.07589	IM+PG up vs IM
mmu-mir-652	0	1.07589	IM+PG up vs IM
mmu-mir-6908	0	1.07589	IM+PG up vs IM
mmu-miR-208b-3p	0	1.07589	IM+PG up vs IM
mmu-mir-34a	0	1.07589	IM+PG up vs IM
mmu-mir-1960	0	1.07589	IM+PG up vs IM
mmu-mir-5620	0	1.07589	IM+PG up vs IM
mmu-mir-6984	0	1.07589	IM+PG up vs IM
mmu-miR-7057-3p	0	1.07589	IM+PG up vs IM
mmu-miR-147-5p	0.78679	1.07599	IM+PG up vs IM
mmu-miR-1191	0.78679	1.07599	IM+PG up vs IM
mmu-miR-466n-3p	0.726527	1.07763	IM+PG up vs IM
mmu-miR-7043-5p	0.464664	1.07793	IM+PG up vs IM
mmu-miR-1930-5p	0.666667	1.07798	IM+PG up vs IM
mmu-miR-6947-3p	0.666667	1.07798	IM+PG up vs IM
mmu-mir-5110	0.666667	1.07798	IM+PG up vs IM
mmu-miR-7030-3p	0.666667	1.07798	IM+PG up vs IM
mmu-miR-6957-3p	0.666667	1.07798	IM+PG up vs IM
mmu-miR-466n-5p	0.666667	1.07798	IM+PG up vs IM
mmu-miR-344e-3p	0.666667	1.07798	IM+PG up vs IM
mmu-mir-7074	0.666667	1.07798	IM+PG up vs IM
mmu-miR-196b-3p	0.666667	1.07798	IM+PG up vs IM
mmu-miR-6912-5p	0.666667	1.07798	IM+PG up vs IM
mmu-miR-138-2-3p	0.694905	1.07822	IM+PG up vs IM
mmu-mir-301a	0.214185	1.07865	IM+PG up vs IM
mmu-miR-203-3p	0.764283	1.07871	IM+PG up vs IM
mmu-miR-1946b	0.556754	1.07894	IM+PG up vs IM
mmu-let-7c-1-3p	0.556754	1.07894	IM+PG up vs IM
mmu-mir-3075	0.774276	1.07905	IM+PG up vs IM
mmu-miR-6928-5p	0.688123	1.07981	IM+PG up vs IM
mmu-mir-125b-2	0.666667	1.08021	IM+PG up vs IM
mmu-mir-125b-2	0.666667	1.08021	IM+PG up vs IM
mmu-miR-149-5p	0.666667	1.08021	IM+PG up vs IM
mmu-miR-7a-2-3p	0.836313	1.08093	IM+PG up vs IM
mmu-mir-6371	0.730192	1.08102	IM+PG up vs IM
mmu-mir-7071	0.245177	1.08208	IM+PG up vs IM
mmu-miR-15b-3p	0.856378	1.0821	IM+PG up vs IM
mmu-miR-380-5p	0.761447	1.0821	IM+PG up vs IM
mmu-mir-453	0.47898	1.08213	IM+PG up vs IM
mmu-mir-664	0.863511	1.08233	IM+PG up vs IM
mmu-miR-106a-5p	0.863045	1.08282	IM+PG up vs IM

mmu-miR-7012-5p	0.863045	1.08282	IM+PG up vs IM
mmu-mir-10b	0.252577	1.08297	IM+PG up vs IM
mmu-miR-7659-5p	0.832217	1.08314	IM+PG up vs IM
mmu-miR-6546-3p	0.521729	1.08337	IM+PG up vs IM
mmu-miR-7237-3p	0.563915	1.08342	IM+PG up vs IM
mmu-miR-7092-3p	0.563915	1.08342	IM+PG up vs IM
mmu-let-7a-5p	0.666667	1.08349	IM+PG up vs IM
mmu-miR-875-3p	0.503022	1.08362	IM+PG up vs IM
mmu-miR-1843b-3p	0.666667	1.0838	IM+PG up vs IM
mmu-miR-130b-3p	0.666667	1.0838	IM+PG up vs IM
mmu-mir-124-1	0.666667	1.0838	IM+PG up vs IM
mmu-miR-92a-3p	0.666667	1.08442	IM+PG up vs IM
mmu-miR-7032-5p	0.506109	1.08482	IM+PG up vs IM
mmu-mir-8107	0	1.0852	IM+PG up vs IM
mmu-miR-23b-5p	0	1.0852	IM+PG up vs IM
mmu-miR-7232-5p	0	1.0852	IM+PG up vs IM
mmu-miR-7019-5p	0.666667	1.08555	IM+PG up vs IM
mmu-miR-26a-2-3p	0.666667	1.08555	IM+PG up vs IM
mmu-mir-5615-1	0.666667	1.08555	IM+PG up vs IM
mmu-miR-466f-3p	0.666667	1.08555	IM+PG up vs IM
mmu-mir-7686	0.666667	1.08555	IM+PG up vs IM
mmu-mir-3103	0.666667	1.08555	IM+PG up vs IM
mmu-miR-376b-5p	0.666667	1.08555	IM+PG up vs IM
mmu-miR-6345	0.666667	1.08555	IM+PG up vs IM
mmu-mir-1895	0.666667	1.08555	IM+PG up vs IM
mmu-miR-1901	0.666667	1.08555	IM+PG up vs IM
mmu-miR-7116-3p	0.666667	1.08555	IM+PG up vs IM
mmu-mir-466f-1	0.666667	1.08555	IM+PG up vs IM
mmu-mir-351	0.666667	1.08555	IM+PG up vs IM
mmu-mir-509	0.666667	1.08555	IM+PG up vs IM
mmu-mir-466q	0.666667	1.08555	IM+PG up vs IM
mmu-miR-697	0.666667	1.08555	IM+PG up vs IM
mmu-miR-300-5p	0.666667	1.08555	IM+PG up vs IM
mmu-mir-28b	0.666667	1.08555	IM+PG up vs IM
mmu-miR-6342	0.52867	1.0866	IM+PG up vs IM
mmu-miR-652-5p	0.813196	1.08709	IM+PG up vs IM
mmu-mir-3091	0.573027	1.08713	IM+PG up vs IM
mmu-mir-6918	0.569333	1.08726	IM+PG up vs IM
mmu-miR-1955-3p	0.524066	1.08792	IM+PG up vs IM
mmu-miR-181d-5p	0.570249	1.08795	IM+PG up vs IM
mmu-mir-1668	0.570249	1.08795	IM+PG up vs IM
mmu-miR-3088-5p	0.570249	1.08795	IM+PG up vs IM
mmu-miR-721	0.570249	1.08795	IM+PG up vs IM

mmu-miR-3081-5p	0.570249	1.08795	IM+PG up vs IM
mmu-miR-6936-3p	0.514111	1.08817	IM+PG up vs IM
mmu-mir-718	0.678993	1.08858	IM+PG up vs IM
mmu-mir-6968	0.149782	1.0886	IM+PG up vs IM
mmu-miR-7087-3p	0.781635	1.08968	IM+PG up vs IM
mmu-miR-3089-5p	0.517688	1.08978	IM+PG up vs IM
mmu-miR-7046-5p	0.867783	1.08997	IM+PG up vs IM
mmu-miR-5124b	0.666667	1.09072	IM+PG up vs IM
mmu-miR-126a-3p	0.666667	1.09072	IM+PG up vs IM
mmu-miR-214-3p	0.666667	1.09072	IM+PG up vs IM
mmu-miR-677-3p	0.754783	1.09099	IM+PG up vs IM
mmu-mir-3569	0.754783	1.09099	IM+PG up vs IM
mmu-miR-218-2-3p	0.537321	1.09111	IM+PG up vs IM
mmu-mir-7233	0.537321	1.09111	IM+PG up vs IM
mmu-miR-344c-5p	0.537321	1.09111	IM+PG up vs IM
mmu-miR-873a-3p	0.537321	1.09111	IM+PG up vs IM
mmu-mir-8090	0.537321	1.09111	IM+PG up vs IM
mmu-miR-5127	0.666667	1.0912	IM+PG up vs IM
mmu-miR-3473c	0.666667	1.09139	IM+PG up vs IM
mmu-miR-1962	0.666667	1.09286	IM+PG up vs IM
mmu-mir-761	0.666667	1.09286	IM+PG up vs IM
mmu-miR-3086-3p	0.666667	1.09286	IM+PG up vs IM
mmu-miR-125b-5p	0.666667	1.09286	IM+PG up vs IM
mmu-miR-7a-5p	0.666667	1.09286	IM+PG up vs IM
mmu-miR-511-3p	0.666667	1.09286	IM+PG up vs IM
mmu-miR-467e-5p	0.666667	1.09286	IM+PG up vs IM
mmu-mir-7668	0.666667	1.09286	IM+PG up vs IM
mmu-miR-222-5p	0.666667	1.09286	IM+PG up vs IM
mmu-miR-5620-3p	0.666667	1.09286	IM+PG up vs IM
mmu-mir-324	0.666667	1.09286	IM+PG up vs IM
mmu-miR-188-5p	0.666667	1.09286	IM+PG up vs IM
mmu-miR-148b-5p	0.666667	1.09286	IM+PG up vs IM
mmu-mir-680-2	0.666667	1.09286	IM+PG up vs IM
mmu-miR-3060-5p	0.666667	1.09286	IM+PG up vs IM
mmu-miR-3103-5p	0.666667	1.09286	IM+PG up vs IM
mmu-mir-6991	0.666667	1.09286	IM+PG up vs IM
mmu-mir-6358	0.666667	1.09286	IM+PG up vs IM
mmu-mir-6899	0.666667	1.09286	IM+PG up vs IM
mmu-mir-7002	0.666667	1.09286	IM+PG up vs IM
mmu-miR-376c-3p	0.666667	1.09286	IM+PG up vs IM
mmu-mir-6335	0.600053	1.09298	IM+PG up vs IM
mmu-miR-30c-2-3p	0.617666	1.09317	IM+PG up vs IM
mmu-mir-3079	0	1.09334	IM+PG up vs IM

mmu-mir-7027	0	1.09334	IM+PG up vs IM
mmu-mir-7079	0	1.09334	IM+PG up vs IM
mmu-mir-195b	0.664936	1.09347	IM+PG up vs IM
mmu-mir-1898	0.198733	1.09406	IM+PG up vs IM
mmu-mir-20a	0.526878	1.09431	IM+PG up vs IM
mmu-miR-6390	0.840166	1.09531	IM+PG up vs IM
mmu-miR-293-3p	0.761365	1.09536	IM+PG up vs IM
mmu-miR-148a-3p	0.761365	1.09536	IM+PG up vs IM
mmu-mir-1970	0.545016	1.09568	IM+PG up vs IM
mmu-miR-1896	0.69958	1.09568	IM+PG up vs IM
mmu-mir-200b	0.545015	1.09568	IM+PG up vs IM
mmu-mir-7083	0.545015	1.09568	IM+PG up vs IM
mmu-miR-465b-5p	0.706308	1.09576	IM+PG up vs IM
mmu-miR-3473b	0.569312	1.09591	IM+PG up vs IM
mmu-mir-149	0.605537	1.09721	IM+PG up vs IM
mmu-miR-6952-5p	0.517243	1.09735	IM+PG up vs IM
mmu-mir-6917	0.837973	1.09739	IM+PG up vs IM
mmu-miR-7050-5p	0.396862	1.09787	IM+PG up vs IM
mmu-mir-6384	0.849503	1.09808	IM+PG up vs IM
mmu-miR-3470a	0	1.09841	IM+PG up vs IM
mmu-miR-7225-5p	0.684349	1.09878	IM+PG up vs IM
mmu-mir-1843a	0.666667	1.09881	IM+PG up vs IM
mmu-miR-3064-3p	0.666667	1.09881	IM+PG up vs IM
mmu-miR-5046	0.666667	1.09881	IM+PG up vs IM
mmu-mir-7090	0.666667	1.09881	IM+PG up vs IM
mmu-miR-7071-5p	0.666667	1.09881	IM+PG up vs IM
mmu-miR-296-5p	0.666667	1.09881	IM+PG up vs IM
mmu-miR-7036b-5p	0.666667	1.09881	IM+PG up vs IM
mmu-miR-6935-3p	0.666667	1.09881	IM+PG up vs IM
mmu-miR-1193-3p	0.666667	1.09881	IM+PG up vs IM
mmu-miR-7019-3p	0.666667	1.09881	IM+PG up vs IM
mmu-miR-6925-5p	0.666667	1.09881	IM+PG up vs IM
mmu-mir-7023	0.666667	1.09881	IM+PG up vs IM
mmu-miR-6934-3p	0.666667	1.09881	IM+PG up vs IM
mmu-miR-1905	0.666667	1.09881	IM+PG up vs IM
mmu-mir-466b-2	0.53507	1.09889	IM+PG up vs IM
mmu-miR-7226-3p	0.53507	1.09889	IM+PG up vs IM
mmu-mir-3473b	0.597326	1.10002	IM+PG up vs IM
mmu-miR-6908-5p	0.923662	1.10035	IM+PG up vs IM
mmu-miR-7679-5p	0.276087	1.1007	IM+PG up vs IM
mmu-mir-883a	0.666667	1.10124	IM+PG up vs IM
mmu-miR-6943-5p	0.666667	1.10124	IM+PG up vs IM
mmu-mir-6769b	0.666667	1.10124	IM+PG up vs IM

mmu-miR-669a-3p	0.547396	1.10126	IM+PG up vs IM
mmu-miR-669o-3p	0.547396	1.10126	IM+PG up vs IM
mmu-miR-7241-3p	0.666667	1.10129	IM+PG up vs IM
mmu-miR-6966-5p	0.666667	1.10129	IM+PG up vs IM
mmu-miR-143-3p	0.666667	1.10129	IM+PG up vs IM
mmu-miR-3087-5p	0.666667	1.10129	IM+PG up vs IM
mmu-miR-6413	0.666667	1.10129	IM+PG up vs IM
mmu-miR-200a-5p	0.666306	1.10131	IM+PG up vs IM
mmu-miR-7010-3p	0.666667	1.10144	IM+PG up vs IM
mmu-miR-7077-3p	0	1.1015	IM+PG up vs IM
mmu-miR-6398	0	1.1015	IM+PG up vs IM
mmu-miR-7060-3p	0.540006	1.10194	IM+PG up vs IM
mmu-miR-1946a	0.554607	1.10227	IM+PG up vs IM
mmu-mir-20b	0.666667	1.10227	IM+PG up vs IM
mmu-miR-698-5p	0.666667	1.10227	IM+PG up vs IM
mmu-mir-210	0.666667	1.10227	IM+PG up vs IM
mmu-mir-3970	0.666667	1.10227	IM+PG up vs IM
mmu-miR-3059-3p	0.666667	1.10227	IM+PG up vs IM
mmu-miR-3076-3p	0.833518	1.10288	IM+PG up vs IM
mmu-miR-7054-5p	0.54186	1.10315	IM+PG up vs IM
mmu-mir-7236	0.54186	1.10315	IM+PG up vs IM
mmu-mir-709	0.587069	1.10361	IM+PG up vs IM
mmu-mir-9-3	0.587069	1.10361	IM+PG up vs IM
mmu-miR-1950	0.587069	1.10361	IM+PG up vs IM
mmu-miR-503-3p	0.587069	1.10361	IM+PG up vs IM
mmu-mir-142b	0.542959	1.10388	IM+PG up vs IM
mmu-mir-291b	0.542959	1.10388	IM+PG up vs IM
mmu-mir-680-2	0.633836	1.10485	IM+PG up vs IM
mmu-mir-6964	0.845234	1.10491	IM+PG up vs IM
mmu-miR-500-5p	0.531761	1.10541	IM+PG up vs IM
mmu-miR-8112	0.203204	1.10632	IM+PG up vs IM
mmu-mir-7217	0.546787	1.10655	IM+PG up vs IM
mmu-miR-5132-5p	0.546787	1.10655	IM+PG up vs IM
mmu-miR-758-5p	0.560405	1.10685	IM+PG up vs IM
mmu-mir-463	0.717221	1.1077	IM+PG up vs IM
mmu-mir-684-1	0	1.1086	IM+PG up vs IM
mmu-mir-684-2	0	1.1086	IM+PG up vs IM
mmu-miR-3062-5p	0.8597	1.1097	IM+PG up vs IM
mmu-miR-6399	0.379816	1.11117	IM+PG up vs IM
mmu-miR-301a-3p	0.666667	1.11132	IM+PG up vs IM
mmu-miR-5103	0.666667	1.11132	IM+PG up vs IM
mmu-miR-7684-5p	0.666667	1.11132	IM+PG up vs IM
mmu-miR-669g	0.666667	1.11132	IM+PG up vs IM

mmu-miR-490-5p	0.666667	1.11132	IM+PG up vs IM
mmu-mir-1843a	0.666667	1.11132	IM+PG up vs IM
mmu-mir-1966	0.593343	1.11136	IM+PG up vs IM
mmu-mir-1963	0.593343	1.11136	IM+PG up vs IM
mmu-miR-450a-5p	0.565638	1.11144	IM+PG up vs IM
mmu-miR-135a-5p	0.565638	1.11144	IM+PG up vs IM
mmu-miR-1952	0.615998	1.11176	IM+PG up vs IM
mmu-miR-7076-3p	0.412956	1.11216	IM+PG up vs IM
mmu-miR-7024-5p	0.532105	1.11264	IM+PG up vs IM
mmu-let-7j	0.365226	1.11279	IM+PG up vs IM
mmu-miR-6973b-3p	0.568064	1.11375	IM+PG up vs IM
mmu-miR-503-5p	0.55604	1.11378	IM+PG up vs IM
mmu-miR-6980-3p	0.534325	1.11413	IM+PG up vs IM
mmu-mir-344-2	0.513862	1.11442	IM+PG up vs IM
mmu-mir-7048	0.682111	1.11466	IM+PG up vs IM
mmu-miR-7655-3p	0.557112	1.1147	IM+PG up vs IM
mmu-mir-6941	0.557112	1.1147	IM+PG up vs IM
mmu-miR-7010-5p	0.641146	1.11597	IM+PG up vs IM
mmu-miR-218-1-3p	0.0113842	1.11691	IM+PG up vs IM
mmu-miR-193b-3p	0.559712	1.11701	IM+PG up vs IM
mmu-miR-669b-3p	0.535075	1.11702	IM+PG up vs IM
mmu-miR-24-3p	0.213481	1.11755	IM+PG up vs IM
mmu-miR-24-1-5p	0.460023	1.11765	IM+PG up vs IM
mmu-mir-7044	0.460023	1.11765	IM+PG up vs IM
mmu-miR-694	0.826981	1.11805	IM+PG up vs IM
mmu-miR-3965	0.598503	1.11885	IM+PG up vs IM
mmu-miR-6939-5p	0.598503	1.11885	IM+PG up vs IM
mmu-mir-450a-2	0.176573	1.11888	IM+PG up vs IM
mmu-miR-7222-5p	0.666667	1.11892	IM+PG up vs IM
mmu-miR-7242-3p	0.666667	1.11892	IM+PG up vs IM
mmu-mir-3572	0.550541	1.11892	IM+PG up vs IM
mmu-mir-3963	0.666667	1.11892	IM+PG up vs IM
mmu-miR-181c-5p	0.666667	1.11892	IM+PG up vs IM
mmu-mir-297b	0.666667	1.11892	IM+PG up vs IM
mmu-miR-6996-5p	0.666667	1.11892	IM+PG up vs IM
mmu-miR-494-3p	0.666667	1.11892	IM+PG up vs IM
mmu-mir-5615-1	0.573401	1.11925	IM+PG up vs IM
mmu-mir-412	0.573401	1.11925	IM+PG up vs IM
mmu-miR-190a-5p	0.573401	1.11925	IM+PG up vs IM
mmu-miR-375-5p	0.573401	1.11925	IM+PG up vs IM
mmu-miR-5625-3p	0.573401	1.11925	IM+PG up vs IM
mmu-mir-8118	0.573401	1.11925	IM+PG up vs IM
mmu-miR-6350	0.573401	1.11925	IM+PG up vs IM

mmu-miR-1971	0.573401	1.11925	IM+PG up vs IM
mmu-mir-3082	0.573401	1.11925	IM+PG up vs IM
mmu-mir-7663	0.573401	1.11925	IM+PG up vs IM
mmu-miR-539-5p	0.573401	1.11925	IM+PG up vs IM
mmu-mir-669b	0.43151	1.11952	IM+PG up vs IM
mmu-mir-669b	0.43151	1.11952	IM+PG up vs IM
mmu-mir-6988	0.586028	1.1199	IM+PG up vs IM
mmu-mir-466h	0.542597	1.1202	IM+PG up vs IM
mmu-mir-186	0	1.12078	IM+PG up vs IM
mmu-mir-101c	0.563805	1.12089	IM+PG up vs IM
mmu-mir-467c	0.568624	1.12121	IM+PG up vs IM
mmu-mir-6996	0.66181	1.12201	IM+PG up vs IM
mmu-miR-344i	0.554772	1.12241	IM+PG up vs IM
mmu-miR-1964-3p	0.565439	1.12253	IM+PG up vs IM
mmu-miR-1191b-3p	0.565439	1.12253	IM+PG up vs IM
mmu-miR-6383	0.565439	1.12253	IM+PG up vs IM
mmu-miR-30c-5p	0.545676	1.12267	IM+PG up vs IM
mmu-mir-6375	0.545676	1.12267	IM+PG up vs IM
mmu-miR-6984-5p	0.828319	1.12338	IM+PG up vs IM
mmu-mir-7010	0.421586	1.1236	IM+PG up vs IM
mmu-mir-124-1	0.556114	1.12384	IM+PG up vs IM
mmu-miR-3082-5p	0.604106	1.12409	IM+PG up vs IM
mmu-miR-6951-5p	0.44399	1.12417	IM+PG up vs IM
mmu-miR-1964-5p	0.44399	1.12417	IM+PG up vs IM
mmu-miR-7037-5p	0.44399	1.12417	IM+PG up vs IM
mmu-miR-598-3p	0.113531	1.12428	IM+PG up vs IM
mmu-miR-6371	0.656033	1.12492	IM+PG up vs IM
mmu-miR-7238-3p	0.531489	1.125	IM+PG up vs IM
mmu-miR-5709-5p	0.841485	1.12521	IM+PG up vs IM
mmu-mir-7239	0.558026	1.12565	IM+PG up vs IM
mmu-miR-362-5p	0.835828	1.12621	IM+PG up vs IM
mmu-mir-487b	0.479992	1.12668	IM+PG up vs IM
mmu-mir-3057	0.559379	1.12677	IM+PG up vs IM
mmu-miR-8103	0.579812	1.12679	IM+PG up vs IM
mmu-miR-96-5p	0.579812	1.12679	IM+PG up vs IM
mmu-miR-344f-3p	0.579812	1.12679	IM+PG up vs IM
mmu-mir-365-1	0.735208	1.12777	IM+PG up vs IM
mmu-mir-2137	0.513311	1.12801	IM+PG up vs IM
mmu-miR-6973b-5p	0.552034	1.12822	IM+PG up vs IM
mmu-mir-1912	0.581093	1.12844	IM+PG up vs IM
mmu-miR-702-5p	0.581093	1.12844	IM+PG up vs IM
mmu-miR-15a-5p	0.57091	1.12844	IM+PG up vs IM
mmu-miR-448-3p	0.604125	1.12848	IM+PG up vs IM

mmu-miR-1264-5p	0.0728212	1.12895	IM+PG up vs IM
mmu-mir-342	0	1.12925	IM+PG up vs IM
mmu-miR-326-5p	0.56237	1.13001	IM+PG up vs IM
mmu-mir-3068	0.572329	1.13009	IM+PG up vs IM
mmu-miR-484	0.572329	1.13009	IM+PG up vs IM
mmu-mir-466b-3	0.572329	1.13009	IM+PG up vs IM
mmu-miR-6365	0.572329	1.13009	IM+PG up vs IM
mmu-miR-208a-3p	0.572329	1.13009	IM+PG up vs IM
mmu-mir-7014	0.572329	1.13009	IM+PG up vs IM
mmu-miR-6977-5p	0.487552	1.13063	IM+PG up vs IM
mmu-miR-5616-3p	0.818551	1.13114	IM+PG up vs IM
mmu-mir-138-2	0.820583	1.13288	IM+PG up vs IM
mmu-mir-138-2	0.820583	1.13288	IM+PG up vs IM
mmu-mir-7030	0.584387	1.13292	IM+PG up vs IM
mmu-miR-431-5p	0.584387	1.13292	IM+PG up vs IM
mmu-miR-883a-3p	0.584387	1.13292	IM+PG up vs IM
mmu-miR-451b	0.584387	1.13292	IM+PG up vs IM
mmu-mir-7665	0.584387	1.13292	IM+PG up vs IM
mmu-miR-5133	0.584387	1.13292	IM+PG up vs IM
mmu-mir-6972	0.556936	1.13295	IM+PG up vs IM
mmu-miR-681	0.623083	1.13309	IM+PG up vs IM
mmu-miR-6999-5p	0.565351	1.13323	IM+PG up vs IM
mmu-miR-7056-5p	0.637774	1.13325	IM+PG up vs IM
mmu-miR-1953	0.479597	1.13466	IM+PG up vs IM
mmu-miR-7075-5p	0.816422	1.1347	IM+PG up vs IM
mmu-miR-467a-3p	0.586065	1.13536	IM+PG up vs IM
mmu-mir-6357	0.586113	1.13543	IM+PG up vs IM
mmu-mir-6379	0.586113	1.13543	IM+PG up vs IM
mmu-miR-874-3p	0.506561	1.13587	IM+PG up vs IM
mmu-mir-330	0.666667	1.13605	IM+PG up vs IM
mmu-mir-6344	0.577253	1.13624	IM+PG up vs IM
mmu-miR-1948-3p	0.586825	1.13649	IM+PG up vs IM
mmu-miR-3099-3p	0.586825	1.13649	IM+PG up vs IM
mmu-mir-181b-1	0.586825	1.13649	IM+PG up vs IM
mmu-miR-6941-3p	0.530865	1.13771	IM+PG up vs IM
mmu-miR-3090-5p	0.578432	1.13782	IM+PG up vs IM
mmu-miR-6979-5p	0.457417	1.13816	IM+PG up vs IM
mmu-miR-33-3p	0.457417	1.13816	IM+PG up vs IM
mmu-miR-1983	0.411998	1.13857	IM+PG up vs IM
mmu-let-7a-2	0.579112	1.13875	IM+PG up vs IM
mmu-mir-7045	0.37828	1.13911	IM+PG up vs IM
mmu-mir-1950	0.570592	1.1394	IM+PG up vs IM
mmu-miR-7028-3p	0.570592	1.1394	IM+PG up vs IM

mmu-miR-8120	0.570592	1.1394	IM+PG up vs IM
mmu-miR-7033-3p	0.579879	1.13982	IM+PG up vs IM
mmu-mir-669c	0.572572	1.14192	IM+PG up vs IM
mmu-miR-6983-5p	0.572616	1.14197	IM+PG up vs IM
mmu-miR-409-5p	0.310564	1.14218	IM+PG up vs IM
mmu-miR-767	0.310564	1.14218	IM+PG up vs IM
mmu-mir-5098	0.556389	1.14223	IM+PG up vs IM
mmu-miR-194-2-3p	0.424151	1.14333	IM+PG up vs IM
mmu-mir-1968	0.646272	1.14337	IM+PG up vs IM
mmu-miR-3083-5p	0.591335	1.14373	IM+PG up vs IM
mmu-mir-6363	0.794071	1.14393	IM+PG up vs IM
mmu-miR-669h-5p	0.291628	1.14475	IM+PG up vs IM
mmu-miR-668-3p	0.749432	1.14503	IM+PG up vs IM
mmu-miR-7231-3p	0	1.1451	IM+PG up vs IM
mmu-miR-3073a-3p	0.486914	1.14512	IM+PG up vs IM
mmu-miR-32-5p	0.485739	1.14569	IM+PG up vs IM
mmu-mir-6337	0.485739	1.14569	IM+PG up vs IM
mmu-miR-222-3p	0.666667	1.14747	IM+PG up vs IM
mmu-miR-466b-5p	0.3925	1.14842	IM+PG up vs IM
mmu-miR-466o-5p	0.3925	1.14842	IM+PG up vs IM
mmu-miR-615-5p	0.779153	1.14852	IM+PG up vs IM
mmu-miR-6408	0.666667	1.14878	IM+PG up vs IM
mmu-miR-717	0.666667	1.14878	IM+PG up vs IM
mmu-mir-6405	0.666667	1.14878	IM+PG up vs IM
mmu-miR-6366	0.144191	1.15018	IM+PG up vs IM
mmu-mir-6977	0.72703	1.15077	IM+PG up vs IM
mmu-miR-7083-3p	0.442092	1.1513	IM+PG up vs IM
mmu-mir-679	0.141884	1.15154	IM+PG up vs IM
mmu-miR-191-3p	0.141884	1.15154	IM+PG up vs IM
mmu-miR-6919-3p	0.141884	1.15154	IM+PG up vs IM
mmu-mir-5098	0.564653	1.1519	IM+PG up vs IM
mmu-mir-7225	0.499247	1.15316	IM+PG up vs IM
mmu-miR-6896-3p	0.499247	1.15316	IM+PG up vs IM
mmu-miR-6940-5p	0.405023	1.15322	IM+PG up vs IM
mmu-mir-466n	0.596714	1.15366	IM+PG up vs IM
mmu-miR-6947-5p	0	1.15468	IM+PG up vs IM
mmu-miR-883a-5p	0.629224	1.15568	IM+PG up vs IM
mmu-miR-7021-3p	0.13836	1.15583	IM+PG up vs IM
mmu-mir-764	0.675199	1.1563	IM+PG up vs IM
mmu-mir-451a	0.415701	1.15769	IM+PG up vs IM
mmu-mir-6939	0.415701	1.15769	IM+PG up vs IM
mmu-mir-1969	0.630659	1.16011	IM+PG up vs IM
mmu-miR-7217-5p	0.173509	1.1613	IM+PG up vs IM

mmu-miR-150-5p	0.666667	1.16136	IM+PG up vs IM
mmu-miR-204-5p	0.666667	1.16136	IM+PG up vs IM
mmu-miR-7028-5p	0.666667	1.16136	IM+PG up vs IM
mmu-mir-8113	0.666667	1.16136	IM+PG up vs IM
mmu-miR-7241-5p	0.666667	1.16136	IM+PG up vs IM
mmu-miR-7002-5p	0.730919	1.16194	IM+PG up vs IM
mmu-miR-470-3p	0.619947	1.16305	IM+PG up vs IM
mmu-miR-193a-3p	0.450597	1.16414	IM+PG up vs IM
mmu-mir-1896	0.493242	1.16424	IM+PG up vs IM
mmu-mir-7072	0.269696	1.16545	IM+PG up vs IM
mmu-mir-216b	0.474616	1.16647	IM+PG up vs IM
mmu-mir-8095	0	1.16777	IM+PG up vs IM
mmu-miR-7212-5p	0.129358	1.1679	IM+PG up vs IM
mmu-miR-7678-5p	0.472065	1.16793	IM+PG up vs IM
mmu-mir-6244	0.472065	1.16793	IM+PG up vs IM
mmu-mir-5100	0.206178	1.16859	IM+PG up vs IM
mmu-miR-293-5p	0.145692	1.16946	IM+PG up vs IM
mmu-miR-3113-5p	0.677485	1.17001	IM+PG up vs IM
mmu-let-7d-3p	0.666667	1.17019	IM+PG up vs IM
mmu-mir-6932	0.110251	1.17086	IM+PG up vs IM
mmu-miR-135a-1-3p	0.242516	1.17136	IM+PG up vs IM
mmu-mir-7004	0.769149	1.17168	IM+PG up vs IM
mmu-miR-7038-3p	0.666667	1.17221	IM+PG up vs IM
mmu-mir-7672	0.666667	1.17221	IM+PG up vs IM
mmu-mir-466k	0.767805	1.17239	IM+PG up vs IM
mmu-mir-6400	0	1.173	IM+PG up vs IM
mmu-mir-7076	0	1.173	IM+PG up vs IM
mmu-miR-5131	0	1.173	IM+PG up vs IM
mmu-miR-693-3p	0	1.173	IM+PG up vs IM
mmu-miR-883b-3p	0	1.173	IM+PG up vs IM
mmu-miR-217-3p	0	1.173	IM+PG up vs IM
mmu-mir-7049	0.714076	1.17472	IM+PG up vs IM
mmu-miR-1932	0.621935	1.1761	IM+PG up vs IM
mmu-miR-6985-3p	0.667763	1.1767	IM+PG up vs IM
mmu-miR-423-5p	0.86973	1.1768	IM+PG up vs IM
mmu-miR-599	0.027904	1.17787	IM+PG up vs IM
mmu-miR-7218-5p	0.454987	1.17803	IM+PG up vs IM
mmu-miR-6382	0.693947	1.17979	IM+PG up vs IM
mmu-mir-222	0.607968	1.18072	IM+PG up vs IM
mmu-miR-5108	0.725131	1.18321	IM+PG up vs IM
mmu-mir-5624	0.609229	1.18445	IM+PG up vs IM
mmu-miR-6372	0.603457	1.18619	IM+PG up vs IM
mmu-mir-6339	0.666667	1.18635	IM+PG up vs IM

mmu-miR-344f-5p	0.666667	1.18635	IM+PG up vs IM
mmu-miR-140-5p	0.666667	1.18635	IM+PG up vs IM
mmu-mir-293	0.666667	1.18635	IM+PG up vs IM
mmu-miR-6388	0.776704	1.18684	IM+PG up vs IM
mmu-miR-7088-5p	0.666667	1.18694	IM+PG up vs IM
mmu-miR-7667-5p	0.666667	1.18694	IM+PG up vs IM
mmu-miR-6420	0.604082	1.18792	IM+PG up vs IM
mmu-miR-6926-3p	0.537783	1.18862	IM+PG up vs IM
mmu-mir-6369	0.303164	1.18906	IM+PG up vs IM
mmu-mir-6383	0.734942	1.18921	IM+PG up vs IM
mmu-miR-483-3p	0.666667	1.18962	IM+PG up vs IM
mmu-mir-1a-2	0	1.18966	IM+PG up vs IM
mmu-mir-450a-2	0	1.18966	IM+PG up vs IM
mmu-mir-680-3	0	1.18966	IM+PG up vs IM
mmu-mir-590	0	1.18966	IM+PG up vs IM
mmu-mir-449b	0	1.18966	IM+PG up vs IM
mmu-mir-544	0	1.18966	IM+PG up vs IM
mmu-mir-1953	0	1.18966	IM+PG up vs IM
mmu-mir-6931	0	1.18966	IM+PG up vs IM
mmu-mir-7015	0	1.18966	IM+PG up vs IM
mmu-mir-7086	0	1.18966	IM+PG up vs IM
mmu-mir-7671	0	1.18966	IM+PG up vs IM
mmu-miR-145a-5p	0	1.18966	IM+PG up vs IM
mmu-miR-201-5p	0	1.18966	IM+PG up vs IM
mmu-miR-542-3p	0	1.18966	IM+PG up vs IM
mmu-miR-27a-5p	0	1.18966	IM+PG up vs IM
mmu-miR-582-5p	0	1.18966	IM+PG up vs IM
mmu-miR-1902	0	1.18966	IM+PG up vs IM
mmu-miR-3108-3p	0	1.18966	IM+PG up vs IM
mmu-miR-1198-3p	0	1.18966	IM+PG up vs IM
mmu-miR-30f	0	1.18966	IM+PG up vs IM
mmu-miR-6960-5p	0	1.18966	IM+PG up vs IM
mmu-miR-6978-5p	0	1.18966	IM+PG up vs IM
mmu-miR-6982-3p	0	1.18966	IM+PG up vs IM
mmu-miR-7683-3p	0	1.18966	IM+PG up vs IM
mmu-miR-1668	0	1.18966	IM+PG up vs IM
mmu-let-7g-3p	0.769297	1.19016	IM+PG up vs IM
mmu-miR-7029-3p	0.0956839	1.19107	IM+PG up vs IM
mmu-miR-7227-5p	0.095684	1.19107	IM+PG up vs IM
mmu-mir-880	0.114246	1.19277	IM+PG up vs IM
mmu-miR-1197-5p	0.114246	1.19277	IM+PG up vs IM
mmu-mir-669k	0.114246	1.19277	IM+PG up vs IM
mmu-miR-7053-5p	0.666667	1.19358	IM+PG up vs IM

mmu-miR-412-5p	0.111398	1.19447	IM+PG up vs IM
mmu-mir-6356	0.113148	1.19486	IM+PG up vs IM
mmu-miR-6994-5p	0.722296	1.19541	IM+PG up vs IM
mmu-miR-6963-3p	0.0974734	1.19579	IM+PG up vs IM
mmu-miR-6368	0.123684	1.19723	IM+PG up vs IM
mmu-miR-5623-3p	0.613188	1.19742	IM+PG up vs IM
mmu-miR-7060-5p	0.613188	1.19742	IM+PG up vs IM
mmu-mir-1947	0.121362	1.19897	IM+PG up vs IM
mmu-miR-145b	0.56104	1.19982	IM+PG up vs IM
mmu-miR-6236	0.56104	1.19982	IM+PG up vs IM
mmu-miR-7029-5p	0.0953289	1.20069	IM+PG up vs IM
mmu-mir-344e	0.666667	1.20084	IM+PG up vs IM
mmu-mir-18a	0.666667	1.20084	IM+PG up vs IM
mmu-miR-468-3p	0.180548	1.20385	IM+PG up vs IM
mmu-mir-669l	0.404506	1.20434	IM+PG up vs IM
mmu-miR-7020-3p	0.675747	1.20487	IM+PG up vs IM
mmu-miR-216a-3p	0.0802693	1.20609	IM+PG up vs IM
mmu-miR-338-3p	0.600174	1.20707	IM+PG up vs IM
mmu-mir-669i	0.616161	1.20861	IM+PG up vs IM
mmu-mir-7682	0	1.20896	IM+PG up vs IM
mmu-miR-28a-3p	0.122132	1.20902	IM+PG up vs IM
mmu-miR-511-5p	0.283373	1.20989	IM+PG up vs IM
mmu-miR-296-3p	0.629437	1.21098	IM+PG up vs IM
mmu-miR-7048-5p	0.103355	1.21144	IM+PG up vs IM
mmu-miR-3102-5p.2-5p	0.666667	1.21278	IM+PG up vs IM
mmu-miR-8104	0.666667	1.21278	IM+PG up vs IM
mmu-mir-320	0.176842	1.21284	IM+PG up vs IM
mmu-mir-1188	0.630058	1.21456	IM+PG up vs IM
mmu-mir-1191b	0	1.215	IM+PG up vs IM
mmu-miR-129-2-3p	0	1.215	IM+PG up vs IM
mmu-mir-30b	0	1.215	IM+PG up vs IM
mmu-mir-1199	0	1.215	IM+PG up vs IM
mmu-mir-6340	0	1.215	IM+PG up vs IM
mmu-mir-6341	0	1.215	IM+PG up vs IM
mmu-mir-6343	0	1.215	IM+PG up vs IM
mmu-mir-6906	0	1.215	IM+PG up vs IM
mmu-mir-6981	0	1.215	IM+PG up vs IM
mmu-miR-217-5p	0	1.215	IM+PG up vs IM
mmu-miR-678	0	1.215	IM+PG up vs IM
mmu-miR-3084-5p	0	1.215	IM+PG up vs IM
mmu-miR-6377	0	1.215	IM+PG up vs IM
mmu-miR-7023-3p	0	1.215	IM+PG up vs IM
mmu-miR-7027-5p	0	1.215	IM+PG up vs IM

mmu-miR-7689-5p	0	1.215	IM+PG up vs IM
mmu-miR-8118	0	1.215	IM+PG up vs IM
mmu-mir-93	0	1.215	IM+PG up vs IM
mmu-mir-8103	0.118348	1.21538	IM+PG up vs IM
mmu-mir-3077	0.00821471	1.21677	IM+PG up vs IM
mmu-mir-6952	0.603348	1.21682	IM+PG up vs IM
mmu-mir-31	0	1.21712	IM+PG up vs IM
mmu-miR-7686-3p	0	1.21712	IM+PG up vs IM
mmu-mir-1940	0.132622	1.21873	IM+PG up vs IM
mmu-miR-7215-5p	0.42026	1.2191	IM+PG up vs IM
mmu-miR-125a-5p	0.805225	1.21934	IM+PG up vs IM
mmu-miR-369-5p	0	1.21997	IM+PG up vs IM
mmu-mir-340	0.172983	1.22144	IM+PG up vs IM
mmu-mir-1949	0.619539	1.22318	IM+PG up vs IM
mmu-mir-7b	0.619539	1.22318	IM+PG up vs IM
mmu-miR-3092-5p	0.619539	1.22318	IM+PG up vs IM
mmu-miR-7683-5p	0.619539	1.22318	IM+PG up vs IM
mmu-miR-574-3p	0.619539	1.22318	IM+PG up vs IM
mmu-miR-434-5p	0.61967	1.22379	IM+PG up vs IM
mmu-miR-3471	0.617494	1.22489	IM+PG up vs IM
mmu-mir-6345	0.575087	1.22655	IM+PG up vs IM
mmu-mir-6538	0.615262	1.22676	IM+PG up vs IM
mmu-miR-6405	0.616037	1.23015	IM+PG up vs IM
mmu-miR-8108	0.822998	1.23084	IM+PG up vs IM
mmu-mir-6953	0.517648	1.23343	IM+PG up vs IM
mmu-miR-878-5p	0.633035	1.23375	IM+PG up vs IM
mmu-mir-28a	0	1.23503	IM+PG up vs IM
mmu-mir-2183	0	1.23503	IM+PG up vs IM
mmu-mir-6413	0	1.23503	IM+PG up vs IM
mmu-mir-8119	0	1.23503	IM+PG up vs IM
mmu-miR-495-3p	0	1.23503	IM+PG up vs IM
mmu-miR-759	0	1.23503	IM+PG up vs IM
mmu-miR-21a-3p	0	1.23503	IM+PG up vs IM
mmu-miR-3105-5p	0	1.23503	IM+PG up vs IM
mmu-miR-378a-5p	0.781742	1.23565	IM+PG up vs IM
mmu-mir-1945	0.0791898	1.23587	IM+PG up vs IM
mmu-miR-1981-5p	0.666667	1.23731	IM+PG up vs IM
mmu-miR-719	0.422631	1.23783	IM+PG up vs IM
mmu-let-7k	0.622531	1.23813	IM+PG up vs IM
mmu-miR-1249-3p	0	1.2414	IM+PG up vs IM
mmu-miR-3080-5p	0	1.2414	IM+PG up vs IM
mmu-miR-3473d	0	1.2414	IM+PG up vs IM
mmu-miR-8099	0	1.2414	IM+PG up vs IM

mmu-miR-135b-5p	0.61851	1.24175	IM+PG up vs IM
mmu-miR-7684-3p	0.0314084	1.2427	IM+PG up vs IM
mmu-miR-7003-5p	0.0344584	1.24348	IM+PG up vs IM
mmu-miR-6904-5p	0.544619	1.24364	IM+PG up vs IM
mmu-miR-376b-3p	0.569995	1.2437	IM+PG up vs IM
mmu-miR-290a-5p	0.514577	1.24573	IM+PG up vs IM
mmu-miR-7034-3p	0.0504415	1.24762	IM+PG up vs IM
mmu-miR-221-5p	0.547953	1.2495	IM+PG up vs IM
mmu-miR-207	0.472862	1.25027	IM+PG up vs IM
mmu-miR-130a-3p	0.262316	1.2517	IM+PG up vs IM
mmu-miR-3109-5p	0.532682	1.25489	IM+PG up vs IM
mmu-miR-8115	0.0870897	1.25775	IM+PG up vs IM
mmu-mir-7220	0.36628	1.25803	IM+PG up vs IM
mmu-miR-592-3p	0.337682	1.2587	IM+PG up vs IM
mmu-mir-7218	0.418944	1.26312	IM+PG up vs IM
mmu-miR-6971-3p	0.268732	1.26423	IM+PG up vs IM
mmu-miR-670-5p	0.322187	1.26446	IM+PG up vs IM
mmu-miR-34b-5p	0.666667	1.26623	IM+PG up vs IM
mmu-miR-7243-3p	0.327178	1.26708	IM+PG up vs IM
mmu-miR-6961-5p	0.666667	1.26738	IM+PG up vs IM
mmu-miR-6985-5p	0.637611	1.27179	IM+PG up vs IM
mmu-miR-7210-3p	0.33684	1.27237	IM+PG up vs IM
mmu-mir-7053	0.628206	1.27356	IM+PG up vs IM
mmu-miR-6979-3p	0.491323	1.27423	IM+PG up vs IM
mmu-miR-5626-5p	0.670519	1.27448	IM+PG up vs IM
mmu-mir-3090	0	1.27507	IM+PG up vs IM
mmu-mir-7670	0	1.27507	IM+PG up vs IM
mmu-miR-376a-5p	0	1.27507	IM+PG up vs IM
mmu-mir-6954	0	1.27507	IM+PG up vs IM
mmu-mir-465c-1	0.336968	1.27511	IM+PG up vs IM
mmu-mir-465c-2	0.336968	1.27511	IM+PG up vs IM
mmu-miR-322-5p	0.414279	1.27635	IM+PG up vs IM
mmu-mir-1193	0.34958	1.27638	IM+PG up vs IM
mmu-miR-100-3p	0.645708	1.27863	IM+PG up vs IM
mmu-miR-18a-5p	0.227989	1.27894	IM+PG up vs IM
mmu-mir-132	0.589943	1.27926	IM+PG up vs IM
mmu-miR-674-5p	0.432136	1.28085	IM+PG up vs IM
mmu-miR-3572-5p	0.425981	1.28613	IM+PG up vs IM
mmu-miR-666-5p	0.628357	1.28701	IM+PG up vs IM
mmu-miR-15b-5p	0.360171	1.28932	IM+PG up vs IM
mmu-miR-703	0.322995	1.29143	IM+PG up vs IM
mmu-miR-1306-5p	0.322995	1.29143	IM+PG up vs IM
mmu-mir-7029	0.322995	1.29143	IM+PG up vs IM

mmu-miR-7656-5p	0.666953	1.29313	IM+PG up vs IM
mmu-mir-146b	0.63086	1.29444	IM+PG up vs IM
mmu-mir-137	0.259484	1.29616	IM+PG up vs IM
mmu-miR-669f-3p	0.384338	1.29644	IM+PG up vs IM
mmu-miR-342-5p	0.666667	1.29716	IM+PG up vs IM
mmu-miR-7648-5p	0.666667	1.29716	IM+PG up vs IM
mmu-miR-6938-3p	0.589529	1.29719	IM+PG up vs IM
mmu-miR-184-3p	0.376905	1.29802	IM+PG up vs IM
mmu-miR-3110-3p	0.670765	1.29846	IM+PG up vs IM
mmu-mir-326	0.575384	1.2994	IM+PG up vs IM
mmu-miR-532-5p	0.288485	1.29973	IM+PG up vs IM
mmu-mir-8093	0.337443	1.30013	IM+PG up vs IM
mmu-miR-6375	0.291997	1.30068	IM+PG up vs IM
mmu-miR-106b-3p	0	1.3022	IM+PG up vs IM
mmu-miR-471-5p	0.666667	1.30357	IM+PG up vs IM
mmu-miR-126a-5p	0.666667	1.30357	IM+PG up vs IM
mmu-miR-1843a-5p	0.526584	1.30457	IM+PG up vs IM
mmu-miR-122-5p	0.666667	1.30717	IM+PG up vs IM
mmu-mir-6402	0.350654	1.30887	IM+PG up vs IM
mmu-mir-361	0.640958	1.30926	IM+PG up vs IM
mmu-mir-6915	0.235237	1.31245	IM+PG up vs IM
mmu-miR-432	0.290818	1.31245	IM+PG up vs IM
mmu-miR-7116-5p	0.632851	1.31253	IM+PG up vs IM
mmu-mir-451a	0.21966	1.31372	IM+PG up vs IM
mmu-mir-181a-2	0.645683	1.315	IM+PG up vs IM
mmu-miR-1298-5p	0.302046	1.31893	IM+PG up vs IM
mmu-mir-17	0.369049	1.32209	IM+PG up vs IM
mmu-miR-15a-3p	0.31528	1.3271	IM+PG up vs IM
mmu-mir-433	0.074686	1.32755	IM+PG up vs IM
mmu-mir-678	0.316412	1.32782	IM+PG up vs IM
mmu-mir-883b	0.316412	1.32782	IM+PG up vs IM
mmu-miR-6917-3p	0.316412	1.32782	IM+PG up vs IM
mmu-mir-128-1	0.314776	1.33014	IM+PG up vs IM
mmu-miR-362-3p	0.289959	1.33677	IM+PG up vs IM
mmu-miR-133b-5p	0.175444	1.33699	IM+PG up vs IM
mmu-miR-543-5p	0.32566	1.33738	IM+PG up vs IM
mmu-mir-1938	0.331555	1.338	IM+PG up vs IM
mmu-miR-466i-3p	0.331555	1.338	IM+PG up vs IM
mmu-miR-191-5p	0.0932833	1.34562	IM+PG up vs IM
mmu-miR-219c-3p	0.283123	1.3476	IM+PG up vs IM
mmu-mir-6963	0.636096	1.34775	IM+PG up vs IM
mmu-mir-7068	0.584735	1.34933	IM+PG up vs IM
mmu-miR-17-3p	0.524239	1.35046	IM+PG up vs IM

mmu-miR-34c-5p	0.025338	1.35285	IM+PG up vs IM
mmu-miR-7048-3p	0	1.36306	IM+PG up vs IM
mmu-miR-590-5p	0.417373	1.36666	IM+PG up vs IM
mmu-miR-711	0.666667	1.36793	IM+PG up vs IM
mmu-miR-93-5p	0.552191	1.36907	IM+PG up vs IM
mmu-miR-194-5p	0.334489	1.37343	IM+PG up vs IM
mmu-miR-5098	0	1.37408	IM+PG up vs IM
mmu-miR-6378	0.33709	1.37551	IM+PG up vs IM
mmu-miR-30d-5p	0.301091	1.37569	IM+PG up vs IM
mmu-miR-483-5p	0.013324	1.37741	IM+PG up vs IM
mmu-mir-883a	0.430148	1.38163	IM+PG up vs IM
mmu-miR-680	0.294714	1.38679	IM+PG up vs IM
mmu-miR-669c-3p	0.437437	1.39096	IM+PG up vs IM
mmu-miR-7230-3p	0.440105	1.39454	IM+PG up vs IM
mmu-miR-5129-3p	0.397469	1.39577	IM+PG up vs IM
mmu-mir-224	0.395892	1.39821	IM+PG up vs IM
mmu-miR-299a-3p	0	1.40191	IM+PG up vs IM
mmu-miR-7227-3p	0	1.40191	IM+PG up vs IM
mmu-miR-7040-5p	0.366858	1.41352	IM+PG up vs IM
mmu-miR-133c	0.412705	1.41355	IM+PG up vs IM
mmu-miR-3061-5p	0.248106	1.4136	IM+PG up vs IM
mmu-mir-106a	0.0488208	1.42835	IM+PG up vs IM
mmu-miR-7016-3p	0.314269	1.42869	IM+PG up vs IM
mmu-miR-465c-5p	0.196198	1.43802	IM+PG up vs IM
mmu-miR-361-5p	0.0525902	1.45118	IM+PG up vs IM
mmu-miR-320-3p	0.080062	1.45337	IM+PG up vs IM
mmu-miR-7685-3p	0.184508	1.45584	IM+PG up vs IM
mmu-mir-383	0.444468	1.45902	IM+PG up vs IM
mmu-miR-27a-3p	0.469977	1.46457	IM+PG up vs IM
mmu-miR-6969-3p	0.155134	1.48358	IM+PG up vs IM
mmu-miR-7666-5p	0	1.49115	IM+PG up vs IM
mmu-miR-7012-3p	0.515688	1.50009	IM+PG up vs IM
mmu-mir-543	0.248377	1.50105	IM+PG up vs IM
mmu-miR-7016-5p	0.155048	1.56951	IM+PG up vs IM
mmu-miR-7036-5p	0.565311	1.57326	IM+PG up vs IM
mmu-miR-24-2-5p	0.198141	1.57975	IM+PG up vs IM
mmu-miR-146a-5p	0.382172	1.58892	IM+PG up vs IM
mmu-miR-151-3p	0.484104	1.59725	IM+PG up vs IM
mmu-miR-34b-3p	0.298947	1.6459	IM+PG up vs IM
mmu-miR-181a-1-3p	0.170027	1.68136	IM+PG up vs IM
mmu-miR-181a-5p	0.292594	1.69559	IM+PG up vs IM
mmu-miR-17-5p	0.00672965	1.71043	IM+PG up vs IM
mmu-miR-185-5p	0.110208	1.73014	IM+PG up vs IM

mmu-mir-212	0.283715	1.73623	IM+PG up vs IM
mmu-miR-324-5p	0.022696	1.76033	IM+PG up vs IM
mmu-miR-23a-3p	0.117752	1.76243	IM+PG up vs IM
mmu-miR-5128	0.388242	1.78399	IM+PG up vs IM
mmu-miR-652-3p	0.302535	1.7935	IM+PG up vs IM
mmu-let-7g-5p	0.43071	1.85936	IM+PG up vs IM
mmu-miR-16-5p	0.0720105	1.86115	IM+PG up vs IM
mmu-miR-7221-5p	0.426227	1.86285	IM+PG up vs IM
mmu-miR-140-3p	0.0981016	1.87762	IM+PG up vs IM
mmu-miR-486-5p	0.416388	1.90332	IM+PG up vs IM
mmu-miR-3107-5p	0.416388	1.90332	IM+PG up vs IM
mmu-miR-425-5p	0.40879	1.91123	IM+PG up vs IM
mmu-miR-2137	0.114334	2.21331	IM+PG up vs IM
mmu-miR-1940	0.484688	2.28689	IM+PG up vs IM
mmu-miR-20a-5p	0.139558	2.30894	IM+PG up vs IM
mmu-miR-106b-5p	0.0223135	2.3942	IM+PG up vs IM
mmu-miR-146b-5p	0.175044	2.57713	IM+PG up vs IM
mmu-miR-378d	0.334126	2.79839	IM+PG up vs IM
mmu-miR-378b	0.0459755	2.81608	IM+PG up vs IM
mmu-miR-342-3p	0.0824228	2.84725	IM+PG up vs IM
mmu-miR-181b-5p	0.397956	2.88516	IM+PG up vs IM
mmu-miR-212-3p	0.136304	2.91851	IM+PG up vs IM
mmu-miR-8101	0.0685086	3.10551	IM+PG up vs IM
mmu-miR-7052-5p	0.0237201	3.30719	IM+PG up vs IM
mmu-miR-378a-3p	0.0499991	3.82516	IM+PG up vs IM
mmu-miR-132-3p	0	4.14981	IM+PG up vs IM
mmu-miR-378c	0.170716	6.24246	IM+PG up vs IM
mmu-miR-1224-5p	0.0577113	6.572	IM+PG up vs IM
mmu-miR-221-3p	0.0509443	6.88652	IM+PG up vs IM
