# Supplementary material for: Porphyromonas gingivalis Provokes Exosome Secretion and Paracrine Immune Senescence in Bystander Dendritic Cells
Source: Front Cell Infect Microbiol. 2021 Jun 1;11:669989. doi: 10.3389/fcimb.2021.669989 (PMC8204290; doi:10.3389/fcimb.2021.669989)
Supplement: Supplementary file 3 [file DataSheet_2.pdf]

Transcript ID(Array Design)	p-value(StimDCs EXO vs. IDCs EXO)	Fold-Change(StimDCs EXO vs. IDCs EXO)	Fold-Change(StimDCs EXO vs. IDCs EXO) (Description)
mmu-miR-7686-5p	0.226265	-11.7925	M down vs IM
mmu-miR-6937-5p	0.00608417	-3.44304	M down vs IM
mmu-miR-7047-5p	0.0747058	-4.98528	M down vs IM
mmu-miR-1934-3p	0.062668	-5.27142	M down vs IM
mmu-miR-7648-3p	0.0604193	-3.43169	M down vs IM
mmu-miR-6970-5p	0.0772582	-6.21128	M down vs IM
mmu-miR-7658-5p	0.0880033	-3.15822	M down vs IM
mmu-miR-3620-5p	0.323885	-3.44443	M down vs IM
mmu-miR-7221-3p	0.276679	-4.65042	M down vs IM
mmu-miR-346-3p	0.166671	-3.18846	M down vs IM
mmu-miR-3102-5p	0.541776	-1.13901	M down vs IM
mmu-miR-7235-5p	0.0212049	-3.49376	M down vs IM
mmu-miR-762	0.297906	-1.93934	M down vs IM
mmu-miR-690	0.274901	-2.64182	M down vs IM
mmu-miR-155-5p	0.0723588	2.12086	M up vs IM
mmu-miR-7238-5p	0.0320517	-2.60591	M down vs IM
mmu-miR-6909-5p	0.0810929	-2.26929	M down vs IM
mmu-miR-6944-5p	0.315974	-2.31194	M down vs IM
mmu-miR-328-5p	0.0313186	-2.07369	M down vs IM
mmu-miR-705	0.276373	-2.03588	M down vs IM
mmu-miR-501-3p	0.489121	-1.72843	M down vs IM
mmu-miR-7044-5p	0.136451	-2.23334	M down vs IM
mmu-miR-6931-5p	0.948048	1.02732	M up vs IM
mmu-miR-5126	0.0426035	-1.67351	M down vs IM
mmu-miR-3077-5p	0.0826013	-1.09669	M down vs IM
mmu-miR-7023-5p	0.145905	-1.90354	M down vs IM
mmu-miR-3547-5p	0.229849	1.06207	M up vs IM
mmu-miR-6991-5p	0	-2.59036	M down vs IM
mmu-miR-7687-5p	0.411524	-1.5729	M down vs IM
mmu-miR-6970-3p	0.386099	-1.60335	M down vs IM
mmu-miR-6987-3p	0.0318921	-1.5772	M down vs IM
mmu-miR-8109	0.193755	-1.58401	M down vs IM
mmu-miR-6240	0.543132	1.15488	M up vs IM
mmu-miR-675-5p	0.229692	-1.5451	M down vs IM
mmu-miR-6959-5p	0.151905	-1.63502	M down vs IM
mmu-miR-7085-5p	0.534667	-1.42464	M down vs IM
mmu-miR-6349	0	-1.65692	M down vs IM
mmu-miR-147-3p	0	-1.03355	M down vs IM

mmu-miR-7118-5p	0.567177	1.27264	M up vs IM
mmu-miR-1968-3p	0.298882	-1.52003	M down vs IM
mmu-mir-6946	0	-1.91495	M down vs IM
mmu-miR-6911-5p	0.194089	-1.56688	M down vs IM
mmu-miR-667-5p	0.0767952	-1.54792	M down vs IM
mmu-miR-7003-3p	0.389541	-1.62746	M down vs IM
mmu-miR-7115-3p	0.448707	-1.08698	M down vs IM
mmu-miR-3547-3p	0.361874	-1.61325	M down vs IM
mmu-miR-3968	0	-1.63502	M down vs IM
mmu-miR-7654-3p	0	-1.83106	M down vs IM
mmu-miR-291b-3p	0.228081	-1.67323	M down vs IM
mmu-miR-6906-5p	0	-1.61033	M down vs IM
mmu-miR-504-3p	0.469681	-1.38748	M down vs IM
mmu-miR-1943-3p	0.0498269	-1.63502	M down vs IM
mmu-miR-6910-5p	0.0378693	-1.38748	M down vs IM
mmu-mir-3070b	0.108958	-1.32211	M down vs IM
mmu-miR-5100	0	1.31837	M up vs IM
mmu-miR-344e-5p	0	-1.56688	M down vs IM
mmu-miR-344h-5p	0	-1.56688	M down vs IM
mmu-miR-7030-5p	0.178862	-1.68263	M down vs IM
mmu-miR-3544-5p	0.273707	-1.38748	M down vs IM
mmu-mir-219a-1	0.0477741	-1.52774	M down vs IM
mmu-miR-802-5p	0.0505769	-1.5772	M down vs IM
mmu-miR-1188-3p	0.0559955	-1.52003	M down vs IM
mmu-miR-16-1-3p	0.0767775	-1.30071	M down vs IM
mmu-miR-714	0.52857	-1.32211	M down vs IM
mmu-miR-8119	0.382979	-1.31591	M down vs IM
mmu-mir-5099	0	-1.59844	M down vs IM
mmu-miR-1982-5p	0.237432	-1.35751	M down vs IM
mmu-miR-544-3p	0.386821	-1.35728	M down vs IM
mmu-let-7e-5p	0.108006	3.10618	M up vs IM
mmu-mir-6978	0.284358	-1.40883	M down vs IM
mmu-miR-6914-3p	0.770044	1.11193	M up vs IM
mmu-miR-770-5p	0.273707	-1.38748	M down vs IM
mmu-miR-7229-3p	0.0589319	-1.40947	M down vs IM
mmu-mir-485	0.203144	-1.40883	M down vs IM
mmu-miR-7668-5p	0.543757	-1.10369	M down vs IM
mmu-miR-1931	0.179141	-2.00162	M down vs IM
mmu-mir-26a-1	0.311131	-1.27855	M down vs IM
mmu-miR-7045-5p	0.525193	1.36616	M up vs IM
mmu-miR-195a-3p	0.154881	1.61904	M up vs IM
mmu-miR-3971	0	-2.02105	M down vs IM
mmu-miR-5624-3p	0.0609297	-1.49932	M down vs IM

mmu-miR-3963	0.261462	-1.32211	M down vs IM
mmu-mir-8101	0.10399	-1.60335	M down vs IM
mmu-miR-6990-3p	0.341543	-1.28727	M down vs IM
mmu-mir-7066	0	-1.33616	M down vs IM
mmu-miR-210-3p	0.327444	3.78106	M up vs IM
mmu-miR-7020-5p	0.241715	-1.50892	M down vs IM
mmu-miR-3110-5p	0.32812	-1.33616	M down vs IM
mmu-mir-8101	0.148502	-1.38748	M down vs IM
mmu-mir-6374	0.237551	-1.35728	M down vs IM
mmu-miR-6917-5p	0.286977	-1.33906	M down vs IM
mmu-miR-345-3p	0.348853	-1.35728	M down vs IM
mmu-miR-6239	0.0908592	-1.39914	M down vs IM
mmu-miR-101c	0.40272	-1.30071	M down vs IM
mmu-miR-2861	0.184225	-1.31544	M down vs IM
mmu-miR-341-5p	0.305461	-1.30071	M down vs IM
mmu-mir-6926	0.212396	-1.48401	M down vs IM
mmu-miR-211-3p	0	-1.57953	M down vs IM
mmu-mir-6975	0.314128	-1.32211	M down vs IM
mmu-miR-7081-5p	0.242026	-2.62613	M down vs IM
mmu-mir-6935	0.73963	-1.08618	M down vs IM
mmu-miR-6922-5p	0.182702	-1.49962	M down vs IM
mmu-mir-484	0.0599902	-1.66951	M down vs IM
mmu-mir-195a	0.108958	-1.32211	M down vs IM
mmu-miR-7229-5p	0.299284	-1.32211	M down vs IM
mmu-mir-7654	0	-1.51758	M down vs IM
mmu-miR-34a-5p	0	1.05334	M up vs IM
mmu-mir-669n	0.221203	-1.33616	M down vs IM
mmu-miR-7092-5p	0.187575	-1.48302	M down vs IM
mmu-miR-379-3p	0.33865	-1.30071	M down vs IM
mmu-mir-6418	0.329871	-1.30071	M down vs IM
mmu-let-7i-5p	0.997665	-1.00119	M down vs IM
mmu-mir-301b	0.130001	-1.35728	M down vs IM
mmu-miR-339-5p	0.227684	-1.32423	M down vs IM
mmu-miR-182-5p	0.715596	-1.05156	M down vs IM
mmu-mir-8114	0.402714	-1.27623	M down vs IM
mmu-miR-338-5p	0.305461	-1.30071	M down vs IM
mmu-mir-501	0.181127	-1.27623	M down vs IM
mmu-mir-21a	0	-1.38748	M down vs IM
mmu-miR-351-5p	0	-1.38748	M down vs IM
mmu-miR-429-3p	0	-1.38748	M down vs IM
mmu-miR-6935-5p	0	-1.32211	M down vs IM
mmu-miR-216b-3p	0.0881485	-1.35728	M down vs IM
mmu-miR-760-3p	0.0767775	-1.30071	M down vs IM

mmu-miR-7220-3p	0.683023	1.09334	M up vs IM
mmu-miR-3472	0.377143	-1.27623	M down vs IM
mmu-miR-665-3p	0.152155	-1.64712	M down vs IM
mmu-miR-880-3p	0.0765801	-1.35728	M down vs IM
mmu-mir-3074-2	0.0765801	-1.35728	M down vs IM
mmu-miR-8095	0.53621	-1.12704	M down vs IM
mmu-miR-223-5p	0.208032	-1.27623	M down vs IM
mmu-miR-466m-5p	0.0710306	-1.32895	M down vs IM
mmu-miR-669m-5p	0.0710306	-1.32895	M down vs IM
mmu-miR-8110	0.607174	1.13733	M up vs IM
mmu-miR-3065-3p	0.0427104	-1.38748	M down vs IM
mmu-miR-7689-3p	0.331839	-1.81826	M down vs IM
mmu-miR-7088-3p	0.385857	-1.57381	M down vs IM
mmu-miR-196a-1-3p	0.421007	-1.21284	M down vs IM
mmu-mir-8120	0.313095	-1.27623	M down vs IM
mmu-miR-6950-3p	0.0806825	-1.33616	M down vs IM
mmu-miR-7017-5p	0.436106	-1.56688	M down vs IM
mmu-mir-99b	0.70223	-1.16685	M down vs IM
mmu-mir-3081	0	-1.35728	M down vs IM
mmu-mir-7210	0	-1.16145	M down vs IM
mmu-mir-23b	0	-1.35728	M down vs IM
mmu-mir-199a-1	0	-1.52003	M down vs IM
mmu-mir-221	0	-1.35728	M down vs IM
mmu-mir-7069	0	-1.50748	M down vs IM
mmu-mir-7077	0	-1.35728	M down vs IM
mmu-mir-7087	0	-1.35728	M down vs IM
mmu-miR-330-5p	0	-1.35728	M down vs IM
mmu-miR-381-5p	0	-1.50748	M down vs IM
mmu-miR-6516-3p	0	-1.50748	M down vs IM
mmu-miR-6908-3p	0	-1.35728	M down vs IM
mmu-miR-344g-5p	0	-1.29333	M down vs IM
mmu-miR-6931-3p	0.416448	-1.48216	M down vs IM
mmu-miR-8100	0.0762868	-1.49305	M down vs IM
mmu-miR-1843a-3p	0.0963172	-1.32211	M down vs IM
mmu-miR-881-3p	0	-1.30071	M down vs IM
mmu-miR-669l-3p	0.731023	-1.15179	M down vs IM
mmu-miR-7072-3p	0.816764	-1.09752	M down vs IM
mmu-miR-290a-3p	0.829952	-1.08983	M down vs IM
mmu-miR-21b	0.731023	-1.15179	M down vs IM
mmu-mir-3074-2	0.0837023	-1.32211	M down vs IM
mmu-miR-3473f	0.0837023	-1.32211	M down vs IM
mmu-mir-6401	0.35874	-1.25175	M down vs IM
mmu-miR-302b-5p	0.723011	-1.15179	M down vs IM

mmu-miR-27b-3p	0	-1.33906	M down vs IM
mmu-mir-130c	0.0500457	-1.47425	M down vs IM
mmu-mir-7007	0	-1.33616	M down vs IM
mmu-miR-7046-3p	0	-1.33616	M down vs IM
mmu-mir-692-1	0.347087	-1.36952	M down vs IM
mmu-mir-692-2	0.347087	-1.36952	M down vs IM
mmu-mir-692-3	0.347087	-1.36952	M down vs IM
mmu-miR-669k-5p	1	1	o change between M and IM
mmu-miR-325-5p	0.192972	-1.09209	M down vs IM
mmu-let-7a-1	0.70218	1.28214	M up vs IM
mmu-mir-1839	0.0153066	-1.26701	M down vs IM
mmu-miR-1194	0.160482	-1.60335	M down vs IM
mmu-miR-466d-3p	0.7372	-1.11822	M down vs IM
mmu-miR-669d-3p	0.56394	-1.25786	M down vs IM
mmu-miR-6991-3p	0	-1.32451	M down vs IM
mmu-mir-3072	0	-1.32423	M down vs IM
mmu-miR-590-3p	0	-1.32423	M down vs IM
mmu-miR-6975-5p	0	-1.49545	M down vs IM
mmu-miR-7664-5p	0	-1.32423	M down vs IM
mmu-mir-200a	0	-1.27623	M down vs IM
mmu-miR-6414	0.668624	-1.16171	M down vs IM
mmu-miR-7017-3p	0	-1.32211	M down vs IM
mmu-miR-7080-5p	0.646823	-1.09752	M down vs IM
mmu-miR-20a-3p	0.52417	-1.27623	M down vs IM
mmu-mir-1a-1	0.197438	-1.38748	M down vs IM
mmu-miR-31-3p	0	-1.25786	M down vs IM
mmu-mir-450a-1	0.189665	-1.09752	M down vs IM
mmu-miR-6968-5p	0.133622	1.52075	M up vs IM
mmu-miR-7049-3p	0.330437	-1.38748	M down vs IM
mmu-miR-18b-5p	0.679317	-1.15179	M down vs IM
mmu-miR-7679-3p	0.245373	-1.3388	M down vs IM
mmu-mir-32	0.777335	-1.09752	M down vs IM
mmu-miR-6337	0.226329	-1.38748	M down vs IM
mmu-miR-7001-5p	0.367997	2.25406	M up vs IM
mmu-mir-705	0	-1.30071	M down vs IM
mmu-mir-7688	0	-1.30071	M down vs IM
mmu-miR-187-5p	0	-1.30071	M down vs IM
mmu-miR-7119-3p	0	-1.42757	M down vs IM
mmu-miR-219c-5p	0	-1.30071	M down vs IM
mmu-mir-218-2	0.0956521	-1.27623	M down vs IM
mmu-mir-6372	0.0956521	-1.27623	M down vs IM
mmu-miR-1966-3p	0.0956521	-1.27623	M down vs IM
mmu-mir-671	0.11239	-1.22996	M down vs IM

mmu-miR-350-3p	0.0671337	-1.41745	M down vs IM
mmu-mir-3065	0.392635	-1.21284	M down vs IM
mmu-miR-149-3p	0.181054	-1.20443	M down vs IM
mmu-miR-6967-3p	0.0269357	-1.12704	M down vs IM
mmu-mir-719	0.578552	-1.07781	M down vs IM
mmu-miR-3109-3p	0.762934	-1.09209	M down vs IM
mmu-mir-3473a	0.608805	-1.16171	M down vs IM
mmu-mir-669f	0.226775	-1.38959	M down vs IM
mmu-miR-7064-5p	0.713504	-1.07524	M down vs IM
mmu-miR-23b-3p	0.162391	-1.30246	M down vs IM
mmu-mir-7650	0.626286	-1.15179	M down vs IM
mmu-miR-509-5p	0.945591	-1.00782	M down vs IM
mmu-miR-3072-3p	0	-1.42925	M down vs IM
mmu-miR-3092-3p	0	-1.44124	M down vs IM
mmu-miR-3473a	0	-1.27623	M down vs IM
mmu-miR-7008-5p	0	-1.27623	M down vs IM
mmu-miR-200c-5p	0	-1.27623	M down vs IM
mmu-mir-466i	0	-1.27623	M down vs IM
mmu-miR-7085-3p	0.783795	-1.33112	M down vs IM
mmu-miR-6974-5p	0.747645	-1.05346	M down vs IM
mmu-miR-10a-5p	0.776421	-1.06723	M down vs IM
mmu-mir-539	0.242868	-1.54176	M down vs IM
mmu-miR-7025-5p	0.916661	1.06555	M up vs IM
mmu-miR-3074-2-3p	0.0897361	-1.25175	M down vs IM
mmu-miR-671-3p	0.0897361	-1.25175	M down vs IM
mmu-miR-6962-5p	0.553725	-1.16171	M down vs IM
mmu-mir-207	0.447309	-1.1824	M down vs IM
mmu-mir-6965	0.441258	-1.15179	M down vs IM
mmu-miR-3475-3p	0.380935	-1.29026	M down vs IM
mmu-mir-381	0.546826	-1.16171	M down vs IM
mmu-mir-16-1	0.654519	-1.10407	M down vs IM
mmu-miR-6943-3p	0.252942	-1.56688	M down vs IM
mmu-miR-5119	0.257234	-1.55384	M down vs IM
mmu-mir-299a	0.484832	-1.18789	M down vs IM
mmu-miR-7663-5p	0.290862	-1.35728	M down vs IM
mmu-mir-6392	0.491349	-1.16685	M down vs IM
mmu-miR-758-3p	0.334227	-1.29597	M down vs IM
mmu-miR-7659-3p	0.376638	1.2665	M up vs IM
mmu-mir-381	0.565281	-1.15179	M down vs IM
mmu-miR-3073b-3p	0.513774	-1.16171	M down vs IM
mmu-mir-378d	0.523636	-1.07977	M down vs IM
mmu-miR-6971-5p	0.671701	-1.09752	M down vs IM
mmu-miR-1930-3p	0.916066	1.06229	M up vs IM

mmu-miR-7047-3p	0.509236	-1.16171	M down vs IM
mmu-mir-6987	0.126891	-1.75173	M down vs IM
mmu-mir-3473c	0.634561	-1.10178	M down vs IM
mmu-mir-6380	0.620687	-1.10697	M down vs IM
mmu-miR-466h-3p	0.892415	-1.09209	M down vs IM
mmu-mir-7033	0	-1.19958	M down vs IM
mmu-miR-1942	0	-1.03232	M down vs IM
mmu-miR-6990-5p	0	-1.40184	M down vs IM
mmu-mir-8112	0.799956	-1.03749	M down vs IM
mmu-mir-3086	0.547521	-1.15179	M down vs IM
mmu-miR-101b-3p	0.355344	-1.30071	M down vs IM
mmu-let-7k	0.365965	1.2884	M up vs IM
mmu-mir-218-1	0.658525	1.07589	M up vs IM
mmu-miR-329-5p	0.646823	-1.09752	M down vs IM
mmu-mir-3971	0.482516	-1.16171	M down vs IM
mmu-miR-6913-5p	0.482516	-1.16171	M down vs IM
mmu-miR-539-3p	0.335397	-1.27501	M down vs IM
mmu-miR-1a-1-5p	0.407794	-1.24637	M down vs IM
mmu-miR-7082-5p	0.546134	-1.14931	M down vs IM
mmu-miR-6976-3p	0.598984	-1.13502	M down vs IM
mmu-miR-7650-3p	0.850224	1.04349	M up vs IM
mmu-mir-676	0.973716	1.00622	M up vs IM
mmu-miR-7236-5p	0.629686	-1.10379	M down vs IM
mmu-mir-3073a	0.684946	-1.08426	M down vs IM
mmu-miR-1955-5p	0.788333	-1.09209	M down vs IM
mmu-miR-302c-3p	0.307973	-1.28235	M down vs IM
mmu-miR-7082-3p	0.392453	1.20528	M up vs IM
mmu-mir-3544	0.635095	-1.10178	M down vs IM
mmu-mir-7038	0.276143	-1.32211	M down vs IM
mmu-miR-1199-5p	0.260388	-1.31772	M down vs IM
mmu-miR-7118-3p	0.387407	-1.26569	M down vs IM
mmu-miR-3962	0.572027	-1.13959	M down vs IM
mmu-mir-5135	0.703839	-1.08589	M down vs IM
mmu-mir-7018	0.572027	-1.13959	M down vs IM
mmu-mir-499	0.783434	1.04945	M up vs IM
mmu-miR-7094-3p	0.5	-1.15179	M down vs IM
mmu-miR-7234-5p	0.366285	-1.24453	M down vs IM
mmu-miR-7213-3p	0.305461	-1.30071	M down vs IM
mmu-miR-411-3p	0.69328	1.06453	M up vs IM
mmu-miR-1839-3p	0.725848	-1.25356	M down vs IM
mmu-miR-7668-3p	0.24939	-1.30071	M down vs IM
mmu-miR-465d-5p	0.606024	-1.09752	M down vs IM
mmu-mir-147	0.303567	1.33734	M up vs IM

mmu-miR-6905-5p	0.329187	1.3022	M up vs IM
mmu-mir-470	0.544142	-1.13959	M down vs IM
mmu-miR-7682-5p	0.231778	-1.48302	M down vs IM
mmu-mir-6898	0.544142	-1.13959	M down vs IM
mmu-mir-6998	0.662251	-1.09209	M down vs IM
mmu-miR-193a-5p	0.544142	-1.13959	M down vs IM
mmu-mir-148b	0.330862	-1.29693	M down vs IM
mmu-mir-5103	0.635808	-1.09209	M down vs IM
mmu-mir-450a-1	0.503393	-1.23389	M down vs IM
mmu-miR-10b-5p	1	1	o change between M and IM
mmu-miR-7062-3p	0.526176	-1.13959	M down vs IM
mmu-mir-1900	0.646257	-1.09209	M down vs IM
mmu-mir-190a	0.271773	-1.30071	M down vs IM
mmu-mir-465a	0.39672	-1.08058	M down vs IM
mmu-mir-465b-1	0.39672	-1.08058	M down vs IM
mmu-mir-465b-2	0.39672	-1.08058	M down vs IM
mmu-mir-223	0.514634	-1.13959	M down vs IM
mmu-mir-7039	0.514634	-1.13959	M down vs IM
mmu-mir-6935	0.567131	-1.09752	M down vs IM
mmu-miR-466q	0.373275	-1.25786	M down vs IM
mmu-miR-7086-3p	0.251995	-1.48119	M down vs IM
mmu-miR-6982-5p	0.697909	-1.10407	M down vs IM
mmu-mir-3473g	0.495847	-1.13959	M down vs IM
mmu-miR-7059-3p	0.354527	-1.27276	M down vs IM
mmu-miR-7038-5p	0.571851	-1.12704	M down vs IM
mmu-let-7b-5p	0.307643	1.15251	M up vs IM
mmu-miR-7089-5p	0.441463	-1.27623	M down vs IM
mmu-mir-804	0	-1.10165	M down vs IM
mmu-miR-877-5p	0	-1.07893	M down vs IM
mmu-miR-1224-3p	0	-1.84458	M down vs IM
mmu-miR-741-5p	0	-1.78079	M down vs IM
mmu-mir-29b-1	0	-1.21272	M down vs IM
mmu-mir-181a-1	0	-1.56516	M down vs IM
mmu-mir-181a-1	0	-1.36952	M down vs IM
mmu-mir-8109	0	-1.21272	M down vs IM
mmu-miR-6417	0	-1.21272	M down vs IM
mmu-miR-7687-3p	0	-1.21272	M down vs IM
mmu-miR-876-5p	0.595821	-1.09209	M down vs IM
mmu-mir-551b	0.471851	-1.13959	M down vs IM
mmu-mir-7653	0.471851	-1.13959	M down vs IM
mmu-mir-7036b	0.232005	-1.2899	M down vs IM
mmu-miR-224-3p	0.691192	-1.10178	M down vs IM
mmu-miR-5112	0	-1.2076	M down vs IM

mmu-miR-496a-3p	0.330375	-1.30071	M down vs IM
mmu-mir-6389	0.124119	-1.09752	M down vs IM
mmu-miR-1231-3p	0.208299	-1.39637	M down vs IM
mmu-miR-181a-2-3p	0.27672	-1.27623	M down vs IM
mmu-miR-107-3p	0	-1.9313	M down vs IM
mmu-mir-350	0.36665	-1.10407	M down vs IM
mmu-miR-151-5p	0.716196	-1.16171	M down vs IM
mmu-mir-758	0.528149	-1.12704	M down vs IM
mmu-miR-3089-3p	0.411807	1.15311	M up vs IM
mmu-miR-1970	0.58475	1.0852	M up vs IM
mmu-miR-103-2-5p	0.676028	-1.10178	M down vs IM
mmu-mir-7037	0	-1.02818	M down vs IM
mmu-miR-7676-3p	0	-1.89385	M down vs IM
mmu-mir-762	0.724539	-1.09209	M down vs IM
mmu-miR-5101	1	1	o change between M and IM
mmu-miR-1936	0.692735	-1.09752	M down vs IM
mmu-mir-7231	0.680112	-1.09209	M down vs IM
mmu-mir-6896	0.482091	-1.27623	M down vs IM
mmu-miR-423-3p	0.654519	-1.10407	M down vs IM
mmu-miR-466g	0.875921	-1.05165	M down vs IM
mmu-miR-3091-5p	0.397737	-1.13959	M down vs IM
mmu-mir-7012	0.555453	-1.09209	M down vs IM
mmu-miR-6927-3p	0.671701	-1.09752	M down vs IM
mmu-miR-6945-3p	0.671701	-1.09752	M down vs IM
mmu-miR-7073-3p	0.671701	-1.09752	M down vs IM
mmu-miR-7665-3p	0.671701	-1.09752	M down vs IM
mmu-miR-181d-3p	0	-1.19113	M down vs IM
mmu-mir-202	0.198304	-1.16171	M down vs IM
mmu-mir-29b-1	0.125622	-1.21272	M down vs IM
mmu-miR-7069-5p	0.213317	-1.11187	M down vs IM
mmu-miR-214-5p	0.555453	-1.09209	M down vs IM
mmu-miR-136-5p	0.428112	-1.25175	M down vs IM
mmu-miR-6967-5p	0.707025	1.09334	M up vs IM
mmu-miR-7651-5p	0.176167	-1.16171	M down vs IM
mmu-miR-1934-5p	0.176167	-1.16171	M down vs IM
mmu-miR-742-3p	1	1	o change between M and IM
mmu-mir-300	0.686456	-1.09209	M down vs IM
mmu-mir-7094-1	0.717273	-1.08618	M down vs IM
mmu-mir-7094-2	0.717273	-1.08618	M down vs IM
mmu-miR-878-3p	0.717273	-1.08618	M down vs IM
mmu-miR-6539	0.673949	-1.27623	M down vs IM
mmu-mir-2861	0.227128	-1.15269	M down vs IM
mmu-miR-7070-5p	0.231713	-1.10178	M down vs IM

mmu-miR-6361	0.153767	-1.16171	M down vs IM
mmu-miR-1928	0.971492	1.00782	M up vs IM
mmu-mir-7064	0.437579	-1.09752	M down vs IM
mmu-let-7f-1	0.413071	-1.21897	M down vs IM
mmu-mir-434	0.646823	-1.09752	M down vs IM
mmu-miR-345-5p	0.715496	1.17463	M up vs IM
mmu-mir-7065	0.618434	-1.10178	M down vs IM
mmu-mir-6940	0.329376	-1.13959	M down vs IM
mmu-mir-682	0	-1.32211	M down vs IM
mmu-miR-7093-3p	0.115575	-1.30071	M down vs IM
mmu-miR-7007-5p	0.630435	-1.09752	M down vs IM
mmu-mir-467f	0.592465	-1.27623	M down vs IM
mmu-miR-874-5p	0.906852	1.04945	M up vs IM
mmu-miR-29b-1-5p	0.812615	-1.04658	M down vs IM
mmu-miR-433-3p	0.18627	-1.15179	M down vs IM
mmu-mir-331	0.144827	-1.20188	M down vs IM
mmu-mir-8091	0.18627	-1.15179	M down vs IM
mmu-miR-8111	0.377143	-1.27623	M down vs IM
mmu-miR-335-5p	1	1	o change between M and IM
mmu-mir-802	0.413402	1.0852	M up vs IM
mmu-miR-449a-3p	0.5	-1.08618	M down vs IM
mmu-mir-5615-2	0.162705	-1.15179	M down vs IM
mmu-miR-3070a-5p	0.162705	-1.15179	M down vs IM
mmu-miR-3070b-5p	0.162705	-1.15179	M down vs IM
mmu-miR-181b-2-3p	0.162705	-1.15179	M down vs IM
mmu-miR-6336	0.162705	-1.15179	M down vs IM
mmu-mir-6347	0.162705	-1.15179	M down vs IM
mmu-miR-6920-3p	0.162705	-1.15179	M down vs IM
mmu-miR-5621-5p	0.430613	-1.57775	M down vs IM
mmu-miR-7036-3p	0.559782	-1.10407	M down vs IM
mmu-miR-1247-3p	0.254886	-1.13959	M down vs IM
mmu-miR-7005-5p	0.208338	1.09841	M up vs IM
mmu-miR-763	0.474888	-1.08618	M down vs IM
mmu-miR-7061-5p	0.833893	-1.04091	M down vs IM
mmu-miR-677-5p	0.266314	-1.40184	M down vs IM
mmu-miR-7042-3p	0.679639	-1.08618	M down vs IM
mmu-miR-124-3p	0.683603	-1.08486	M down vs IM
mmu-miR-1251-3p	0.0767775	-1.30071	M down vs IM
mmu-miR-410-5p	0.988845	1.00056	M up vs IM
mmu-miR-3075-3p	0.141174	-1.15179	M down vs IM
mmu-miR-106a-3p	0.887758	1.0576	M up vs IM
mmu-miR-5627-5p	0.566387	-1.10178	M down vs IM
mmu-mir-653	0.635808	-1.09209	M down vs IM

mmu-miR-32-3p	0.437122	1.09334	M up vs IM
mmu-miR-6997-3p	0.225245	-1.13959	M down vs IM
mmu-miR-7089-3p	0.119052	1.15226	M up vs IM
mmu-miR-802-3p	0.475392	1.05325	M up vs IM
mmu-miR-295-5p	0.438417	-1.59898	M down vs IM
mmu-mir-5626	0	-1.33616	M down vs IM
mmu-miR-31-5p	0	-1.16685	M down vs IM
mmu-miR-297c-5p	0	-1.28065	M down vs IM
mmu-miR-1192	0	-1.16685	M down vs IM
mmu-miR-181c-3p	0	-1.27431	M down vs IM
mmu-miR-5107-5p	0	-1.5427	M down vs IM
mmu-miR-6909-3p	0	-1.16685	M down vs IM
mmu-miR-6981-5p	0	-1.16685	M down vs IM
mmu-miR-341-3p	1	1	o change between M and IM
mmu-miR-7230-5p	0.386483	-1.22662	M down vs IM
mmu-mir-7660	0.270681	-1.29693	M down vs IM
mmu-mir-30f	0.547592	-1.30071	M down vs IM
mmu-mir-874	0.911851	1.04349	M up vs IM
mmu-miR-470-5p	0.0182044	-1.16685	M down vs IM
mmu-miR-873a-5p	0.890208	1.05439	M up vs IM
mmu-miR-5129-5p	0.200507	-1.13959	M down vs IM
mmu-mir-5112	0	1.54593	M up vs IM
mmu-mir-5616	0	-1.11329	M down vs IM
mmu-mir-6982	0	1.23702	M up vs IM
mmu-mir-7212	0	-1.16171	M down vs IM
mmu-mir-7679	0	-1.16171	M down vs IM
mmu-miR-331-3p	0	-1.16171	M down vs IM
mmu-miR-200b-5p	0	-1.49932	M down vs IM
mmu-miR-1947-5p	0	-1.10178	M down vs IM
mmu-miR-1249-5p	0	-1.16171	M down vs IM
mmu-miR-3063-5p	0	-1.31191	M down vs IM
mmu-miR-3085-3p	0	-1.32211	M down vs IM
mmu-miR-6929-5p	0	-1.29026	M down vs IM
mmu-miR-6965-3p	0	-1.11329	M down vs IM
mmu-miR-7009-5p	0	1.31188	M up vs IM
mmu-miR-7035-5p	0	-1.16171	M down vs IM
mmu-miR-7647-5p	0	-1.26183	M down vs IM
mmu-mir-133b	0.88334	1.05686	M up vs IM
mmu-miR-7091-5p	0.0925308	-1.28693	M down vs IM
mmu-mir-741	0.270056	1.0852	M up vs IM
mmu-mir-5104	0.0956521	-1.27623	M down vs IM
mmu-miR-3097-3p	0.175335	-1.13959	M down vs IM
mmu-miR-190b-5p	0.175335	-1.13959	M down vs IM

mmu-miR-6933-3p	0.175335	-1.13959	M down vs IM
mmu-mir-196b	0.528903	1.05439	M up vs IM
mmu-miR-6393	0.427551	-1.0689	M down vs IM
mmu-mir-6929	0.839645	-1.03375	M down vs IM
mmu-mir-467a-3	0.641391	-1.08486	M down vs IM
mmu-mir-467a-6	0.641391	-1.08486	M down vs IM
mmu-mir-467a-10	0.641391	-1.08486	M down vs IM
mmu-mir-344g	0.0373068	-1.15179	M down vs IM
mmu-miR-449c-3p	0.631947	-1.26979	M down vs IM
mmu-miR-672-5p	0.33641	-1.08618	M down vs IM
mmu-miR-19a-5p	1	1	o change between M and IM
mmu-miR-107-5p	1	1	o change between M and IM
mmu-mir-7214	0.843946	-1.09209	M down vs IM
mmu-miR-7646-5p	0.337205	-1.22662	M down vs IM
mmu-mir-6338	0.764827	1.04739	M up vs IM
mmu-miR-1963	0.373992	-1.19674	M down vs IM
mmu-mir-541	1	1	o change between M and IM
mmu-mir-375	0.56675	-1.04658	M down vs IM
mmu-miR-741-3p	0	-1.15179	M down vs IM
mmu-miR-7039-3p	0	-1.27925	M down vs IM
mmu-miR-7058-5p	0	-1.15179	M down vs IM
mmu-mir-431	0	-1.24954	M down vs IM
mmu-mir-494	0	-1.2899	M down vs IM
mmu-mir-369	0	-1.15179	M down vs IM
mmu-mir-374b	0	-1.15179	M down vs IM
mmu-mir-667	0	-1.30071	M down vs IM
mmu-mir-670	0	-1.2899	M down vs IM
mmu-mir-466c-1	0	-1.15179	M down vs IM
mmu-mir-1901	0	-1.25105	M down vs IM
mmu-mir-669m-2	0	-1.26412	M down vs IM
mmu-mir-1951	0	-1.15179	M down vs IM
mmu-mir-3064	0	-1.10379	M down vs IM
mmu-mir-5621	0	-1.09238	M down vs IM
mmu-mir-5627	0	-1.26903	M down vs IM
mmu-mir-6386	0	-1.09752	M down vs IM
mmu-mir-6910	0	-1.48652	M down vs IM
mmu-mir-7649	0	-1.15179	M down vs IM
mmu-mir-126b	0	-1.15179	M down vs IM
mmu-mir-8108	0	-1.15179	M down vs IM
mmu-mir-8111	0	-1.15179	M down vs IM
mmu-mir-8111	0	-1.15179	M down vs IM
mmu-miR-129-5p	0	-1.15179	M down vs IM
mmu-miR-383-5p	0	-1.27925	M down vs IM

mmu-miR-410-3p	0	1.10787	M up vs IM
mmu-miR-489-3p	0	-1.15179	M down vs IM
mmu-miR-547-3p	0	-1.15179	M down vs IM
mmu-miR-488-5p	0	1.32317	M up vs IM
mmu-miR-186-3p	0	-1.15179	M down vs IM
mmu-let-7b-3p	0	-1.15179	M down vs IM
mmu-miR-871-5p	0	-1.31082	M down vs IM
mmu-miR-466f	0	-1.46995	M down vs IM
mmu-miR-1251-5p	0	-1.30071	M down vs IM
mmu-miR-3063-3p	0	-1.15179	M down vs IM
mmu-let-7e-3p	0	1.09334	M up vs IM
mmu-miR-487b-5p	0	-1.15179	M down vs IM
mmu-miR-5136	0	-1.15179	M down vs IM
mmu-miR-6918-3p	0	-1.2899	M down vs IM
mmu-miR-6929-3p	0	1.09334	M up vs IM
mmu-miR-6942-5p	0	-1.09752	M down vs IM
mmu-miR-7070-3p	0	1.32317	M up vs IM
mmu-miR-7072-5p	0	-1.47077	M down vs IM
mmu-miR-6546-5p	0	-1.15179	M down vs IM
mmu-miR-7662-3p	0	1.55924	M up vs IM
mmu-miR-7675-5p	0	-1.2899	M down vs IM
mmu-miR-8105	0	-1.10379	M down vs IM
mmu-mir-297a-1	0	-1.15179	M down vs IM
mmu-miR-3078-5p	0	-1.09209	M down vs IM
mmu-miR-7024-3p	0.0762951	-1.42407	M down vs IM
mmu-mir-3087	0.217711	-1.12704	M down vs IM
mmu-miR-150-3p	1	1	o change between M and IM
mmu-miR-7222-3p	0	-1.25515	M down vs IM
mmu-miR-7669-5p	0	-1.14931	M down vs IM
mmu-mir-219b	0.284598	-1.08007	M down vs IM
mmu-let-7d-5p	0.0401429	1.57573	M up vs IM
mmu-miR-6402	0.787628	1.09841	M up vs IM
mmu-miR-6389	0.88774	1.04945	M up vs IM
mmu-miR-7006-5p	0.521402	-1.09209	M down vs IM
mmu-miR-7688-3p	0.359847	-1.20749	M down vs IM
mmu-miR-98-5p	0.0910086	1.23668	M up vs IM
mmu-mir-429	1	1	o change between M and IM
mmu-miR-134-3p	0.0874256	1.2593	M up vs IM
mmu-miR-6369	0.818205	-1.09209	M down vs IM
mmu-miR-770-3p	0.910687	1.03762	M up vs IM
mmu-mir-542	1	1	o change between M and IM
mmu-mir-7648	0	-1.47077	M down vs IM
mmu-miR-6948-5p	0	-1.25072	M down vs IM

mmu-mir-194-1	0	-1.45437	M down vs IM
mmu-mir-194-1	0	-1.50392	M down vs IM
mmu-let-7b	0	-1.13959	M down vs IM
mmu-mir-7a-2	0	-1.23629	M down vs IM
mmu-mir-574	0	-1.13959	M down vs IM
mmu-mir-467e	0	-1.27623	M down vs IM
mmu-mir-1930	0	-1.2378	M down vs IM
mmu-mir-1957a	0	-1.13959	M down vs IM
mmu-mir-1961	0	-1.27623	M down vs IM
mmu-mir-2136	0	-1.26569	M down vs IM
mmu-mir-599	0	1.0852	M up vs IM
mmu-mir-3062	0	1.03762	M up vs IM
mmu-mir-466o	0	1.04349	M up vs IM
mmu-mir-1231	0	1.02203	M up vs IM
mmu-mir-3473e	0	1.06404	M up vs IM
mmu-mir-7003	0	-1.13959	M down vs IM
mmu-mir-7005	0	-1.09209	M down vs IM
mmu-miR-295-3p	0	-1.09827	M down vs IM
mmu-miR-376a-3p	0	-1.13959	M down vs IM
mmu-miR-499-5p	0	-1.13959	M down vs IM
mmu-miR-30c-1-3p	0	-1.27623	M down vs IM
mmu-miR-29a-5p	0	-1.09209	M down vs IM
mmu-miR-879-5p	0	1.02547	M up vs IM
mmu-miR-872-3p	0	-1.13959	M down vs IM
mmu-miR-1190	0	-1.22756	M down vs IM
mmu-miR-3060-3p	0	-1.13959	M down vs IM
mmu-miR-3064-5p	0	-1.27623	M down vs IM
mmu-miR-3065-5p	0	-1.13959	M down vs IM
mmu-miR-3087-3p	0	-1.09209	M down vs IM
mmu-miR-190a-3p	0	-1.13959	M down vs IM
mmu-miR-496a-5p	0	-1.09209	M down vs IM
mmu-miR-5114	0	1.1611	M up vs IM
mmu-miR-6374	0	-1.13959	M down vs IM
mmu-miR-6916-5p	0	-1.09209	M down vs IM
mmu-miR-6930-5p	0	-1.29026	M down vs IM
mmu-miR-7007-3p	0	-1.27623	M down vs IM
mmu-miR-7068-5p	0	-1.09209	M down vs IM
mmu-miR-7091-3p	0	-1.10178	M down vs IM
mmu-miR-7651-3p	0	-1.24453	M down vs IM
mmu-miR-7036b-3p	0	-1.28693	M down vs IM
mmu-miR-126b-3p	0	-1.13959	M down vs IM
mmu-mir-467b	0.491007	-1.17836	M down vs IM
mmu-mir-382	0.985976	1.0071	M up vs IM

mmu-mir-6948	0.0183533	-1.09209	M down vs IM
mmu-miR-6951-3p	0.735235	-1.1199	M down vs IM
mmu-miR-467e-3p	0.786783	1.09334	M up vs IM
mmu-miR-7652-3p	0.652375	1.16861	M up vs IM
mmu-let-7c-1	0.12198	-1.08618	M down vs IM
mmu-miR-19a-3p	0.588106	1.21257	M up vs IM
mmu-mir-7046	1	1	o change between M and IM
mmu-miR-7074-5p	0.777335	-1.09752	M down vs IM
mmu-miR-329-3p	1	1	o change between M and IM
mmu-miR-302a-3p	1	1	o change between M and IM
mmu-miR-7225-3p	1	1	o change between M and IM
mmu-mir-677	0.714281	1.21178	M up vs IM
mmu-miR-497-3p	0.0770067	-1.09209	M down vs IM
mmu-mir-29b-2	0.143573	1.04945	M up vs IM
mmu-miR-764-3p	0.937692	1.03105	M up vs IM
mmu-miR-5617-5p	0	-1.09209	M down vs IM
mmu-miR-7117-3p	0.579891	-1.27623	M down vs IM
mmu-mir-3098	0.462693	-1.42407	M down vs IM
mmu-miR-3098-3p	0.15715	-1.23389	M down vs IM
mmu-miR-3070b-3p	0.318351	-1.10178	M down vs IM
mmu-mir-582	0.533809	-1.21272	M down vs IM
mmu-miR-29a-3p	0.895615	1.06244	M up vs IM
mmu-mir-30c-2	0.5826	-1.27623	M down vs IM
mmu-miR-8117	0.471318	-1.25175	M down vs IM
mmu-miR-3098-5p	0.0440521	-1.08486	M down vs IM
mmu-miR-5620-5p	0.255293	1.03762	M up vs IM
mmu-miR-7212-3p	0.85149	-1.08618	M down vs IM
mmu-miR-1897-5p	0.294097	-1.10178	M down vs IM
mmu-mir-5622	0	-1.07032	M down vs IM
mmu-miR-449b	1	1	o change between M and IM
mmu-miR-879-3p	0	-1.22417	M down vs IM
mmu-mir-185	0	1.07589	M up vs IM
mmu-mir-370	0	-1.08007	M down vs IM
mmu-mir-7028	0	-1.19313	M down vs IM
mmu-miR-485-3p	0	-1.25175	M down vs IM
mmu-miR-682	0	-1.19605	M down vs IM
mmu-miR-1187	0	-1.12704	M down vs IM
mmu-miR-3073a-5p	0	-1.08618	M down vs IM
mmu-miR-3075-5p	0	1.07589	M up vs IM
mmu-miR-7240-3p	0	1.05564	M up vs IM
mmu-miR-7675-3p	0	-1.12704	M down vs IM
mmu-mir-7031	0.304582	-1.09752	M down vs IM
mmu-miR-5130	0	1.03202	M up vs IM

mmu-miR-1941-3p	0.68081	-1.12704	M down vs IM
mmu-miR-687	0.231713	-1.10178	M down vs IM
mmu-mir-6391	1	1	o change between M and IM
mmu-miR-26a-1-3p	0	-1.08618	M down vs IM
mmu-mir-6943	0.431041	-1.46335	M down vs IM
mmu-mir-6979	0.884182	-1.08618	M down vs IM
mmu-mir-1247	0.0939946	-1.27623	M down vs IM
mmu-miR-467f	1	1	o change between M and IM
mmu-miR-509-3p	0.548429	1.21257	M up vs IM
mmu-miR-6977-3p	0.459474	-1.29062	M down vs IM
mmu-mir-675	0.85982	-1.06741	M down vs IM
mmu-mir-466b-8	0.319065	-1.09209	M down vs IM
mmu-miR-6955-5p	0.319065	-1.09209	M down vs IM
mmu-mir-6384	0.736618	-1.14493	M down vs IM
mmu-miR-7677-5p	0.240482	-1.09753	M down vs IM
mmu-miR-6954-5p	0.240482	-1.09752	M down vs IM
mmu-mir-6236	0.240482	-1.09752	M down vs IM
mmu-mir-6903	0.0627874	-1.50392	M down vs IM
mmu-mir-294	1	1	o change between M and IM
mmu-miR-3069-5p	1	1	o change between M and IM
mmu-mir-7240	0.286575	-1.09209	M down vs IM
mmu-miR-6993-3p	0.286575	-1.09209	M down vs IM
mmu-mir-450b	0.441771	-1.0525	M down vs IM
mmu-mir-467a-1	0.286575	-1.09209	M down vs IM
mmu-miR-182-3p	0.286575	-1.09209	M down vs IM
mmu-mir-3069	0.338361	-1.32211	M down vs IM
mmu-miR-1957b	0.975902	-1.01005	M down vs IM
mmu-mir-3964	1	1	o change between M and IM
mmu-miR-216a-5p	0.578817	-1.25175	M down vs IM
mmu-miR-292b-5p	0	-1.30071	M down vs IM
mmu-miR-7223-5p	0.942939	1.02378	M up vs IM
mmu-miR-669a-5p	0.33641	-1.08618	M down vs IM
mmu-miR-669p-5p	0.33641	-1.08618	M down vs IM
mmu-mir-217	0.252683	-1.09209	M down vs IM
mmu-mir-5108	0.252683	-1.09209	M down vs IM
mmu-mir-16-2	0.679738	1.124	M up vs IM
mmu-mir-7043	0.874088	1.04349	M up vs IM
mmu-mir-1931	1	1	o change between M and IM
mmu-miR-6940-3p	0.927043	1.04945	M up vs IM
mmu-mir-3108	0.302744	-1.08618	M down vs IM
mmu-miR-20b-5p	0.302744	-1.08618	M down vs IM
mmu-miR-7115-5p	0.0942819	-1.25175	M down vs IM
mmu-miR-92b-5p	0.784146	1.05439	M up vs IM

mmu-miR-7239-3p	0.113856	-1.22662	M down vs IM
mmu-mir-681	0.267436	-1.08618	M down vs IM
mmu-mir-7052	0.267436	-1.08618	M down vs IM
mmu-mir-669a-1	0.267436	-1.08618	M down vs IM
mmu-mir-669a-2	0.267436	-1.08618	M down vs IM
mmu-mir-466b-4	0.267436	-1.08618	M down vs IM
mmu-mir-466b-6	0.267436	-1.08618	M down vs IM
mmu-mir-411	0.267436	-1.08618	M down vs IM
mmu-mir-669a-4	0.267436	-1.08618	M down vs IM
mmu-mir-669a-5	0.267436	-1.08618	M down vs IM
mmu-mir-669a-6	0.267436	-1.08618	M down vs IM
mmu-mir-669a-7	0.267436	-1.08618	M down vs IM
mmu-mir-669a-8	0.267436	-1.08618	M down vs IM
mmu-mir-669a-9	0.267436	-1.08618	M down vs IM
mmu-mir-669a-10	0.267436	-1.08618	M down vs IM
mmu-mir-669a-11	0.267436	-1.08618	M down vs IM
mmu-mir-669a-12	0.267436	-1.08618	M down vs IM
mmu-mir-5615-2	0.267436	-1.08618	M down vs IM
mmu-mir-8105	0.267436	-1.08618	M down vs IM
mmu-miR-9-5p	0.267436	-1.08618	M down vs IM
mmu-miR-1961	0.267436	-1.08618	M down vs IM
mmu-miR-6966-3p	0.267436	-1.08618	M down vs IM
mmu-miR-6715-5p	0.267436	-1.08618	M down vs IM
mmu-mir-6911	0.343564	1.23668	M up vs IM
mmu-miR-877-3p	0.114196	-1.23695	M down vs IM
mmu-miR-701-5p	0.599899	1.0852	M up vs IM
mmu-mir-128-2	1	1	o change between M and IM
mmu-mir-3078	0.561711	-1.17206	M down vs IM
mmu-mir-7662	0	-1.23645	M down vs IM
mmu-miR-193b-5p	0	-1.10407	M down vs IM
mmu-miR-7056-3p	0	-1.10407	M down vs IM
mmu-mir-6355	0.039269	-1.40947	M down vs IM
mmu-miR-6397	0.234203	-1.08618	M down vs IM
mmu-miR-6976-5p	0.752027	-1.10407	M down vs IM
mmu-miR-103-3p	0.470564	-1.19616	M down vs IM
mmu-miR-7219-5p	0.344796	1.05439	M up vs IM
mmu-mir-3102	0.810713	1.0852	M up vs IM
mmu-miR-153-3p	1	1	o change between M and IM
mmu-mir-3093	0	-1.10178	M down vs IM
mmu-mir-5133	0	-1.39216	M down vs IM
mmu-miR-409-3p	0	-1.10178	M down vs IM
mmu-miR-448-5p	0.304636	1.2593	M up vs IM
mmu-miR-3535	1	1	o change between M and IM

mmu-miR-6964-5p	1	1	o change between M and IM
mmu-miR-7674-3p	1	1	o change between M and IM
mmu-miR-669a-3-3p	0.742058	-1.08618	M down vs IM
mmu-miR-7670-3p	0.711495	1.09841	M up vs IM
mmu-mir-188	1	1	o change between M and IM
mmu-miR-5113	0.86611	-1.09752	M down vs IM
mmu-miR-98-3p	1	1	o change between M and IM
mmu-miR-337-3p	1	1	o change between M and IM
mmu-miR-6538	0.00606968	-1.05064	M down vs IM
mmu-miR-367-3p	0.788333	-1.09209	M down vs IM
mmu-mir-295	0.806739	-1.08301	M down vs IM
mmu-miR-675-3p	0.718425	1.04945	M up vs IM
mmu-mir-497	0	-1.09752	M down vs IM
mmu-mir-3063	0	-1.09752	M down vs IM
mmu-mir-466p	0	-1.09752	M down vs IM
mmu-mir-3969	0	-1.09752	M down vs IM
mmu-mir-7651	0	-1.09752	M down vs IM
mmu-miR-1a-3p	0	-1.09752	M down vs IM
mmu-miR-1193-5p	0	-1.09752	M down vs IM
mmu-miR-669e-3p	0	-1.09752	M down vs IM
mmu-miR-6984-3p	0	-1.21897	M down vs IM
mmu-miR-7216-5p	0	-1.09752	M down vs IM
mmu-miR-7094b-2-5p	0.93929	1.06229	M up vs IM
mmu-miR-3067-5p	0.815542	-1.1199	M down vs IM
mmu-miR-467d-3p	0.866149	1.07589	M up vs IM
mmu-miR-463-5p	0.831929	-1.1293	M down vs IM
mmu-mir-6382	0.31744	1.21257	M up vs IM
mmu-mir-465a	1	1	o change between M and IM
mmu-miR-6396	1	1	o change between M and IM
mmu-mir-6342	0.711437	1.07589	M up vs IM
mmu-mir-19b-1	0.768815	-1.10178	M down vs IM
mmu-miR-3475-5p	0.725328	1.0852	M up vs IM
mmu-miR-292-3p	0.815496	1.05439	M up vs IM
mmu-mir-328	0.815496	1.05439	M up vs IM
mmu-miR-7025-3p	0.815496	1.05439	M up vs IM
mmu-miR-3068-3p	1	1	o change between M and IM
mmu-mir-3474	0.188347	1.34263	M up vs IM
mmu-mir-10a	0.811283	-1.08618	M down vs IM
mmu-mir-19a	0	-1.09209	M down vs IM
mmu-mir-695	0	-1.09209	M down vs IM
mmu-mir-208b	0	-1.09209	M down vs IM
mmu-mir-1971	0	-1.09209	M down vs IM
mmu-mir-6416	0	-1.09209	M down vs IM

mmu-mir-7228	0	-1.09209	M down vs IM
mmu-mir-7667	0	-1.09209	M down vs IM
mmu-miR-493-5p	0	-1.09209	M down vs IM
mmu-miR-6537-5p	0	-1.09209	M down vs IM
mmu-miR-6901-3p	0	-1.09209	M down vs IM
mmu-miR-7071-3p	0	-1.09209	M down vs IM
mmu-mir-7089	0.682591	1.0852	M up vs IM
mmu-mir-322	0.534595	1.16861	M up vs IM
mmu-miR-136-3p	0.847026	1.04349	M up vs IM
mmu-miR-7000-5p	0.670263	-1.10407	M down vs IM
mmu-mir-130a	1	1	o change between M and IM
mmu-miR-7664-3p	1	1	o change between M and IM
mmu-mir-29b-2	1	1	o change between M and IM
mmu-mir-344d-1	0	-1.43866	M down vs IM
mmu-miR-199b-5p	0.415415	-1.301	M down vs IM
mmu-mir-297a-3	0.453211	-1.16171	M down vs IM
mmu-mir-1894	0.790245	1.08203	M up vs IM
mmu-mir-6516	0.266468	-1.2899	M down vs IM
mmu-miR-7671-3p	0.606146	-1.12853	M down vs IM
mmu-miR-1957a	0.217164	1.3022	M up vs IM
mmu-miR-7658-3p	0.311486	-1.33616	M down vs IM
mmu-mir-669a-3	0	-1.08618	M down vs IM
mmu-mir-6406	0	-1.19695	M down vs IM
mmu-mir-7226	0	-1.08618	M down vs IM
mmu-miR-101a-3p	0	-1.04091	M down vs IM
mmu-miR-291a-3p	0	-1.08618	M down vs IM
mmu-miR-381-3p	0	-1.0468	M down vs IM
mmu-miR-1897-3p	0	-1.08618	M down vs IM
mmu-miR-3058-5p	0	-1.08618	M down vs IM
mmu-miR-3106-5p	0	-1.08618	M down vs IM
mmu-miR-3090-3p	0	-1.22662	M down vs IM
mmu-miR-194-1-3p	0	-1.08618	M down vs IM
mmu-miR-101b-5p	0	-1.08618	M down vs IM
mmu-miR-486-3p	0	-1.08618	M down vs IM
mmu-miR-208b-5p	0	-1.08618	M down vs IM
mmu-miR-6905-3p	0	-1.08618	M down vs IM
mmu-miR-6946-3p	0	1.07589	M up vs IM
mmu-miR-7087-5p	0	-1.08618	M down vs IM
mmu-miR-7681-3p	0	-1.08618	M down vs IM
mmu-miR-7685-5p	0	-1.08618	M down vs IM
mmu-miR-8096	0	-1.08618	M down vs IM
mmu-miR-8097	0	-1.08618	M down vs IM
mmu-mir-532	0.666206	1.09334	M up vs IM

mmu-miR-99b-5p	0.160374	1.89237	M up vs IM
mmu-miR-7646-3p	0.234517	1.53005	M up vs IM
mmu-miR-487b-3p	1	1	o change between M and IM
mmu-mir-6385	0.37733	1.27603	M up vs IM
mmu-miR-542-5p	1	1	o change between M and IM
mmu-miR-10b-3p	0.516611	1.16861	M up vs IM
mmu-miR-1258-5p	1	1	o change between M and IM
mmu-miR-299b-5p	0.817972	1.04945	M up vs IM
mmu-miR-8113	1	1	o change between M and IM
mmu-mir-146a	0.817972	1.04945	M up vs IM
mmu-mir-124-2	0.817972	1.04945	M up vs IM
mmu-mir-7115	0.671701	-1.09752	M down vs IM
mmu-mir-1264	0.838471	1.04349	M up vs IM
mmu-miR-467a-5p	0.838471	1.04349	M up vs IM
mmu-miR-2183	0.838471	1.04349	M up vs IM
mmu-miR-23a-5p	0.594148	-1.1293	M down vs IM
mmu-mir-5124b	1	1	o change between M and IM
mmu-miR-7650-5p	1	1	o change between M and IM
mmu-mir-292	1	1	o change between M and IM
mmu-miR-139-5p	1	1	o change between M and IM
mmu-miR-139-3p	1	1	o change between M and IM
mmu-miR-367-5p	1	1	o change between M and IM
mmu-mir-193b	0.706789	-1.08486	M down vs IM
mmu-mir-7684	0.47111	-1.08618	M down vs IM
mmu-mir-30a	0	-1.08486	M down vs IM
mmu-mir-698	0	-1.0289	M down vs IM
mmu-mir-92b	0	-1.08486	M down vs IM
mmu-mir-1954	0	-1.08486	M down vs IM
mmu-mir-1954	0	-1.08486	M down vs IM
mmu-miR-466a-5p	?	1	o change between M and IM
mmu-miR-574-5p	0	1.23668	M up vs IM
mmu-miR-6406	0	-1.20491	M down vs IM
mmu-miR-7672-5p	0	-1.21494	M down vs IM
mmu-mir-135b	0.706368	1.04945	M up vs IM
mmu-miR-219a-1-3p	1	1	o change between M and IM
mmu-miR-1a-2-5p	0.653496	-1.27623	M down vs IM
mmu-mir-5119	1	1	o change between M and IM
mmu-miR-134-5p	1	1	o change between M and IM
mmu-mir-3107	1	1	o change between M and IM
mmu-miR-196b-5p	1	1	o change between M and IM
mmu-mir-668	0.244555	-1.29646	M down vs IM
mmu-miR-26a-5p	0.396392	-1.12898	M down vs IM
mmu-mir-1932	0.716155	-1.09752	M down vs IM

mmu-let-7a-1	0.320625	1.32317	M up vs IM
mmu-miR-667-3p	0.642934	-1.10178	M down vs IM
mmu-mir-5106	1	1	o change between M and IM
mmu-miR-5135	1	1	o change between M and IM
mmu-miR-6953-5p	0.471826	-1.15179	M down vs IM
mmu-mir-142	0.434752	1.2984	M up vs IM
mmu-miR-6769b-5p	0	2.02217	M up vs IM
mmu-mir-6897	1	1	o change between M and IM
mmu-mir-467d	0.386426	1.2414	M up vs IM
mmu-miR-7688-5p	0.775366	1.05686	M up vs IM
mmu-miR-743a-5p	0.662251	-1.09209	M down vs IM
mmu-miR-6999-3p	0.352387	1.27507	M up vs IM
mmu-miR-29b-2-5p	0.351509	1.27603	M up vs IM
mmu-mir-122	1	1	o change between M and IM
mmu-miR-6938-5p	0.682591	1.0852	M up vs IM
mmu-miR-1912-3p	0.824141	1.04349	M up vs IM
mmu-mir-103-1	0.784147	1.05439	M up vs IM
mmu-mir-7118	0.588665	-1.1199	M down vs IM
mmu-miR-6962-3p	0.802038	1.04945	M up vs IM
mmu-miR-7079-5p	1	1	o change between M and IM
mmu-mir-467a-2	1	1	o change between M and IM
mmu-mir-467a-4	1	1	o change between M and IM
mmu-mir-467a-5	1	1	o change between M and IM
mmu-mir-467a-7	1	1	o change between M and IM
mmu-mir-467a-8	1	1	o change between M and IM
mmu-mir-467a-9	1	1	o change between M and IM
mmu-miR-881-5p	1	1	o change between M and IM
mmu-miR-143-5p	1	1	o change between M and IM
mmu-miR-6925-3p	1	1	o change between M and IM
mmu-mir-201	0.351509	1.27603	M up vs IM
mmu-mir-3099	0.367526	1.2593	M up vs IM
mmu-miR-3057-3p	1	1	o change between M and IM
mmu-miR-365-1-5p	0.338687	-1.29062	M down vs IM
mmu-mir-466f-3	0.643257	1.09881	M up vs IM
mmu-miR-6373	0.802038	1.04945	M up vs IM
mmu-mir-376c	1	1	o change between M and IM
mmu-mir-1948	1	1	o change between M and IM
mmu-miR-92b-3p	0.328684	1.3022	M up vs IM
mmu-mir-7032	1	1	o change between M and IM
mmu-mir-7055	0.646299	-1.09752	M down vs IM
mmu-mir-7078	0.578339	1.124	M up vs IM
mmu-mir-3960	0.710976	1.07589	M up vs IM
mmu-mir-466f-4	0	-1.0772	M down vs IM

mmu-miR-7002-3p	0.17233	-1.50392	M down vs IM
mmu-miR-6987-5p	0.658683	-1.13959	M down vs IM
mmu-let-7f-5p	0.807065	1.0852	M up vs IM
mmu-miR-6358	0.619751	-1.09752	M down vs IM
mmu-mir-1942	0.773095	1.0744	M up vs IM
mmu-mir-216a	0.436078	1.26608	M up vs IM
mmu-miR-669f-5p	0.73497	1.09848	M up vs IM
mmu-miR-743b-3p	0.496796	-1.15179	M down vs IM
mmu-miR-7031-3p	0.100854	-1.23329	M down vs IM
mmu-miR-290b-3p	0.667176	1.0852	M up vs IM
mmu-miR-468-5p	0.611222	-1.10441	M down vs IM
mmu-miR-6339	1	1	o change between M and IM
mmu-mir-6414	1	1	o change between M and IM
mmu-miR-3100-5p	1	1	o change between M and IM
mmu-mir-1251	1	1	o change between M and IM
mmu-mir-3070a	1	1	o change between M and IM
mmu-mir-3070b	1	1	o change between M and IM
mmu-miR-6401	1	1	o change between M and IM
mmu-mir-7067	0.696956	1.07589	M up vs IM
mmu-mir-6398	0.837907	1.03762	M up vs IM
mmu-mir-497b	1	1	o change between M and IM
mmu-miR-7653-3p	0.569931	-1.1199	M down vs IM
mmu-miR-126b-5p	1	1	o change between M and IM
mmu-mir-3473f	0.617264	-1.10178	M down vs IM
mmu-miR-130b-5p	0.34597	-1.26355	M down vs IM
mmu-mir-1906-1	0.689991	-1.1199	M down vs IM
mmu-mir-1906-2	0.689991	-1.1199	M down vs IM
mmu-miR-3076-5p	0.645131	-1.09209	M down vs IM
mmu-miR-18b-3p	0.77173	1.05439	M up vs IM
mmu-mir-742	0.777985	1.05273	M up vs IM
mmu-miR-669d-2-3p	0.790494	1.04945	M up vs IM
mmu-miR-101a-5p	0.790494	1.04945	M up vs IM
mmu-mir-338	0.663043	-1.08618	M down vs IM
mmu-miR-466c-5p	1	1	o change between M and IM
mmu-miR-1195	1	1	o change between M and IM
mmu-mir-5126	0.834114	-1.08486	M down vs IM
mmu-mir-592	0.477118	-1.13959	M down vs IM
mmu-miR-350-5p	0.477118	-1.13959	M down vs IM
mmu-miR-3960	0.0879318	-1.15595	M down vs IM
mmu-miR-7055-5p	0.304331	1.3022	M up vs IM
mmu-miR-3095-5p	1	1	o change between M and IM
mmu-miR-146b-3p	0.471047	-1.59752	M down vs IM
mmu-miR-6907-5p	1	1	o change between M and IM

mmu-mir-300	1	1	o change between M and IM
mmu-miR-7656-3p	0.718463	1.0737	M up vs IM
mmu-miR-380-3p	0.441129	-1.28696	M down vs IM
mmu-miR-615-3p	0.899841	1.03105	M up vs IM
mmu-mir-7666	0.641469	-1.08618	M down vs IM
mmu-miR-7653-5p	1	1	o change between M and IM
mmu-miR-141-3p	0.254051	1.36324	M up vs IM
mmu-miR-377-5p	0.774755	1.04945	M up vs IM
mmu-miR-92a-2-5p	0.622358	-1.09209	M down vs IM
mmu-miR-6986-3p	1	1	o change between M and IM
mmu-miR-7680-3p	1	1	o change between M and IM
mmu-mir-7011	0.314082	1.27507	M up vs IM
mmu-miR-300-3p	1	1	o change between M and IM
mmu-miR-669h-3p	0.799493	1.04349	M up vs IM
mmu-miR-3112-5p	0.797274	1.04402	M up vs IM
mmu-mir-5113	1	1	o change between M and IM
mmu-miR-145a-3p	0.799493	1.04349	M up vs IM
mmu-miR-485-5p	0.603433	1.09841	M up vs IM
mmu-miR-92a-1-5p	1	1	o change between M and IM
mmu-miR-7063-5p	1	1	o change between M and IM
mmu-mir-376c	0.603433	1.09841	M up vs IM
mmu-mir-1952	0.644048	1.0852	M up vs IM
mmu-miR-6949-5p	0.644048	1.0852	M up vs IM
mmu-miR-6340	0.522882	-1.1293	M down vs IM
mmu-mir-1956	0.754845	1.05439	M up vs IM
mmu-miR-465a-3p	0.774755	1.04945	M up vs IM
mmu-miR-465b-3p	0.774755	1.04945	M up vs IM
mmu-miR-465c-3p	0.774755	1.04945	M up vs IM
mmu-miR-205-5p	0.774755	1.04945	M up vs IM
mmu-miR-6942-3p	0.774755	1.04945	M up vs IM
mmu-mir-6928	0.799493	1.04349	M up vs IM
mmu-mir-1946b	1	1	o change between M and IM
mmu-miR-7661-5p	1	1	o change between M and IM
mmu-miR-1258-3p	1	1	o change between M and IM
mmu-mir-7035	0.522882	-1.1293	M down vs IM
mmu-miR-7672-3p	0.622358	-1.09209	M down vs IM
mmu-mir-30e	1	1	o change between M and IM
mmu-mir-30e	1	1	o change between M and IM
mmu-mir-291b	1	1	o change between M and IM
mmu-mir-7678	1	1	o change between M and IM
mmu-miR-29c-3p	1	1	o change between M and IM
mmu-miR-7011-3p	1	1	o change between M and IM
mmu-miR-7067-5p	1	1	o change between M and IM

mmu-mir-491	0.65252	-1.09752	M down vs IM
mmu-miR-28a-5p	0.447467	-1.49689	M down vs IM
mmu-miR-3112-3p	1	1	o change between M and IM
mmu-miR-6899-3p	1	1	o change between M and IM
mmu-miR-873b	1	1	o change between M and IM
mmu-mir-7016	0.76141	1.08204	M up vs IM
mmu-miR-6989-5p	0.751414	1.04772	M up vs IM
mmu-miR-3620-3p	0.444107	-1.04504	M down vs IM
mmu-mir-24-2	0.617249	-1.09752	M down vs IM
mmu-mir-873b	0.261592	1.09334	M up vs IM
mmu-miR-3070a-3p	0.490471	-1.27623	M down vs IM
mmu-mir-6541	0.720779	-1.09209	M down vs IM
mmu-mir-760	0.771339	1.05439	M up vs IM
mmu-miR-6900-3p	0.645648	-1.09209	M down vs IM
mmu-miR-491-5p	0.224047	1.38496	M up vs IM
mmu-miR-488-3p	0.207671	-1.42407	M down vs IM
mmu-miR-704	0.311553	-1.25175	M down vs IM
mmu-mir-22	0.304625	1.2593	M up vs IM
mmu-mir-134	0.32643	1.23668	M up vs IM
mmu-mir-7a-1	0.517356	-1.1199	M down vs IM
mmu-mir-7646	0.353545	1.21257	M up vs IM
mmu-mir-6537	0.517356	-1.1199	M down vs IM
mmu-miR-7035-3p	0.85069	1.02899	M up vs IM
mmu-miR-489-5p	0.614959	-1.08618	M down vs IM
mmu-miR-6936-5p	0.614959	-1.08618	M down vs IM
mmu-miR-219b-3p	1	1	o change between M and IM
mmu-miR-3066-3p	1	1	o change between M and IM
mmu-miR-7039-5p	0.618231	1.0852	M up vs IM
mmu-mir-1843b	0.809348	1.03762	M up vs IM
mmu-mir-1958	0.517356	-1.1199	M down vs IM
mmu-mir-196a-2	0.734597	1.05439	M up vs IM
mmu-mir-6909	0.734597	1.05439	M up vs IM
mmu-miR-7053-3p	0.734597	1.05439	M up vs IM
mmu-miR-679-3p	0.614959	-1.08618	M down vs IM
mmu-mir-7056	0.755814	1.04945	M up vs IM
mmu-miR-669i	0.755814	1.04945	M up vs IM
mmu-let-7a-2-3p	0.755814	1.04945	M up vs IM
mmu-miR-5099	0.755814	1.04945	M up vs IM
mmu-mir-6359	0.657624	-1.07394	M down vs IM
mmu-miR-6902-5p	0.541449	-1.11066	M down vs IM
mmu-mir-7117	1	1	o change between M and IM
mmu-miR-6898-3p	1	1	o change between M and IM
mmu-mir-30a	1	1	o change between M and IM

mmu-miR-6910-3p	0.614959	-1.08618	M down vs IM
mmu-mir-128-2	1	1	o change between M and IM
mmu-mir-6346	1	1	o change between M and IM
mmu-miR-3108-5p	1	1	o change between M and IM
mmu-miR-7086-5p	1	1	o change between M and IM
mmu-mir-7017	1	1	o change between M and IM
mmu-miR-7042-5p	1	1	o change between M and IM
mmu-miR-6933-5p	0.392404	-1.29062	M down vs IM
mmu-miR-676-3p	0.852741	1.04349	M up vs IM
mmu-miR-199a-5p	0.355344	-1.30071	M down vs IM
mmu-mir-374c	0.36464	-1.2899	M down vs IM
mmu-miR-453	0.404294	1.06229	M up vs IM
mmu-miR-187-3p	0.635095	-1.10178	M down vs IM
mmu-miR-8106	0.388197	-1.30071	M down vs IM
mmu-miR-294-5p	0.718994	-1.09209	M down vs IM
mmu-miR-3079-3p	1	1	o change between M and IM
mmu-miR-1954	1	1	o change between M and IM
mmu-mir-3961	0.338356	1.21257	M up vs IM
mmu-mir-6966	0.458034	-1.13807	M down vs IM
mmu-miR-455-5p	0.721659	1.05439	M up vs IM
mmu-miR-6367	0.743671	1.04945	M up vs IM
mmu-mir-139	1	1	o change between M and IM
mmu-miR-7649-5p	0.680927	-1.09752	M down vs IM
mmu-mir-703	0.435178	-1.2603	M down vs IM
mmu-mir-344d-3	0.840394	1.04945	M up vs IM
mmu-mir-200c	0.566693	-1.1293	M down vs IM
mmu-miR-467c-5p	0.431775	-1.24659	M down vs IM
mmu-mir-7237	0.892472	-1.03994	M down vs IM
mmu-mir-449a	0.646823	-1.09752	M down vs IM
mmu-mir-24-2	0.646823	-1.09752	M down vs IM
mmu-miR-302d-3p	0.646823	-1.09752	M down vs IM
mmu-miR-7670-5p	0.634066	-1.1199	M down vs IM
mmu-miR-211-5p	0.879469	1.03407	M up vs IM
mmu-miR-6347	0.55625	-1.54599	M down vs IM
mmu-miR-1291	0.742239	1.0683	M up vs IM
mmu-miR-532-3p	0.433732	-1.26869	M down vs IM
mmu-mir-155	1	1	o change between M and IM
mmu-mir-6970	0.596182	-1.13959	M down vs IM
mmu-mir-1190	0.397742	-1.27925	M down vs IM
mmu-mir-6925	0.433998	-1.24464	M down vs IM
mmu-miR-29c-5p	0.871727	1.03694	M up vs IM
mmu-miR-7018-3p	0.692735	-1.09752	M down vs IM
mmu-miR-804	0.706853	-1.09209	M down vs IM

mmu-miR-346-5p	1	1	o change between M and IM
mmu-miR-696	0.754683	1.04349	M up vs IM
mmu-miR-7064-3p	1	1	o change between M and IM
mmu-miR-3474	1	1	o change between M and IM
mmu-mir-145a	1	1	o change between M and IM
mmu-miR-3107-3p	1	1	o change between M and IM
mmu-miR-429-5p	0.5	-1.10407	M down vs IM
mmu-miR-6537-3p	0.30951	-1.20543	M down vs IM
mmu-miR-1198-5p	0.777561	-1.12853	M down vs IM
mmu-miR-653-3p	1	1	o change between M and IM
mmu-miR-6983-3p	0.668185	-1.09529	M down vs IM
mmu-miR-6968-3p	0.256126	-1.47077	M down vs IM
mmu-miR-452-3p	0.705828	1.0852	M up vs IM
mmu-miR-6993-5p	0.36728	-1.28693	M down vs IM
mmu-miR-6934-5p	0.377144	-1.27623	M down vs IM
mmu-mir-374c	0.417548	-1.2378	M down vs IM
mmu-mir-297a-4	0.572027	-1.13959	M down vs IM
mmu-mir-181c	0.686456	-1.09209	M down vs IM
mmu-miR-541-3p	0.241214	-1.27623	M down vs IM
mmu-mir-3076	1	1	o change between M and IM
mmu-miR-7013-5p	?	1	o change between M and IM
mmu-miR-7b-5p	0.91371	1.04945	M up vs IM
mmu-miR-6992-3p	0	-1.0158	M down vs IM
mmu-miR-7076-5p	1	1	o change between M and IM
mmu-mir-496a	0.372178	-1.28693	M down vs IM
mmu-mir-488	0.701584	1.08821	M up vs IM
mmu-mir-489	0.841047	1.04349	M up vs IM
mmu-mir-205	1	1	o change between M and IM
mmu-miR-669j	1	1	o change between M and IM
mmu-miR-8091	0.590377	-1.15179	M down vs IM
mmu-mir-6368	1	1	o change between M and IM
mmu-mir-493	0.718192	1.07589	M up vs IM
mmu-mir-1907	0.234517	1.53005	M up vs IM
mmu-miR-7660-3p	0.823573	1.06229	M up vs IM
mmu-miR-463-3p	1	1	o change between M and IM
mmu-mir-467h	0.845195	-1.08618	M down vs IM
mmu-miR-7021-5p	1	1	o change between M and IM
mmu-mir-299b	1	1	o change between M and IM
mmu-miR-7026-5p	0.807957	1.04349	M up vs IM
mmu-miR-6418-3p	0.435565	1.18264	M up vs IM
mmu-mir-365-2	0.89171	1.02378	M up vs IM
mmu-mir-490	0.872124	1.03105	M up vs IM
mmu-miR-128-1-5p	0.37526	-1.25175	M down vs IM

mmu-mir-302c	0.352387	1.27507	M up vs IM
mmu-miR-688	0.711437	1.07589	M up vs IM
mmu-miR-7061-3p	0.676812	1.08713	M up vs IM
mmu-miR-412-3p	0.662251	-1.09209	M down vs IM
mmu-mir-1843b	0.596769	1.11658	M up vs IM
mmu-miR-3082-3p	0.872124	1.03105	M up vs IM
mmu-miR-218-5p	0.698749	-1.07977	M down vs IM
mmu-miR-3102-3p.2-3p	1	1	o change between M and IM
mmu-miR-3071-3p	0.519706	-1.09752	M down vs IM
mmu-mir-669o	0.59455	-1.1199	M down vs IM
mmu-mir-19b-1	1	1	o change between M and IM
mmu-mir-669p-1	0.684798	-1.08618	M down vs IM
mmu-mir-669p-2	0.684798	-1.08618	M down vs IM
mmu-mir-6420	1	1	o change between M and IM
mmu-miR-7667-3p	0.722656	-1.08618	M down vs IM
mmu-miR-1264-3p	0.208509	-1.4303	M down vs IM
mmu-miR-494-5p	0.400659	-1.27623	M down vs IM
mmu-miR-7674-5p	0.701497	1.07904	M up vs IM
mmu-mir-873a	0.334987	-1.29505	M down vs IM
mmu-mir-6983	0.701497	1.07904	M up vs IM
mmu-miR-6954-3p	1	1	o change between M and IM
mmu-miR-30d-3p	0.257685	1.39914	M up vs IM
mmu-miR-505-5p	1	1	o change between M and IM
mmu-mir-717	0.640231	-1.08486	M down vs IM
mmu-miR-7054-3p	0.693405	1.04349	M up vs IM
mmu-miR-466d-5p	0.659474	1.04945	M up vs IM
mmu-miR-7080-3p	0.693405	1.04349	M up vs IM
mmu-miR-2136	0.49529	-1.08618	M down vs IM
mmu-mir-207	1	1	o change between M and IM
mmu-miR-7043-3p	1	1	o change between M and IM
mmu-mir-7685	0.320548	1.33255	M up vs IM
mmu-miR-7051-5p	0.774363	1.07589	M up vs IM
mmu-miR-7066-3p	0.797242	-1.19103	M down vs IM
mmu-mir-7243	1	1	o change between M and IM
mmu-miR-496b	1	1	o change between M and IM
mmu-miR-344c-3p	0.3522	1.248	M up vs IM
mmu-miR-7004-3p	0.844108	1.03931	M up vs IM
mmu-miR-5134-3p	0.599189	-1.12704	M down vs IM
mmu-miR-7240-5p	0.718905	-1.09752	M down vs IM
mmu-mir-183	?	1	o change between M and IM
mmu-mir-7689	?	1	o change between M and IM
mmu-miR-370-5p	?	1	o change between M and IM
mmu-miR-6900-5p	0	1.09334	M up vs IM

mmu-miR-6958-3p	0	1.38496	M up vs IM
mmu-mir-1981	0.374657	-1.25175	M down vs IM
mmu-mir-1905	0.112191	1.95733	M up vs IM
mmu-mir-6938	0.567302	1.10147	M up vs IM
mmu-miR-3104-3p	0.639247	-1.09209	M down vs IM
mmu-miR-210-5p	0.840036	1.03694	M up vs IM
mmu-mir-7001	0.715378	-1.08618	M down vs IM
mmu-mir-6997	0.292532	1.3022	M up vs IM
mmu-miR-709	0.160458	-3.03701	M down vs IM
mmu-miR-3091-3p	0.622708	-1.12704	M down vs IM
mmu-mir-7242	0.235098	-1.38678	M down vs IM
mmu-mir-344d-2	0.6455	1.10978	M up vs IM
mmu-mir-344d-2	0.6455	1.10978	M up vs IM
mmu-miR-3569-3p	0.579836	-1.08486	M down vs IM
mmu-miR-7094-1-5p	0.602938	-1.09752	M down vs IM
mmu-miR-7119-5p	0.602938	-1.09752	M down vs IM
mmu-miR-466k	0.873794	-1.08618	M down vs IM
mmu-miR-7236-3p	0.345879	-1.22304	M down vs IM
mmu-mir-6994	0.600854	-1.09209	M down vs IM
mmu-miR-3062-3p	0.600854	-1.09209	M down vs IM
mmu-miR-698-3p	0.744659	1.0852	M up vs IM
mmu-mir-547	1	1	o change between M and IM
mmu-mir-106b	0.568973	1.04349	M up vs IM
mmu-mir-505	1	1	o change between M and IM
mmu-let-7f-2	0.5	-1.05439	M down vs IM
mmu-miR-6403	0.826129	1.03407	M up vs IM
mmu-miR-450b-5p	0.312328	-1.23615	M down vs IM
mmu-miR-26b-3p	0.598564	-1.08618	M down vs IM
mmu-mir-181b-2	0.394384	-1.20924	M down vs IM
mmu-miR-1b-5p	0.93416	-1.05027	M down vs IM
mmu-miR-1892	0.0906968	1.30842	M up vs IM
mmu-miR-449a-5p	0.606261	-1.08618	M down vs IM
mmu-mir-297a-2	1	1	o change between M and IM
mmu-mir-344b	0.5	1.04945	M up vs IM
mmu-mir-6397	1	1	o change between M and IM
mmu-miR-6411	0.33947	1.0852	M up vs IM
mmu-mir-219c	0.33947	1.0852	M up vs IM
mmu-mir-6986	0.470463	1.05439	M up vs IM
mmu-mir-455	1	1	o change between M and IM
mmu-miR-7022-3p	1	1	o change between M and IM
mmu-miR-206-3p	1	1	o change between M and IM
mmu-miR-294-3p	1	1	o change between M and IM
mmu-mir-410	1	1	o change between M and IM

mmu-mir-3547	1	1	o change between M and IM
mmu-mir-6980	1	1	o change between M and IM
mmu-miR-125a-3p	1	1	o change between M and IM
mmu-let-7j	1	1	o change between M and IM
mmu-miR-7050-3p	1	1	o change between M and IM
mmu-mir-3080	0.61933	-1.09209	M down vs IM
mmu-miR-6955-3p	0.613336	-1.08618	M down vs IM
mmu-mir-144	1	1	o change between M and IM
mmu-miR-6343	0.888163	1.03407	M up vs IM
mmu-miR-6998-3p	0.279691	1.25022	M up vs IM
mmu-miR-199a-3p	0.619886	-1.08618	M down vs IM
mmu-miR-199b-3p	0.619886	-1.08618	M down vs IM
mmu-miR-466p-5p	0.66414	1.11254	M up vs IM
mmu-mir-710	0.627701	1.11481	M up vs IM
mmu-miR-3102-3p	0.834611	-1.03108	M down vs IM
mmu-mir-1a-2	0.099249	-1.31108	M down vs IM
mmu-miR-6353	0.5	1.04349	M up vs IM
mmu-mir-3473d	1	1	o change between M and IM
mmu-mir-3104	0.169791	1.16861	M up vs IM
mmu-miR-3085-5p	1	1	o change between M and IM
mmu-let-7f-1	1	1	o change between M and IM
mmu-miR-6973a-3p	1	1	o change between M and IM
mmu-mir-6546	0.5	1.04349	M up vs IM
mmu-mir-7092	1	1	o change between M and IM
mmu-mir-6417	1	1	o change between M and IM
mmu-miR-5619-3p	1	1	o change between M and IM
mmu-miR-322-3p	1	1	o change between M and IM
mmu-miR-499-3p	0.807303	1.09334	M up vs IM
mmu-miR-320-5p	1	1	o change between M and IM
mmu-mir-100	1	1	o change between M and IM
mmu-mir-1941	1	1	o change between M and IM
mmu-mir-28c	1	1	o change between M and IM
mmu-mir-6914	1	1	o change between M and IM
mmu-mir-6960	1	1	o change between M and IM
mmu-miR-26b-5p	1	1	o change between M and IM
mmu-miR-30b-3p	1	1	o change between M and IM
mmu-miR-1843b-5p	1	1	o change between M and IM
mmu-miR-5125	1	1	o change between M and IM
mmu-miR-6924-5p	1	1	o change between M and IM
mmu-miR-7211-3p	1	1	o change between M and IM
mmu-miR-216c-3p	1	1	o change between M and IM
mmu-mir-3071	1	1	o change between M and IM
mmu-miR-421-3p	1	1	o change between M and IM

mmu-miR-7066-5p	0.624255	-1.08486	M down vs IM
mmu-miR-3071-5p	0.167388	1.27507	M up vs IM
mmu-miR-7213-5p	0.756224	-1.09752	M down vs IM
mmu-miR-344g-3p	0.600722	1.07904	M up vs IM
mmu-mir-1936	0.94175	-1.00293	M down vs IM
mmu-miR-7051-3p	0.0556488	1.52402	M up vs IM
mmu-miR-1906	0.049571	1.60522	M up vs IM
mmu-miR-7228-3p	0.109567	1.23668	M up vs IM
mmu-miR-7084-3p	0.109567	1.23668	M up vs IM
mmu-mir-337	0.297613	1.07589	M up vs IM
mmu-mir-6919	0.148136	1.16861	M up vs IM
mmu-miR-6897-3p	0.148136	1.16861	M up vs IM
mmu-mir-598	1	1	o change between M and IM
mmu-miR-543-3p	0.270962	-1.08486	M down vs IM
mmu-miR-7077-5p	0.454867	1.04349	M up vs IM
mmu-miR-706	1	1	o change between M and IM
mmu-miR-504-5p	1	1	o change between M and IM
mmu-let-7e	0.162093	-1.15244	M down vs IM
mmu-miR-7215-3p	1	1	o change between M and IM
mmu-miR-7657-5p	0.249768	1.09334	M up vs IM
mmu-miR-1966-5p	0.249768	1.09334	M up vs IM
mmu-mir-767	0.252683	-1.09209	M down vs IM
mmu-miR-7578	0.215372	-1.11066	M down vs IM
mmu-miR-6481	0.270056	1.0852	M up vs IM
mmu-mir-669e	0.297613	1.07589	M up vs IM
mmu-mir-5136	0.252683	-1.09209	M down vs IM
mmu-mir-7063	0.252683	-1.09209	M down vs IM
mmu-miR-7059-5p	0.454867	1.04349	M up vs IM
mmu-let-7i-3p	0.387607	1.05439	M up vs IM
mmu-mir-125b-1	0.415776	1.04945	M up vs IM
mmu-mir-540	0.454867	1.04349	M up vs IM
mmu-miR-137-5p	0.454867	1.04349	M up vs IM
mmu-mir-3067	0.454867	1.04349	M up vs IM
mmu-miR-3970	0.454867	1.04349	M up vs IM
mmu-mir-683-1	0.5	1.03762	M up vs IM
mmu-mir-683-2	0.5	1.03762	M up vs IM
mmu-miR-669b-5p	1	1	o change between M and IM
mmu-mir-130a	1	1	o change between M and IM
mmu-mir-19b-2	1	1	o change between M and IM
mmu-mir-19b-2	1	1	o change between M and IM
mmu-mir-148a	1	1	o change between M and IM
mmu-mir-3475	1	1	o change between M and IM
mmu-mir-1191	1	1	o change between M and IM

mmu-mir-1892	1	1	o change between M and IM
mmu-mir-5619	1	1	o change between M and IM
mmu-mir-6362	1	1	o change between M and IM
mmu-mir-6957	1	1	o change between M and IM
mmu-mir-7211	1	1	o change between M and IM
mmu-miR-183-5p	1	1	o change between M and IM
mmu-miR-219a-5p	1	1	o change between M and IM
mmu-miR-365-3p	1	1	o change between M and IM
mmu-miR-375-3p	1	1	o change between M and IM
mmu-miR-7a-1-3p	1	1	o change between M and IM
mmu-miR-344d-2-5p	1	1	o change between M and IM
mmu-miR-16-2-3p	1	1	o change between M and IM
mmu-miR-184-5p	1	1	o change between M and IM
mmu-miR-6381	1	1	o change between M and IM
mmu-miR-7015-3p	1	1	o change between M and IM
mmu-miR-142b	1	1	o change between M and IM
mmu-mir-343	1	1	o change between M and IM
mmu-mir-302a	0.398874	1.2414	M up vs IM
mmu-mir-92a-2	0.751936	-1.09209	M down vs IM
mmu-miR-467d-5p	0.566273	1.12191	M up vs IM
mmu-miR-883b-5p	0.78814	1.03694	M up vs IM
mmu-mir-378c	0.371475	1.04945	M up vs IM
mmu-mir-7022	1	1	o change between M and IM
mmu-miR-6975-3p	1	1	o change between M and IM
mmu-mir-302d	1	1	o change between M and IM
mmu-let-7f-1-3p	0.208338	1.09841	M up vs IM
mmu-miR-5618-3p	1	1	o change between M and IM
mmu-mir-219c	0.236565	1.0852	M up vs IM
mmu-miR-6949-3p	0.26153	1.07589	M up vs IM
mmu-mir-219b	0.409037	1.04349	M up vs IM
mmu-miR-467c-3p	1	1	o change between M and IM
mmu-miR-5134-5p	1	1	o change between M and IM
mmu-mir-3094	1	1	o change between M and IM
mmu-miR-7008-3p	1	1	o change between M and IM
mmu-mir-1197	1	1	o change between M and IM
mmu-mir-5121	1	1	o change between M and IM
mmu-mir-129b	1	1	o change between M and IM
mmu-miR-3113-3p	1	1	o change between M and IM
mmu-miR-6386	1	1	o change between M and IM
mmu-miR-7040-3p	1	1	o change between M and IM
mmu-miR-7235-3p	1	1	o change between M and IM
mmu-miR-7084-5p	0.617278	1.10849	M up vs IM
mmu-miR-467g	1	1	o change between M and IM

mmu-miR-3105-3p	1	1	o change between M and IM
mmu-let-7c-5p	0.528937	1.10898	M up vs IM
mmu-miR-7027-3p	0.0903927	1.21257	M up vs IM
mmu-mir-7241	0.137469	1.13383	M up vs IM
mmu-miR-8098	0.11142	1.16861	M up vs IM
mmu-miR-7055-3p	0.893263	1.07935	M up vs IM
mmu-miR-6923-5p	1	1	o change between M and IM
mmu-miR-216b-5p	1	1	o change between M and IM
mmu-mir-8106	0.682729	-1.15179	M down vs IM
mmu-miR-203-5p	0.72192	1.173	M up vs IM
mmu-miR-128-3p	0.478357	-1.19211	M down vs IM
mmu-mir-1897	0.499185	-1.09209	M down vs IM
mmu-miR-7678-3p	0.579891	-1.27623	M down vs IM
mmu-miR-6927-5p	0.45519	1.09841	M up vs IM
mmu-miR-3078-3p	0.154837	1.38856	M up vs IM
mmu-mir-5125	1	1	o change between M and IM
mmu-miR-466a-3p	1	1	o change between M and IM
mmu-miR-466e-3p	1	1	o change between M and IM
mmu-mir-7013	1	1	o change between M and IM
mmu-mir-7021	0.72328	-1.1199	M down vs IM
mmu-mir-26a-2	0	1.07589	M up vs IM
mmu-miR-215-5p	1	1	o change between M and IM
mmu-miR-7069-3p	0.579635	-1.30071	M down vs IM
mmu-mir-6967	1	1	o change between M and IM
mmu-mir-219a-2	0.761986	-1.09209	M down vs IM
mmu-miR-6944-3p	0.203614	1.27881	M up vs IM
mmu-miR-7663-3p	0.65079	1.03762	M up vs IM
mmu-miR-224-5p	0.555622	-1.13426	M down vs IM
mmu-mir-153	0.435476	-1.22304	M down vs IM
mmu-miR-344d-3-5p	1	1	o change between M and IM
mmu-mir-6539	0.935451	-1.04501	M down vs IM
mmu-miR-301a-5p	0.590878	1.11899	M up vs IM
mmu-miR-718	1	1	o change between M and IM
mmu-miR-6932-3p	0.477055	1.0852	M up vs IM
mmu-miR-6416-3p	0.924933	-1.04834	M down vs IM
mmu-mir-1943	0.4904	-1.18435	M down vs IM
mmu-miR-299b-3p	0.86179	1.03407	M up vs IM
mmu-miR-129b-3p	1	1	o change between M and IM
mmu-mir-5131	1	1	o change between M and IM
mmu-miR-215-3p	1	1	o change between M and IM
mmu-miR-6360	1	1	o change between M and IM
mmu-miR-3966	1	1	o change between M and IM
mmu-miR-202-3p	1	1	o change between M and IM

mmu-miR-471-3p	1	1	o change between M and IM
mmu-miR-6924-3p	0.975866	-1.00278	M down vs IM
mmu-mir-127	0.537807	-1.27925	M down vs IM
mmu-mir-7655	0.700049	-1.15179	M down vs IM
mmu-mir-1194	1	1	o change between M and IM
mmu-mir-6390	1	1	o change between M and IM
mmu-miR-744-3p	1	1	o change between M and IM
mmu-mir-9-2	0	1.09334	M up vs IM
mmu-mir-133a-1	0	1.09881	M up vs IM
mmu-mir-140	0	1.01992	M up vs IM
mmu-mir-187	0	-1.1293	M down vs IM
mmu-mir-203	?	1	o change between M and IM
mmu-mir-298	0	-1.10178	M down vs IM
mmu-mir-34c	0	-1.26355	M down vs IM
mmu-mir-130b	0	-1.26355	M down vs IM
mmu-mir-196a-1	0	-1.08618	M down vs IM
mmu-let-7b	0	1.04945	M up vs IM
mmu-mir-96	0	1.23668	M up vs IM
mmu-mir-339	0	1.04349	M up vs IM
mmu-mir-345	0	1.17463	M up vs IM
mmu-mir-138-1	0	-1.09209	M down vs IM
mmu-mir-362	0	1.03238	M up vs IM
mmu-mir-377	?	1	o change between M and IM
mmu-mir-378a	0	-1.21144	M down vs IM
mmu-mir-335	0	1.05439	M up vs IM
mmu-mir-133a-2	?	1	o change between M and IM
mmu-mir-215	0	-1.1293	M down vs IM
mmu-mir-466a	?	1	o change between M and IM
mmu-mir-468	0	1.05439	M up vs IM
mmu-mir-483	?	1	o change between M and IM
mmu-mir-367	0	1.05686	M up vs IM
mmu-mir-503	0	1.07589	M up vs IM
mmu-mir-1249	?	1	o change between M and IM
mmu-mir-770	0	-1.27623	M down vs IM
mmu-mir-672	0	-1.08618	M down vs IM
mmu-mir-3059	0	1.04945	M up vs IM
mmu-mir-674	?	1	o change between M and IM
mmu-mir-680-1	0	-1.1293	M down vs IM
mmu-mir-688	0	1.06229	M up vs IM
mmu-mir-697	0	-1.1293	M down vs IM
mmu-mir-704	0	-1.1293	M down vs IM
mmu-mir-707	0	1.0852	M up vs IM
mmu-mir-713	0	1.2593	M up vs IM

mmu-mir-500		0	-1.13807	M down vs IM
mmu-mir-615		0	-1.26355	M down vs IM
mmu-mir-181d		0	-1.10407	M down vs IM
mmu-mir-181d		0	-1.1293	M down vs IM
mmu-mir-871	?		1	o change between M and IM
mmu-mir-190b		0	-1.08618	M down vs IM
mmu-mir-297c		0	1.04349	M up vs IM
mmu-mir-466e	?		1	o change between M and IM
mmu-mir-466f-2		0	1.09334	M up vs IM
mmu-mir-466g		0	-1.1293	M down vs IM
mmu-mir-875		0	1.21257	M up vs IM
mmu-mir-669d	?		1	o change between M and IM
mmu-mir-669d	?		1	o change between M and IM
mmu-mir-669i	?		1	o change between M and IM
mmu-mir-1893	?		1	o change between M and IM
mmu-mir-1306		0	-1.08618	M down vs IM
mmu-mir-1955		0	-1.31971	M down vs IM
mmu-mir-1962		0	-1.26355	M down vs IM
mmu-mir-1964	?		1	o change between M and IM
mmu-mir-1982		0	1.32317	M up vs IM
mmu-mir-3074-1		0	1.27603	M up vs IM
mmu-mir-3074-1		0	1.0852	M up vs IM
mmu-mir-3085		0	-1.1293	M down vs IM
mmu-mir-669d-2		0	-1.1293	M down vs IM
mmu-mir-466c-2	?		1	o change between M and IM
mmu-mir-3092		0	-1.12462	M down vs IM
mmu-mir-3095		0	1.04945	M up vs IM
mmu-mir-3100	?		1	o change between M and IM
mmu-mir-344f		0	1.04945	M up vs IM
mmu-mir-3967	?		1	o change between M and IM
mmu-mir-5046	?		1	o change between M and IM
mmu-mir-5120		0	1.04945	M up vs IM
mmu-mir-5122		0	-1.08589	M down vs IM
mmu-mir-5123	?		1	o change between M and IM
mmu-mir-5617		0	-1.1293	M down vs IM
mmu-mir-6237		0	1.04349	M up vs IM
mmu-mir-6348		0	-1.31971	M down vs IM
mmu-mir-6349		0	-1.29062	M down vs IM
mmu-mir-6350		0	-1.1293	M down vs IM
mmu-mir-21b		0	-1.1451	M down vs IM
mmu-mir-6381	?		1	o change between M and IM
mmu-mir-6395		0	1.04349	M up vs IM
mmu-mir-6396		0	-1.29062	M down vs IM

mmu-mir-6403	0	-1.1293	M down vs IM
mmu-mir-6411	0	1.04349	M up vs IM
mmu-mir-6412	0	-1.1199	M down vs IM
mmu-mir-6415	0	-1.1293	M down vs IM
mmu-mir-6419	0	-1.08486	M down vs IM
mmu-mir-6540	0	-1.08618	M down vs IM
mmu-mir-6901	0	1.04945	M up vs IM
mmu-mir-6902	?	1	o change between M and IM
mmu-mir-6904	0	1.09334	M up vs IM
mmu-mir-6905	?	1	o change between M and IM
mmu-mir-6922	0	1.05439	M up vs IM
mmu-mir-6927	0	1.04945	M up vs IM
mmu-mir-6934	?	1	o change between M and IM
mmu-mir-6936	0	1.05439	M up vs IM
mmu-mir-6944	?	1	o change between M and IM
mmu-mir-6947	0	1.05686	M up vs IM
mmu-mir-6949	0	-1.16877	M down vs IM
mmu-mir-6959	0	1.03762	M up vs IM
mmu-mir-6969	0	1.04349	M up vs IM
mmu-mir-6976	?	1	o change between M and IM
mmu-mir-6989	?	1	o change between M and IM
mmu-mir-6990	0	1.23668	M up vs IM
mmu-mir-7008	0	1.04349	M up vs IM
mmu-mir-7019	0	1.18971	M up vs IM
mmu-mir-7040	0	1.04349	M up vs IM
mmu-mir-7041	?	1	o change between M and IM
mmu-mir-7042	?	1	o change between M and IM
mmu-mir-7047	0	-1.11066	M down vs IM
mmu-mir-7059	0	-1.1199	M down vs IM
mmu-mir-7061	0	1.09334	M up vs IM
mmu-mir-7080	0	-1.11066	M down vs IM
mmu-mir-7081	0	1.21257	M up vs IM
mmu-mir-7091	0	1.27507	M up vs IM
mmu-mir-7223	0	-1.09209	M down vs IM
mmu-mir-7229	?	1	o change between M and IM
mmu-mir-7232	0	1.07589	M up vs IM
mmu-mir-7235	?	1	o change between M and IM
mmu-mir-7238	0	1.09334	M up vs IM
mmu-mir-7578	0	-1.27623	M down vs IM
mmu-mir-7658	0	1.05686	M up vs IM
mmu-mir-7675	0	-1.09752	M down vs IM
mmu-mir-7676-1	0	-1.27623	M down vs IM
mmu-mir-7676-2	0	-1.27623	M down vs IM

mmu-mir-7680	0	1.04945	M up vs IM
mmu-mir-7687	0	1.27507	M up vs IM
mmu-mir-1258	0	1.05439	M up vs IM
mmu-mir-8092	?	1	o change between M and IM
mmu-mir-8104	?	1	o change between M and IM
mmu-mir-8116	0	-1.1199	M down vs IM
mmu-miR-29b-3p	0	-1.1293	M down vs IM
mmu-miR-127-3p	?	1	o change between M and IM
mmu-miR-9-3p	0	1.04945	M up vs IM
mmu-miR-186-5p	0	-1.1199	M down vs IM
mmu-miR-195a-5p	0	1.43706	M up vs IM
mmu-miR-30e-5p	0	-1.1293	M down vs IM
mmu-miR-297a-5p	0	-1.09209	M down vs IM
mmu-miR-19b-3p	?	1	o change between M and IM
mmu-miR-326-3p	0	-1.08486	M down vs IM
mmu-miR-344-3p	0	1.0852	M up vs IM
mmu-miR-223-3p	0	-1.08618	M down vs IM
mmu-miR-33-5p	0	1.04349	M up vs IM
mmu-miR-133b-3p	0	-1.08618	M down vs IM
mmu-miR-369-3p	0	1.04349	M up vs IM
mmu-miR-302c-5p	0	-1.13807	M down vs IM
mmu-miR-497-5p	0	1.23668	M up vs IM
mmu-miR-133a-5p	0	-1.10407	M down vs IM
mmu-miR-669c-5p	0	-1.13807	M down vs IM
mmu-miR-297b-5p	0	-1.09752	M down vs IM
mmu-miR-700-3p	0	1.27603	M up vs IM
mmu-miR-707	0	-1.1293	M down vs IM
mmu-miR-501-5p	0	-1.34877	M down vs IM
mmu-miR-676-5p	0	-1.1199	M down vs IM
mmu-miR-761	0	1.04349	M up vs IM
mmu-miR-744-5p	0	2.29382	M up vs IM
mmu-miR-125b-2-3p	0	-1.1199	M down vs IM
mmu-miR-127-5p	0	-1.08618	M down vs IM
mmu-miR-188-3p	0	-1.1293	M down vs IM
mmu-miR-302a-5p	0	1.0852	M up vs IM
mmu-let-7a-1-3p	0	1.0852	M up vs IM
mmu-miR-93-3p	0	1.23668	M up vs IM
mmu-miR-323-5p	?	1	o change between M and IM
mmu-miR-325-3p	0	1.04945	M up vs IM
mmu-miR-10a-3p	?	1	o change between M and IM
mmu-miR-138-1-3p	0	-1.08618	M down vs IM
mmu-miR-20b-3p	0	-1.1293	M down vs IM
mmu-miR-297b-3p	0	-1.25175	M down vs IM

mmu-miR-742-5p	0	-1.1199	M down vs IM
mmu-miR-297a-3p	0	-1.25175	M down vs IM
mmu-miR-297c-3p	0	-1.25175	M down vs IM
mmu-miR-466b-3p	0	1.23668	M up vs IM
mmu-miR-466c-3p	0	1.23668	M up vs IM
mmu-miR-466e-5p	0	1.03762	M up vs IM
mmu-miR-582-3p	0	-1.09752	M down vs IM
mmu-miR-376c-5p	0	1.21257	M up vs IM
mmu-let-7c-2-3p	0	1.0852	M up vs IM
mmu-miR-669d-5p	0	1.04349	M up vs IM
mmu-miR-466j	0	1.04349	M up vs IM
mmu-miR-1197-3p	?	1	o change between M and IM
mmu-miR-1900	0	1.04349	M up vs IM
mmu-miR-1907	0	1.04349	M up vs IM
mmu-miR-1894-5p	0	1.124	M up vs IM
mmu-miR-1927	0	1.03762	M up vs IM
mmu-miR-1933-5p	0	1.04349	M up vs IM
mmu-miR-1933-3p	0	1.3022	M up vs IM
mmu-miR-1945	0	1.21257	M up vs IM
mmu-miR-1306-3p	0	-1.1199	M down vs IM
mmu-miR-669m-3p	?	1	o change between M and IM
mmu-miR-669o-5p	?	1	o change between M and IM
mmu-miR-1951	?	1	o change between M and IM
mmu-miR-669n	0	-1.08486	M down vs IM
mmu-miR-1969	0	1.27603	M up vs IM
mmu-miR-1247-5p	0	1.23668	M up vs IM
mmu-miR-1298-3p	?	1	o change between M and IM
mmu-miR-3061-3p	0	1.04349	M up vs IM
mmu-miR-3067-3p	0	-1.29062	M down vs IM
mmu-miR-3069-3p	0	1.0852	M up vs IM
mmu-miR-3072-5p	0	-1.27623	M down vs IM
mmu-miR-466o-3p	0	1.05686	M up vs IM
mmu-miR-466p-3p	0	1.23668	M up vs IM
mmu-miR-3093-3p	0	1.2593	M up vs IM
mmu-miR-3094-5p	?	1	o change between M and IM
mmu-miR-3101-5p	0	1.27507	M up vs IM
mmu-miR-344b-5p	0	-1.08618	M down vs IM
mmu-miR-3104-5p	0	1.6287	M up vs IM
mmu-miR-3470b	0	1.04349	M up vs IM
mmu-miR-132-5p	?	1	o change between M and IM
mmu-miR-153-5p	0	1.04349	M up vs IM
mmu-miR-135b-3p	0	-1.1199	M down vs IM
mmu-miR-135a-2-3p	0	-1.1199	M down vs IM

mmu-miR-128-2-5p	0	1.48397	M up vs IM
mmu-miR-361-3p	0	-1.29062	M down vs IM
mmu-miR-363-5p	0	1.27603	M up vs IM
mmu-miR-547-5p	0	-1.08618	M down vs IM
mmu-miR-668-5p	0	-1.45108	M down vs IM
mmu-miR-665-5p	0	1.04945	M up vs IM
mmu-miR-672-3p	0	-1.09209	M down vs IM
mmu-miR-670-3p	0	-1.09209	M down vs IM
mmu-miR-700-5p	0	1.07589	M up vs IM
mmu-miR-421-5p	0	1.04349	M up vs IM
mmu-miR-1199-3p	0	-1.15519	M down vs IM
mmu-miR-664-5p ?		1	o change between M and IM
mmu-miR-3964	0	-1.25175	M down vs IM
mmu-miR-3969	0	1.04349	M up vs IM
mmu-miR-5106	0	1.21257	M up vs IM
mmu-miR-5122	0	1.2593	M up vs IM
mmu-miR-5124a	0	-1.09209	M down vs IM
mmu-miR-5615-3p	0	-1.13766	M down vs IM
mmu-miR-1231-5p	0	-1.26355	M down vs IM
mmu-miR-5622-5p	0	-1.1293	M down vs IM
mmu-miR-5622-3p	0	-1.08486	M down vs IM
mmu-miR-5626-3p ?		1	o change between M and IM
mmu-miR-5710	0	1.23668	M up vs IM
mmu-miR-1929-3p	0	-1.27623	M down vs IM
mmu-miR-219a-2-3p	0	1.28214	M up vs IM
mmu-miR-5132-3p	0	-1.0772	M down vs IM
mmu-miR-6238	0	1.07589	M up vs IM
mmu-miR-6244	0	1.04349	M up vs IM
mmu-miR-6351	0	1.04945	M up vs IM
mmu-miR-6354 ?		1	o change between M and IM
mmu-miR-6356	0	1.03762	M up vs IM
mmu-miR-6370 ?		1	o change between M and IM
mmu-miR-6380	0	-1.11066	M down vs IM
mmu-miR-6392-5p	0	-1.11066	M down vs IM
mmu-miR-6407	0	1.04945	M up vs IM
mmu-miR-6409	0	-1.1293	M down vs IM
mmu-miR-6410	0	-1.08486	M down vs IM
mmu-miR-6419	0	1.33255	M up vs IM
mmu-miR-6540-5p ?		1	o change between M and IM
mmu-miR-6896-5p	0	1.28214	M up vs IM
mmu-miR-6901-5p	0	-1.1199	M down vs IM
mmu-miR-6904-3p	0	1.08713	M up vs IM
mmu-miR-6914-5p	0	-1.1293	M down vs IM

mmu-miR-6918-5p		0	1.04349	M up vs IM
mmu-miR-6919-5p	?		1	o change between M and IM
mmu-miR-6921-5p		0	-1.29062	M down vs IM
mmu-miR-6923-3p		0	-1.34877	M down vs IM
mmu-miR-6926-5p		0	-1.25175	M down vs IM
mmu-miR-6945-5p	?		1	o change between M and IM
mmu-miR-6953-3p	?		1	o change between M and IM
mmu-miR-6956-5p	?		1	o change between M and IM
mmu-miR-6958-5p		0	1.43706	M up vs IM
mmu-miR-6961-3p		0	-1.25175	M down vs IM
mmu-miR-6969-5p		0	1.04349	M up vs IM
mmu-miR-6978-3p		0	-1.09752	M down vs IM
mmu-miR-6988-3p		0	-1.08618	M down vs IM
mmu-miR-6995-5p		0	1.04349	M up vs IM
mmu-miR-7004-5p		0	1.04349	M up vs IM
mmu-miR-7009-3p		0	-1.1199	M down vs IM
mmu-miR-7011-5p		0	1.19113	M up vs IM
mmu-miR-7014-3p		0	-1.1199	M down vs IM
mmu-miR-7015-5p		0	1.04945	M up vs IM
mmu-miR-7018-5p	?		1	o change between M and IM
mmu-miR-7031-5p		0	-1.1199	M down vs IM
mmu-miR-7032-3p	?		1	o change between M and IM
mmu-miR-7033-5p	?		1	o change between M and IM
mmu-miR-7052-3p	?		1	o change between M and IM
mmu-miR-7062-5p	?		1	o change between M and IM
mmu-miR-7065-5p	?		1	o change between M and IM
mmu-miR-7067-3p	?		1	o change between M and IM
mmu-miR-7068-3p		0	1.04349	M up vs IM
mmu-miR-7079-3p		0	1.21257	M up vs IM
mmu-miR-7083-5p		0	-1.31971	M down vs IM
mmu-miR-7093-5p	?		1	o change between M and IM
mmu-miR-7214-5p	?		1	o change between M and IM
mmu-miR-7217-3p		0	-1.1293	M down vs IM
mmu-miR-7224-3p		0	1.43706	M up vs IM
mmu-miR-7226-5p		0	-1.09209	M down vs IM
mmu-miR-7228-5p		0	1.04349	M up vs IM
mmu-miR-7234-3p		0	-1.11066	M down vs IM
mmu-miR-7239-5p		0	1.03762	M up vs IM
mmu-miR-7647-3p		0	-1.1199	M down vs IM
mmu-miR-7660-5p		0	1.0852	M up vs IM
mmu-miR-7673-3p		0	1.0852	M up vs IM
mmu-miR-7680-5p		0	-1.31108	M down vs IM
mmu-miR-7681-5p	?		1	o change between M and IM

mmu-miR-7682-3p		0	1.2593	M up vs IM
mmu-miR-216c-5p		0	-1.09752	M down vs IM
mmu-miR-290b-5p		0	-1.38801	M down vs IM
mmu-miR-8102		0	1.09841	M up vs IM
mmu-miR-8107	?		1	o change between M and IM
mmu-miR-8116		0	1.06229	M up vs IM
mmu-miR-200c-3p		0.905769	1.05439	M up vs IM
mmu-let-7i	?		1	o change between M and IM
mmu-mir-99a	?		1	o change between M and IM
mmu-mir-126a	?		1	o change between M and IM
mmu-mir-128-1	?		1	o change between M and IM
mmu-mir-133a-1	?		1	o change between M and IM
mmu-mir-152	?		1	o change between M and IM
mmu-mir-191	?		1	o change between M and IM
mmu-mir-206	?		1	o change between M and IM
mmu-mir-290a	?		1	o change between M and IM
mmu-let-7d	?		1	o change between M and IM
mmu-let-7f-2	?		1	o change between M and IM
mmu-mir-26b	?		1	o change between M and IM
mmu-mir-103-2	?		1	o change between M and IM
mmu-mir-341	?		1	o change between M and IM
mmu-mir-346	?		1	o change between M and IM
mmu-mir-199b	?		1	o change between M and IM
mmu-mir-9-1	?		1	o change between M and IM
mmu-mir-9-1	?		1	o change between M and IM
mmu-mir-363	?		1	o change between M and IM
mmu-mir-376a	?		1	o change between M and IM
mmu-mir-379	?		1	o change between M and IM
mmu-mir-665	?		1	o change between M and IM
mmu-mir-1298	?		1	o change between M and IM
mmu-mir-686	?		1	o change between M and IM
mmu-mir-504	?		1	o change between M and IM
mmu-mir-669g	?		1	o change between M and IM
mmu-mir-669j	?		1	o change between M and IM
mmu-mir-1195	?		1	o change between M and IM
mmu-mir-1904	?		1	o change between M and IM
mmu-mir-1934	?		1	o change between M and IM
mmu-mir-466m	?		1	o change between M and IM
mmu-mir-3110	?		1	o change between M and IM
mmu-mir-3472	?		1	o change between M and IM
mmu-mir-5114	?		1	o change between M and IM
mmu-mir-5116	?		1	o change between M and IM
mmu-mir-5127	?		1	o change between M and IM

mmu-mir-5134	?	1	o change between M and IM
mmu-mir-5618	?	1	o change between M and IM
mmu-mir-5710	?	1	o change between M and IM
mmu-mir-6241	?	1	o change between M and IM
mmu-mir-6353	?	1	o change between M and IM
mmu-mir-6360	?	1	o change between M and IM
mmu-mir-145b	?	1	o change between M and IM
mmu-mir-6366	?	1	o change between M and IM
mmu-mir-6376	?	1	o change between M and IM
mmu-mir-6377	?	1	o change between M and IM
mmu-mir-6394	?	1	o change between M and IM
mmu-mir-6395	?	1	o change between M and IM
mmu-mir-451b	?	1	o change between M and IM
mmu-mir-451b	?	1	o change between M and IM
mmu-mir-6923	?	1	o change between M and IM
mmu-mir-6924	?	1	o change between M and IM
mmu-mir-6950	?	1	o change between M and IM
mmu-mir-6951	?	1	o change between M and IM
mmu-mir-6974	?	1	o change between M and IM
mmu-mir-6995	?	1	o change between M and IM
mmu-mir-6999	?	1	o change between M and IM
mmu-mir-7020	?	1	o change between M and IM
mmu-mir-7024	?	1	o change between M and IM
mmu-mir-7036	?	1	o change between M and IM
mmu-mir-7058	?	1	o change between M and IM
mmu-mir-7070	?	1	o change between M and IM
mmu-mir-7073	?	1	o change between M and IM
mmu-mir-7221	?	1	o change between M and IM
mmu-mir-7222	?	1	o change between M and IM
mmu-mir-7227	?	1	o change between M and IM
mmu-mir-7674	?	1	o change between M and IM
mmu-mir-216c	?	1	o change between M and IM
mmu-mir-8094	?	1	o change between M and IM
mmu-mir-8110	?	1	o change between M and IM
mmu-mir-8115	?	1	o change between M and IM
mmu-miR-99a-5p	?	1	o change between M and IM
mmu-miR-133a-3p	?	1	o change between M and IM
mmu-miR-196a-5p	?	1	o change between M and IM
mmu-miR-200a-3p	?	1	o change between M and IM
mmu-miR-340-3p	?	1	o change between M and IM
mmu-miR-363-3p	?	1	o change between M and IM
mmu-miR-433-5p	?	1	o change between M and IM
mmu-miR-291b-5p	?	1	o change between M and IM

mmu-miR-684	?	1	o change between M and IM
mmu-miR-713	?	1	o change between M and IM
mmu-miR-505-3p	?	1	o change between M and IM
mmu-miR-592-5p	?	1	o change between M and IM
mmu-miR-671-5p	?	1	o change between M and IM
mmu-miR-551b-3p	?	1	o change between M and IM
mmu-miR-22-5p	?	1	o change between M and IM
mmu-miR-339-3p	?	1	o change between M and IM
mmu-miR-743b-5p	?	1	o change between M and IM
mmu-miR-105	?	1	o change between M and IM
mmu-miR-343	?	1	o change between M and IM
mmu-miR-875-5p	?	1	o change between M and IM
mmu-miR-669k-3p	?	1	o change between M and IM
mmu-miR-1898	?	1	o change between M and IM
mmu-miR-1938	?	1	o change between M and IM
mmu-miR-344d-1-5p	?	1	o change between M and IM
mmu-miR-3066-5p	?	1	o change between M and IM
mmu-miR-3080-3p	?	1	o change between M and IM
mmu-miR-3101-3p	?	1	o change between M and IM
mmu-miR-206-5p	?	1	o change between M and IM
mmu-miR-96-3p	?	1	o change between M and IM
mmu-miR-34a-3p	?	1	o change between M and IM
mmu-miR-301b-5p	?	1	o change between M and IM
mmu-miR-544-5p	?	1	o change between M and IM
mmu-miR-598-5p	?	1	o change between M and IM
mmu-miR-466i-5p	?	1	o change between M and IM
mmu-miR-1948-5p	?	1	o change between M and IM
mmu-miR-5104	?	1	o change between M and IM
mmu-miR-5118	?	1	o change between M and IM
mmu-miR-5625-5p	?	1	o change between M and IM
mmu-miR-6237	?	1	o change between M and IM
mmu-miR-6387	?	1	o change between M and IM
mmu-miR-5709-3p	?	1	o change between M and IM
mmu-miR-6912-3p	?	1	o change between M and IM
mmu-miR-6915-3p	?	1	o change between M and IM
mmu-miR-6994-3p	?	1	o change between M and IM
mmu-miR-7001-3p	?	1	o change between M and IM
mmu-miR-7026-3p	?	1	o change between M and IM
mmu-miR-7074-3p	?	1	o change between M and IM
mmu-miR-7078-3p	?	1	o change between M and IM
mmu-miR-7081-3p	?	1	o change between M and IM
mmu-miR-7211-5p	?	1	o change between M and IM
mmu-miR-7231-5p	?	1	o change between M and IM

mmu-miR-7665-5p	?	1	o change between M and IM
mmu-miR-3569-5p	?	1	o change between M and IM
mmu-miR-7676-5p	?	1	o change between M and IM
mmu-miR-1191b-5p	?	1	o change between M and IM
mmu-miR-465d-3p	?	1	o change between M and IM
mmu-miR-6980-5p	0.403001	-1.54873	M down vs IM
mmu-miR-3097-5p	0.706789	-1.08486	M down vs IM
mmu-mir-1929	0.706853	-1.09209	M down vs IM
mmu-miR-411-5p	0.706853	-1.09209	M down vs IM
mmu-mir-449c	0.706853	-1.09209	M down vs IM
mmu-mir-1187	0.706853	-1.09209	M down vs IM
mmu-miR-5120	0.706853	-1.09209	M down vs IM
mmu-mir-6961	0.707999	-1.09752	M down vs IM
mmu-mir-3966	0.921508	1.04349	M up vs IM
mmu-miR-351-3p	0.553856	-1.25175	M down vs IM
mmu-mir-466h	0.39141	-1.24424	M down vs IM
mmu-mir-7673	0	-1.09752	M down vs IM
mmu-miR-450b-3p	0.781726	-1.13959	M down vs IM
mmu-mir-3084-1	1	1	o change between M and IM
mmu-mir-3084-2	1	1	o change between M and IM
mmu-mir-743a	0.0874062	1.30637	M up vs IM
mmu-miR-7655-5p	0.324445	1.0683	M up vs IM
mmu-mir-881	0.639191	1.02378	M up vs IM
mmu-mir-34b	0.832267	-1.10378	M down vs IM
mmu-miR-6960-3p	1	1	o change between M and IM
mmu-mir-546	0.721619	-1.09209	M down vs IM
mmu-miR-374b-3p	0.640486	1.03105	M up vs IM
mmu-miR-344h-3p	0.640486	1.03105	M up vs IM
mmu-miR-3473e	1	1	o change between M and IM
mmu-mir-5623	1	1	o change between M and IM
mmu-miR-137-3p	0.913679	1.04378	M up vs IM
mmu-mir-2139	0.925491	1.03762	M up vs IM
mmu-mir-409	0.862787	-1.03965	M down vs IM
mmu-miR-3572-3p	0.667437	1.06229	M up vs IM
mmu-miR-1949	0.603471	1.03105	M up vs IM
mmu-miR-5616-5p	0.603471	1.03105	M up vs IM
mmu-miR-880-5p	0.613717	1.03407	M up vs IM
mmu-mir-7025	1	1	o change between M and IM
mmu-mir-135a-2	0.617675	1.03694	M up vs IM
mmu-miR-7662-5p	0.0588326	1.23668	M up vs IM
mmu-miR-6385	0	-1.09752	M down vs IM
mmu-mir-8100	1	1	o change between M and IM
mmu-mir-1933	0	-1.27623	M down vs IM

mmu-mir-6930	0	-1.27623	M down vs IM
mmu-miR-5624-5p	1	1	o change between M and IM
mmu-miR-6941-5p	0.911852	1.04349	M up vs IM
mmu-mir-666	0.776438	-1.1293	M down vs IM
mmu-mir-101b	1	1	o change between M and IM
mmu-mir-448	0.165957	-1.09209	M down vs IM
mmu-miR-204-3p	0.217546	1.0683	M up vs IM
mmu-miR-28b	1	1	o change between M and IM
mmu-mir-3470a	0.178813	-1.08486	M down vs IM
mmu-miR-540-5p	0.121536	-1.1293	M down vs IM
mmu-mir-199a-1	0.130259	-1.1199	M down vs IM
mmu-miR-34c-3p	0.178813	-1.08486	M down vs IM
mmu-mir-486	0.809243	-1.09209	M down vs IM
mmu-mir-466b-1	0.559705	1.03105	M up vs IM
mmu-miR-299a-5p	0.202174	1.11889	M up vs IM
mmu-mir-8117	0.297613	1.07589	M up vs IM
mmu-mir-466b-5	0.297613	1.07589	M up vs IM
mmu-mir-466b-7	0.297613	1.07589	M up vs IM
mmu-miR-3095-3p	0.264185	1.08743	M up vs IM
mmu-miR-6359	0.26496	1.08713	M up vs IM
mmu-mir-693	0.297613	1.07589	M up vs IM
mmu-miR-6404	0.559705	1.03105	M up vs IM
mmu-mir-7677	0.324804	1.07904	M up vs IM
mmu-miR-374c-5p	0.925708	1.03407	M up vs IM
mmu-miR-2139	0.798483	-1.09856	M down vs IM
mmu-miR-5123	0.650539	1.07589	M up vs IM
mmu-miR-8090	0.426351	1.10835	M up vs IM
mmu-miR-6412	0.105725	-1.30071	M down vs IM
mmu-miR-3967	0.165468	1.12729	M up vs IM
mmu-miR-6972-3p	0.513112	1.03105	M up vs IM
mmu-mir-6955	0.550716	1.03694	M up vs IM
mmu-mir-125b-1	0.0412283	1.09134	M up vs IM
mmu-miR-190b-3p	0.530943	1.03407	M up vs IM
mmu-miR-6939-3p	0.530943	1.03407	M up vs IM
mmu-mir-721	0.227824	1.0852	M up vs IM
mmu-mir-495	0.202102	-1.09752	M down vs IM
mmu-miR-30a-5p	0.225534	-1.08618	M down vs IM
mmu-mir-3965	1	1	o change between M and IM
mmu-miR-7013-3p	1	1	o change between M and IM
mmu-miR-6950-5p	0.147792	-1.13807	M down vs IM
mmu-miR-1981-3p	0.180474	-1.11066	M down vs IM
mmu-mir-5124a	1	1	o change between M and IM
mmu-mir-7219	1	1	o change between M and IM

mmu-miR-291a-5p	1	1	o change between M and IM
mmu-miR-1895	1	1	o change between M and IM
mmu-mir-495	0.202102	-1.09752	M down vs IM
mmu-mir-1192	0.225534	-1.08618	M down vs IM
mmu-miR-7073-5p	0.225534	-1.08618	M down vs IM
mmu-miR-568	0.396529	1.04349	M up vs IM
mmu-mir-105	1	1	o change between M and IM
mmu-mir-6973a	1	1	o change between M and IM
mmu-mir-5625	0.206781	-1.11375	M down vs IM
mmu-mir-7659	1	1	o change between M and IM
mmu-miR-6986-5p	0.858623	-1.09209	M down vs IM
mmu-mir-7230	1	1	o change between M and IM
mmu-mir-1b	0.86722	-1.0592	M down vs IM
mmu-mir-690	0.0942819	-1.25175	M down vs IM
mmu-miR-6932-5p	0.0803361	1.3022	M up vs IM
mmu-mir-6993	0.0995752	1.23668	M up vs IM
mmu-miR-450a-2-3p	0.0918576	1.2593	M up vs IM
mmu-miR-200b-3p	0.231338	-1.09209	M down vs IM
mmu-mir-27a	1	1	o change between M and IM
mmu-mir-452	0.248404	-1.08486	M down vs IM
mmu-miR-7034-5p	0.219982	-1.09752	M down vs IM
mmu-mir-1927	1	1	o change between M and IM
mmu-miR-6384	0.16135	-1.13807	M down vs IM
mmu-miR-202-5p	0.245108	-1.08618	M down vs IM
mmu-mir-3061	1	1	o change between M and IM
mmu-miR-6364	0.248404	-1.08486	M down vs IM
mmu-mir-154	1	1	o change between M and IM
mmu-mir-302b	1	1	o change between M and IM
mmu-mir-743b	1	1	o change between M and IM
mmu-mir-876	1	1	o change between M and IM
mmu-mir-6365	1	1	o change between M and IM
mmu-mir-6393	1	1	o change between M and IM
mmu-mir-6921	1	1	o change between M and IM
mmu-mir-6962	1	1	o change between M and IM
mmu-mir-6973b	1	1	o change between M and IM
mmu-mir-7026	1	1	o change between M and IM
mmu-miR-540-3p	1	1	o change between M and IM
mmu-miR-692	1	1	o change between M and IM
mmu-miR-301b-3p	1	1	o change between M and IM
mmu-miR-1893	1	1	o change between M and IM
mmu-miR-6899-5p	1	1	o change between M and IM
mmu-miR-6911-3p	1	1	o change between M and IM
mmu-miR-654-3p	0.908709	1.04349	M up vs IM

mmu-mir-214	0.248404	-1.08486	M down vs IM
mmu-miR-6376	0.484056	1.03407	M up vs IM
mmu-miR-344-5p	0.505647	1.03694	M up vs IM
mmu-miR-3094-3p	0.288945	-1.0772	M down vs IM
mmu-miR-6241	1	1	o change between M and IM
mmu-miR-466l-3p	0.0899195	-1.29062	M down vs IM
mmu-mir-3109	0.105814	1.2414	M up vs IM
mmu-mir-5132	0.174554	-1.13766	M down vs IM
mmu-miR-6952-3p	0.184576	-1.1293	M down vs IM
mmu-miR-6989-3p	1	1	o change between M and IM
mmu-miR-195b	0.410338	1.04945	M up vs IM
mmu-miR-5121	0.184576	-1.1293	M down vs IM
mmu-miR-7669-3p	0.184576	-1.1293	M down vs IM
mmu-mir-5107	0.265844	1.0852	M up vs IM
mmu-miR-686	0.288945	-1.0772	M down vs IM
mmu-mir-7051	1	1	o change between M and IM
mmu-mir-291a	1	1	o change between M and IM
mmu-mir-291a	1	1	o change between M and IM
mmu-let-7c-2	1	1	o change between M and IM
mmu-let-7c-2	1	1	o change between M and IM
mmu-mir-877	1	1	o change between M and IM
mmu-mir-6912	1	1	o change between M and IM
mmu-mir-7093	1	1	o change between M and IM
mmu-mir-692-1	1	1	o change between M and IM
mmu-miR-683	0.263255	-1.08618	M down vs IM
mmu-miR-7232-3p	0.236635	-1.09752	M down vs IM
mmu-miR-7000-3p	1	1	o change between M and IM
mmu-miR-7220-5p	1	1	o change between M and IM
mmu-miR-7218-3p	0.904444	-1.09752	M down vs IM
mmu-miR-3074-5p	1	1	o change between M and IM
mmu-mir-125a	0.89049	1.05613	M up vs IM
mmu-miR-6394	0.25385	1.09584	M up vs IM
mmu-miR-1982-3p	0.114314	1.23668	M up vs IM
mmu-mir-6352	0.262659	-1.09209	M down vs IM
mmu-miR-702-3p	0.18446	-1.13807	M down vs IM
mmu-mir-6956	0.468549	1.04349	M up vs IM
mmu-miR-7041-5p	1	1	o change between M and IM
mmu-mir-3620	0.208931	-1.1199	M down vs IM
mmu-miR-1956	0.208931	-1.1199	M down vs IM
mmu-mir-25	0.224148	-1.11066	M down vs IM
mmu-mir-496b	0.224148	-1.11066	M down vs IM
mmu-miR-6415	1	1	o change between M and IM
mmu-mir-7216	0.24815	1.09841	M up vs IM

mmu-miR-3073b-5p	0.400633	1.05439	M up vs IM
mmu-mir-6920	0.468549	1.04349	M up vs IM
mmu-miR-5116	1	1	o change between M and IM
mmu-mir-6958	1	1	o change between M and IM
mmu-mir-471	1	1	o change between M and IM
mmu-mir-6378	1	1	o change between M and IM
mmu-mir-7009	1	1	o change between M and IM
mmu-miR-7214-3p	1	1	o change between M and IM
mmu-miR-21a-5p	0.563991	-1.32796	M down vs IM
mmu-mir-700	0	1.30721	M up vs IM
mmu-miR-6902-3p	0.58779	1.15244	M up vs IM
mmu-miR-19b-2-5p	1	1	o change between M and IM
mmu-miR-7044-3p	0	-1.09752	M down vs IM
mmu-mir-6942	0.798258	-1.09752	M down vs IM
mmu-miR-6974-3p	1	1	o change between M and IM
mmu-miR-495-5p	0.850242	-1.08618	M down vs IM
mmu-mir-669h	0	1.08118	M up vs IM
mmu-mir-1946b	0	1.02301	M up vs IM
mmu-mir-3112	0	1.02301	M up vs IM
mmu-mir-5118	0	-1.26159	M down vs IM
mmu-miR-99a-3p	0	1.02301	M up vs IM
mmu-miR-466l-5p	0	1.02301	M up vs IM
mmu-mir-376b	0	1.02378	M up vs IM
mmu-mir-759	0	1.02378	M up vs IM
mmu-miR-298-3p	0	1.02378	M up vs IM
mmu-miR-6992-5p	0	1.12453	M up vs IM
mmu-mir-1224	0	1.0744	M up vs IM
mmu-mir-466d	0	-1.08486	M down vs IM
mmu-mir-3470b	0	1.02378	M up vs IM
mmu-mir-21c	0	-1.09389	M down vs IM
mmu-mir-6907	0	1.02378	M up vs IM
mmu-mir-7006	0	-1.26064	M down vs IM
mmu-mir-8097	0	-1.09389	M down vs IM
mmu-miR-152-3p	0	1.06229	M up vs IM
mmu-miR-148b-3p	0	1.17984	M up vs IM
mmu-miR-196a-2-3p	0	1.0683	M up vs IM
mmu-miR-337-5p	0	1.26608	M up vs IM
mmu-miR-1899	0	1.06229	M up vs IM
mmu-miR-1929-5p	0	1.06007	M up vs IM
mmu-miR-1958	0	1.111	M up vs IM
mmu-miR-3099-5p	0	1.2414	M up vs IM
mmu-miR-146a-3p	0	1.2414	M up vs IM
mmu-miR-185-3p	0	1.02378	M up vs IM

mmu-miR-103-1-5p	0	1.02378	M up vs IM
mmu-miR-302d-5p	0	1.08199	M up vs IM
mmu-miR-6540-3p	0	1.02378	M up vs IM
mmu-miR-6907-3p	0	1.02378	M up vs IM
mmu-miR-7037-3p	0	1.0683	M up vs IM
mmu-miR-7216-3p	0	1.06229	M up vs IM
mmu-mir-24-1	0	-1.06673	M down vs IM
mmu-mir-30d	0	1.02378	M up vs IM
mmu-mir-1983	0	1.02378	M up vs IM
mmu-mir-133c	0	1.02378	M up vs IM
mmu-mir-7681	0	1.02378	M up vs IM
mmu-miR-451a	0	1.58484	M up vs IM
mmu-miR-708-3p	0	1.02378	M up vs IM
mmu-miR-455-3p	0	-1.11164	M down vs IM
mmu-miR-3103-3p	0	1.07946	M up vs IM
mmu-miR-122-3p	0	1.0744	M up vs IM
mmu-miR-6957-5p	0	-1.08486	M down vs IM
mmu-miR-6965-5p	0	1.02378	M up vs IM
mmu-miR-8094	0	-1.43418	M down vs IM
mmu-miR-382-5p	0.872362	1.0852	M up vs IM
mmu-mir-3107	0.84112	1.07589	M up vs IM
mmu-miR-7041-3p	1	1	o change between M and IM
mmu-miR-7661-3p	0.744205	1.03407	M up vs IM
mmu-miR-7049-5p	0.405261	1.11254	M up vs IM
mmu-mir-7088	0	-1.09209	M down vs IM
mmu-miR-30b-5p	0	-1.09209	M down vs IM
mmu-mir-327	1	1	o change between M and IM
mmu-miR-7654-5p	1	1	o change between M and IM
mmu-miR-6516-5p	0.746062	-1.15244	M down vs IM
mmu-miR-382-3p	0.846693	-1.08618	M down vs IM
mmu-mir-378b	0.264176	-1.1293	M down vs IM
mmu-miR-22-3p	1	1	o change between M and IM
mmu-miR-467b-3p	0.804386	-1.09752	M down vs IM
mmu-miR-1960	1	1	o change between M and IM
mmu-miR-491-3p	0.938068	1.06229	M up vs IM
mmu-mir-465d	1	1	o change between M and IM
mmu-mir-141	0	1.03105	M up vs IM
mmu-mir-15a	0	1.03105	M up vs IM
mmu-mir-29c	0	1.03105	M up vs IM
mmu-mir-107	0	1.03105	M up vs IM
mmu-mir-6238	0	1.03105	M up vs IM
mmu-mir-7224	0	1.03105	M up vs IM
mmu-miR-154-5p	0	1.03105	M up vs IM

mmu-miR-449c-5p	0	1.03105	M up vs IM
mmu-miR-743a-3p	0	1.34637	M up vs IM
mmu-miR-6541	0	1.03105	M up vs IM
mmu-mir-129-1	0	1.03105	M up vs IM
mmu-mir-129-1	0	1.03105	M up vs IM
mmu-mir-30c-1	0	1.27507	M up vs IM
mmu-mir-323	0	1.2984	M up vs IM
mmu-mir-673	0	1.27507	M up vs IM
mmu-mir-711	0	1.03105	M up vs IM
mmu-mir-3070a	0	1.07589	M up vs IM
mmu-mir-5101	0	1.27507	M up vs IM
mmu-mir-6351	0	1.03105	M up vs IM
mmu-mir-6364	0	-1.09529	M down vs IM
mmu-mir-6916	0	1.09527	M up vs IM
mmu-miR-30e-3p	0	1.18822	M up vs IM
mmu-miR-192-5p	0	1.07589	M up vs IM
mmu-miR-328-3p	0	1.27507	M up vs IM
mmu-miR-99b-3p	0	1.03105	M up vs IM
mmu-miR-124-5p	0	1.03105	M up vs IM
mmu-miR-183-3p	0	-1.09529	M down vs IM
mmu-miR-331-5p	0	1.07589	M up vs IM
mmu-miR-125b-1-3p	0	1.07589	M up vs IM
mmu-miR-876-3p	0	-1.08486	M down vs IM
mmu-miR-1903	0	1.27507	M up vs IM
mmu-miR-3088-3p	0	1.12729	M up vs IM
mmu-miR-201-3p	0	1.03105	M up vs IM
mmu-miR-208a-5p	0	1.2984	M up vs IM
mmu-miR-25-5p	0	1.07589	M up vs IM
mmu-miR-19b-1-5p	0	1.03105	M up vs IM
mmu-miR-551b-5p	0	1.27507	M up vs IM
mmu-miR-5619-5p	0	-1.05219	M down vs IM
mmu-miR-6338	0	1.03105	M up vs IM
mmu-miR-6344	0	-1.08618	M down vs IM
mmu-miR-6355	0	1.07589	M up vs IM
mmu-miR-130c	0	1.07589	M up vs IM
mmu-miR-6392-3p	0	1.07589	M up vs IM
mmu-miR-7006-3p	0	-1.07054	M down vs IM
mmu-miR-7014-5p	0	1.07589	M up vs IM
mmu-miR-7063-3p	0	1.07589	M up vs IM
mmu-miR-7075-3p	0	1.03105	M up vs IM
mmu-miR-7223-3p	0	1.03105	M up vs IM
mmu-miR-7243-5p	0	-1.08618	M down vs IM
mmu-miR-497b	0	1.03105	M up vs IM

mmu-mir-193a	0	1.03105	M up vs IM
mmu-mir-129-2	0	1.03105	M up vs IM
mmu-mir-687	0	1.03105	M up vs IM
mmu-mir-702	0	1.07589	M up vs IM
mmu-mir-421	0	1.08713	M up vs IM
mmu-mir-1902	0	1.03105	M up vs IM
mmu-mir-669l	0	1.03105	M up vs IM
mmu-mir-3060	0	1.03105	M up vs IM
mmu-mir-3066	0	1.07589	M up vs IM
mmu-mir-3471-1	0	1.03105	M up vs IM
mmu-mir-5128	0	1.08713	M up vs IM
mmu-mir-6354	0	1.07589	M up vs IM
mmu-mir-6370	0	1.03105	M up vs IM
mmu-mir-6407	0	1.03105	M up vs IM
mmu-mir-6410	0	1.27507	M up vs IM
mmu-mir-6937	0	1.08713	M up vs IM
mmu-mir-7054	0	1.07589	M up vs IM
mmu-mir-7215	0	1.03105	M up vs IM
mmu-mir-7669	0	-1.08618	M down vs IM
mmu-mir-8098	0	1.07589	M up vs IM
mmu-miR-138-5p	0	1.40556	M up vs IM
mmu-miR-377-3p	0	1.03105	M up vs IM
mmu-miR-302b-3p	0	-1.08486	M down vs IM
mmu-miR-712-5p	0	1.2984	M up vs IM
mmu-miR-27b-5p	0	-1.07721	M down vs IM
mmu-miR-340-5p	0	-1.08618	M down vs IM
mmu-miR-335-3p	0	1.27507	M up vs IM
mmu-miR-466h-5p	0	1.07589	M up vs IM
mmu-miR-467b-5p	0	1.08203	M up vs IM
mmu-miR-3068-5p	0	-1.07721	M down vs IM
mmu-miR-3081-3p	0	1.03105	M up vs IM
mmu-miR-3093-5p	0	1.03105	M up vs IM
mmu-miR-152-5p	0	1.03105	M up vs IM
mmu-miR-192-3p	0	1.07589	M up vs IM
mmu-miR-6346	0	1.03105	M up vs IM
mmu-miR-6391	0	1.03105	M up vs IM
mmu-miR-6898-5p	0	1.03105	M up vs IM
mmu-miR-6921-3p	0	1.03105	M up vs IM
mmu-miR-6973a-5p	0	1.08203	M up vs IM
mmu-miR-6998-5p	0	1.37392	M up vs IM
mmu-miR-6769b-3p	0	1.03105	M up vs IM
mmu-miR-7090-5p	0	1.03105	M up vs IM
mmu-miR-7219-3p	0	-1.28229	M down vs IM

mmu-mir-29a	0.005746	1.03105	M up vs IM
mmu-miR-7078-5p	0.785653	-1.1199	M down vs IM
mmu-miR-7233-5p	0.374547	1.13899	M up vs IM
mmu-mir-92a-1	0.8841	1.06229	M up vs IM
mmu-mir-466j	0.060686	1.03105	M up vs IM
mmu-miR-6922-3p	0.00671279	1.3195	M up vs IM
mmu-mir-3106	0.0215183	1.09031	M up vs IM
mmu-mir-3083	0.0161443	1.12217	M up vs IM
mmu-mir-878	0.0554186	1.03407	M up vs IM
mmu-mir-7000	0.0177294	-1.11066	M down vs IM
mmu-miR-292b-3p	0.835927	1.04349	M up vs IM
mmu-miR-3059-5p	1	1	o change between M and IM
mmu-mir-467h	0.722656	-1.08618	M down vs IM
mmu-mir-9-2	0	1.13059	M up vs IM
mmu-mir-135a-1	0	-1.09209	M down vs IM
mmu-mir-325	0	1.03407	M up vs IM
mmu-mir-329	0	1.0852	M up vs IM
mmu-mir-199a-2	0	1.03407	M up vs IM
mmu-mir-384	0	1.0852	M up vs IM
mmu-mir-694	0	1.03407	M up vs IM
mmu-mir-879	0	1.0852	M up vs IM
mmu-mir-18b	0	1.09286	M up vs IM
mmu-mir-568	0	1.07904	M up vs IM
mmu-mir-872	0	1.13624	M up vs IM
mmu-mir-1899	0	1.0852	M up vs IM
mmu-mir-1967	0	-1.22192	M down vs IM
mmu-mir-432	0	-1.06741	M down vs IM
mmu-mir-3105	0	1.03407	M up vs IM
mmu-mir-3962	0	1.0852	M up vs IM
mmu-mir-344h-1	0	1.48601	M up vs IM
mmu-mir-344h-2	0	1.48601	M up vs IM
mmu-mir-7085	0	1.03407	M up vs IM
mmu-mir-6715	0	-1.09209	M down vs IM
mmu-mir-8096	0	1.03407	M up vs IM
mmu-mir-3535	0	1.03407	M up vs IM
mmu-miR-323-3p	0	1.07904	M up vs IM
mmu-miR-324-3p	0	1.03407	M up vs IM
mmu-miR-330-3p	0	1.09031	M up vs IM
mmu-miR-653-5p	0	1.0852	M up vs IM
mmu-miR-664-3p	0	1.60077	M up vs IM
mmu-miR-669p-3p	0	1.03407	M up vs IM
mmu-miR-205-3p	0	-1.08301	M down vs IM
mmu-miR-212-5p	0	1.0852	M up vs IM

mmu-miR-3961	0	1.03407	M up vs IM
mmu-miR-6948-3p	0	1.03407	M up vs IM
mmu-miR-6963-5p	0	1.03407	M up vs IM
mmu-miR-6981-3p	0	1.20842	M up vs IM
mmu-miR-6995-3p	0	-1.09209	M down vs IM
mmu-miR-7005-3p	0	-1.10058	M down vs IM
mmu-miR-7210-5p	0	1.03407	M up vs IM
mmu-miR-8092	0	-1.29062	M down vs IM
mmu-mir-7657	0.881609	1.05927	M up vs IM
mmu-mir-669m-1	0.458157	1.06229	M up vs IM
mmu-miR-674-3p	0.838179	-1.10178	M down vs IM
mmu-miR-292-5p	1	1	o change between M and IM
mmu-miR-6996-3p	0.834835	1.0852	M up vs IM
mmu-miR-666-3p	0.0189878	-1.09752	M down vs IM
mmu-miR-3058-3p	0.0137023	-1.13766	M down vs IM
mmu-miR-6906-3p	0.621548	-1.26903	M down vs IM
mmu-mir-7082	1	1	o change between M and IM
mmu-mir-299b	0.0509902	1.03709	M up vs IM
mmu-miR-1b-3p	0.219392	1.10835	M up vs IM
mmu-mir-136	0	1.03694	M up vs IM
mmu-mir-151	0	1.11563	M up vs IM
mmu-mir-208a	0	1.09334	M up vs IM
mmu-mir-5130	0	1.03694	M up vs IM
mmu-mir-6361	0	1.09334	M up vs IM
mmu-mir-6388	0	1.17068	M up vs IM
mmu-miR-141-5p	0	-1.08906	M down vs IM
mmu-miR-5618-5p	0	-1.08001	M down vs IM
mmu-miR-6916-3p	0	1.09334	M up vs IM
mmu-miR-6937-3p	0	1.03694	M up vs IM
mmu-miR-6972-5p	0.194316	1.2593	M up vs IM
mmu-mir-344c	1	1	o change between M and IM
mmu-mir-7050	1	1	o change between M and IM
mmu-miR-450a-1-3p	0.219983	-1.09752	M down vs IM
mmu-miR-3106-3p	0.654172	1.2414	M up vs IM
mmu-mir-124-3	0.282813	1.05033	M up vs IM
mmu-mir-124-1	0.282813	1.05033	M up vs IM
mmu-mir-124-2	0.282813	1.05033	M up vs IM
mmu-miR-6928-3p	0.730083	-1.15244	M down vs IM
mmu-miR-490-3p	0.836282	-1.08618	M down vs IM
mmu-miR-144-3p	0.836282	-1.08618	M down vs IM
mmu-miR-374c-3p	0.588665	-1.1199	M down vs IM
mmu-mir-143	0.682591	1.0852	M up vs IM
mmu-mir-98	1	1	o change between M and IM

mmu-mir-1928	1	1	o change between M and IM
mmu-miR-6903-5p	1	1	o change between M and IM
mmu-mir-6481	1	1	o change between M and IM
mmu-miR-7224-5p	0.488256	1.16861	M up vs IM
mmu-mir-299a	0	-1.1293	M down vs IM
mmu-mir-8099-1	0	1.07841	M up vs IM
mmu-mir-8099-2	0	1.07841	M up vs IM
mmu-miR-695	0	1.03931	M up vs IM
mmu-miR-872-5p	0	1.03931	M up vs IM
mmu-miR-5110	0	1.33255	M up vs IM
mmu-miR-6418-5p	0	-1.28767	M down vs IM
mmu-miR-6930-3p	0	1.13632	M up vs IM
mmu-miR-467h	0.120739	1.08713	M up vs IM
mmu-miR-1947-3p	0.113571	1.09334	M up vs IM
mmu-miR-6956-3p	0.582842	1.02378	M up vs IM
mmu-miR-6379	0.794765	1.03105	M up vs IM
mmu-miR-425-3p	0.0572986	1.42341	M up vs IM
mmu-miR-710	1	1	o change between M and IM
mmu-miR-344b-3p	1	1	o change between M and IM
mmu-miR-871-3p	1	1	o change between M and IM
mmu-miR-654-5p	1	1	o change between M and IM
mmu-miR-6715-3p	0.605392	-1.27623	M down vs IM
mmu-miR-30a-3p	0.849377	-1.08589	M down vs IM
mmu-miR-6915-5p	1	1	o change between M and IM
mmu-miR-1904	0.610567	1.02378	M up vs IM
mmu-miR-6400	0.333667	-1.05967	M down vs IM
mmu-miR-6362	0.610567	1.02378	M up vs IM
mmu-let-7g	0.274654	-1.47077	M down vs IM
mmu-mir-423	0.606269	-1.27623	M down vs IM
mmu-mir-3058	0.352765	1.06149	M up vs IM
mmu-miR-7058-3p	0.634056	1.02378	M up vs IM
mmu-miR-691	0.297967	1.0744	M up vs IM
mmu-miR-708-5p	0.841514	-1.09752	M down vs IM
mmu-miR-669l-5p	0.0803572	1.33774	M up vs IM
mmu-miR-7065-3p	0.34921	1.06229	M up vs IM
mmu-mir-6900	0.34921	1.06229	M up vs IM
mmu-mir-7084	0.846923	-1.08589	M down vs IM
mmu-miR-3086-5p	0.348801	1.15244	M up vs IM
mmu-miR-6348	0.840759	1.0852	M up vs IM
mmu-miR-370-3p	1	1	o change between M and IM
mmu-miR-546	0.936383	1.03407	M up vs IM
mmu-mir-6408	0.842715	-1.08618	M down vs IM
mmu-miR-673-3p	0.308922	1.06229	M up vs IM

mmu-miR-344d-3p	0.87401	-1.06856	M down vs IM
mmu-mir-1957b	0.803258	-1.08486	M down vs IM
mmu-miR-181b-1-3p	0.755727	1.10849	M up vs IM
mmu-miR-7057-5p	1	1	o change between M and IM
mmu-mir-184	1	1	o change between M and IM
mmu-miR-1943-5p	0.790135	-1.1293	M down vs IM
mmu-miR-760-5p	1	1	o change between M and IM
mmu-miR-693-5p	0.529027	1.03105	M up vs IM
mmu-miR-7117-5p	0.529027	1.03105	M up vs IM
mmu-mir-6367	0.529026	1.03105	M up vs IM
mmu-mir-466j	0.529026	1.03105	M up vs IM
mmu-mir-6387	0.348429	1.07235	M up vs IM
mmu-miR-701-3p	0.554148	1.03105	M up vs IM
mmu-mir-7664	0.554148	1.03105	M up vs IM
mmu-mir-3968	0.554148	1.03105	M up vs IM
mmu-miR-3473g	0.187164	1.12729	M up vs IM
mmu-miR-155-3p	0.212679	-1.09209	M down vs IM
mmu-miR-764-5p	0.243305	1.07904	M up vs IM
mmu-miR-3079-5p	0.251548	1.12268	M up vs IM
mmu-miR-148a-5p	0.435035	-1.1293	M down vs IM
mmu-miR-493-3p	1	1	o change between M and IM
mmu-miR-7045-3p	1	1	o change between M and IM
mmu-miR-7657-3p	1	1	o change between M and IM
mmu-mir-425	0.942671	1.03105	M up vs IM
mmu-miR-1839-5p	0.84602	1.08713	M up vs IM
mmu-miR-142-3p	0.573187	1.03105	M up vs IM
mmu-mir-1903	0.573187	1.03105	M up vs IM
mmu-mir-292b	0.28959	1.08203	M up vs IM
mmu-miR-1188-5p	0.830638	1.09334	M up vs IM
mmu-mir-763	0.856479	1.06229	M up vs IM
mmu-miR-7242-5p	0.137328	1.20842	M up vs IM
mmu-mir-3088	0.265844	1.0852	M up vs IM
mmu-mir-744	0.802195	-1.09752	M down vs IM
mmu-miR-129-1-3p	0.335544	1.07589	M up vs IM
mmu-mir-7213	0.335544	1.07589	M up vs IM
mmu-mir-6336	0.551119	1.03694	M up vs IM
mmu-mir-3101	0.853886	1.07589	M up vs IM
mmu-miR-8093	0.433987	-1.13807	M down vs IM
mmu-mir-1a-1	1	1	o change between M and IM
mmu-mir-466l	1	1	o change between M and IM
mmu-miR-500-3p	0.563225	1.3195	M up vs IM
mmu-mir-6971	1	1	o change between M and IM
mmu-miR-6946-5p	0.544627	1.03407	M up vs IM

mmu-mir-7683	0.240188	1.08821	M up vs IM
mmu-miR-18a-3p	0.474683	1.03694	M up vs IM
mmu-mir-7656	0.52785	1.26329	M up vs IM
mmu-miR-7677-3p	0.210139	1.12268	M up vs IM
mmu-miR-7666-3p	0.602029	-1.28065	M down vs IM
mmu-mir-141	0	-1.06039	M down vs IM
mmu-mir-192	0	1.05613	M up vs IM
mmu-mir-1946a	0	1.05613	M up vs IM
mmu-mir-3097	0	1.05613	M up vs IM
mmu-mir-8102	0	1.05613	M up vs IM
mmu-miR-1968-5p	0	1.05613	M up vs IM
mmu-miR-3057-5p	0	1.11357	M up vs IM
mmu-miR-3074-1-3p	0	1.05613	M up vs IM
mmu-miR-130a-5p	0	1.05613	M up vs IM
mmu-miR-365-2-5p	0	1.11357	M up vs IM
mmu-miR-6913-3p	0	1.05613	M up vs IM
mmu-miR-7022-5p	0	1.28063	M up vs IM
mmu-miR-219b-5p	0	1.05613	M up vs IM
mmu-mir-6913	0.218243	1.09841	M up vs IM
mmu-miR-6335	0.386696	-1.27623	M down vs IM
mmu-miR-7233-3p	0.794215	-1.1199	M down vs IM
mmu-miR-1894-3p	0.868999	-1.15179	M down vs IM
mmu-miR-8114	0.869673	1.03105	M up vs IM
mmu-mir-708	0.356088	1.0852	M up vs IM
mmu-miR-1941-5p	0.54349	-1.10378	M down vs IM
mmu-miR-669e-5p	0.428844	-1.15244	M down vs IM
mmu-mir-344i	1	1	o change between M and IM
mmu-mir-344i	1	1	o change between M and IM
mmu-miR-384-3p	1	1	o change between M and IM
mmu-mir-7661	0.743526	1.04945	M up vs IM
mmu-miR-6341	0.524179	-1.11066	M down vs IM
mmu-mir-7034	1	1	o change between M and IM
mmu-miR-679-5p	1	1	o change between M and IM
mmu-mir-882	0.548735	-1.10198	M down vs IM
mmu-mir-7060	1	1	o change between M and IM
mmu-miR-6997-5p	1	1	o change between M and IM
mmu-mir-701	1	1	o change between M and IM
mmu-miR-434-3p	0.56359	1.33456	M up vs IM
mmu-mir-5129	0.431286	1.3022	M up vs IM
mmu-miR-712-3p	0.563906	1.32317	M up vs IM
mmu-miR-6964-3p	0.548559	1.06229	M up vs IM
mmu-miR-452-5p	0.510433	1.06229	M up vs IM
mmu-mir-182	0	1.10849	M up vs IM

mmu-mir-101a	0	1.10849	M up vs IM
mmu-mir-3070a	0	1.10849	M up vs IM
mmu-mir-6373	0	1.06229	M up vs IM
mmu-mir-7057	0	1.06229	M up vs IM
mmu-mir-7062	0	1.06229	M up vs IM
mmu-mir-7652	0	1.06229	M up vs IM
mmu-miR-298-5p	0	1.06229	M up vs IM
mmu-miR-100-5p	0	1.06229	M up vs IM
mmu-miR-673-5p	0	1.06229	M up vs IM
mmu-miR-154-3p	0	1.06229	M up vs IM
mmu-miR-7b-3p	0	1.06229	M up vs IM
mmu-miR-3544-3p	0	1.31371	M up vs IM
mmu-miR-5617-3p	0	1.06229	M up vs IM
mmu-miR-6395	0	1.06229	M up vs IM
mmu-miR-6959-3p	0	1.06229	M up vs IM
mmu-mir-654	0.563443	1.06229	M up vs IM
mmu-mir-3073b	0	1.06229	M up vs IM
mmu-mir-6933	0	1.06229	M up vs IM
mmu-miR-379-5p	0	1.10225	M up vs IM
mmu-miR-431-3p	0	1.11481	M up vs IM
mmu-miR-6363	0	-1.07095	M down vs IM
mmu-miR-384-5p	0.294415	-1.27623	M down vs IM
mmu-miR-466m-3p	1	1	o change between M and IM
mmu-miR-3083-3p	1	1	o change between M and IM
mmu-miR-3077-3p	0.570979	-1.10198	M down vs IM
mmu-mir-33	0.624255	-1.08486	M down vs IM
mmu-miR-6988-5p	0.619886	-1.08618	M down vs IM
mmu-mir-6239	0.738488	1.05439	M up vs IM
mmu-miR-6903-3p	1	1	o change between M and IM
mmu-mir-27b	1	1	o change between M and IM
mmu-mir-27b	1	1	o change between M and IM
mmu-mir-380	1	1	o change between M and IM
mmu-mir-344-1	0.893739	1.03545	M up vs IM
mmu-mir-1291	0.846222	1.03694	M up vs IM
mmu-miR-144-5p	0.0356923	-1.05347	M down vs IM
mmu-mir-511	0.806807	1.06229	M up vs IM
mmu-miR-5621-3p	1	1	o change between M and IM
mmu-miR-5107-3p	0.19202	1.12602	M up vs IM
mmu-mir-3089	1	1	o change between M and IM
mmu-miR-25-3p	1	1	o change between M and IM
mmu-miR-882	0.298697	-1.29062	M down vs IM
mmu-miR-6357	0.298697	-1.29062	M down vs IM
mmu-mir-691	0.32153	-1.26355	M down vs IM

mmu-mir-6945	1	1	o change between M and IM
mmu-miR-383-3p	1	1	o change between M and IM
mmu-mir-6399	0.542166	-1.1199	M down vs IM
mmu-miR-5615-5p	0.772628	1.04945	M up vs IM
mmu-miR-6920-5p	1	1	o change between M and IM
mmu-mir-6404	0.61933	-1.09209	M down vs IM
mmu-miR-3100-3p	0.637936	-1.08618	M down vs IM
mmu-mir-6409	1	1	o change between M and IM
mmu-miR-6897-5p	1	1	o change between M and IM
mmu-miR-7652-5p	1	1	o change between M and IM
mmu-mir-296	0.333509	1.15384	M up vs IM
mmu-mir-24-1	0.753664	-1.06673	M down vs IM
mmu-miR-465a-5p	0.811051	1.08203	M up vs IM
mmu-miR-129b-5p	0.629535	-1.13766	M down vs IM
mmu-let-7f-2-3p	1	1	o change between M and IM
mmu-miR-21c	0.367768	-1.09713	M down vs IM
mmu-miR-1967	1	1	o change between M and IM
mmu-miR-6416-5p	0	-1.05611	M down vs IM
mmu-miR-3084-3p	0.322533	1.14089	M up vs IM
mmu-mir-211	0.865462	-1.08618	M down vs IM
mmu-mir-150	1	1	o change between M and IM
mmu-miR-7671-5p	0.302933	-1.30071	M down vs IM
mmu-mir-3113	1	1	o change between M and IM
mmu-miR-28c	0.616866	-1.09752	M down vs IM
mmu-mir-1198	1	1	o change between M and IM
mmu-mir-194-2	1	1	o change between M and IM
mmu-miR-374b-5p	1	1	o change between M and IM
mmu-miR-541-5p	1	1	o change between M and IM
mmu-miR-1912-5p	0.851924	1.06229	M up vs IM
mmu-miR-5623-5p	1	1	o change between M and IM
mmu-mir-7075	0.872124	1.03105	M up vs IM
mmu-miR-7237-5p	0.170796	1.32304	M up vs IM
mmu-mir-5709	0	-1.11773	M down vs IM
mmu-miR-7673-5p	0	1.03105	M up vs IM
mmu-miR-6352	0.186533	1.06229	M up vs IM
mmu-mir-6992	0.378207	-1.30071	M down vs IM
mmu-miR-466f-5p	0.14339	1.14276	M up vs IM
mmu-mir-204	0.876034	1.03407	M up vs IM
mmu-miR-5627-3p	1	1	o change between M and IM
mmu-miR-7090-3p	1	1	o change between M and IM
mmu-mir-6985	1	1	o change between M and IM
mmu-miR-7649-3p	0.620978	1.15244	M up vs IM
mmu-miR-142-5p	0.819242	1.04349	M up vs IM

mmu-miR-327	0.140421	1.17902	M up vs IM
mmu-mir-15b	0	1.07589	M up vs IM
mmu-mir-23a	0	1.12909	M up vs IM
mmu-mir-652	0	1.10196	M up vs IM
mmu-mir-6908	0	1.07589	M up vs IM
mmu-miR-208b-3p	0	1.07589	M up vs IM
mmu-mir-34a	0	1.07589	M up vs IM
mmu-mir-1960	0	1.30459	M up vs IM
mmu-mir-5620	0	1.12909	M up vs IM
mmu-mir-6984	0	1.35487	M up vs IM
mmu-miR-7057-3p	0	1.07589	M up vs IM
mmu-miR-147-5p	1	1	o change between M and IM
mmu-miR-1191	1	1	o change between M and IM
mmu-miR-466n-3p	0.888002	1.03407	M up vs IM
mmu-miR-7043-5p	0.995415	1.00056	M up vs IM
mmu-miR-1930-5p	0.679639	-1.08618	M down vs IM
mmu-miR-6947-3p	1	1	o change between M and IM
mmu-mir-5110	0.611672	-1.11066	M down vs IM
mmu-miR-7030-3p	0.611672	-1.11066	M down vs IM
mmu-miR-6957-3p	1	1	o change between M and IM
mmu-miR-466n-5p	1	1	o change between M and IM
mmu-miR-344e-3p	0.611672	-1.11066	M down vs IM
mmu-mir-7074	0.887499	-1.02718	M down vs IM
mmu-miR-196b-3p	1	1	o change between M and IM
mmu-miR-6912-5p	1	1	o change between M and IM
mmu-miR-138-2-3p	0.911187	1.02378	M up vs IM
mmu-mir-301a	0.298225	1.06229	M up vs IM
mmu-miR-203-3p	0.642324	-1.15244	M down vs IM
mmu-miR-1946b	0.521493	-1.10307	M down vs IM
mmu-let-7c-1-3p	0.859769	1.02378	M up vs IM
mmu-mir-3075	1	1	o change between M and IM
mmu-miR-6928-5p	0.884215	1.03105	M up vs IM
mmu-mir-125b-2	0.686895	-1.08618	M down vs IM
mmu-mir-125b-2	0.690801	-1.08486	M down vs IM
mmu-miR-149-5p	0.689804	1.0852	M up vs IM
mmu-miR-7a-2-3p	0.494078	-1.41649	M down vs IM
mmu-mir-6371	1	1	o change between M and IM
mmu-mir-7071	0.388418	-1.05424	M down vs IM
mmu-miR-15b-3p	0.508865	1.47123	M up vs IM
mmu-miR-380-5p	0.754937	1.09841	M up vs IM
mmu-mir-453	1	1	o change between M and IM
mmu-mir-664	0.843484	-1.11102	M down vs IM
mmu-miR-106a-5p	1	1	o change between M and IM

mmu-miR-7012-5p	1	1	o change between M and IM
mmu-mir-10b	0.361594	1.06229	M up vs IM
mmu-miR-7659-5p	0.865769	1.07589	M up vs IM
mmu-miR-6546-3p	0.809304	1.03105	M up vs IM
mmu-miR-7237-3p	0.64042	1.0744	M up vs IM
mmu-miR-7092-3p	0.869741	1.02378	M up vs IM
mmu-let-7a-5p	0.457999	-1.20089	M down vs IM
mmu-miR-875-3p	0.312146	1.1917	M up vs IM
mmu-miR-1843b-3p	0.701747	-1.08486	M down vs IM
mmu-miR-130b-3p	0.766668	-1.06373	M down vs IM
mmu-mir-124-1	0.72844	1.07589	M up vs IM
mmu-miR-92a-3p	0.0784677	-3.70019	M down vs IM
mmu-miR-7032-5p	0.733872	1.04349	M up vs IM
mmu-mir-8107	0	1.13886	M up vs IM
mmu-miR-23b-5p	0	1.34204	M up vs IM
mmu-miR-7232-5p	0	1.0852	M up vs IM
mmu-miR-7019-5p	0.364005	-1.29062	M down vs IM
mmu-miR-26a-2-3p	0.733152	1.07589	M up vs IM
mmu-mir-5615-1	0.250916	1.48397	M up vs IM
mmu-miR-466f-3p	0.371625	1.28214	M up vs IM
mmu-mir-7686	0.377144	-1.27623	M down vs IM
mmu-mir-3103	0.615573	-1.1199	M down vs IM
mmu-miR-376b-5p	1	1	o change between M and IM
mmu-miR-6345	0.5752	-1.13807	M down vs IM
mmu-mir-1895	0.924259	-1.01982	M down vs IM
mmu-miR-1901	1	1	o change between M and IM
mmu-miR-7116-3p	0.615573	-1.1199	M down vs IM
mmu-mir-466f-1	0.671701	-1.09752	M down vs IM
mmu-mir-351	1	1	o change between M and IM
mmu-mir-509	1	1	o change between M and IM
mmu-mir-466q	1	1	o change between M and IM
mmu-miR-697	1	1	o change between M and IM
mmu-miR-300-5p	0.838471	1.04349	M up vs IM
mmu-mir-28b	0.364005	-1.29062	M down vs IM
mmu-miR-6342	0.612502	1.07589	M up vs IM
mmu-miR-652-5p	0.793015	1.11357	M up vs IM
mmu-mir-3091	0.283593	-1.29002	M down vs IM
mmu-mir-6918	0.246819	1.34263	M up vs IM
mmu-miR-1955-3p	0.579836	-1.08486	M down vs IM
mmu-miR-181d-5p	0.878464	1.02378	M up vs IM
mmu-mir-1668	0.595227	-1.09389	M down vs IM
mmu-miR-3088-5p	0.595227	-1.09389	M down vs IM
mmu-miR-721	0.878464	1.02378	M up vs IM

mmu-miR-3081-5p	0.878464	1.02378	M up vs IM
mmu-miR-6936-3p	0.768129	-1.03965	M down vs IM
mmu-mir-718	0.437733	1.2414	M up vs IM
mmu-mir-6968	0.197893	1.07589	M up vs IM
mmu-miR-7087-3p	1	1	o change between M and IM
mmu-miR-3089-5p	0.319289	1.21096	M up vs IM
mmu-miR-7046-5p	0.804277	-1.16171	M down vs IM
mmu-miR-5124b	0.406564	-1.26355	M down vs IM
mmu-miR-126a-3p	0.430767	1.2414	M up vs IM
mmu-miR-214-3p	0.68456	1.09841	M up vs IM
mmu-miR-677-3p	1	1	o change between M and IM
mmu-mir-3569	1	1	o change between M and IM
mmu-miR-218-2-3p	0.832113	1.03105	M up vs IM
mmu-mir-7233	0.832113	1.03105	M up vs IM
mmu-miR-344c-5p	0.832113	1.03105	M up vs IM
mmu-miR-873a-3p	0.598564	-1.08618	M down vs IM
mmu-mir-8090	0.832113	1.03105	M up vs IM
mmu-miR-5127	0.812464	1.05439	M up vs IM
mmu-miR-3473c	0.828602	1.04945	M up vs IM
mmu-miR-1962	1	1	o change between M and IM
mmu-mir-761	1	1	o change between M and IM
mmu-miR-3086-3p	0.316032	-1.38801	M down vs IM
mmu-miR-125b-5p	0.331322	1.36324	M up vs IM
mmu-miR-7a-5p	0.446267	-1.2342	M down vs IM
mmu-miR-511-3p	0.722656	-1.08618	M down vs IM
mmu-miR-467e-5p	0.617798	-1.1293	M down vs IM
mmu-mir-7668	0.570881	-1.15244	M down vs IM
mmu-miR-222-5p	0.417852	1.2593	M up vs IM
mmu-miR-5620-3p	0.59929	-1.13807	M down vs IM
mmu-mir-324	0.617798	-1.1293	M down vs IM
mmu-miR-188-5p	0.722656	-1.08618	M down vs IM
mmu-miR-148b-5p	0.692735	-1.09752	M down vs IM
mmu-mir-680-2	0.706853	-1.09209	M down vs IM
mmu-miR-3060-5p	0.706853	-1.09209	M down vs IM
mmu-miR-3103-5p	0.706853	-1.09209	M down vs IM
mmu-mir-6991	0.831077	1.04945	M up vs IM
mmu-mir-6358	1	1	o change between M and IM
mmu-mir-6899	1	1	o change between M and IM
mmu-mir-7002	1	1	o change between M and IM
mmu-miR-376c-3p	1	1	o change between M and IM
mmu-mir-6335	0.380171	1.231	M up vs IM
mmu-miR-30c-2-3p	0.953075	-1.01115	M down vs IM
mmu-mir-3079	0	1.15281	M up vs IM

mmu-mir-7027	0	1.09334	M up vs IM
mmu-mir-7079	0	1.09334	M up vs IM
mmu-mir-195b	0.703572	1.09334	M up vs IM
mmu-mir-1898	0.273409	1.07589	M up vs IM
mmu-mir-20a	0.816896	1.03407	M up vs IM
mmu-miR-6390	0.9438	1.03694	M up vs IM
mmu-miR-293-3p	1	1	o change between M and IM
mmu-miR-148a-3p	1	1	o change between M and IM
mmu-mir-1970	0.843166	1.03105	M up vs IM
mmu-miR-1896	0.906522	1.03105	M up vs IM
mmu-mir-200b	0.655225	1.07589	M up vs IM
mmu-mir-7083	0.843166	1.03105	M up vs IM
mmu-miR-465b-5p	0.900425	1.03407	M up vs IM
mmu-miR-3473b	0.22868	-1.42013	M down vs IM
mmu-mir-149	0.777951	1.05613	M up vs IM
mmu-miR-6952-5p	0.802642	1.03694	M up vs IM
mmu-mir-6917	0.87589	-1.08486	M down vs IM
mmu-miR-7050-5p	0.609314	1.05613	M up vs IM
mmu-mir-6384	1	1	o change between M and IM
mmu-miR-3470a	0	1.09841	M up vs IM
mmu-miR-7225-5p	0.7779	1.07589	M up vs IM
mmu-mir-1843a	0.305137	1.43706	M up vs IM
mmu-miR-3064-3p	0.721619	-1.09209	M down vs IM
mmu-miR-5046	0.818283	1.05686	M up vs IM
mmu-mir-7090	0.740278	-1.08486	M down vs IM
mmu-miR-7071-5p	0.418983	1.27603	M up vs IM
mmu-miR-296-5p	0.617094	-1.13807	M down vs IM
mmu-miR-7036b-5p	0.655531	-1.1199	M down vs IM
mmu-miR-6935-3p	1	1	o change between M and IM
mmu-miR-1193-3p	1	1	o change between M and IM
mmu-miR-7019-3p	0.764294	1.07589	M up vs IM
mmu-miR-6925-5p	0.825586	1.05439	M up vs IM
mmu-mir-7023	0.840394	1.04945	M up vs IM
mmu-miR-6934-3p	0.357842	-1.34877	M down vs IM
mmu-miR-1905	0.617094	-1.13807	M down vs IM
mmu-mir-466b-2	0.828854	1.03407	M up vs IM
mmu-miR-7226-3p	0.828854	1.03407	M up vs IM
mmu-mir-3473b	0.914711	1.02045	M up vs IM
mmu-miR-6908-5p	1	1	o change between M and IM
mmu-miR-7679-5p	0.270799	-1.1199	M down vs IM
mmu-mir-883a	0.641891	-1.1293	M down vs IM
mmu-miR-6943-5p	0.727231	-1.09209	M down vs IM
mmu-mir-6769b	1	1	o change between M and IM

mmu-miR-669a-3p	0.642198	-1.08486	M down vs IM
mmu-miR-669o-3p	0.642198	-1.08486	M down vs IM
mmu-miR-7241-3p	0.403075	-1.30071	M down vs IM
mmu-miR-6966-5p	0.443641	1.2593	M up vs IM
mmu-miR-143-3p	0.843978	1.04945	M up vs IM
mmu-miR-3087-5p	1	1	o change between M and IM
mmu-miR-6413	1	1	o change between M and IM
mmu-miR-200a-5p	0.436726	1.26546	M up vs IM
mmu-miR-7010-3p	0.711161	1.09881	M up vs IM
mmu-miR-7077-3p	0	1.1015	M up vs IM
mmu-miR-6398	0	1.1494	M up vs IM
mmu-miR-7060-3p	0.614246	-1.09209	M down vs IM
mmu-miR-1946a	0.647223	-1.08618	M down vs IM
mmu-mir-20b	1	1	o change between M and IM
mmu-miR-698-5p	0.744433	-1.08618	M down vs IM
mmu-mir-210	0.685315	-1.11066	M down vs IM
mmu-mir-3970	0.744433	-1.08618	M down vs IM
mmu-miR-3059-3p	1	1	o change between M and IM
mmu-miR-3076-3p	0.653807	1.29102	M up vs IM
mmu-miR-7054-5p	0.83865	1.03407	M up vs IM
mmu-mir-7236	0.83865	1.03407	M up vs IM
mmu-mir-709	0.901178	1.02378	M up vs IM
mmu-mir-9-3	0.325255	1.30721	M up vs IM
mmu-miR-1950	0.386426	1.2414	M up vs IM
mmu-miR-503-3p	0.756456	1.06229	M up vs IM
mmu-mir-142b	0.280238	-1.31971	M down vs IM
mmu-mir-291b	1	1	o change between M and IM
mmu-mir-680-2	0.706853	-1.09209	M down vs IM
mmu-mir-6964	0.925496	1.05613	M up vs IM
mmu-miR-500-5p	0.538619	-1.1199	M down vs IM
mmu-miR-8112	0.191448	1.13239	M up vs IM
mmu-mir-7217	0.632984	-1.09209	M down vs IM
mmu-miR-5132-5p	0.845691	1.03407	M up vs IM
mmu-miR-758-5p	0.371307	1.23989	M up vs IM
mmu-mir-463	0.810646	1.07904	M up vs IM
mmu-mir-684-1	0	1.0852	M up vs IM
mmu-mir-684-2	0	1.0852	M up vs IM
mmu-miR-3062-5p	0.708826	1.3022	M up vs IM
mmu-miR-6399	0.754892	1.03407	M up vs IM
mmu-miR-301a-3p	0.623276	-1.15244	M down vs IM
mmu-miR-5103	0.686538	-1.1199	M down vs IM
mmu-miR-7684-5p	1	1	o change between M and IM
mmu-miR-669g	0.748314	-1.09209	M down vs IM

mmu-miR-490-5p	0.748314	-1.09209	M down vs IM
mmu-mir-1843a	0.856911	1.04945	M up vs IM
mmu-mir-1966	0.909485	1.02378	M up vs IM
mmu-mir-1963	0.756411	1.0683	M up vs IM
mmu-miR-450a-5p	0.352387	1.27507	M up vs IM
mmu-miR-135a-5p	0.872124	1.03105	M up vs IM
mmu-miR-1952	0.722656	-1.08618	M down vs IM
mmu-miR-7076-3p	0.842528	1.02378	M up vs IM
mmu-miR-7024-5p	0.5168	-1.13807	M down vs IM
mmu-let-7j	0.746811	1.03407	M up vs IM
mmu-miR-6973b-3p	0.875465	1.03105	M up vs IM
mmu-miR-503-5p	0.754012	1.06229	M up vs IM
mmu-miR-6980-3p	0.295777	-1.31971	M down vs IM
mmu-mir-344-2	0.410635	1.18966	M up vs IM
mmu-mir-7048	0.915786	1.03105	M up vs IM
mmu-miR-7655-3p	0.662251	-1.09209	M down vs IM
mmu-mir-6941	0.682591	1.0852	M up vs IM
mmu-miR-7010-5p	0.467638	1.24848	M up vs IM
mmu-miR-218-1-3p	0.0198654	1.07589	M up vs IM
mmu-miR-193b-3p	0.863902	1.03407	M up vs IM
mmu-miR-669b-3p	0.74501	1.06229	M up vs IM
mmu-miR-24-3p	0.196386	1.15068	M up vs IM
mmu-miR-24-1-5p	0.441179	1.14611	M up vs IM
mmu-mir-7044	0.713941	1.05613	M up vs IM
mmu-miR-694	1	1	o change between M and IM
mmu-miR-3965	0.75553	1.0744	M up vs IM
mmu-miR-6939-5p	0.702126	-1.09389	M down vs IM
mmu-mir-450a-2	0.23861	1.09841	M up vs IM
mmu-miR-7222-5p	0.702844	-1.1199	M down vs IM
mmu-miR-7242-3p	1	1	o change between M and IM
mmu-mir-3572	0.316898	1.32317	M up vs IM
mmu-mir-3963	1	1	o change between M and IM
mmu-miR-181c-5p	0.762163	-1.09209	M down vs IM
mmu-mir-297b	1	1	o change between M and IM
mmu-miR-6996-5p	1	1	o change between M and IM
mmu-miR-494-3p	0.712817	-1.11501	M down vs IM
mmu-mir-5615-1	0.234517	1.53005	M up vs IM
mmu-mir-412	0.341093	1.31851	M up vs IM
mmu-miR-190a-5p	0.344071	1.31466	M up vs IM
mmu-miR-375-5p	0.332143	1.33053	M up vs IM
mmu-miR-5625-3p	0.703022	-1.08618	M down vs IM
mmu-mir-8118	0.729187	-1.07721	M down vs IM
mmu-miR-6350	0.714977	1.08203	M up vs IM

mmu-miR-1971	0.733152	1.07589	M up vs IM
mmu-mir-3082	0.357289	1.2984	M up vs IM
mmu-mir-7663	0.403561	1.25022	M up vs IM
mmu-miR-539-5p	0.882771	1.03105	M up vs IM
mmu-mir-669b	0.667437	1.06229	M up vs IM
mmu-mir-669b	0.667437	1.06229	M up vs IM
mmu-mir-6988	0.754278	1.07235	M up vs IM
mmu-mir-466h	0.295674	-1.34877	M down vs IM
mmu-mir-186	0	-1.14875	M down vs IM
mmu-mir-101c	0.372952	1.27507	M up vs IM
mmu-mir-467c	0.376128	1.2773	M up vs IM
mmu-mir-6996	0.547544	1.215	M up vs IM
mmu-miR-344i	0.724773	1.07589	M up vs IM
mmu-miR-1964-3p	0.87185	1.03407	M up vs IM
mmu-miR-1191b-3p	0.705828	1.0852	M up vs IM
mmu-miR-6383	0.723755	1.07904	M up vs IM
mmu-miR-30c-5p	0.741327	-1.06865	M down vs IM
mmu-mir-6375	0.844108	1.03931	M up vs IM
mmu-miR-6984-5p	0.913985	1.0683	M up vs IM
mmu-mir-7010	0.555339	-1.09209	M down vs IM
mmu-mir-124-1	0.677712	1.09334	M up vs IM
mmu-miR-3082-5p	0.289803	1.46959	M up vs IM
mmu-miR-6951-5p	0.687958	1.06229	M up vs IM
mmu-miR-1964-5p	0.687958	1.06229	M up vs IM
mmu-miR-7037-5p	0.801471	-1.03718	M down vs IM
mmu-miR-598-3p	1	1	o change between M and IM
mmu-miR-6371	0.71361	1.11563	M up vs IM
mmu-miR-7238-3p	0.225896	1.49974	M up vs IM
mmu-miR-5709-5p	1	1	o change between M and IM
mmu-mir-7239	0.332882	1.32951	M up vs IM
mmu-miR-362-5p	0.85386	-1.1293	M down vs IM
mmu-mir-487b	0.745648	1.05613	M up vs IM
mmu-mir-3057	0.736139	1.07589	M up vs IM
mmu-miR-8103	0.722656	-1.08618	M down vs IM
mmu-miR-96-5p	0.725328	1.0852	M up vs IM
mmu-miR-344f-3p	0.891459	1.03105	M up vs IM
mmu-mir-365-1	1	1	o change between M and IM
mmu-mir-2137	1	1	o change between M and IM
mmu-miR-6973b-5p	0.853132	1.03931	M up vs IM
mmu-mir-1912	0.715638	1.09031	M up vs IM
mmu-miR-702-5p	0.729256	1.0852	M up vs IM
mmu-miR-15a-5p	0.889678	1.03105	M up vs IM
mmu-miR-448-3p	0.489861	-1.22268	M down vs IM

mmu-miR-1264-5p	1	1	o change between M and IM
mmu-mir-342	0	1.17837	M up vs IM
mmu-miR-326-5p	0.726229	1.08204	M up vs IM
mmu-mir-3068	0.706853	-1.09209	M down vs IM
mmu-miR-484	0.376927	1.3022	M up vs IM
mmu-mir-466b-3	0.706853	-1.09209	M down vs IM
mmu-miR-6365	0.706853	-1.09209	M down vs IM
mmu-miR-208a-3p	0.725328	1.0852	M up vs IM
mmu-mir-7014	0.725328	1.0852	M up vs IM
mmu-miR-6977-5p	0.283903	1.32999	M up vs IM
mmu-miR-5616-3p	0.944337	1.04349	M up vs IM
mmu-mir-138-2	0.896702	-1.08486	M down vs IM
mmu-mir-138-2	0.896702	-1.08486	M down vs IM
mmu-mir-7030	0.503502	1.20489	M up vs IM
mmu-miR-431-5p	0.897601	1.03105	M up vs IM
mmu-miR-883a-3p	0.897601	1.03105	M up vs IM
mmu-miR-451b	0.764294	1.07589	M up vs IM
mmu-mir-7665	0.692705	-1.1038	M down vs IM
mmu-miR-5133	0.897601	1.03105	M up vs IM
mmu-mir-6972	0.699465	-1.09209	M down vs IM
mmu-miR-681	0.841306	1.05613	M up vs IM
mmu-miR-6999-5p	0.739654	-1.08001	M down vs IM
mmu-miR-7056-5p	0.833579	1.06229	M up vs IM
mmu-miR-1953	0.339608	1.2607	M up vs IM
mmu-miR-7075-5p	0.894009	-1.08618	M down vs IM
mmu-miR-467a-3p	0.423213	1.27878	M up vs IM
mmu-mir-6357	0.899906	1.03105	M up vs IM
mmu-mir-6379	0.752962	1.08203	M up vs IM
mmu-miR-874-3p	0.541393	1.14089	M up vs IM
mmu-mir-330	1	1	o change between M and IM
mmu-mir-6344	0.837528	-1.0504	M down vs IM
mmu-miR-1948-3p	0.900854	1.03105	M up vs IM
mmu-miR-3099-3p	0.900854	1.03105	M up vs IM
mmu-mir-181b-1	0.808464	1.06229	M up vs IM
mmu-miR-6941-3p	0.822735	1.04805	M up vs IM
mmu-miR-3090-5p	0.172377	1.99152	M up vs IM
mmu-miR-6979-5p	0.376226	1.215	M up vs IM
mmu-miR-33-3p	0.606024	-1.09752	M down vs IM
mmu-miR-1983	0.634894	1.07589	M up vs IM
mmu-let-7a-2	0.890515	1.03407	M up vs IM
mmu-mir-7045	0.577593	1.08268	M up vs IM
mmu-mir-1950	0.878934	1.03694	M up vs IM
mmu-miR-7028-3p	0.878934	1.03694	M up vs IM

mmu-miR-8120	0.731549	1.08821	M up vs IM
mmu-miR-7033-3p	0.891549	1.03407	M up vs IM
mmu-mir-669c	0.881641	1.03694	M up vs IM
mmu-miR-6983-5p	0.89057	1.03407	M up vs IM
mmu-miR-409-5p	0.325499	1.15624	M up vs IM
mmu-miR-767	0.89201	-1.01405	M down vs IM
mmu-mir-5098	0.715045	-1.09209	M down vs IM
mmu-miR-194-2-3p	0.531635	1.11636	M up vs IM
mmu-mir-1968	0.637397	1.173	M up vs IM
mmu-miR-3083-5p	0.819556	1.06229	M up vs IM
mmu-mir-6363	0.656449	-1.31971	M down vs IM
mmu-miR-669h-5p	0.481979	-1.08486	M down vs IM
mmu-miR-668-3p	0.637197	1.27293	M up vs IM
mmu-miR-7231-3p	?	1	o change between M and IM
mmu-miR-3073a-3p	0.756456	1.06229	M up vs IM
mmu-miR-32-5p	0.334122	1.2961	M up vs IM
mmu-mir-6337	0.682591	1.0852	M up vs IM
mmu-miR-222-3p	0.489944	1.32837	M up vs IM
mmu-miR-466b-5p	0.601896	1.0852	M up vs IM
mmu-miR-466o-5p	0.601896	1.0852	M up vs IM
mmu-miR-615-5p	0.913559	1.06229	M up vs IM
mmu-miR-6408	1	1	o change between M and IM
mmu-miR-717	1	1	o change between M and IM
mmu-mir-6405	1	1	o change between M and IM
mmu-miR-6366	0.271724	1.08531	M up vs IM
mmu-mir-6977	1	1	o change between M and IM
mmu-miR-7083-3p	0.684853	1.07589	M up vs IM
mmu-mir-679	0.111944	1.231	M up vs IM
mmu-miR-191-3p	0.0581234	1.49678	M up vs IM
mmu-miR-6919-3p	0.144776	1.173	M up vs IM
mmu-mir-5098	0.736272	-1.09209	M down vs IM
mmu-mir-7225	0.775473	1.06229	M up vs IM
mmu-miR-6896-3p	0.775473	1.06229	M up vs IM
mmu-miR-6940-5p	0.623139	1.0852	M up vs IM
mmu-mir-466n	0.913911	1.03105	M up vs IM
mmu-miR-6947-5p	0	1.03105	M up vs IM
mmu-miR-883a-5p	0.851127	1.06229	M up vs IM
mmu-miR-7021-3p	0.107672	1.2414	M up vs IM
mmu-mir-764	0.688503	1.173	M up vs IM
mmu-mir-451a	0.64111	1.0852	M up vs IM
mmu-mir-6939	0.64111	1.0852	M up vs IM
mmu-mir-1969	0.869009	1.05613	M up vs IM
mmu-miR-7217-5p	0.172561	1.18966	M up vs IM

mmu-miR-150-5p	0.729291	-1.1451	M down vs IM
mmu-miR-204-5p	1	1	o change between M and IM
mmu-miR-7028-5p	0.590127	-1.25175	M down vs IM
mmu-mir-8113	0.828382	-1.08618	M down vs IM
mmu-miR-7241-5p	1	1	o change between M and IM
mmu-miR-7002-5p	0.913044	1.05439	M up vs IM
mmu-miR-470-3p	0.866499	1.05613	M up vs IM
mmu-miR-193a-3p	0.415437	1.21712	M up vs IM
mmu-mir-1896	0.641075	1.11481	M up vs IM
mmu-mir-7072	1	1	o change between M and IM
mmu-mir-216b	0.705828	1.0852	M up vs IM
mmu-mir-8095	0	1.03407	M up vs IM
mmu-miR-7212-5p	0.0710503	1.39025	M up vs IM
mmu-miR-7678-5p	0.733152	1.07589	M up vs IM
mmu-mir-6244	0.332143	1.33053	M up vs IM
mmu-mir-5100	0.0834591	1.58093	M up vs IM
mmu-miR-293-5p	0.299903	-1.08618	M down vs IM
mmu-miR-3113-5p	0.940595	1.03105	M up vs IM
mmu-let-7d-3p	0.836282	-1.08618	M down vs IM
mmu-mir-6932	0.115514	1.18966	M up vs IM
mmu-miR-135a-1-3p	1	1	o change between M and IM
mmu-mir-7004	0.891914	-1.08618	M down vs IM
mmu-miR-7038-3p	1	1	o change between M and IM
mmu-mir-7672	0.754562	-1.13766	M down vs IM
mmu-mir-466k	1	1	o change between M and IM
mmu-mir-6400	0	1.231	M up vs IM
mmu-mir-7076	0	1.2368	M up vs IM
mmu-miR-5131	0	1.42234	M up vs IM
mmu-miR-693-3p	0	1.7407	M up vs IM
mmu-miR-883b-3p	0	1.22401	M up vs IM
mmu-miR-217-3p	0	1.173	M up vs IM
mmu-mir-7049	0.679565	1.23668	M up vs IM
mmu-miR-1932	0.9462	1.02378	M up vs IM
mmu-miR-6985-3p	0.929604	1.03694	M up vs IM
mmu-miR-423-5p	0.897662	1.15821	M up vs IM
mmu-miR-599	0.063636	-1.08618	M down vs IM
mmu-miR-7218-5p	0.705828	1.0852	M up vs IM
mmu-miR-6382	0.946946	1.03105	M up vs IM
mmu-mir-222	0.410692	1.43367	M up vs IM
mmu-miR-5108	1	1	o change between M and IM
mmu-mir-5624	0.818185	-1.08486	M down vs IM
mmu-miR-6372	0.8685	-1.0592	M down vs IM
mmu-mir-6339	0.591767	-1.29062	M down vs IM

mmu-miR-344f-5p	0.796303	-1.1199	M down vs IM
mmu-miR-140-5p	1	1	o change between M and IM
mmu-mir-293	1	1	o change between M and IM
mmu-miR-6388	0.94591	-1.04712	M down vs IM
mmu-miR-7088-5p	1	1	o change between M and IM
mmu-miR-7667-5p	1	1	o change between M and IM
mmu-miR-6420	0.923492	1.03407	M up vs IM
mmu-miR-6926-3p	0.83278	1.06229	M up vs IM
mmu-mir-6369	1	1	o change between M and IM
mmu-mir-6383	1	1	o change between M and IM
mmu-miR-483-3p	1	1	o change between M and IM
mmu-mir-1a-2	0	1.05613	M up vs IM
mmu-mir-450a-2	0	1.18966	M up vs IM
mmu-mir-680-3	0	1.2414	M up vs IM
mmu-mir-590	0	1.18966	M up vs IM
mmu-mir-449b	0	1.47123	M up vs IM
mmu-mir-544	0	1.18966	M up vs IM
mmu-mir-1953	0	1.18966	M up vs IM
mmu-mir-6931	0	1.24848	M up vs IM
mmu-mir-7015	0	1.18966	M up vs IM
mmu-mir-7086	0	1.08395	M up vs IM
mmu-mir-7671	0	1.18966	M up vs IM
mmu-miR-145a-5p	0	1.2414	M up vs IM
mmu-miR-201-5p	0	1.18966	M up vs IM
mmu-miR-542-3p	0	1.18966	M up vs IM
mmu-miR-27a-5p	0	1.23441	M up vs IM
mmu-miR-582-5p	0	1.18966	M up vs IM
mmu-miR-1902	0	1.18966	M up vs IM
mmu-miR-3108-3p	0	1.24848	M up vs IM
mmu-miR-1198-3p	0	1.07113	M up vs IM
mmu-miR-30f	0	1.47123	M up vs IM
mmu-miR-6960-5p	0	1.18966	M up vs IM
mmu-miR-6978-5p	0	1.25437	M up vs IM
mmu-miR-6982-3p	0	1.25437	M up vs IM
mmu-miR-7683-3p	0	1.07589	M up vs IM
mmu-miR-1668	0	-1.10931	M down vs IM
mmu-let-7g-3p	0.912724	1.07589	M up vs IM
mmu-miR-7029-3p	0.120531	1.173	M up vs IM
mmu-miR-7227-5p	0.120531	1.173	M up vs IM
mmu-mir-880	0.0942083	1.28108	M up vs IM
mmu-miR-1197-5p	0.0942083	1.28108	M up vs IM
mmu-mir-669k	0.0668116	1.41986	M up vs IM
mmu-miR-7053-5p	1	1	o change between M and IM

mmu-miR-412-5p	0.142315	1.173	M up vs IM
mmu-mir-6356	0.0960031	1.27507	M up vs IM
mmu-miR-6994-5p	1	1	o change between M and IM
mmu-miR-6963-3p	0.103245	1.215	M up vs IM
mmu-miR-6368	0.100948	1.29145	M up vs IM
mmu-miR-5623-3p	0.935165	1.03105	M up vs IM
mmu-miR-7060-5p	0.935164	1.03105	M up vs IM
mmu-mir-1947	0.361869	-1.06535	M down vs IM
mmu-miR-145b	0.865753	1.05613	M up vs IM
mmu-miR-6236	0.399003	1.42234	M up vs IM
mmu-miR-7029-5p	0.10116	1.21997	M up vs IM
mmu-mir-344e	1	1	o change between M and IM
mmu-mir-18a	0.989703	1.00594	M up vs IM
mmu-miR-468-3p	0.206009	1.20463	M up vs IM
mmu-mir-669l	0.519295	1.16067	M up vs IM
mmu-miR-7020-3p	0.940243	1.03694	M up vs IM
mmu-miR-216a-3p	0.176911	1.10092	M up vs IM
mmu-miR-338-3p	1	1	o change between M and IM
mmu-mir-669i	0.855973	1.07589	M up vs IM
mmu-mir-7682	0	1.06229	M up vs IM
mmu-miR-28a-3p	0.105852	1.28874	M up vs IM
mmu-miR-511-5p	0.150469	1.54611	M up vs IM
mmu-miR-296-3p	0.344599	1.74831	M up vs IM
mmu-miR-7048-5p	0.387356	1.05345	M up vs IM
mmu-miR-3102-5p.2-5p	1	1	o change between M and IM
mmu-miR-8104	0.83092	-1.11066	M down vs IM
mmu-mir-320	0.374971	-1.09977	M down vs IM
mmu-mir-1188	0.956295	1.02378	M up vs IM
mmu-mir-1191b	0	-1.34031	M down vs IM
mmu-miR-129-2-3p	0	1.215	M up vs IM
mmu-mir-30b	0	1.32841	M up vs IM
mmu-mir-1199	0	1.08491	M up vs IM
mmu-mir-6340	0	1.215	M up vs IM
mmu-mir-6341	0	1.27507	M up vs IM
mmu-mir-6343	0	1.215	M up vs IM
mmu-mir-6906	0	1.215	M up vs IM
mmu-mir-6981	0	1.215	M up vs IM
mmu-miR-217-5p	0	1.215	M up vs IM
mmu-miR-678	0	1.06104	M up vs IM
mmu-miR-3084-5p	0	1.50256	M up vs IM
mmu-miR-6377	0	1.215	M up vs IM
mmu-miR-7023-3p	0	1.27507	M up vs IM
mmu-miR-7027-5p	0	1.215	M up vs IM

mmu-miR-7689-5p	0	1.26784	M up vs IM
mmu-miR-8118	0	1.26784	M up vs IM
mmu-mir-93	0	1.215	M up vs IM
mmu-mir-8103	0.123152	1.2414	M up vs IM
mmu-mir-3077	0.022505	-1.08618	M down vs IM
mmu-mir-6952	0.83679	-1.08618	M down vs IM
mmu-mir-31	0	1.21712	M up vs IM
mmu-miR-7686-3p	0	1.21712	M up vs IM
mmu-mir-1940	0.136564	-1.2481	M down vs IM
mmu-miR-7215-5p	0.887236	1.0323	M up vs IM
mmu-miR-125a-5p	0.319048	3.75608	M up vs IM
mmu-miR-369-5p	0	1.21997	M up vs IM
mmu-mir-340	0.552016	1.05613	M up vs IM
mmu-mir-1949	0.619902	-1.26155	M down vs IM
mmu-mir-7b	0.943195	1.03105	M up vs IM
mmu-miR-3092-5p	0.629935	-1.25175	M down vs IM
mmu-miR-7683-5p	0.855668	1.08203	M up vs IM
mmu-miR-574-3p	0.843274	1.08967	M up vs IM
mmu-miR-434-5p	0.849336	-1.08618	M down vs IM
mmu-miR-3471	0.850507	1.0852	M up vs IM
mmu-mir-6345	0.885064	1.05613	M up vs IM
mmu-mir-6538	0.937796	1.03407	M up vs IM
mmu-miR-6405	0.938777	1.03407	M up vs IM
mmu-miR-8108	1	1	o change between M and IM
mmu-mir-6953	0.803245	1.0852	M up vs IM
mmu-miR-878-5p	0.897791	1.06229	M up vs IM
mmu-mir-28a	0	1.23503	M up vs IM
mmu-mir-2183	0	1.55528	M up vs IM
mmu-mir-6413	0	1.2961	M up vs IM
mmu-mir-8119	0	1.23503	M up vs IM
mmu-miR-495-3p	0	1.2961	M up vs IM
mmu-miR-759	0	1.23503	M up vs IM
mmu-miR-21a-3p	0	1.23503	M up vs IM
mmu-miR-3105-5p	0	1.23503	M up vs IM
mmu-miR-378a-5p	0.879923	-1.13959	M down vs IM
mmu-mir-1945	0.12243	-1.17003	M down vs IM
mmu-miR-1981-5p	0.769961	1.17463	M up vs IM
mmu-miR-719	0.225113	1.68566	M up vs IM
mmu-let-7k	0.946946	1.03105	M up vs IM
mmu-miR-1249-3p	0	1.2414	M up vs IM
mmu-miR-3080-5p	0	1.53521	M up vs IM
mmu-miR-3473d	0	1.2414	M up vs IM
mmu-miR-8099	0	1.2414	M up vs IM

mmu-miR-135b-5p	0.860099	1.0852	M up vs IM
mmu-miR-7684-3p	0.117579	-1.06856	M down vs IM
mmu-miR-7003-5p	0.0309686	1.32317	M up vs IM
mmu-miR-6904-5p	0.8426	1.07589	M up vs IM
mmu-miR-376b-3p	0.56774	1.28874	M up vs IM
mmu-miR-290a-5p	0.316262	1.63221	M up vs IM
mmu-miR-7034-3p	0.14407	-1.09209	M down vs IM
mmu-miR-221-5p	0.847351	1.07589	M up vs IM
mmu-miR-207	0.490598	-1.27623	M down vs IM
mmu-miR-130a-3p	0.714073	1.05613	M up vs IM
mmu-miR-3109-5p	0.825383	1.0852	M up vs IM
mmu-miR-8115	0.102454	-1.25175	M down vs IM
mmu-mir-7220	0.474653	1.20463	M up vs IM
mmu-miR-592-3p	0.429596	1.215	M up vs IM
mmu-mir-7218	1	1	o change between M and IM
mmu-miR-6971-3p	0.326107	1.2414	M up vs IM
mmu-miR-670-5p	0.415437	1.21712	M up vs IM
mmu-miR-34b-5p	0.890827	1.0852	M up vs IM
mmu-miR-7243-3p	0.232628	1.49678	M up vs IM
mmu-miR-6961-5p	0.840103	-1.1293	M down vs IM
mmu-miR-6985-5p	0.768153	1.17984	M up vs IM
mmu-miR-7210-3p	0.445997	1.21272	M up vs IM
mmu-mir-7053	0.89086	1.07589	M up vs IM
mmu-miR-6979-3p	0.536161	1.27507	M up vs IM
mmu-miR-5626-5p	0.956714	1.03407	M up vs IM
mmu-mir-3090	0	1.33053	M up vs IM
mmu-mir-7670	0	1.27507	M up vs IM
mmu-miR-376a-5p	0	1.33053	M up vs IM
mmu-mir-6954	0	1.27507	M up vs IM
mmu-mir-465c-1	0.33974	1.32028	M up vs IM
mmu-mir-465c-2	0.33974	1.32028	M up vs IM
mmu-miR-322-5p	0.46042	1.27507	M up vs IM
mmu-mir-1193	0.744567	1.07589	M up vs IM
mmu-miR-100-3p	0.924155	1.05613	M up vs IM
mmu-miR-18a-5p	1	1	o change between M and IM
mmu-mir-132	0.695296	1.21712	M up vs IM
mmu-miR-674-5p	0.318202	1.52533	M up vs IM
mmu-miR-3572-5p	0.244586	1.76543	M up vs IM
mmu-miR-666-5p	0.761202	1.18966	M up vs IM
mmu-miR-15b-5p	0.234489	-1.62055	M down vs IM
mmu-miR-703	0.179	1.76543	M up vs IM
mmu-miR-1306-5p	0.198428	1.6645	M up vs IM
mmu-mir-7029	0.294276	1.39025	M up vs IM

mmu-miR-7656-5p	0.94779	1.04321	M up vs IM
mmu-mir-146b	0.957283	1.03105	M up vs IM
mmu-mir-137	0.373215	1.215	M up vs IM
mmu-miR-669f-3p	0.512413	1.21997	M up vs IM
mmu-miR-342-5p	0.85386	-1.1293	M down vs IM
mmu-miR-7648-5p	1	1	o change between M and IM
mmu-miR-6938-3p	0.955983	-1.02806	M down vs IM
mmu-miR-184-3p	0.412565	1.30673	M up vs IM
mmu-miR-3110-3p	0.563993	1.54197	M up vs IM
mmu-mir-326	0.538064	1.40594	M up vs IM
mmu-miR-532-5p	0.282676	1.36324	M up vs IM
mmu-mir-8093	0.256086	1.51804	M up vs IM
mmu-miR-6375	0.235751	1.47123	M up vs IM
mmu-miR-106b-3p	0	1.3022	M up vs IM
mmu-miR-471-5p	1	1	o change between M and IM
mmu-miR-126a-5p	1	1	o change between M and IM
mmu-miR-1843a-5p	0.885985	1.06229	M up vs IM
mmu-miR-122-5p	0.886046	-1.10178	M down vs IM
mmu-mir-6402	0.460363	1.24146	M up vs IM
mmu-mir-361	0.681961	-1.30796	M down vs IM
mmu-mir-6915	0.332695	1.23503	M up vs IM
mmu-miR-432	0.351995	1.28408	M up vs IM
mmu-miR-7116-5p	0.959733	1.03105	M up vs IM
mmu-mir-451a	0.30712	1.2414	M up vs IM
mmu-mir-181a-2	0.898829	-1.08486	M down vs IM
mmu-miR-1298-5p	0.703022	-1.08618	M down vs IM
mmu-mir-17	0.498462	-1.23629	M down vs IM
mmu-miR-15a-3p	0.409125	1.26586	M up vs IM
mmu-mir-433	0.0923111	1.3022	M up vs IM
mmu-mir-678	0.72303	1.08604	M up vs IM
mmu-mir-883b	0.470705	1.215	M up vs IM
mmu-miR-6917-3p	0.751272	1.07589	M up vs IM
mmu-mir-128-1	0.467875	1.21712	M up vs IM
mmu-miR-362-3p	0.80868	-1.05219	M down vs IM
mmu-miR-133b-5p	0.236865	1.27507	M up vs IM
mmu-miR-543-5p	0.411888	1.28332	M up vs IM
mmu-mir-1938	0.873211	1.03969	M up vs IM
mmu-miR-466i-3p	0.49692	1.215	M up vs IM
mmu-miR-191-5p	0.29029	1.10866	M up vs IM
mmu-miR-219c-3p	0.353624	1.30278	M up vs IM
mmu-mir-6963	0.913611	1.07589	M up vs IM
mmu-mir-7068	0.986621	-1.00957	M down vs IM
mmu-miR-17-3p	1	1	o change between M and IM

mmu-miR-34c-5p	0.0508318	1.18966	M up vs IM
mmu-miR-7048-3p	0	1.68566	M up vs IM
mmu-miR-590-5p	0.643909	1.18966	M up vs IM
mmu-miR-711	0.649072	1.46996	M up vs IM
mmu-miR-93-5p	0.698759	-1.24478	M down vs IM
mmu-miR-194-5p	0.766096	1.0852	M up vs IM
mmu-miR-5098	?	1	o change between M and IM
mmu-miR-6378	0.391574	1.35657	M up vs IM
mmu-miR-30d-5p	0.444249	1.25198	M up vs IM
mmu-miR-483-5p	0.0252878	1.215	M up vs IM
mmu-mir-883a	0.665183	1.18966	M up vs IM
mmu-miR-680	0.75918	1.07777	M up vs IM
mmu-miR-669c-3p	0.6007	1.25437	M up vs IM
mmu-miR-7230-3p	0.554527	1.30673	M up vs IM
mmu-miR-5129-3p	0.610345	1.215	M up vs IM
mmu-mir-224	0.607667	1.21712	M up vs IM
mmu-miR-299a-3p	0	1.40191	M up vs IM
mmu-miR-7227-3p	0	1.47123	M up vs IM
mmu-miR-7040-5p	0.55796	1.2414	M up vs IM
mmu-miR-133c	0.864534	1.06759	M up vs IM
mmu-miR-3061-5p	0.354103	1.3022	M up vs IM
mmu-mir-106a	0.0589374	1.40594	M up vs IM
mmu-miR-7016-3p	0.756349	1.09334	M up vs IM
mmu-miR-465c-5p	0.415099	1.19113	M up vs IM
mmu-miR-361-5p	0.116961	-1.21118	M down vs IM
mmu-miR-320-3p	0.101759	1.40293	M up vs IM
mmu-miR-7685-3p	0.250959	1.36467	M up vs IM
mmu-mir-383	0.633826	1.26784	M up vs IM
mmu-miR-27a-3p	0.918206	1.05316	M up vs IM
mmu-miR-6969-3p	0.205904	1.40191	M up vs IM
mmu-miR-7666-5p	0	1.77837	M up vs IM
mmu-miR-7012-3p	0.758078	1.21712	M up vs IM
mmu-mir-543	0.283575	1.49774	M up vs IM
mmu-miR-7016-5p	0.205775	1.47123	M up vs IM
mmu-miR-7036-5p	1	1	o change between M and IM
mmu-miR-24-2-5p	0.388367	1.27507	M up vs IM
mmu-miR-146a-5p	0.739951	1.17168	M up vs IM
mmu-miR-151-3p	0.899647	1.0852	M up vs IM
mmu-miR-34b-3p	0.440557	1.42234	M up vs IM
mmu-miR-181a-1-3p	0.220507	1.57594	M up vs IM
mmu-miR-181a-5p	0.488513	1.36735	M up vs IM
mmu-miR-17-5p	0.0164275	-1.28898	M down vs IM
mmu-miR-185-5p	0.187639	1.43996	M up vs IM

mmu-mir-212	0.554312	1.29224	M up vs IM
mmu-miR-324-5p	0.176658	1.0852	M up vs IM
mmu-miR-23a-3p	0.420741	-1.17054	M down vs IM
mmu-miR-5128	0.651182	1.32975	M up vs IM
mmu-miR-652-3p	0.335418	1.81602	M up vs IM
mmu-let-7g-5p	0.962957	1.03407	M up vs IM
mmu-miR-16-5p	0.44405	1.10211	M up vs IM
mmu-miR-7221-5p	0.914417	1.07993	M up vs IM
mmu-miR-140-3p	0.0947681	2.12434	M up vs IM
mmu-miR-486-5p	1	1	o change between M and IM
mmu-miR-3107-5p	1	1	o change between M and IM
mmu-miR-425-5p	0.719576	-1.3017	M down vs IM
mmu-miR-2137	0.158565	1.92478	M up vs IM
mmu-miR-1940	0.854931	1.23503	M up vs IM
mmu-miR-20a-5p	0.418124	-1.32211	M down vs IM
mmu-miR-106b-5p	0.136059	1.17688	M up vs IM
mmu-miR-146b-5p	0.593372	1.25733	M up vs IM
mmu-miR-378d	0.881455	1.13839	M up vs IM
mmu-miR-378b	0.200168	1.30466	M up vs IM
mmu-miR-342-3p	0.481539	1.18141	M up vs IM
mmu-miR-181b-5p	0.611171	1.85542	M up vs IM
mmu-miR-212-3p	0.487973	1.32218	M up vs IM
mmu-miR-8101	0.156794	1.75468	M up vs IM
mmu-miR-7052-5p	0.632762	1.03407	M up vs IM
mmu-miR-378a-3p	0.11817	1.91411	M up vs IM
mmu-miR-132-3p	0	-1.57562	M down vs IM
mmu-miR-378c	0.898046	1.09841	M up vs IM
mmu-miR-1224-5p	0.0843706	4.40381	M up vs IM
mmu-miR-221-3p	0.0999923	3.09016	M up vs IM
